# Efficient cleavage of tertiary amide bonds *via* radical–polar crossover using a copper(ii) bromide/Selectfluor hybrid system†

**DOI:** 10.1039/d0sc05137c

**Published:** 2020-10-14

**Authors:** Zhe Wang, Akira Matsumoto, Keiji Maruoka

**Affiliations:** Graduate School of Pharmaceutical Sciences, Kyoto University Sakyo Kyoto 606-8501 Japan maruoka.keiji.4w@kyoto-u.ac.jp; School of Chemical Engineering and Light Industry, Guangdong University of Technology Guangzhou 510006 China

## Abstract

A novel approach for the efficient cleavage of the amide bonds in tertiary amides is reported. Based on the selective radical abstraction of a benzylic hydrogen atom by a CuBr_2_/Selectfluor hybrid system followed by a selective cleavage of an N–C bond, an acyl fluoride intermediate is formed. This intermediate may then be derivatized in a one-pot fashion. The reaction proceeds under mild conditions and exhibits a broad substrate scope with respect to the tertiary amide moiety as well as to nitrogen, oxygen, and carbon nucleophiles for the subsequent derivatization. Mechanistic studies suggest that the present reaction proceeds *via* a radical–polar crossover process that involves benzylic carbon radicals generated by the selective radical abstraction of a benzylic hydrogen atom by the CuBr_2_/Selectfluor hybrid system. Furthermore, a synthetic application of this method for the selective cleavage of peptides is described.

## Abstract 2

A novel approach for the efficient cleavage of the amide bonds in tertiary amides is reported.

## Graphical abstract

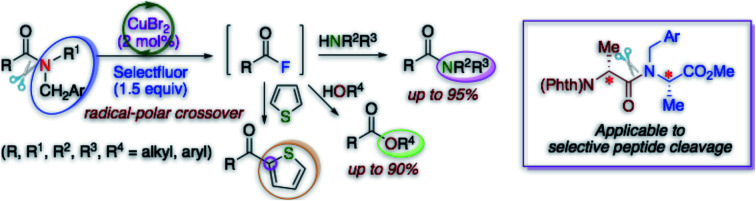

## Introduction

**Fig. 1:**
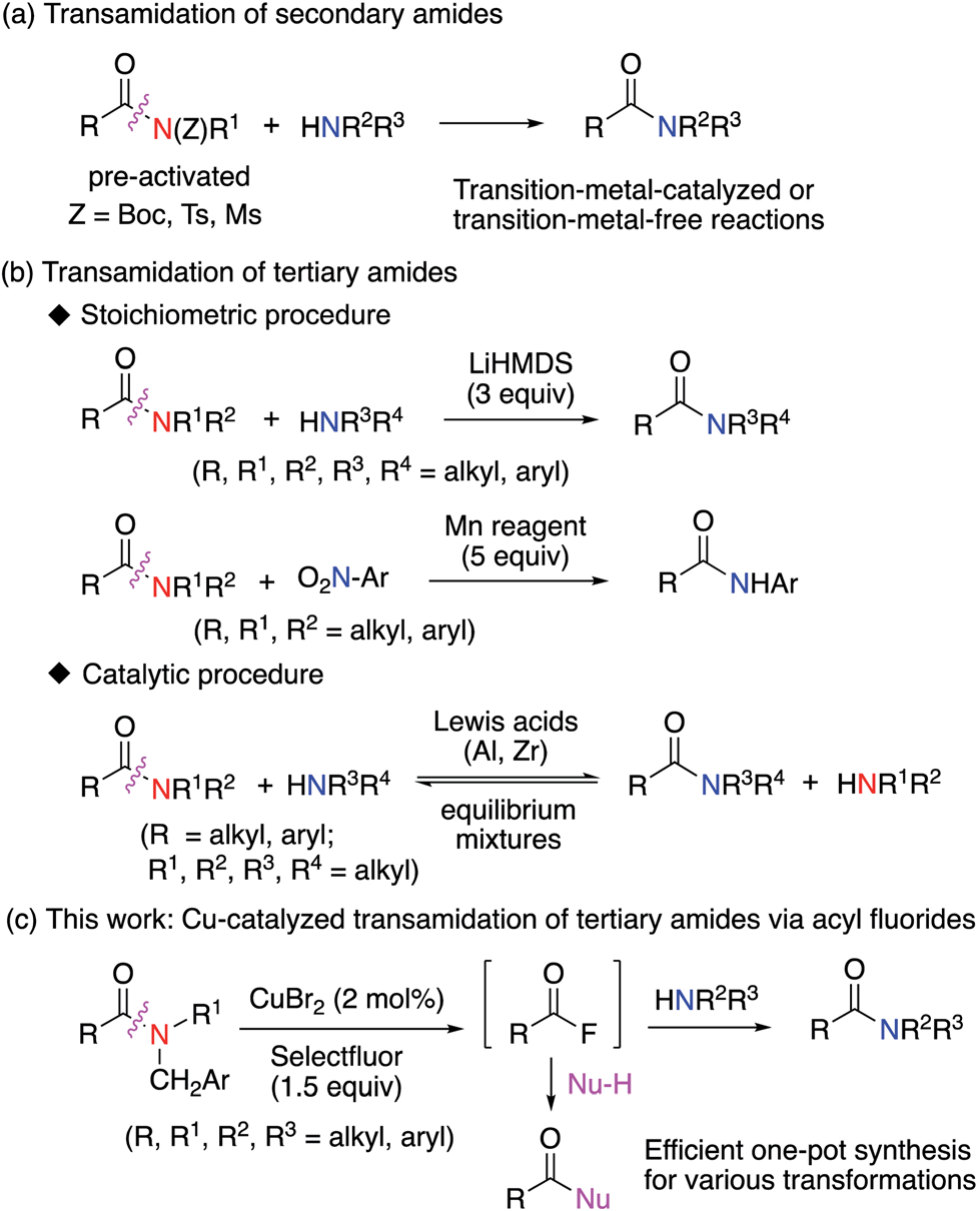
Transamidation of secondary and tertiary amides.

Organic amide groups are ubiquitous in nature, particularly in peptides and proteins.^1^ They are also widely used in the chemical and pharmaceutical industry for manufacturing of fine chemicals, polyamide plastics, pesticides, and medicines.^2–4^ Due to the resonance stabilization of amide groups, the amide N–C bond is relatively stable and unreactive. Accordingly, the selective cleavage of such unreactive amide N–C bonds is highly challenging in organic synthesis.^4–6^ Indeed, various approaches for the catalytic transamidation of secondary amides have already been developed.^7–10^ For example, transition-metal-catalyzed or transition-metal-free transamidations of secondary amides, where the amide nitrogen atom is pre-activated with Boc, Ts, or Ms protecting groups, have been developed (Fig. 1a).^9,10^ In marked contrast to the transamidation of pre-activated secondary amides, the transamidation of tertiary amides is very difficult to achieve and remains under development.^11–14^ Recently, a promising study on this subject has been published, where the use of an excess of a lithium amide^11^ or an excess of a manganese (Mn) reagent^12^ (Fig. 1b) results in the successful transamidation of tertiary amides. Additionally, a Lewis-acid-mediated or Lewis-acid-catalyzed transamidation of tertiary amides has been reported, albeit that the substrate scope is limited in these reactions.^13^ Although the transamidation of amides is undoubtedly a useful chemical transformation, the synthetic utility of these methods are caveated by the difficulties associated with the generation of the reactive acyl derivatives required for further synthetic transformations.^15^ Therefore, the development of an alternative approach that can generate such reactive acyl derivatives under mild conditions would be highly desirable. In this context, we herein report an efficient strategy for the generation of reactive acyl fluorides^16^ from inert tertiary amides *via* a novel radical–polar crossover approach (Fig. 1c).

## Results and discussion

Our strategy is based on the selective radical abstraction of a benzylic hydrogen atom using a Cu(ii)/Selectfluor hybrid system,^17–19^ which induces the selective cleavage of an amide N–C bond and enables the formation of the acyl fluoride. We explored the reaction using *N*-(*p*-methoxybenzyl)-*N*-phenyl-3-phenylpropanamide (**1a**), as a model compound for the desired reactivity (Table 1). After optimizing a range of reaction conditions, we found that the reaction of amide **1a** with Selectfluor (1.5 equiv.) in the presence of a catalytic amount of CuBr_2_ (2 mol%) in MeCN at 80 °C for 1 h gave rise to the corresponding acyl fluoride **2a** in 82% NMR yield and the one-pot transamidation product, *N*-benzyl-3-phenylpropionamide, was isolated in 86% yield (entry 1). As demonstrated in entry 2, the copper salt is essential to produce acyl fluoride **2a**. When the reaction was carried out under atmospheric conditions, the yield decreased (entry 3). Other copper(ii) and copper(i) salts were screened and exhibited a comparable efficiency (entries 4–6). In consideration of costs and availability, CuBr_2_ was selected for the subsequent experiments in this study. Relative to ligand-free systems, a CuBr_2_(2,2′-bipyridine) complex afforded a lower yield of **2a** (entry 7 *vs.* entry 1). This result suggests that a ligand-free system may be more advantageous for our strategy and more widely applicable. Next, we examined various electrophilic fluorine sources. Among these, Selectfluor afforded the highest chemical yield of **2a**. When *N*-fluorobenzenesulfonimide (NFSI) was used, the yield of **2a** was merely moderate (entry 8). Switching the counter anion of Selectfluor from [BF_4_]^−^ to [PF_6_]^−^ also afforded a lower yield of **2a** (entry 9). Other factors such as different metal salts, other fluorine sources, the Selectfluor loading, as well as solvent and temperature effects, were examined and the results are shown in ESI Table S1.†

**Table 1:** Transformation of tertiary amide **1a** into acyl fluoride **2a** by a Cu(ii)-catalyzed cleavage of the N–C amide bond^a^

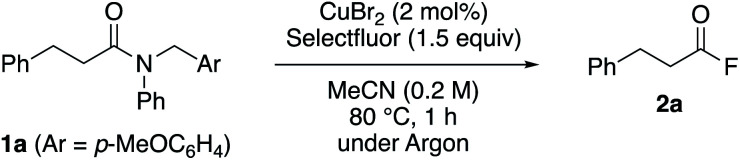			
Entry	Variation from the standard conditions	Conversion^b^ (%)	Yield of **2a**^c^ (%)
1	None	>99	82 (86)^d^
2	Without CuBr_2_	56	<1
3	Atmospheric conditions	>99	71
4	Cu(OAc)_2_	>99	78
5	CuBr	>99	82
6	CuI	>99	81
7	CuBr_2_/bpy^e^	93	73
8	NFSI^f^ (1.5 equiv.)	71	53
9	Selectfluor·PF_6_^g^ (1.5 equiv.)	>99	74

a Reaction conditions: **1a** (0.20 mmol), Selectfluor (0.30 mmol), CuBr_2_ (4 μmol, 2.0 mol% of Cu), and MeCN (1.0 mL) at 80 °C for 1 h.

b The conversion of **1a** was determined by a ^1^H NMR analysis of the crude reaction mixture using 1,1,2,2-tetrachloroethane as an internal standard.

c The yield of **2a** was determined by a ^19^F NMR analysis of the crude reaction mixture using 4,4′-difluorobenzophenone as an internal standard.

d The isolated yield of the one-pot transamidation product, *N*-benzyl-3-phenylpropionamide, is shown in parentheses (for details, see ESI).

e 2 mol% of 2,2′-bipyridine (bpy) was used.

f NFSI: *N*-fluorobenzenesulfonimide.

g Selectfluor·PF_6_: 1-chloromethyl-4-fluoro-1,4-diazoniabicyclo[2.2.2]octane bis(hexafluorophosphate).

With the optimized reaction conditions in hand, we examined the scope of the reaction with respect to other tertiary amides (Table 2). The efficiency of this transformation was determined using the yield of the product of the one-pot transamidation with benzylamine. Firstly, we examined the reactivity of *prim*-, *sec*-, and *tert*-alkanoyl amides **1a–d**, and found that our approach afforded the corresponding transamidation products in moderate to high yields. Notably, sterically hindered pivalamide **1d** could be transformed under the applied conditions. Furthermore, α,β-unsaturated carboxamide **1e** also underwent a smooth transamidation. Moreover, we discovered that a broad scope of benzamides with electron-donating (**1g**, **1h**, **1l**, **1o**) or -withdrawing (**1i–k**) motifs furnished the corresponding transamidation products in high yield. For example, *para*-bromo and *ortho*-substituted benzamides **1k**, **1l**, and **1n** are well-tolerated, albeit that these represent challenging substrates for transition-metal-catalyzed methods.^9^ Moreover, the transamidation of sterically hindered 2,4,6-trimethylbenzamide **1o** was also possible using our method.

**Table 2:** The Cu-catalyzed transamidation of tertiary amides **1**^a^

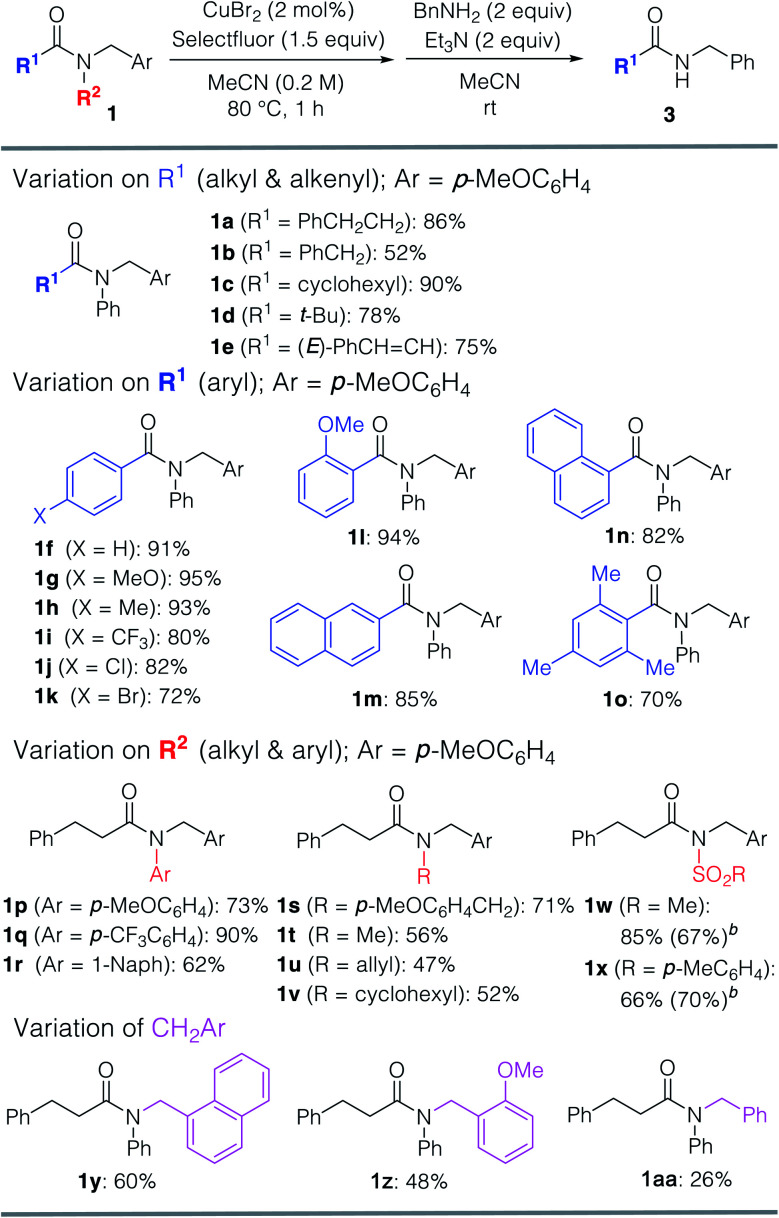

a Reaction conditions: **1a** (0.20 mmol), Selectfluor (0.30 mmol), CuBr_2_ (4 μmol, 2.0 mol% of Cu), and MeCN (1.0 mL) at 80 °C for 1 h under argon. To isolate the product as *N*-benzylamides, the one-pot transamidation was carried out for 3–24 h. For details, see ESI.

b Isolated yield of ethyl 3-phenylpropionate.

Then, we explored which amide *N*-aryl substituents **1p–r** are tolerated under the optimized conditions. Replacement of the phenyl group with an electron-rich arene motif in **1p** did not improve the yield of the transamidation. However, when electron-deficient arene **1q** was used, the reaction proceeded with a satisfactory yield. Interestingly, transamidation of much less activated *N*,*N*-dialkyl amides **1s–v** resulted in the formation of the desired products in moderate to good yields. Pre-activated sulfone amides **1w** and **1x** were not only converted into the corresponding amides, but also into esters *via* the versatile acyl fluoride intermediates. Notably, the N–C amide bond cleavage could be triggered when using the CH_2_Ar motif (Ar = *p*-MeOC_6_H_4_), and also when using other CH_2_Ar motifs. Substrates bearing 1-naphthyl (**1y**) and *o*-MeOC_6_H_4_ (**1z**) CH_2_Ar moieties afforded transamidation products in lower yields.

**Scheme 1:**
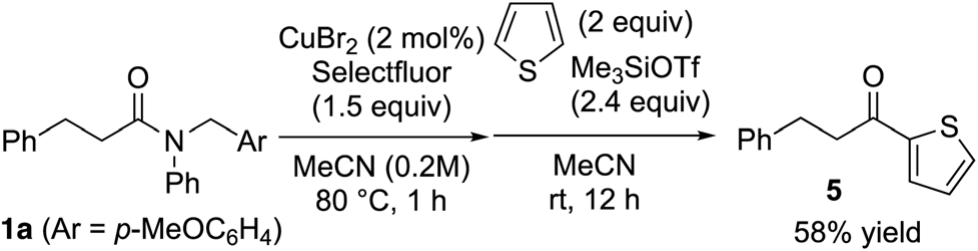
Synthesis of unsymmetrical ketone **5** from tertiary amide **1a**.

Furthermore, various amines and alcohols were screened as nucleophiles for the one-pot transformation of inert tertiary amide **1a** (Table 3). *Prim*-, *sec*-, and even bulky *tert*-alkylamines furnished the corresponding transamidation products **3a** and **3p–s** in high yield. Despite being a weak nucleophile, aniline can also be employed in our one-pot transamidation (**3t**). The synthetically versatile Weinreb-amide **3v** was also obtained in high yield. Moreover, our strategy allows the facile synthesis of a reversible inhibitor of monoamine oxidase A, moclobemide (**3w**). By taking advantage of the reactive acyl fluoride intermediate, one-pot transesterifications^20,21^ (**4a–c**) were accomplished in a highly efficient manner. In addition, thiophene can be used as a carbon nucleophile for the one-pot transformation of inert tertiary amide **1a** to the corresponding ketone **5** (Scheme 1). This result demonstrates that our strategy is highly versatile on account of the synthetic utility of the acyl fluoride intermediates.^22^

**Table 3:** Transamidation and transesterification of tertiary amide **1a**^a^

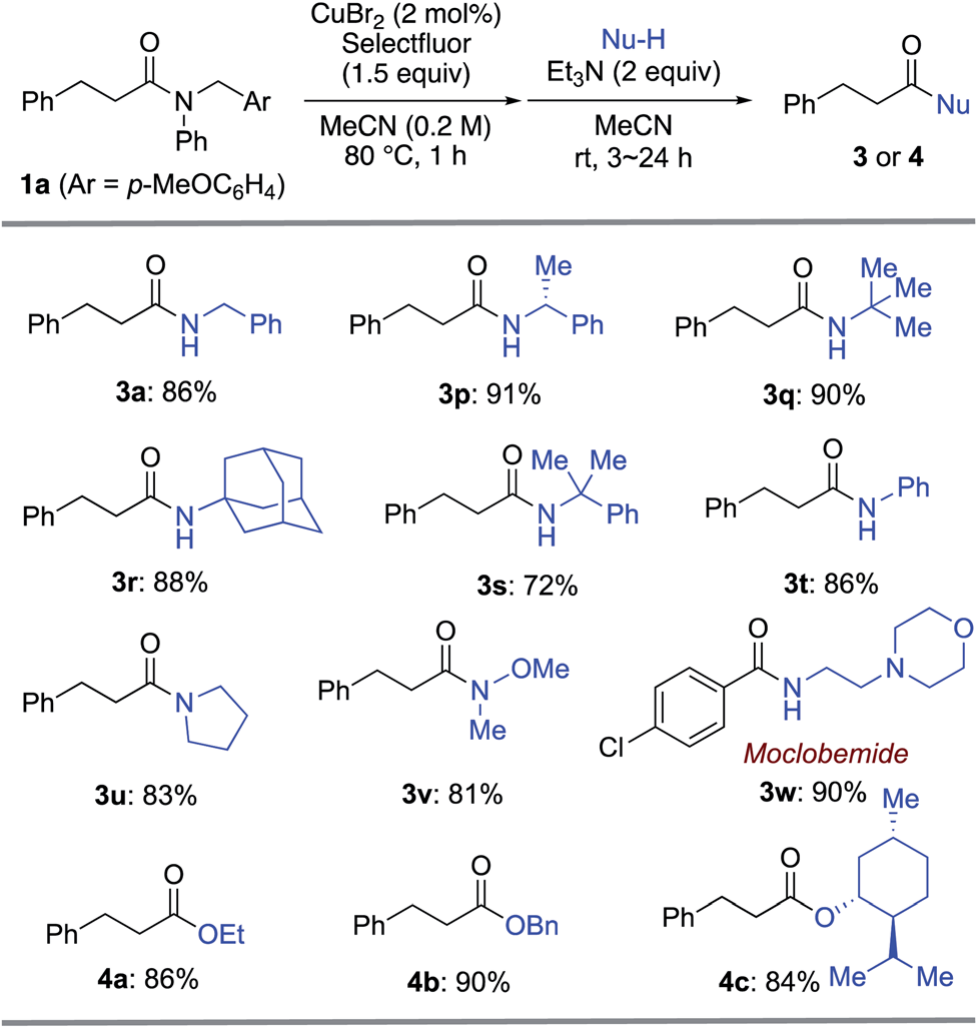

a Reaction conditions: **1a** (0.20 mmol), Selectfluor (0.30 mmol), CuBr_2_ (4 μmol, 2.0 mol% of Cu), and MeCN (1.0 mL) at 80 °C for 1 h under argon. The one-pot transamidation or transesterification was carried out at room temperature for 3–24 h. For details, see ESI.

**Scheme 2:**
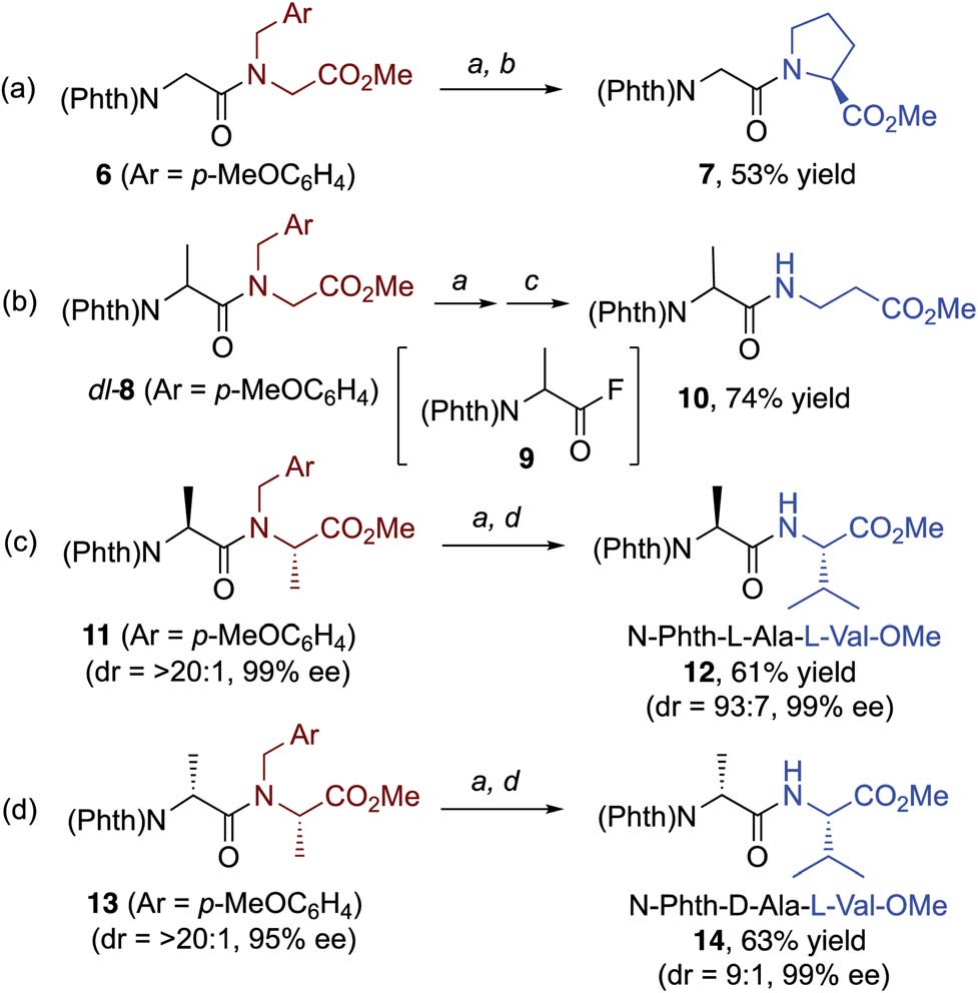
Selective cleavage of dipeptide bonds in **6**, **8**, **11**, and **13** gave new dipeptides **7**, **10**, **12**, and **14**, respectively, *via* the corresponding acyl fluorides. For details, see ESI.† (a) CuBr_2_ (2 mol%), Selectfluor (2 equiv.), MeCN (0.2 M), 80 °C, 1.5 h. (b) l-Pro-OMe·HCl (2 equiv.), *N*,*N*-diisopropylethylamine (DIPEA) (4 equiv.), THF, rt. (c) H_2_N(CH_2_)_2_CO_2_Me·HCl (2 equiv.), DIPEA (4 equiv.), THF, rt. (d) l-Val-OMe·HCl (2 equiv.), DIPEA (4 equiv.), THF, rt.

We discovered that our approach is, in principle, also applicable to the selective cleavage and reconstruction of peptides.^23,24^ Our initial findings on this subject are illustrated in Scheme 2. When *N*-phthaloyl-*N*-(*p*-methoxybenzyl)-Gly-Gly-OMe (**6**) was treated with the CuBr_2_/Selectfluor hybrid system, followed by addition of l-proline methyl ester, dipeptide **7** was obtained in moderate yield (Scheme 2a). Moreover, when dipeptide *dl*-**8** from *N*-Phth-Ala-Gly-OMe was treated with CuBr_2_/Selectfluor, the intermediary acyl fluoride **9** (81% NMR yield) was subsequently treated with an β-amino acid methyl ester to afford dipeptide **10** in good yield (Scheme 2b). Treatment of *N-p*-methoxybenzyl derivatives **11** and **13** from *N*-Phth-l-Ala-l-Ala-OMe and *N*-Phth-d-Ala-l-Ala-OMe with the CuBr_2_/Selectfluor system and subsequent addition of l-Valine methyl ester furnished diastereomerically enriched dipeptides **12** and **14**, respectively. Only minor epimerization was observed and both products were obtained in good yield (Scheme 2c and d).

**Scheme 3:**
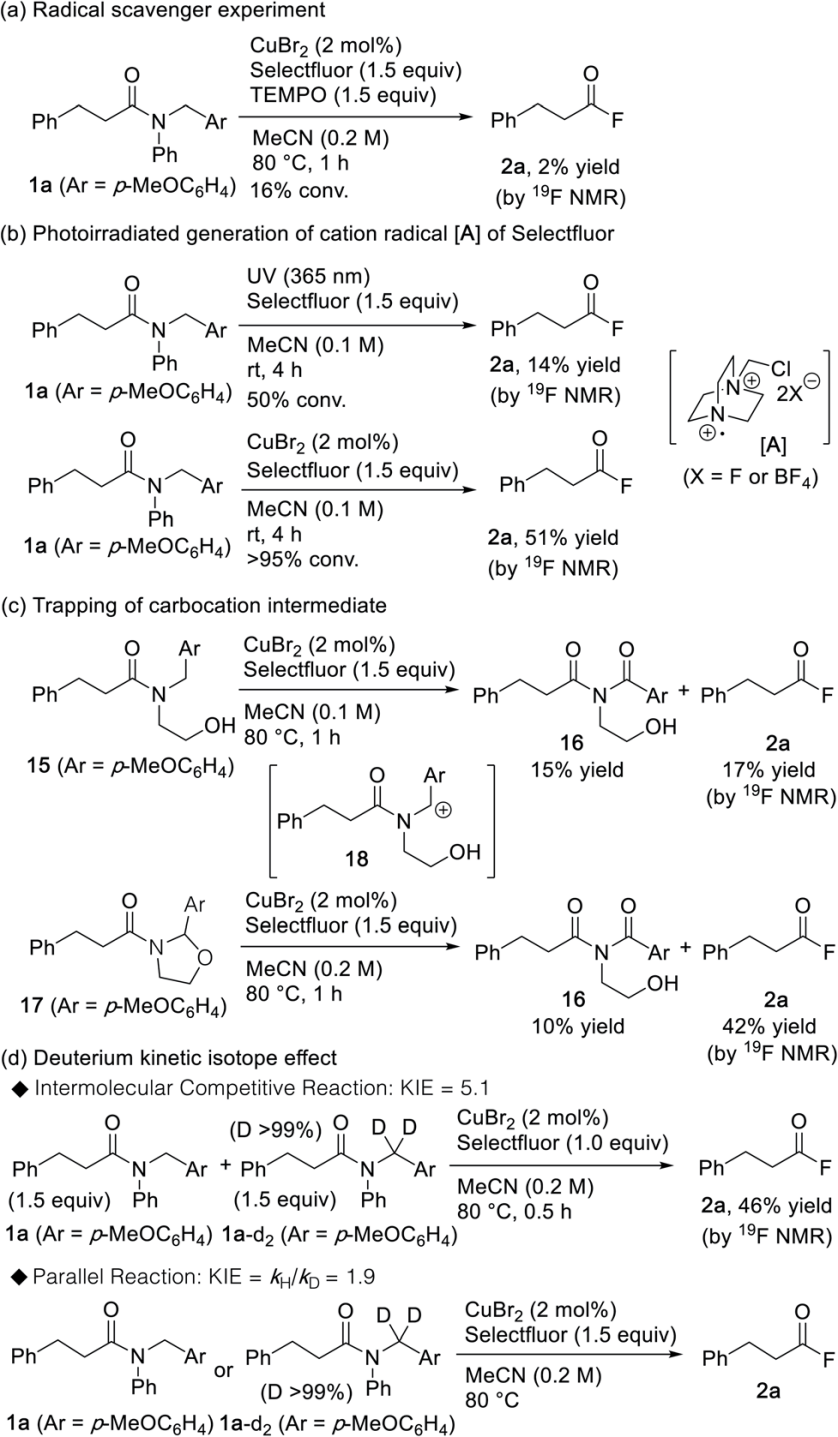
Control experiments conducted to elucidate the reaction mechanism.

Several experiments were conducted in order to gain insight into the underlying mechanistic details of this promising cleavage reaction of amide bonds. As depicted in Scheme 3a, the reaction was fully inhibited in the presence of TEMPO, thereby indicating that a radical-promoted process occurs on the catalytic pathway. It should be noted here that Selectfluor can be activated by photoirradiation, where the generated cation radical **A** is responsible for the hydrogen atom abstraction (HAA) during the C–H activation of various hydrocarbons.^25,26^ Interestingly, acyl fluoride **2a** was also obtained when the reaction was triggered *via* photoirradiation (Scheme 3b). However, when compared to the photoirradiation approach, the CuBr_2_/Selectfluor system showed higher efficiency, which suggests that the role of the copper species may not be that of an initiator, but that of a catalyst involved in the amide-cleavage process. Moreover, when *N*-(2-hydroxyethyl) amide **15** was treated with the CuBr_2_/Selectfluor system, imide^27^**16** was obtained in addition to acyl fluoride **2a** (Scheme 3c). Under similar conditions, cyclic *N*-acyl-*N*,*O*-acetal **17** afforded imide **16** (Scheme 3c). This result implies that cyclic *N*-acyl-*N*,*O*-acetal **17** may be formed *via* the intermediary carbocation **18** during the reaction between amide **15** and CuBr_2_/Selectfluor. Next, we studied the deuterium kinetic isotope effect (KIE) for the generation of the acyl fluoride from substrates **1a** and **1a**-d_2_. KIEs of 5.1 and 1.9 were observed for the intermolecular competition reaction and the parallel reaction, respectively (Scheme 3d). This result indicates that C–H bond cleavage is likely to be involved in the rate-determining step of the reaction.^28^

**Fig. 2:**
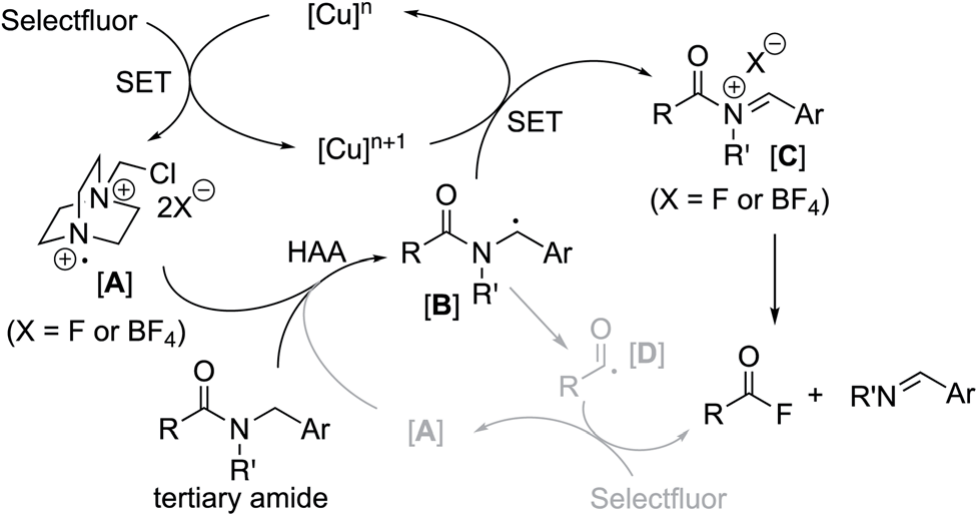
Proposed catalytic cycle for the radical–polar crossover cleavage of tertiary amides.

Based on these experimental findings, a feasible catalytic cycle is proposed for the CuBr_2_/Selectfluor-promoted amide cleavage in Fig. 2. We propose that the formation of a nitrogen-centered cation radical **A** from Selectfluor is initially triggered in the presence of a Cu(ii) salt *via* a single electron transfer (SET) mechanism.^29,30^ A subsequent HAA process could then lead to the formation of benzylic radical species **B** which is readily oxidized to give a carbocation and acyliminium species **C**. This ultimately leads to the formation of an acyl fluoride (RCOF) and imine (ArCH=NR′), most likely *via* an ionic pathway.^31^ Although the acyl fluoride formation ascribed to an acyl radical **D** with Selectfluor cannot be ruled out in our system, with the high yields of one-pot transamidation of **1c** and **1d** (see Table 2), it would be reasonable to understand that the acyl radical is unlikely mainly responsible for the acyl fluoride formation, since the competitive decarbonylation of acyl radicals,^32^ like pivaloyl radical, probably give much lower yield of transamidation.

In summary, we have realized a novel approach for the efficient cleavage of tertiary amides using a CuBr_2_/Selectfluor hybrid system. The resulting acyl fluoride intermediate can be subsequently derivatized in a one-pot fashion with various nitrogen, oxygen, and carbon nucleophiles. Mechanistic studies suggest that the present reaction proceeds *via* a radical–polar crossover process that involves benzylic carbon radicals generated by the selective radical abstraction of a benzylic hydrogen atom by the CuBr_2_/Selectfluor hybrid system. At this point, we expect that our approach may be useful for the late-stage functionalization of multi-functionalized peptides. For example, cyclic peptides such as cyclosporine^33^ could, *via* the selective benzylation and subsequent selective peptide-bond cleavage offered by our approach, be easily derivatized to furnish various types of ring-opened peptides. The exploration of these research avenues is currently in progress in our laboratory.

## Conflicts of interest

There are no conflicts to declare.

## Supplementary Material

SC-011-D0SC05137C-s001

## References

[cit1] Hughes A. B., Amino Acids, Peptides and Proteins in Organic Chemistry, Wiley-VCH, Weinheim, 2011

[cit2] Brown D. G., Boström J. (2016). J. Med. Chem..

[cit3] Marchildon K. (2011). Macromol. React. Eng..

[cit4] Greenberg A., Breneman C. M. and Liebman J. F., The Amide Linkage: Structural Significance in Chemistry, Biochemistry and Materials Science, Wiley-VCH, Weinheim, 2003

[cit5] Gonzalez-Rosende M. E., Castillo E., Lasri J., Sepúlveda-Arques J. (2004). Prog. React. Kinet. Mech..

[cit6] Pattabiraman V. R., Bode J. W. (2011). Nature.

[cit7] Li G., Szostak M. (2020). Chem. Rec..

[cit8] Kissounko D. A., Hoerter J. M., Guzei I. A., Cui Q., Gellman S. H., Stahl S. S. (2007). J. Am. Chem. Soc..

[cit9] Yu S., Shin T., Zhang M., Xia Y., Kim H., Lee S. (2018). Org. Lett..

[cit10] Chen J., Xia Y., Lee S. (2020). Org. Lett..

[cit11] Fairley M., Bole L. J., Mulks F. F., Main L., Kennedy A. R., O'Hara C. T., García-Alvarez J., Hevia E. (2020). Chem. Sci..

[cit12] Cheung C. W., Ma J.-A., Hu X. (2018). J. Am. Chem. Soc..

[cit13] Sheng H., Zeng R., Wang W., Luo S., Feng Y., Liu J., Chen W., Zhu M., Guo Q. (2017). Adv. Synth. Catal..

[cit14] Ghosh T., Jana S., Dash J. (2019). Org. Lett..

[cit15] Luo Z., Wu H., Li Y., Chen Y., Nie J., Lu S., Zhu Y., Zeng Z. (2019). Adv. Synth. Catal..

[cit16] Ogiwara Y., Sakai N. (2020). Angew. Chem., Int. Ed..

[cit17] Yazaki R., Ohshima T. (2019). Tetrahedron Lett..

[cit18] Suh S.-E., Chen S.-J., Mandal M., Guzei I. A., Cramer C. J., Stahl S. S. (2020). J. Am. Chem. Soc..

[cit19] Yang K., Song M., Ali A. I. M., Mudassir S. M., Ge H. (2020). Chem.–Asian J..

[cit20] Mashima K., Nishii Y., Nagae H. (2020). Chem. Rec..

[cit21] Nagae H., Hirai T., Kato D., Soma S., Akebi S., Mashima K. (2019). Chem. Sci..

[cit22] Raghavendra Rao K. V., Vallée Y. (2016). Tetrahedron.

[cit23] Mahesh S., Tang K.-C., Raj M. (2018). Molecules.

[cit24] Seki Y., Tanabe K., Sasaki D., Sohma Y., Oisaki K., Kanai M. (2014). Angew. Chem., Int. Ed..

[cit25] Aguilar Troyano F. J., Merkens K., Gómez-Suárez A. (2020). Asian J. Org. Chem..

[cit26] Aguilar Troyano F. J., Ballaschk F., Jaschinski M., Özkaya Y., Gómez-Suárez A. (2019). Chem.–Eur. J..

[cit27] Sperry J. (2011). Synthesis.

[cit28] Simmons E. M., Hartwig J. F. (2012). Angew. Chem., Int. Ed..

[cit29] Michaudel Q., Thevenet D., Baran P. S. (2012). J. Am. Chem. Soc..

[cit30] Sathyamoorthi S., Lai Y.-H., Bain R. M., Zare R. N. (2018). J. Org. Chem..

[cit31] Wagner R., Wiedel B., Günther W., Görls H., Anders E. (1999). Eur. J. Org. Chem..

[cit32] Chatgilialoglu C., Crich D., Komatsu M., Ryu I. (1999). Chem. Rev..

[cit33] Vinogradov A. A., Yin Y., Suga H. (2019). J. Am. Chem. Soc..

