# Supplementary material for: Efficient cleavage of tertiary amide bonds via radical–polar crossover using a copper(ii) bromide/Selectfluor hybrid system
Source: Chem Sci. 2020 Oct 14;11(45):12323–8. doi: 10.1039/d0sc05137c (PMC8163011; doi:10.1039/d0sc05137c)
Supplement: SC-011-D0SC05137C-s001 [file SC-011-D0SC05137C-s001.pdf]

Electronic Supplementary Material (ESI) for Chemical Science.
This journal is © The Royal Society of Chemistry 2020

Supporting Information

Efficient Cleavage of Tertiary Amide Bonds via Radical-Polar Crossover Using a Copper(II) Bromide/Selectfluor Hybrid System

Zhe Wang,[†] Akira Matsumoto,[†] and Keiji Maruoka*[,†,‡]

[†]*Laboratory of Organocatalytic Chemistry, Graduate School of Pharmaceutical Sciences, Kyoto University, Sakyo, Kyoto, 606-8501, Japan*

[‡]*School of Chemical Engineering and Light Industry, Guangdong University of Technology, Guangzhou, 510006, China*

Email: maruoka.keiji.4w@kyoto-u.ac.jp

Table of Contents:

1. General method S1
2. Materials S1
3. Preparation of tertiary amides substrate reported in Tables 1 & 2 S2
4. A typical procedure for transformation of tertiary amide **1a** to acyl fluoride **2a** (Table 1) S16
5. Reaction condition screening (Table S1) S17
6. A typical procedure for transamidation of tertiary amide **1** to **3** (Table 2 & 3) S18
7. Characterization data of transamidation products **3** S19
8. A typical procedure for transesterification of tertiary amide **1a** to **4** (Table 3) S27
9. Characterization data of transesterification products **4** S28
10. Synthesis of unsymmetrical ketone **5** from tertiary amide **1a** (Scheme 1) S29
11. Synthesis of dipeptides of **6**, *dl*-**8**, **11**, and **13** S30
12. Procedure for transformation of dipeptides of **6**, *dl*-**8**, **11**, and **13** to corresponding new dipeptides **7**, **10**, **12**, and **14** S36
13. Experiments for Scheme 3 S43
14. Study of deuterium kinetic isotope effect (KIE experiments) S50
15. References S57
16. NMR Charts S58

1. General method

All anaerobic and moisture-sensitive manipulations were carried out with standard Schlenk techniques under argon gas. NMR spectra were recorded on a JEOL JNM-FX400 (400 MHz for ^{1}H, 100 MHz for ^{13}C and 376 MHz for ^{19}F). Chemical shifts are reported in δ (ppm) referenced to the internal reference tetramethylsilane (TMS) in $CDCl_3$ (δ 0.00) for ^{1}H NMR and $CDCl_3$ (δ 77.00) and CD_3OD (δ 49.00) for ^{13}C NMR. The following abbreviations are used to explain the multiplicities; s: singlet, d: doublet, t: triplet, q: quartet, m: multiplet, br: broad, app: apparent. High resolution mass spectra (HRMS) were obtained with a Shimadzu ESI-IT-TOF mass spectrometer. Optical rotations were recorded on JASCO P-2000 Digital Polarimeter. Enantiomeric excesses (ee) were determined by analysis on Shimadzu HPLC with Daicel chiral columns. Photocatalytic reactions were conducted using Techno Sigma-PER-AMP-N4 power source, and LED lamp with PER-365 nm (507 mW) or PER-448 nm (680 mW). For thin layer chromatography (TLC), Merck pre-coated TLC plates (Merck 60 F254) were used, and compounds were visualized with a UV light at 254 nm. Further visualization was achieved by basic aqueous $KMnO_4$ solution stain. Flash column chromatography was performed with Silica gel 60 (Kanto Chemical Co. Inc., 40–50 μm). Further purification by preparative thin layer chromatography was performed using Merck silica gel (PTLC 60 F254, 0.5 mm) if necessary.

2. Materials

Dry solvents were purchased and used as received: tetrahydrofuran (THF), dichloromethane (CH_2Cl_2), dimethyl formamide (DMF), diethyl ether, and acetonitrile (MeCN) were purchased from Wako Pure Chemistry Co. Inc. as “Dehydrated”. 1,2-Dichloroethane (DCE) was purchased from Kanto Chemical Co., Inc. as “Dehydrated”. Commercially available reagents were purchased from FUJIFILM Wako, Sigma-Aldrich, Nacalai, and TCI, and used as received for the reactions without any purification.

3. Preparation of tertiary amides substrates reported in Tables 1 & 2

Figure S1 Intermediary secondary amines (Ar = *p*-$MeOC_6H_4$) utilized in tertiary amides synthesis

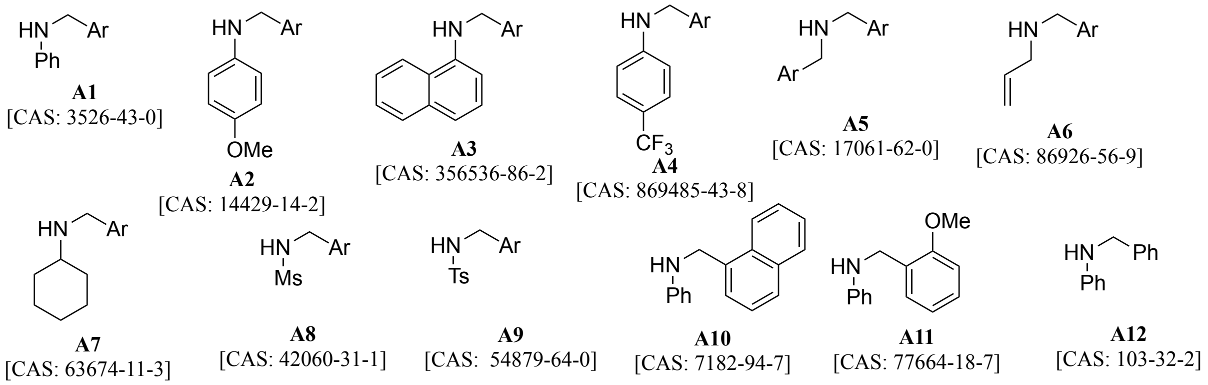

The above mentioned secondary amines are all known compounds and were prepared according to literature methods: **A1**[1], **A5**[2], **A6**[3], **A7**[4], **A8**[5], **A9**[6], **A11**[7], and **A12**[8], or prepared by the modified procedure in way of reductive amination for **A2**, **A3**, **A4,** and **A10**, which is shown as follow:

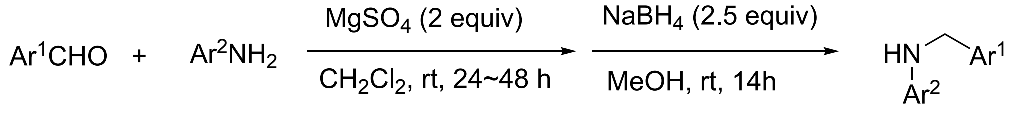

A solution of aldehyde (15 mmol), amine (15 mmol), and anhydrous $MgSO_4$ (3.61 g, 30 mmol) in CH_2Cl_2 (25 mL) was stirred for 24 h (for **A10**) or 48 h (for **A2**, **A3**, and **A4**) at room temperature (rt). The reaction mixture was filtered and the filtrate was concentrated under reduced pressure. Then the residuals were charged with MeOH (30 mL) followed by adding $NaBH_4$ (1.42 g, 37.5 mmol) portionwise at 0 °C. The reaction mixture was stirred for 14 h at rt. MeOH was evaporated by rotary evaporator, and the residuals were extracted with CH_2Cl_2 and washed by brine, dried over Na_2SO_4 and filtered. Evaporation of the solvent followed by flash

[1] M. R. Jafari, H. Yu, J. M. Wickware, Y.-S. Lin, R. Derda, *Org. Biomol. Chem.* **2018**, *16*, 7588–7594.
[2] X. Zhao, S. Liang, X. Fan, T. Yang, W. Yu, *Org. Lett.* **2019**, *21*, 1559–1563.
[3] R. F. A. Gomes, J. A. S. Coelho, C. A. M. Afonso, *ChemSusChem* **2019**, *12*, 420–425.
[4] B. Hu, Y. Li, W. Dong, K. Ren, X. Xie, J. Wan, Z. Zhang, *Chem. Commun.* **2016**, *52*, 3709–3712.
[5] F. Sanchez-Cantalejo, J. D. Priest, P. W. Davies, *Chem. Eur. J.* **2018**, *24*, 17215–17219.
[6] M. Yudasaka, D. Shimbo, T. Maruyama, N. Tada, A. Itoh, *Org. Lett.* **2019**, *21*, 1098−1102.
[7] A. Firsov, A. Sapegin, M. Krasavin, *Eur. J. Org. Chem.* **2019**, 5242–5246.
[8] Q. W. Song, B. Yu, X.-D. Li, R. Ma, Z.-F. Diao, R.-G. Li, W. Li, L.-N. He, *Green Chem.* **2014**, *16*, 1633–1638.

column chromatography on silica gel using hexane and ethyl acetate as eluents gave corresponding secondary amine.

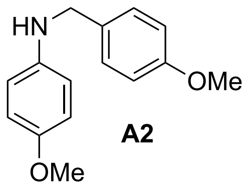

A2

[CAS: 14429-14-2]

4-Methoxy-*N*-(4-methoxybenzyl)aniline (**A2**)

A2, white solid, 65% yield (2.37 g). **^{1}H NMR ($CDCl_3$, 400 MHz)** δ 7.28 (d, *J* = 8.4 Hz, 2H), 6.87 (d, *J* = 8.7 Hz, 2H), 6.77 (d, *J* = 8.9 Hz, 2H), 6.60 (d, *J* = 8.9 Hz, 2H), 4.20 (s, 2H), 3.79 (s, 3H), 3.73 (s, 3H), 3.70 (br s, 1H); **^{13}C NMR ($CDCl_3$, 100 MHz)** δ 158.8, 152.2, 142.5, 131.7, 128.8, 114.9, 114.1, 114.0, 55.8, 55.3, 48.7. **HRMS (ESI)** calcd for $C_{15}H_{18}NO_2$ $[M+H]^+$ 244.1332, found 244.1332.

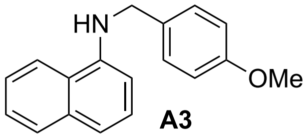

A3

[CAS: 356536-86-2]

N-(4-methoxybenzyl)naphthalen-1-amine (**A3**)

A3, white solid, 45% yield (1.78 g). **^{1}H NMR ($CDCl_3$, 400 MHz)** δ 7.79–7.75 (m, 2H), 7.45–6.88 (m, 6H), 6.89 (d, *J* = 8.2 Hz, 2H), 6.62 (d, *J* = 7.6 Hz, 1H), 4.57 (br s, 1H), 4.38 (s, 2H), 3.78 (s, 3H); **^{13}C NMR ($CDCl_3$, 100 MHz)** δ 159.0, 143.3, 134.2, 131.0, 129.0, 128.6, 126.6, 125.7, 124.7, 123.3, 119.9, 117.5, 114.1, 104.6, 55.3, 48.0. **HRMS (ESI)** calcd for $C_{18}H_{18}NO$ $[M+H]^+$ 264.1383, found 264.1381.

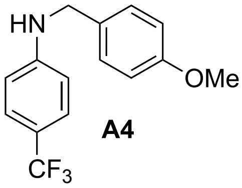

A4

[CAS: 869485-43-8]

N-(4-methoxybenzyl)-4-(trifluoromethyl)aniline (**A4**)

A4, white solid, 55% yield (2.32 g). **^{1}H NMR ($CDCl_3$, 400 MHz)** δ 7.37 (dd, *J* = 8.9 Hz, 0.6 Hz, 2H), 7.24 (d, *J* = 8.8 Hz, 2H), 6.87 (d, *J* = 8.8 Hz, 2H), 7.37 (dd, *J* = 8.9 Hz, 0.6 Hz, 2H), 4.29 (br s, 1H), 4.25 (s, 2H), 3.78 (s, 3H); **^{13}C NMR ($CDCl_3$, 100 MHz)** δ 159.0, 150.5, 130.4,

128.7, 126.6 (q, J_{C-F} = 3.8 Hz), 125.0 (q, J_{C-F} = 268.8 Hz), 118.8 (q, J_{C-F} = 32.5 Hz), 114.1, 111.9, 55.2, 47.2. **HRMS (ESI)** calcd for $C_{15}H_{15}F_3NO$ $[M+H]^+$ 282.1100, found 282.1092.

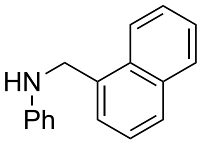

A10

[CAS: 7182-94-7]

N-(naphthalen-1-ylmethyl)aniline (**A10**)

A10, white solid, 82% yield (2.87 g). **^{1}H NMR (CDCl$_3$, 400 MHz)** δ 8.09–8.06 (m, 1H), 7.90–7.88 (m, 1H), 7.81 (d, *J* = 8.2 Hz, 1H), 7.55–7.49 (m, 3H), 7.43 (dd, *J* = 8.3 Hz, 7.1 Hz, 1H), 7.21 (dd, *J* = 8.6 Hz, 7.4 Hz, 2H), 6.75 (tt, *J* = 7.3 Hz, 1.1 Hz, 1H), 6.69 (dd, *J* = 8.7 Hz, 1.1 Hz, 2H), 4.74 (s, 2H), 3.99 (br s, 1H); **^{13}C NMR (CDCl$_3$, 100 MHz)** δ 148.2, 134.3, 133.9, 131.5, 129.3, 128.7, 128.2, 126.3, 126.0, 125.8, 125.5, 123.6, 117.6, 112.7, 46.4. **HRMS (ESI)** calcd for $C_{17}H_{16}N$ $[M+H]^+$ 234.1277, found 234.1273.

Scheme S1. General procedure for the syntheses of tertiary amides

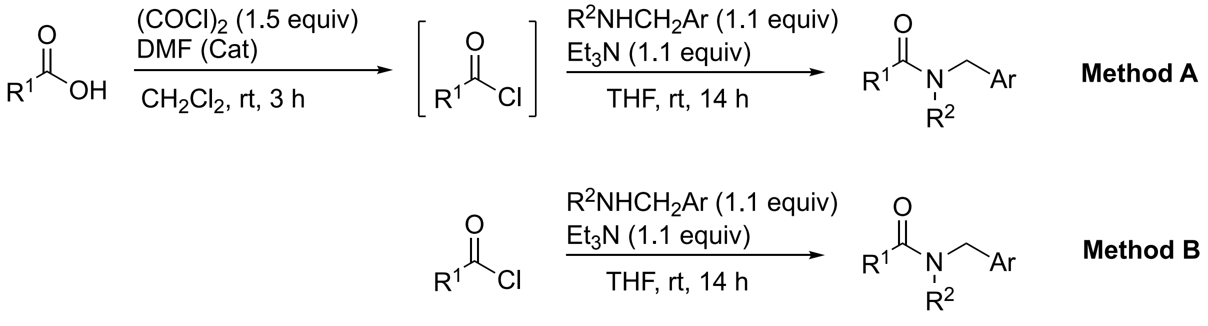

General procedure for method A in Scheme S1

To a solution of carboxylic acid (5 mmol) in CH_2Cl_2 (5 mL) was added two drops of DMF followed by oxalyl chloride (0.64 mL, 7.5 mmol) dropwise at 0 ˚C, and the mixture was stirred at rt for 3 h under argon. After removing the volatiles by rotary evaporator, the residuals were dissolved in THF (10 mL) and corresponding secondary amines, R^2NHCH_2Ar (5.5 mmol), and Et_3N (0.77 mL, 5.5 mmol) were added in sequence at 0 ˚C. The solution was stirred at rt for 14 h under argon. After adding water, the reaction mixture was extracted with ethyl acetate, and the extracts were washed by saturated Na_2CO_3 aq. solution, water, and brine, dried over Na_2SO_4

and filtered. Evaporation of the solvent followed by flash column chromatography on silica gel using hexane and ethyl acetate as eluents gave corresponding tertiary amides.

General procedure for method B in Scheme S1

To a solution of R^2NHCH_2Ar (5.5 mmol) in THF (5 mL) was added corresponding acyl chloride (5 mmol) dropwise at 0 ˚C, and followed by Et_3N (0.77 mL, 5.5 mmol). The reaction was stirred at rt for 14 h under argon. After quenching the reaction with water, the reaction mixture was extracted with ethyl acetate, and the extracts were washed by saturated Na_2CO_3 aq. solution, water, and brine, dried over Na_2SO_4 and filtered. Evaporation of the solvent followed by flash column chromatography on silica gel using hexane and ethyl acetate as eluents gave corresponding tertiary amides.

Unless otherwise stated, the following tertiary amides were prepared via **method A**

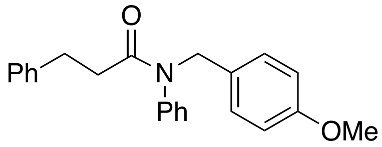

1a

N-(4-methoxybenzyl)-*N*,3-diphenylpropanamide (**1a**)

1a, white solid, 89% yield (1.54 g). **^{1}H NMR ($CDCl_3$, 400 MHz)** δ 7.27–7.12 (m, 6H), 7.06–7.03 (m, 4H), 6.78–6.75 (m, 4H), 4.79 (s, 2H), 3.76 (s, 3H), 2.93 (t, *J* = 7.7 Hz, 2H), 2. 34 (t, *J* = 7.7 Hz, 2H); **^{13}C NMR ($CDCl_3$, 100 MHz)** δ 171.8, 158.8, 142.2, 141.1, 130.1, 129.6, 129.4, 128.4, 128.3, 127.8, 125.9, 113.6, 55.1, 52.3, 36.1, 31.7, (one aromatic carbon signal overlapping). **HRMS (ESI)** calcd for $C_{23}H_{23}NO_2Na$ $[M+Na]^+$ 368.1621, found 368.1625.

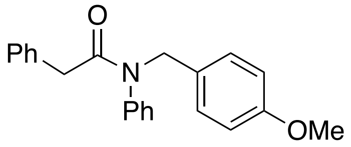

1b

N-(4-methoxybenzyl)-*N*,2-diphenylacetamide (**1b**)

1b was prepared by **method B**, colourless oil, 73% yield (1.21 g). **^{1}H NMR ($CDCl_3$, 400 MHz)** δ 7.30–7.27 (m, 3H), 7.24–7.17 (m, 3H), 7.08 (d, *J* = 8.7 Hz, 2H), 7.06–7.03 (m, 2H), 6.89–6.86 (m, 2H), 6.75 (d, *J* = 8.7 Hz, 2H), 4.81 (s, 2H), 3.75 (s, 3H), 3.43 (s, 2H); **^{13}C NMR ($CDCl_3$, 100 MHz)** δ 170.6, 158.8, 142.1, 135.4, 130.2, 129.6, 129.3, 129.0, 128.7, 128.2,

127.9, 126.5, 113.6, 55.1, 52.5, 41.2. **HRMS (ESI)** calcd for $C_{22}H_{22}NO_2$ [M+H]$^+$ 332.1645, found 332.1647.

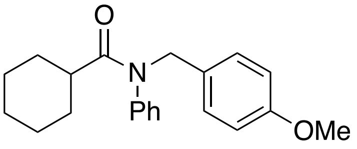

1c

<u>*N*-(4-methoxybenzyl)-*N*-phenylcyclohexanecarboxamide</u> (**1c**)

1c was prepared by **method B**, colourless oil, 90% yield (1.46 g). **^{1}H NMR ($CDCl_3$, 400 MHz)** δ 7.32–7.29 (m, 3H), 7.08 (d, *J* = 8.7 Hz, 2H), 6.95–6.92 (m, 2H), 6.77 (d, *J* = 8.7 Hz, 2H), 4.79 (s, 2H), 3.76 (s, 3H), 2.14–2.08 (m, 1H), 1.67–1.52 (m, 7H), 1.24–1.13 (m, 1H), 0.98–0.89 (m, 2H); **^{13}C NMR ($CDCl_3$, 100 MHz)** δ 176.0, 158.7, 142.4, 130.0, 129.3, 128.3, 127.7, 113.6, 55.1, 52.1, 41.6, 29.4, 25.6, 25.4, (one aromatic carbon signal overlapping). **HRMS (ESI)** calcd for $C_{21}H_{26}NO_2$ [M+H]$^+$ 324.1958, found 324.1958.

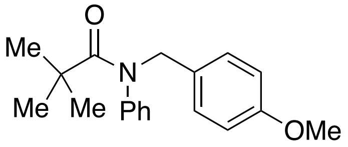

1d

<u>*N*-(4-methoxybenzyl)-*N*-phenylpivalamide</u> (**1d**)

1d, white solid, 61% yield (0.92 g). **^{1}H NMR ($CDCl_3$, 400 MHz)** δ 7.29–7.26 (m, 3H), 7.07 (d, *J* = 8.7 Hz, 2H), 6.98–6.95 (m, 2H), 6.77 (d, *J* = 8.7 Hz, 2H), 4.76 (s, 2H), 3.77 (s, 3H), 1.02 (s, 9H); **^{13}C NMR ($CDCl_3$, 100 MHz)** δ 177.5, 158.7, 143.2, 130.3, 130.06, 130.02, 128.7, 127.8, 113.5, 55.7, 55.1, 41.0, 29.5. **HRMS (ESI)** calcd for $C_{19}H_{23}NO_2Na$ [M+Na]$^+$ 320.1621, found 320.1621.

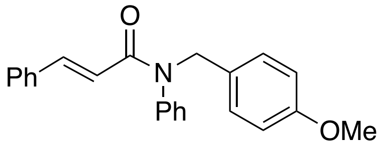

1e

<u>*N*-(4-methoxybenzyl)-*N*-phenylcinnamamide</u> (**1e**)

1e, yellow solid, 43% yield (0.74 g). **^{1}H NMR ($CDCl_3$, 400 MHz)** δ 7.73 (d, *J* = 15.5 Hz, 1H), 7.38–7.32 (m, 3H), 7.30–7.24 (m, 5H), 7.17 (d, *J* = 8.7 Hz, 2H), 7.06–7.03 (m, 2H), 6.79 (d, *J* = 8.7 Hz, 2H), 6.30 (d, *J* = 15.5 Hz, 1H), 4.96 (s, 2H), 3.76 (s, 3H); **^{13}C NMR ($CDCl_3$, 100**

MHz) δ 165.8, 158.8, 142.1, 141.9, 135.2, 130.1, 129.7, 129.5, 129.4, 128.6, 128.5, 127.8, 127.7, 118.9, 113.7, 55.1, 52.6. **HRMS (ESI)** calcd for $C_{23}H_{22}NO_2$ $[M+H]^+$ 344.1645, found 344.1652.

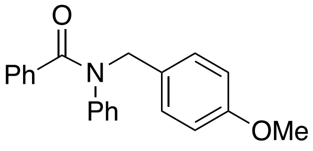

1f CAS: 102478-88-6

N-(4-methoxybenzyl)-*N*-phenylbenzamide (**1f**)

1f was prepared by **method B**, white solid, 80% yield (1.27 g). **^{1}H NMR ($CDCl_3$, 400 MHz)** δ 7.32−7.30 (m, 2H), 7.23−7.17 (m, 3H), 7.15−7.05 (m, 5H), 6.90−6.87 (m, 2H), 6.80 (d, *J* = 8.7 Hz, 2H), 5.06 (s, 2H), 3.76 (s, 3H); **^{13}C NMR ($CDCl_3$, 100 MHz)** δ 170.4, 158.8, 143.4, 136.1, 129.8, 129.7, 129.5, 128.9, 128.7, 127.9, 127.6, 126.6, 113.7, 55.1, 53.2. **HRMS (ESI)** calcd for $C_{21}H_{19}NO_2Na$ $[M+Na]^+$ 340.1308, found 340.1304.

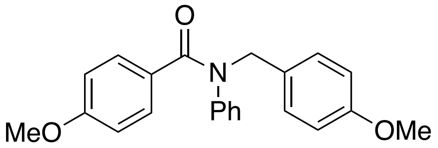

1g CAS: 1519935-84-2

4-Methoxy-*N*-(4-methoxybenzyl)-*N*-phenylbenzamide (**1g**)

1g, colourless oil, 67% yield (1.16 g). **^{1}H NMR ($CDCl_3$, 400 MHz)** δ 7.29 (d, *J* = 8.9 Hz, 2H), 7.22 (d, *J* = 8.7 Hz, 2H), 7.17−7.07 (m, 3H), 6.91−6.88 (m, 2H), 6.80 (d, *J* = 8.7 Hz, 2H), 6.64 (d, *J* = 8.9 Hz, 2H), 5.05 (s, 2H), 3.76 (s, 3H), 3.72 (s, 3H); **^{13}C NMR ($CDCl_3$, 100 MHz)** δ 169.9, 160.5, 158.8, 143.9, 130.9, 129.8, 129.8, 128.9, 128.0, 127.7, 126.3, 113.7, 112.9, 55.1, 55.1, 53.4. **HRMS (ESI)** calcd for $C_{22}H_{22}NO_3$ $[M+H]^+$ 348.1594, found 348.1597.

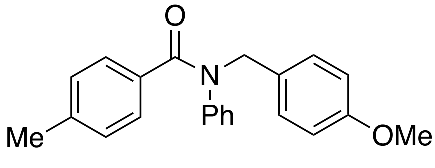

1h

N-(4-methoxybenzyl)-4-methyl-*N*-phenylbenzamide (**1h**)

1h was prepared by **method B**, white solid, 88% yield (1.46 g). **^{1}H NMR ($CDCl_3$, 400 MHz)** δ 7.23−7.20 (m, 4H), 7.15−7.06 (m, 3H), 6.94−6.88 (m, 4H), 6.80 (d, *J* = 8.7 Hz, 2H), 5.05 (s, 2H), 3.75 (s, 3H), 2.22 (s, 3H); **^{13}C NMR ($CDCl_3$, 100 MHz)** δ 170.4, 158.8, 143.7, 139.7,

133.1, 129.8, 128.9, 128.3, 127.8, 126.4, 113.7, 55.1, 53.2, 21.2, (two aromatic carbon signal overlapping). **HRMS (ESI)** calcd for $C_{22}H_{22}NO_2$ $[M+H]^+$ 332.1645, found 332.1648.

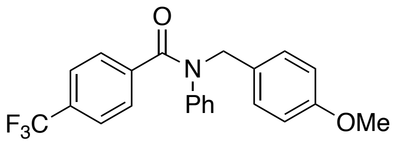

1i

N-(4-methoxybenzyl)-*N*-phenyl-4-(trifluoromethyl)benzamide (**1i**)

1i was prepared by **method B**, white solid, 89% yield (1.71 g). **^{1}H NMR ($CDCl_3$, 400 MHz)** δ 7.41 (s, 4H), 7.23–7.20 (m, 2H), 7.18–7.10 (m, 3H), 6.88–6.86 (m, 2H), 6.81 (d, *J* = 8.7 Hz, 2H), 5.06 (s, 2H), 3.77 (s, 3H); **^{13}C NMR ($CDCl_3$, 100 MHz)** δ 169.0, 159.0, 142.6, 139.6, 131.2 (q, $J_{C\text{-}F}$ = 32.2 Hz), 130.0, 129.2, 128.9, 128.0, 127.2, 124.7 (q, $J_{C\text{-}F}$ = 3.7 Hz), 123.6 (q, $J_{C\text{-}F}$ = 271.0 Hz), 113.8, 55.2, 53.3, (one aromatic carbon signal overlapping). **HRMS (ESI)** calcd for $C_{22}H_{19}F_3NO_2$ $[M+H]^+$ 386.1362, found 386.1362.

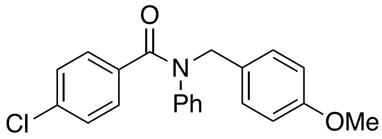

1j

4-Chloro-*N*-(4-methoxybenzyl)-*N*-phenylbenzamide (**1j**)

1j, white solid, 90% yield (1.58 g). **^{1}H NMR ($CDCl_3$, 400 MHz)** δ 7.26–7.23 (m, 2H), 7.21–7.10 (m, 7H), 6.88–6.86 (m, 2H), 6.81 (d, *J* = 8.7 Hz, 2H), 5.04 (s, 2H), 3.77 (s, 3H); **^{13}C NMR ($CDCl_3$, 100 MHz)** δ 169.2, 158.9, 143.1, 135.6, 134.5, 130.2, 129.9, 129.4, 129.1, 127.9, 127.9, 126.9, 113.8, 55.2, 53.3. **HRMS (ESI)** calcd for $C_{21}H_{19}ClNO_2$ $[M+H]^+$ 352.1099, found 352.1102.

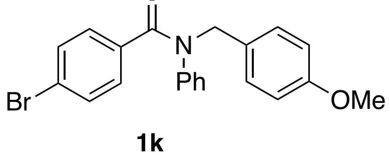

1k

4-Bromo-*N*-(4-methoxybenzyl)-*N*-phenylbenzamide (**1k**)

1k, white solid, 83% yield (1.64 g). **^{1}H NMR ($CDCl_3$, 400 MHz)** δ 7.29–7.25 (m, 2H), 7.21–7.11 (m, 7H), 6.88–6.86 (m, 2H), 6.80 (d, *J* = 8.7 Hz, 2H), 5.03 (s, 2H), 3.77 (s, 3H); **^{13}C NMR ($CDCl_3$, 100 MHz)** δ 169.3, 158.9, 143.1, 134.9, 130.9, 130.4, 129.9, 129.4, 129.1,

127.9, 126.9, 124.0, 113.8, 55.2, 53.3. **HRMS (ESI)** calcd for $C_{21}H_{19}BrNO_2$ $[M+H]^+$ 396.0594, found 396.0596.

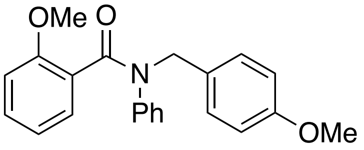

1l

2-Methoxy-*N*-(4-methoxybenzyl)-*N*-phenylbenzamide (**1l**)

1l, colourless oil, 50% yield (0.87 g). **^{1}H NMR (CDCl$_3$, 400 MHz)** δ 7.27–7.00 (m, 7H), 6.89–6.74 (m, 5H), 6.59 (d, *J* = 8.3 Hz, 1H), 5.05 (s, 2H), 3.77 (s, 3H), 3.63 (s, 3H); **^{13}C NMR (CDCl$_3$, 100 MHz)** δ 169.0, 158.7, 154.9, 142.3, 130.1, 129.7, 129.7, 128.5, 128.1, 127.8, 126.7, 126.7, 120.1, 113.6, 110.4, 55.1, 55.0, 52.1. **HRMS (ESI)** calcd for $C_{22}H_{22}NO_3$ $[M+H]^+$ 348.1594, found 348.1595.

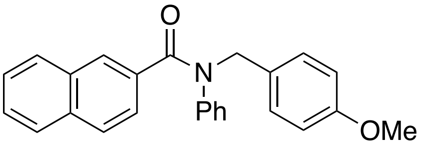

1m

N-(4-methoxybenzyl)-*N*-phenyl-2-naphthamide (**1m**)

1m, white solid, 49% yield (0.90 g). **^{1}H NMR (CDCl$_3$, 400 MHz)** δ 7.90 (s, 1H), 7.70–7.67 (m, 2H), 7.56 (d, *J* = 8.6 Hz, 1H), 7.44–7.37 (m, 2H), 7.34 (dd, *J* = 8.5 Hz, 1.7 Hz, 1H), 7.26 (d, *J* = 8.7 Hz, 2H), 7.11–7.01 (m, 3H), 6.94–6.92 (m, 2H), 6.82 (d, *J* = 8.7 Hz, 2H), 5.11 (s, 2H), 3.75 (s, 3H); **^{13}C NMR (CDCl$_3$, 100 MHz)** δ 170.3, 158.9, 143.4, 133.4, 133.4, 132.3, 129.9, 129.7, 129.4, 129.0, 128.5, 127.9, 127.5, 127.1, 127.0, 126.6, 126.2, 125.4, 113.7, 55.1, 53.4. **HRMS (ESI)** calcd for $C_{25}H_{22}NO_2$ $[M+H]^+$ 368.1645, found 368.1646.

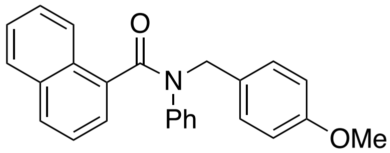

1n

N-(4-methoxybenzyl)-*N*-phenyl-1-naphthamide (**1n**)

1n, pale yellow solid, 45% yield (0.83 g). **^{1}H NMR (CDCl$_3$, 400 MHz)** δ 8.09 (d, *J* = 8.4 Hz, 1H), 7.73–7.41 (m, 4H), 7.32–7.16 (m, 4H), 6.92–6.80 (m, 7H), 5.17 (s, 2H), 3.78 (s, 3H); **^{13}C**

NMR ($CDCl_3$, 100 MHz) δ 170.2, 159.0, 142.4, 134.3, 133.2, 130.3, 130.1, 129.7, 128.9, 128.6, 128.2, 127.6, 126.8, 126.7, 126.0, 125.5, 125.2, 124.3, 113.8, 55.2, 52.4. **HRMS (ESI)** calcd for $C_{25}H_{22}NO_2$ $[M+H]^+$ 368.1645, found 368.1645.

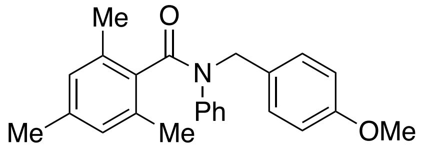

1o

<u>*N*-(4-methoxybenzyl)-2,4,6-trimethyl-*N*-phenylbenzamide</u> (**1o**)

1o, white solid, 20% yield (0.36 g). **^{1}H NMR ($CDCl_3$, 400 MHz)** mixture of rotamers (A:B = 83:17), NMR data reported are for major rotamer A; δ 7.23 (d, *J* = 8.7 Hz, 2H), 7.05–7.03 (m, 3H), 6.87–6.85 (m, 2H), 6.80 (d, *J* = 8.7 Hz, 2H), 6.58 (s, 2H), 5.08 (s, 2H), 3.77 (s, 3H), 2.20 (s, 6H), 2.10 (s, 3H); **^{13}C NMR ($CDCl_3$, 100 MHz)** major rotamer, δ 170.9, 158.9, 141.6, 137.6, 134.0, 133.3, 130.3, 129.8, 128.4, 127.8, 127.0, 126.9, 113.7, 55.1, 51.9, 20.9, 19.6. **HRMS (ESI)** calcd for $C_{24}H_{26}NO_2$ $[M+H]^+$ 360.1958, found 360.1959.

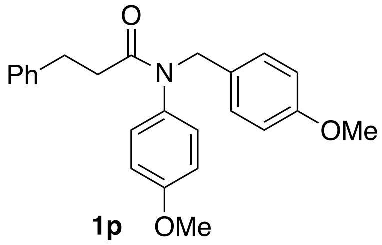

1p

<u>*N*-(4-methoxybenzyl)-*N*-(4-methoxyphenyl)-3-phenylpropanamide</u> (**1p**)

1p, colourless oil, 82% yield (1.54 g). **^{1}H NMR ($CDCl_3$, 400 MHz)** δ 7.23–7.13 (m, 3H), 7.08–7.03 (m, 4H), 6.76 (d, *J* = 8.6 Hz, 2H), 6.74 (d, *J* = 8.8 Hz, 2H), 6.64 (d, *J* = 8.8 Hz, 2H), 4.75 (s, 2H), 3.76 (s, 3H), 3.75 (s, 3H), 2.92 (t, *J* = 7.7 Hz, 2H), 2.33 (t, *J* = 7.7 Hz, 2H); **^{13}C NMR ($CDCl_3$, 100 MHz)** δ 172.2, 158.8, 158.7, 141.2, 134.8, 130.2, 129.7, 129.4, 128.5, 128.2, 125.9, 114.4, 113.5, 55.3, 55.1, 52.3, 36.0, 31.7. **HRMS (ESI)** calcd for $C_{24}H_{26}NO_3$ $[M+H]^+$ 376.1907, found 376.1908.

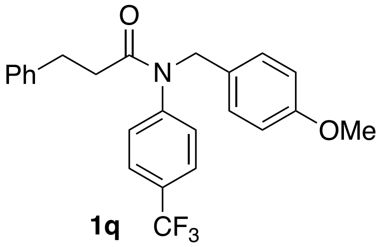

1q

<u>*N*-(4-methoxybenzyl)-3-phenyl-*N*-(4-(trifluoromethyl)phenyl)propenamide</u> (**1q**)

1q, colourless oil, 58% yield (1.20 g). **^{1}H NMR (CDCl$_3$, 400 MHz)** δ 7.51 (d, *J* = 8.0 Hz, 2H), 7.25–7.15 (m, 3H), 7.08–7.06 (m, 2H), 7.02 (d, *J* = 8.7 Hz, 2H), 6.84 (d, *J* = 8.0 Hz, 2H), 6.77 (d, *J* = 8.7 Hz, 2H), 4.80 (s, 2H), 3.77 (s, 3H), 2.95 (t, *J* = 7.5 Hz, 2H), 2.34 (t, *J* = 7.5 Hz, 2H); **^{13}C NMR (CDCl$_3$, 100 MHz)** δ 171.4, 159.0, 145.3, 140.8, 130.0, 129.0, 128.9, 128.5, 128.4, 126.6 (q, $J_{C\text{-}F}$ = 3.8 Hz), 123.6 (q, $J_{C\text{-}F}$ = 271.1 Hz), 119.6, 113.8, 55.2, 52.3, 36.2, 31.6, (one aromatic carbon signal overlapping). **HRMS (ESI)** calcd for $C_{24}H_{23}F_3NO_2$ $[M+H]^+$ 414.1675, found 414.1677.

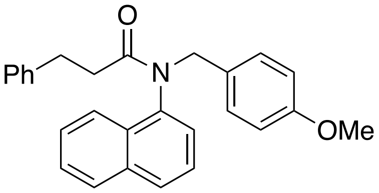

1r

<u>*N*-(4-methoxybenzyl)-*N*-(naphthalen-1-yl)-3-phenylpropanamide</u> (**1r**)

1r, pale yellow oil, 78 % yield (1.54 g). **^{1}H NMR (CDCl$_3$, 400 MHz)** δ 7.87 (dd, *J* = 7.2 Hz, 1.6 Hz, 1H), 7.78 (d, *J* = 8.3 Hz, 1H), 7.65 (d, *J* = 7.8 Hz, 1H), 7.53–7.45 (m, 2H), 7.28–7.23 (m, 1H), 7.16–7.09 (m, 3H), 7.05 (d, *J* = 8.6 Hz, 2H), 6.96–6.93 (m, 2H), 6.74 (d, *J* = 8.6 Hz, 2H), 6.63 (dd, *J* = 7.2 Hz, 1.2 Hz, 1H), 5.62 (d, *J* = 13.9 Hz, 1H), 4.09 (d, *J* = 13.9 Hz, 1H), 3.76 (s, 3H), 2.94 (ddd, *J* = 14.9 Hz, 8.8 Hz, 7.0 Hz, 1H), 2.85 (ddd, *J* = 14.9 Hz, 8.8 Hz, 6.2 Hz, 1H), 2.29 (ddd, *J* = 15.5 Hz, 8.8 Hz, 7.0 Hz, 1H), 2.16 (ddd, *J* = 15.5 Hz, 8.8 Hz, 6.2 Hz, 1H); **^{13}C NMR (CDCl$_3$, 100 MHz)** δ 172.6, 158.9, 141.0, 137.9, 134.6, 130.6, 130.2, 129.9, 128.6, 128.5, 128.4, 128.2, 127.3, 127.0, 126.5, 125.9, 125.3, 122.2, 113.5, 55.1, 51.7, 35.7, 31.6. **HRMS (ESI)** calcd for $C_{27}H_{26}NO_2$ $[M+H]^+$ 396.1958, found 396.1959.

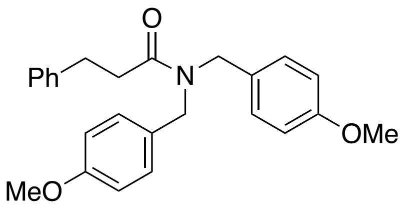

1s

<u>*N*,*N*-bis(4-methoxybenzyl)-3-phenylpropanamide</u> (**1s**)

1s, colourless oil, 78 % yield (1.52 g). **^{1}H NMR (CDCl$_3$, 400 MHz)** δ 7.27–7.17 (m, 5H), 7.11 (d, *J* = 8.7 Hz, 2H), 6.97 (d, *J* = 8.7 Hz, 2H), 6.85 (d, *J* = 8.7 Hz, 2H), 6.83 (d, *J* = 8.7 Hz, 2H), 4.50 (s, 2H), 4.28 (s, 2H), 3.79 (s, 3H), 3.78 (s, 3H), 3.04 (t, *J* = 7.7 Hz, 2H), 2.71 (t, *J* = 7.7 Hz, 2H); **^{13}C NMR (CDCl$_3$, 100 MHz)** δ 172.4, 159.0, 158.9, 141.2, 129.6, 129.5, 128.4, 128.4, 128.3, 127.6, 126.0, 114.2, 113.8, 55.2, 55.2, 49.0, 47.3, 35.0, 31.5. **HRMS (ESI)** calcd for $C_{25}H_{28}NO_3$ $[M+H]^+$ 390.2064, found 390.2068.

Scheme S2 Procedure for the synthesis of tertiary amide **1t**.

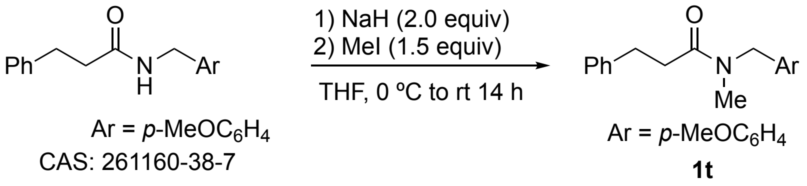

To a solution of *N*-[(4-methoxyphenyl)methyl]benzenepropanamide (0.81 g, 3mmol) in THF (5 mL) was added NaH (60% suspension in paraffin oil) (0.24 g, 6 mmol) portionwise at 0 ˚C under argon. After the mixture was stirred at 0 ˚C for 20 mins, iodomethane (0.28 mL, 4.5 mmol) was added dropwise and the solution was warmed up to rt and stirred there for 14 h under argon. The reaction was quenched with water and the mixture was extracted with ethyl acetate. The extracts were washed by brine, dried over Na_2SO_4, and filtered. Evaporation of the solvent followed by flash column chromatography on silica gel using hexane and ethyl acetate as eluents gave tertiary amide **1t** as colourless oil.

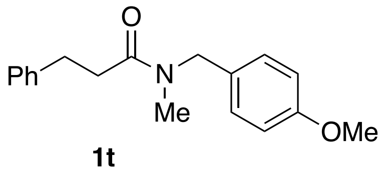

1t

<u>*N*-(4-methoxybenzyl)-*N*-methyl-3-phenylpropanamide</u> (**1t**)

1t, colourless oil, 89% yield (0.76 g). **^{1}H NMR (CDCl$_3$, 400 MHz)** observed mixture of rotamers (A:B = 60:40), NMR data reported are for mixture of rotamers; δ 7.30–7.17 (m, 5H,

A & B), 7.14 (d, J = 8.6 Hz, 1.2H, A), 6.99 (d, J = 8.6 Hz, 0.8H, B), 6.86–6.82 (m, 2H, A & B), 4.52 (s, 1.2H, A), 4.38 (s, 0.8H, A), 3.78 (s, 3H, A & B), 3.03–2.98 (m, 2H, A & B), 2.91 (s, 1.2H, B), 2.81 (s, 1.8H, A), 2.70–2.63 (m, 2H, A & B); **^{13}C NMR ($CDCl_3$, 100 MHz)** No assignments of rotamers, the observed peaks are summarized as follow, δ 172.3, 172.0, 159.0, 158.8, 141.31, 141.26, 129.4, 129.3, 128.4, 127.5, 126.0, 114.2, 113.8, 55.2, 55.1, 52.6, 50.1, 35.3, 34.9, 34.4, 33.6, 31.5, 31.3, (one aromatic carbon signal overlapping for rotamers A & B). **HRMS (ESI)** calcd for $C_{18}H_{22}NO_2$ $[M+H]^+$ 284.1645, found 284.1650.

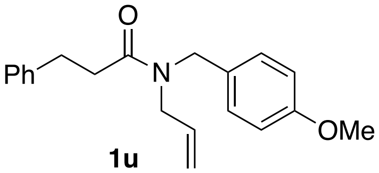

1u

N-allyl-*N*-(4-methoxybenzyl)-3-phenylpropanamide (**1u**)

1u, pale yellow oil, 72% yield (1.11 g). **^{1}H NMR ($CDCl_3$, 400 MHz)** observed mixture of rotamers (A:B = 60:40), NMR data reported are for mixture of rotamers; δ 7.29–7.13 (m, 6.2H, A & B), 6.99 (d, J = 8.7 Hz, 0.8H, B), 6.85–6.81 (m, 2H, A & B), 5.75 (ddt, J = 16.2 Hz, 12.0 Hz, 6.0 Hz, 0.4H, B), 5.65 (ddt, J = 20.0 Hz, 10.0 Hz, 4.8 Hz, 0.6H, A), 5.18–5.03 (m, 2H, A & B), 4.52 (s, 1.2H, A), 4.36 (s, 0.8H, B), 4.00–3.97 (m, 0.8H, B), 3.78 (s, 3H, A & B), 3.74–3.72 (m, 1.2H, A), 3.04–2.98 (m, 2H, A & B), 2.69-2.62 (m, 2H, A & B); **^{13}C NMR ($CDCl_3$, 100 MHz)** No assignments of rotamers, the observed peaks are summarized as follow, δ 172.4, 172.2, 159.0, 158.9, 141.3, 141.2, 133.0, 132.6, 129.6, 129.6, 128.4, 128.4, 128.4, 127.5, 126.0, 117.3, 116.6, 114.2, 113.8, 55.2, 55.2, 49.4, 48.7, 47.8, 47.7, 35.0, 34.8, 31.5, (one aliphatic carbon signal and three aromatic carbon signal overlapping for rotamers A & B) **HRMS (ESI)** calcd for $C_{20}H_{24}NO_2$ $[M+H]^+$ 310.1802, found 310.1805.

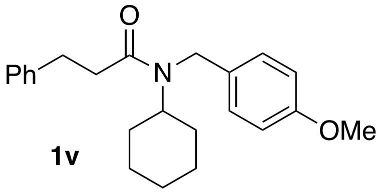

1v

N-cyclohexyl-*N*-(4-methoxybenzyl)-3-phenylpropanamide (**1v**)

1v, colourless oil, 90% yield (1.58 g). **^{1}H NMR ($CDCl_3$, 400 MHz)** observed mixture of rotamers (A:B = 60:40), NMR data reported are for mixture of rotamers; δ 7.32–7.16 (m, 5.8 H, A & B), 7.00 (d, J = 8.6 Hz, 1.2 H, A), 6.83–6.78 (m, 2H, A & B), 4.54–4.46 (m, 0.6 H, A),

4.50 (s, 0.8 H, B), 4.30 (s, 1.2 H, A), 3.78 (s, 1.8H, A), 3.78 (s, 1.2H, B), 3.62–3.54 (m, 0.4H, B), 3.05 (t, *J* = 7.9 Hz, 0.8H, B), 2.95 (t, *J* = 7.6 Hz, 1.2H, A), 2.74 (t, *J* = 7.9 Hz, 0.8H, B), 2.52 (t, *J* = 7.6 Hz, 1.2H, A), 1.75–1.53 (m, 5H, A & B), 1.42–0.95 (m, 5H, A & B); **^{13}C NMR ($CDCl_3$, 100 MHz)** No assignments of rotamers, the observed peaks are summarized as follow, δ 172.8, 172.1, 158.5, 158.2, 141.5, 141.3, 131.9, 130.5, 128.5, 128.5, 128.4, 128.3, 128.2, 126.8, 126.1, 125.9, 113.9, 113.5, 57.5, 55.2, 55.1, 53.8, 46.0, 44.1, 35.8, 35.6, 32.0, 31.8, 31.7, 30.6, 25.9, 25.7, 25.4, 25.2. **HRMS (ESI)** calcd for $C_{23}H_{30}NO_2$ $[M+H]^+$ 352.2271, found 352.2275.

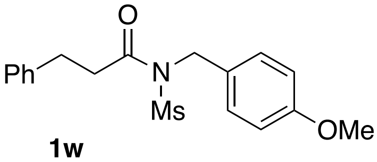

1w

N-(4-methoxybenzyl)-*N*-(methylsulfonyl)-3-phenylpropanamide (**1w**)

1w, colourless oil, 41% yield (0.71 g). **^{1}H NMR ($CDCl_3$, 400 MHz)** δ 7.28–7.24 (m, 2H), 7.22–7.14 (m, 5H), 6.84 (d, *J* = 8.7 Hz, 2H), 4.90 (s, 2H), 3.78 (s, 3H), 3.01–2.97 (m, 5H), 2.94–2.89 (m, 2H); **^{13}C NMR ($CDCl_3$, 100 MHz)** δ 173.1, 159.2, 140.2, 129.0, 128.5, 128.4, 128.1, 126.4, 114.2, 55.2, 48.2, 42.8, 38.0, 30.8. **HRMS (ESI)** calcd for $C_{18}H_{21}NO_4SNa$ $[M+Na]^+$ 370.1084, found 370.1083.

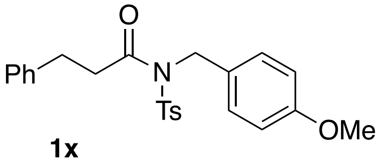

1x

N-(4-methoxybenzyl)-3-phenyl-*N*-tosylpropanamide (**1x**)

1x, pale yellow solid, 40% yield (0.85 g). **^{1}H NMR ($CDCl_3$, 400 MHz)** δ 7.55 (d, *J* = 8.5 Hz, 2H), 7.27–7.14 (m, 7H), 6.99–6.97 (m, 2H), 6.83 (d, *J* = 8.7 Hz, 2H), 4.99 (s, 2H), 3.79 (s, 3H), 2.90–2.81 (m, 4H), 2.40 (s, 3H); **^{13}C NMR ($CDCl_3$, 100 MHz)** δ 172.4, 159.1, 144.6, 140.2, 136.8, 129.6, 129.5, 128.7, 128.3, 128.2, 127.6, 126.1, 113.9, 55.2, 48.7, 37.9, 30.6, 21.5. **HRMS (ESI)** calcd for $C_{24}H_{26}NO_4S$ $[M+H]^+$ 424.1577, found 424.1579.

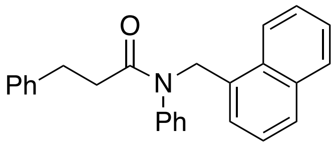

1y

<u>*N*-(naphthalen-1-ylmethyl)-*N*,3-diphenylpropanamide</u> (**1y**)

1y, pale yellow oil, 81% yield (1.48 g). **^{1}H NMR (CDCl$_3$, 400 MHz)** δ 8.14 (d, *J* = 7.4 Hz, 1H), 7.81 (dd, *J* = 7.7 Hz, 1.9Hz, 1H), 7.69 (d, *J* = 8.3 Hz, 1H), 7.52–7.44 (m, 2H), 7.21–7.05 (m, 9H), 6.93 (d, *J* = 6.9 Hz, 1H), 6.61 (d, *J* = 6.7 Hz, 2H), 5.37 (s, 2H), 2.97 (t, *J* = 7.7 Hz, 2H), 2.37 (t, *J* = 7.7 Hz, 2H); **^{13}C NMR (CDCl$_3$, 100 MHz)** δ 171.6, 141.4, 141.1, 133.6, 132.5, 131.7, 129.2, 128.5, 128.4, 128.4, 128.3, 128.2, 128.2, 127.8, 126.4, 125.9, 125.7, 124.9, 124.1, 50.3, 36.2, 31.6. **HRMS (ESI)** calcd for $C_{26}H_{24}NO$ $[M+H]^+$ 366.1852, found 366.1858.

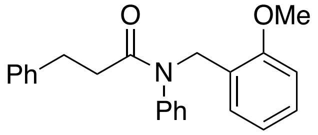

1z

<u>*N*-(2-methoxybenzyl)-*N*,3-diphenylpropanamide</u> (**1z**)

1z, colourless oil, 85% yield (1.47 g). **^{1}H NMR (CDCl$_3$, 400 MHz)** δ 7.24–7.13 (m, 8H), 7.07 (d, *J* = 7.2 Hz, 2H), 6.87–6.83 (m, 3H), 6.72 (d, *J* = 8.1 Hz, 1H), 4.93 (s, 2H), 3.53 (s, 3H), 2.95 (t, *J* = 7.7 Hz, 2H), 2.40 (t, *J* = 7.7 Hz, 2H); **^{13}C NMR (CDCl$_3$, 100 MHz)** δ 172.0, 157.2, 142.6, 141.2, 129.7, 129.1, 128.5, 128.3, 128.2, 128.2, 127.5, 125.9, 125.3, 120.3, 110.1, 54.9, 47.5, 36.1, 31.7. **HRMS (ESI)** calcd for $C_{23}H_{24}NO_2$ $[M+H]^+$ 346.1802, found 346.1807.

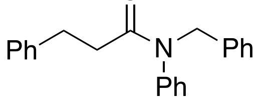

1aa CAS: 857813-92-4

<u>*N*-benzyl-*N*,3-diphenylpropanamide</u> (**1aa**)

1aa, colourless oil, 91% yield (1.44 g). **^{1}H NMR (CDCl$_3$, 400 MHz)** δ 7.25–7.11 (m, 11H), 7.07–7.05 (m, 2H), 6.79–6.77 (m, 2H), 4.86 (s, 2H), 2.94 (t, *J* = 7.7 Hz, 2H), 2.37 (t, *J* = 7.7 Hz, 2H); **^{13}C NMR (CDCl$_3$, 100 MHz)** δ 171.9, 142.2, 141.1, 137.4, 129.4, 128.7, 128.4, 128.3, 128.24, 128.22, 127.8, 127.2, 125.9, 52.9, 36.0, 31.7. **HRMS (ESI)** calcd for $C_{22}H_{22}NO$ $[M+H]^+$ 316.1696, found 316.1697.

4. A typical procedure for transformation of tertiary amide 1a to acyl fluoride 2a by Cu(II)-catalyzed N–C amide bond cleavage (Table 1, entry 1)

Scheme S3

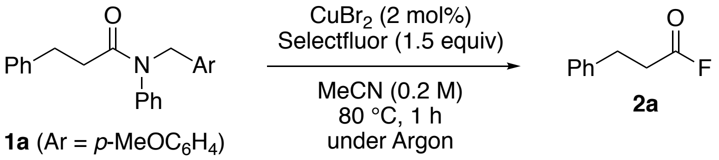

$CuBr_2$ (0.9 mg, 4 μmmol, 2 mol% of Cu), Selectfluor (106.3 mg, 0.30 mmol), and *N*-(4-methoxybenzyl)-*N*-phenyl-3-phenylpropanamide (**1a**) (69.1 mg, 0.20 mmol), were placed in an oven dried 10 mL Schlenk tube. The tube was evacuated and then re-filled with argon gas for 3 cycles. MeCN (1.0 mL) was added successively. The tube was placed in a preheated oil bath at 80 °C and stirred for 1 h equipped with argon balloon. The reaction was cooled down to rt, and the mixture was passed through a short pad of silica gel with ethyl acetate as eluent. Volatiles were removed on a rotary evaporator, and the crude mixture was subjected to NMR analysis. Conversion of **1a** and yield of **2a** in the crude mixture was determined by ^{1}H and ^{19}F NMR using 1,1,2,2-tetrachloroethane ($Cl_2CHCHCl_2$) and 4,4'-difluorobenzophenone as internal standard.

Figure S2. ^{19}F NMR chart of crude mixture (Table 1, entry 1). Illustration for determination NMR yield of **2a**

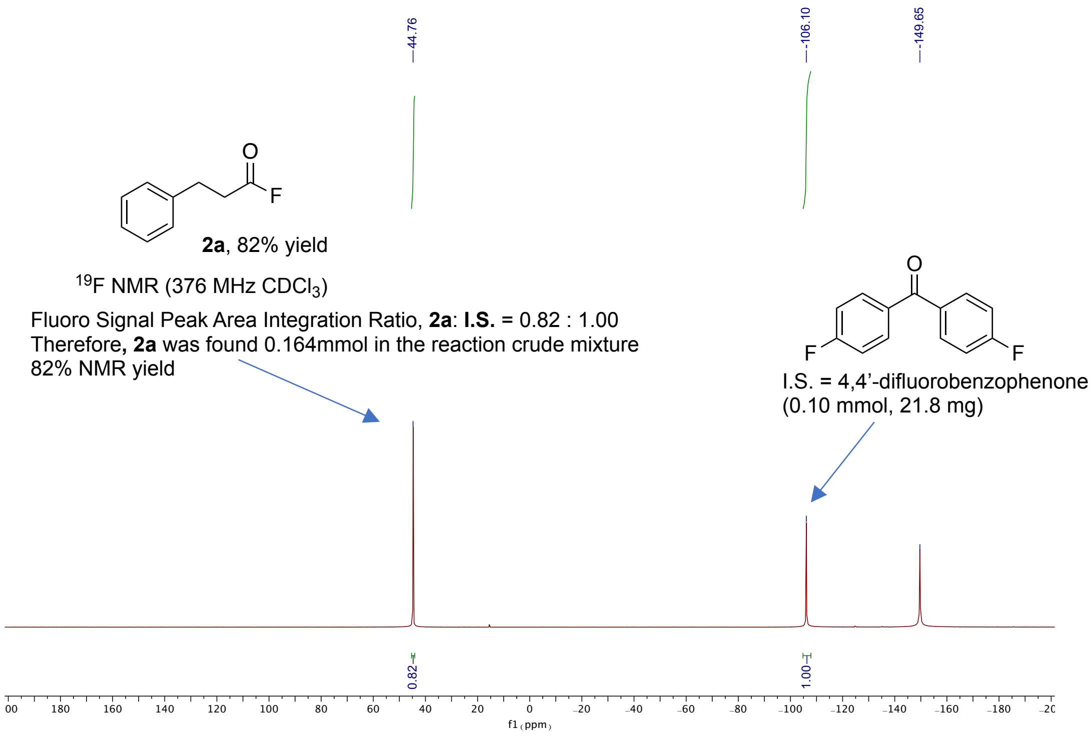

5. Reaction condition screening

Table S1. Transformation of tertiary amide **1a** into acyl fluoride **2a** by Cu(II)-catalyzed cleavage of the N–C amide bond [a]

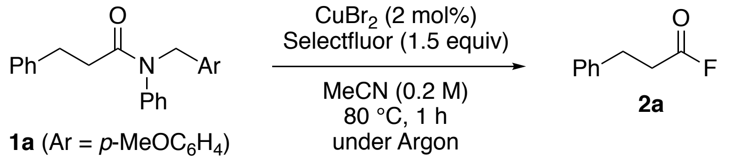

Entry	Variations from standard conditions	Conv(%)[b]	Yield(%)[c]
1	none	>99	82(86)
2	MeCN (0.1M)	>99	76
3	Without $CuBr_2$	56	<1
4	Atmospheric conditions	>99	71
5	$CuBr_2$ (10 mol%)	90	72
6	$Cu(OAc)_2$	>99	78
7	CuBr	>99	82
8	CuI	>99	81
9	$CuBr_2$/bpy[d]	93	73
10	$AgNO_3$ (10 mol%)	69	1
11	AgF (10 mol%)	65	<1
12	NFSI[e] (1.5 equiv)	71	53
13	*N*-Fluoropyridinium tetrafluoroborate (1.5 equiv)	30	15
14	Selectfluor-PF_6[f] (1.5 equiv)	>99	74
15[g]	Selectfluor (0.0 equiv)	<10	(0)
16	Selectfluor (1.0 equiv)	82	42
17	Selectfluor (2.0 equiv)	>99	81
18	$ClCH_2CH_2Cl$	<5	<1
19	at 60 °C	>99	81
20	at 90 °C	>99	81(84)

[a] Reaction conditions: **1a** (0.20 mmol), Selectfluor (0.30 mmol), $CuBr_2$ (4 μmmol, 2.0 mol% of Cu), and MeCN (1.0 mL) at 80 °C for 1 h under argon. [b] The conversion of **1a** was determined by ^{1}H NMR of the crude reaction mixture using 1,1,2,2-tetrachloroethane as an internal standard. [c] The yield of **2a** was determined by ^{19}F NMR of the crude reaction mixture using 4,4'-difluorobenzophenone as an internal standard. Isolated yields of one-port transamidation product, *N*-Benzyl-3-phenylpropionamide, are shown in parentheses. [d] Use of 2 mol% of bpy, 2,2'-bipyridine. [e] NFSI, *N*-fluorobenzenesulfonimide. [f]

Selectfluor-PF_6, 1-chloromethyl-4-fluoro-1,4-diazoniabicyclo[2.2.2]octane bis(hexafluorophosphate). [g] $CuBr_2$, 50 mol%

Attempts on the reactions of *N,N*-diethyl-3-phenylpropanamide and *N*-butyl-*N*,3-diphenylpropanamide

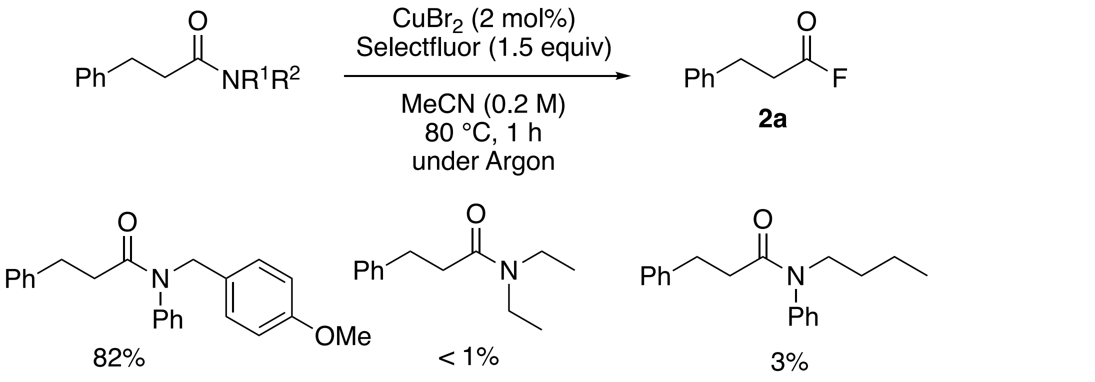

Reaction conditions: tertiary amide (0.20 mmol), Selectfluor (0.30 mmol), $CuBr_2$ (4 μmol, 2.0 mol% of Cu), and MeCN (1.0 mL) at 80 °C for 1 h. The yield of **2a** was determined by a ^{19}F NMR analysis of the crude reaction mixture using 4,4'-difluorobenzophenone as an internal standard.

6. A typical procedure for transamidation of tertiary amide 1 to 3 by Cu(II)-catalyzed N–C amide bond cleavage (Tables 2 & 3)

Scheme S4

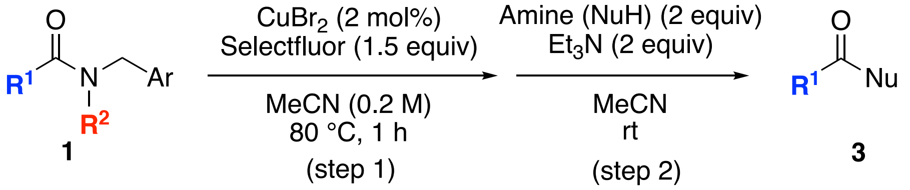

$CuBr_2$ (0.9 mg, 4 μmmol, 2 mol% of Cu), Selectfluor (106.3 mg, 0.30 mmol), and corresponding tertiary amide (**1**) (0.20 mmol), were placed in an oven dried 10 mL Schlenk tube. The tube was evacuated and then re-filled with argon gas for 3 cycles. MeCN (1.0 mL) were added successively. The tube was placed in a preheated oil bath at 80 °C and stirred for 1 h equipped with argon balloon. Subsequently, corresponding amine (0.4 mmol) and Et_3N (56 μL, 0.4 mmol) was added in sequence to the reaction mixture at 0 ˚C and the reaction was stirred at rt for 3 to 24 h with argon balloon. Volatiles were removed on a rotary evaporator, and the crude mixtures was purified by silica gel flash chromatography with hexane and ethyl acetate as eluents. Further purification by PTLC was performed using Merck silica gel (PTLC 60 F254, 0.5 mm) if necessary.

The specific reaction time for step 2 in Table 2 are shown in the following:

For 3 h: **1a**, **1b**, **1f**, **1g**, **1h**, and **1p~1z**; For 6 h: **1i~1k**, and **1m**; For 12 h: **1l** and **1n**;

For 24 h: **1o**

The specific reaction time for step 2 in Table 3 are shown in the following:

For 3 h: **3a**; For 6 h: **3p** and **3u**; For 12 h: **3q**, **3r**, **3t**, **3v**, and **3w**; For 24 h: **3s**

7. Characterization data of transamidation products 3

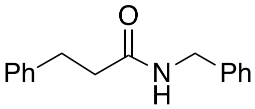

3a CAS: 10264-10-5

N-benzyl-3-phenylpropanamide (**3a**)

3a, white solid, 86% yield. **^{1}H NMR (CDCl$_3$, 400 MHz)** δ 7.30–7.23 (m, 5H), 7.22–7.17 (m, 2H), 7.14–7.11 (m, 2H), 5.77 (br s, 1H), 4.37 (d, *J* = 5.7 Hz, 2H), 2.97 (t, *J* = 7.7 Hz, 2H), 2.49 (t, *J* = 7.7 Hz, 2H); **^{13}C NMR (CDCl$_3$, 100 MHz)** δ 171.9, 140.7, 138.1, 128.6, 128.5, 128.3, 127.7, 127.4, 126.2, 43.5, 38.4, 31.7. HRMS (ESI) calcd for $C_{16}H_{18}NO$ $[M+H]^+$ 240.1383, found 240.1382.

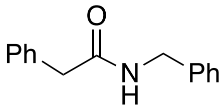

3b CAS: 7500-45-0

N-benzyl-2-phenylacetamide (**3b**)

3b, white solid, 52% yield. **^{1}H NMR (CDCl$_3$, 400 MHz)** δ 7.36–7.22 (m, 8H), 7.18–7.14 (m, 2H), 5.81 (br s, 1H), 4.39 (d, *J* = 5.8 Hz, 2H), 3.61 (s, 2H); **^{13}C NMR (CDCl$_3$, 100 MHz)** δ 170.8, 138.1, 134.7, 129.4, 129.0, 128.6, 127.4, 127.4, 127.3, 43.7, 43.5. **HRMS (ESI)** calcd for $C_{15}H_{16}NO$ $[M+H]^+$ 226.1226, found 226.1228.

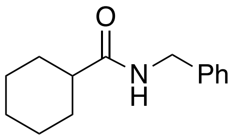

3c CAS: 35665-26-0

N-benzylcyclohexanecarboxamide (**3c**)

3c, white solid, 90% yield. **^{1}H NMR (CDCl$_3$, 400 MHz)** δ 7.36–7.22 (m, 5H), 5.86 (br s, 1H), 4.42 (d, *J* = 5.7 Hz, 2H), 2.11 (tt, *J* = 11.8 Hz, 3.5 Hz, 1H), 1.90–1.65 (m, 5H), 1.51–1.41 (m, 2H), 1.31–1.18 (m, 3H); **^{13}C NMR (CDCl$_3$, 100 MHz)** δ 175.9, 138.5, 128.6, 127.6, 127.4, 45.5, 43.3, 29.7, 25.7, (one cyclohexyl carbon signal overlapping). **HRMS (ESI)** calcd for $C_{14}H_{20}NO$ $[M+H]^+$ 218.1539, found 218.1540.

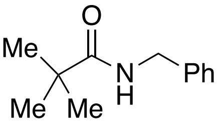

3d CAS: 26209-45-0

<u>*N*-benzylpivalamide</u> (**3d**)

3d, white solid, 78% yield. **^{1}H NMR ($CDCl_3$, 400 MHz)** δ 7.36–7.24 (m, 5H), 5.96 (br s, 1H), 4.43 (d, *J* = 5.6 Hz, 2H), 1.23 (m, 9H); **^{13}C NMR ($CDCl_3$, 100 MHz)** δ 178.2, 138.6, 128.6, 127.6, 127.4, 43.5, 38.7, 27.6. **HRMS (ESI)** calcd for $C_{12}H_{18}NO$ $[M+H]^+$ 192.1383, found 192.1384.

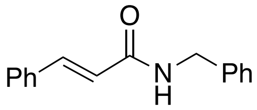

3e CAS: 5100-00-5

<u>*N*-benzylcinnamamide</u> (**3e**)

3e, pale yellow solid, 75% yield. **^{1}H NMR ($CDCl_3$, 400 MHz)** δ 7.66 (d, *J* = 15.6 Hz, 1H), 7.48–7.43 (m, 2H), 7.35–7.25 (m, 8H), 6.44 (d, *J* = 15.6 Hz, 1H), 6.23 (br s, 1H), 4.54 (d, *J* = 5.8 Hz, 2H); **^{13}C NMR ($CDCl_3$, 100 MHz)** δ 165.8, 141.3, 138.1, 134.7, 129.7, 128.76, 128.68, 127.85, 127.76, 127.5, 120.4, 43.8. **HRMS (ESI)** calcd for $C_{16}H_{16}NO$ $[M+H]^+$ 238.1226, found 238.1229.

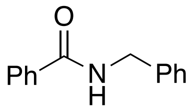

3f CAS: 1485-70-7

<u>*N*-benzylbenzamide</u> (**3f**)

3f, white solid, 91% yield. **^{1}H NMR ($CDCl_3$, 400 MHz)** δ 7.80–7.77 (m, 2H), 7.51–7.46 (m, 1H), 7.43–7.38 (m, 2H), 7.35–7.26 (m, 5H), 6.57 (br s, 1H), 4.63 (d, *J* = 5.7 Hz, 2H); **^{13}C NMR ($CDCl_3$, 100 MHz)** δ 167.3, 138.2, 134.3, 131.5, 128.7, 128.5, 127.9, 127.6, 126.9, 44.0. **HRMS (ESI)** calcd for $C_{14}H_{14}NO$ $[M+H]^+$ 212.1070, found 212.1070.

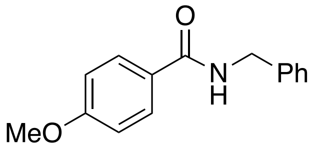

3g CAS: 7465-87-4

<u>*N*-benzyl-4-methoxybenzamide</u> (**3g**)

3g, white solid, 95% yield. **^{1}H NMR ($CDCl_3$, 400 MHz)** δ 7.76 (d, *J* = 8.8 Hz, 2H), 7.34–7.26 (m, 5H), 6.89 (d, *J* = 8.8 Hz, 2H), 6.52 (br s, 1H), 4.60 (d, *J* = 5.7 Hz, 2H), 3.83 (s, 3H); **^{13}C NMR ($CDCl_3$, 100 MHz)** δ 166.8, 162.1, 138.4, 128.7, 128.7, 127.8, 127.5, 126.6, 113.7, 55.3, 44.0. **HRMS (ESI)** calcd for $C_{15}H_{16}NO_2$ $[M+H]^+$ 242.1176, found 242.1175.

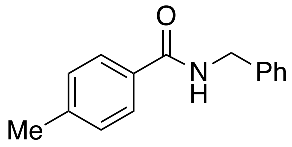

3h CAS: 5436-83-9

<u>*N*-benzyl-4-methylbenzamide</u> (**3h**)

3h, white solid, 93% yield. **^{1}H NMR ($CDCl_3$, 400 MHz)** δ 7.68 (d, *J* = 8.3 Hz, 2H), 7.34–7.25 (m, 5H), 7.20 (d, *J* = 7.6 Hz, 2H), 6.56 (br s, 1H), 4.61 (d, *J* = 5.7 Hz, 2H), 2.38 (s, 3H); **^{13}C NMR ($CDCl_3$, 100 MHz)** δ 167.3, 141.9, 138.3, 131.4, 129.2, 128.7, 127.8, 127.5, 126.9, 44.0, 21.4. **HRMS (ESI)** calcd for $C_{15}H_{16}NO$ $[M+H]^+$ 226.1226, found 226.1227.

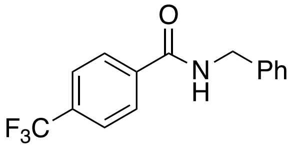

3i CAS: 365274-70-0

<u>*N*-benzyl-4-(trifluoromethyl)benzamide</u> (**3i**)

3i, white solid, 80% yield. **^{1}H NMR ($CDCl_3$, 400 MHz)** δ 7.89 (d, *J* = 8.0 Hz, 2H), δ 7.67 (d, *J* = 8.0 Hz, 2H), 7.38–7.28 (m, 5H), 6.58 (br s, 1H), 4.64 (d, *J* = 5.5 Hz, 2H); **^{13}C NMR (CD_3OD, 100 MHz)** δ 168.7, 139.9, 139.4, 134.1 (q, $J_{C\text{-}F}$ = 32.2 Hz), 129.6, 129.1, 128.6, 128.3, 126.6 (q, $J_{C\text{-}F}$ = 3.8 Hz), 125.3 (q, $J_{C\text{-}F}$ = 270.2 Hz), 44.7. **HRMS (ESI)** calcd for $C_{15}H_{13}F_3NO$ $[M+H]^+$ 280.0944, found 280.0944.

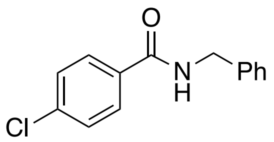

3j CAS: 7461-34-9

<u>*N*-benzyl-4-chlorobenzamide</u> (**3j**)

3j, white solid, 82% yield. **^{1}H NMR ($CDCl_3$, 400 MHz)** δ 7.71 (d, *J* = 8.6 Hz, 2H), 7.39–7.27 (m, 7H), 6.54 (br s, 1H), 4.60 (d, *J* = 5.7 Hz, 2H); **^{13}C NMR ($CDCl_3$, 100 MHz)** δ 166.3, 137.9, 137.8, 132.7, 128.8, 128.4, 127.9, 127.7, 44.2, (one aromatic carbon signal overlapping). **HRMS (ESI)** calcd for $C_{14}H_{13}ClNO$ $[M+H]^+$ 246.0680, found 246.0679.

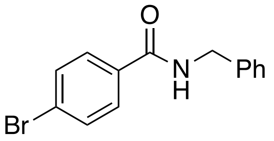

3k CAS: 80311-89-3

<u>*N*-benzyl-4-bromobenzamide</u> (**3k**)

3k, white solid, 72% yield. **^{1}H NMR ($CDCl_3$, 400 MHz)** δ 7.64 (d, *J* = 8.5 Hz, 2H), 7.54 (d, *J* = 8.5 Hz, 2H), 7.39–7.27 (m, 5H), 6.52 (br s, 1H), 4.60 (d, *J* = 5.6 Hz, 2H); **^{13}C NMR ($CDCl_3$, 100 MHz)** δ 166.4, 137.9, 133.2, 131.8, 128.8, 128.6, 127.9, 127.7, 126.2, 44.2. **HRMS (ESI)** calcd for $C_{14}H_{13}BrNO$ $[M+H]^+$ 290.0175, found 290.0177.

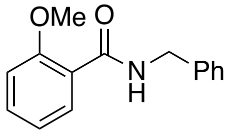

3l CAS: 183198-63-2

<u>*N*-benzyl-2-methoxybenzamide</u> (**3l**)

3l, colourless oil, 94% yield. **^{1}H NMR ($CDCl_3$, 400 MHz)** δ 8.26 (dd, *J* = 7.8 Hz, 1.8 Hz, 1H), 8.21 (br s, 1H), 7.47–7.42 (m, 1H), 7.38–7.25 (m, 5H), 7.09 (t, *J* = 7.8 Hz, 1H), 6.96 (d, *J* = 8.3 Hz, 1H), 4.69 (d, *J* = 5.7 Hz, 2H), 3.90 (s, 3H); **^{13}C NMR ($CDCl_3$, 100 MHz)** δ 165.3, 157.4, 138.8, 132.8, 132.4, 128.6, 127.4, 127.2, 121.3, 121.3, 111.2, 55.9, 43.7. **HRMS (ESI)** calcd for $C_{15}H_{16}NO_2$ $[M+H]^+$ 242.1176, found 242.1176.

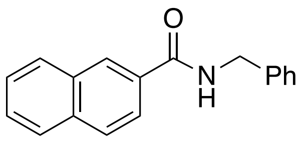

3m CAS: 149358-58-7

<u>*N*-benzyl-2-naphthamide</u> (**3m**)

3m, white solid, 85% yield. **^{1}H NMR ($CDCl_3$, 400 MHz)** δ 8.30 (s, 1H), 7.89–7.83 (m, 4H), 7.57–7.49 (m, 2H), 7.40–7.28 (m, 5H), 6.63 (br s, 1H), 4.70 (d, *J* = 5.6 Hz, 2H); **^{13}C NMR ($CDCl_3$, 100 MHz)** δ 167.4, 138.2, 134.7, 132.6, 131.6, 128.9, 128.8, 128.5, 128.0, 127.7, 127.6, 127.4, 126.7, 123.6, 44.2, (one aromatic carbon signal overlapping). **HRMS (ESI)** calcd for $C_{18}H_{16}NO$ $[M+H]^+$ 262.1226, found 262.1226.

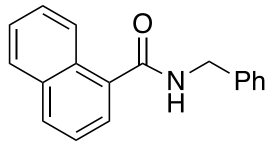

3n CAS: 27466-85-9

<u>*N*-benzyl-1-naphthamide</u> (**3n**)

3n, white solid, 82% yield. **^{1}H NMR ($CDCl_3$, 400 MHz)** δ 8.34 (d, *J* = 7.7 Hz, 1H), 7.91–7.84 (m, 2H), 7.61–7.49 (m, 3H), 7.44–7.27 (m, 6H), 6.33 (br s, 1H), 4.71 (d, *J* = 5.7 Hz, 2H); **^{13}C NMR ($CDCl_3$, 100 MHz)** δ 169.4, 138.1, 134.3, 133.7, 130.7, 130.2, 128.8, 128.3, 127.9, 127.6, 127.1, 126.4, 125.4, 124.9, 124.7, 44.1. **HRMS (ESI)** calcd for $C_{18}H_{16}NO$ $[M+H]^+$ 262.1226, found 262.1226.

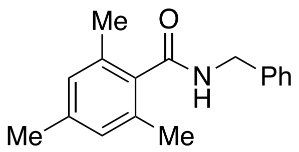

3o CAS: 104385-10-6

<u>*N*-benzyl-2,4,6-trimethylbenzamide</u> (**3o**)

3o, white solid, 70% yield. **^{1}H NMR ($CDCl_3$, 400 MHz)** δ 7.35–7.25 (m, 5H), 6.81 (s, 2H), 5.94 (br s, 1H), 4.61 (d, *J* = 5.7 Hz, 2H), 2.27 (s, 6H), 2.25 (s, 3H); **^{13}C NMR ($CDCl_3$, 100 MHz)** δ 170.3, 138.4, 138.0, 134.6, 134.1, 128.7, 128.1, 128.0, 127.6, 43.8, 21.0, 19.1. **HRMS (ESI)** calcd for $C_{17}H_{20}NO$ $[M+H]^+$ 254.1539, found 254.1540.

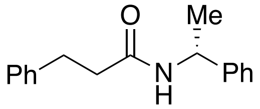

3p CAS: 154458-27-2

(*R*)-3-Phenyl-*N*-(1-phenylethyl)propenamide (**3p**)

3p, white solid, 91% yield. **^{1}H NMR ($CDCl_3$, 400 MHz)** δ 7.31−7.15 (m, 10H), 5.63 (d, *J* = 6.8 Hz, 1H), 5.13−5.05 (m, 1H), 2.95 (t, *J* = 7.6 Hz, 2H), 2.52−2.40 (m, 2H), 1.39 (d, *J* = 7.0 Hz, 3H); **^{13}C NMR ($CDCl_3$, 100 MHz)** δ 171.0, 143.0, 140.7, 128.5, 128.5, 128.4, 127.2, 126.2, 126.1, 48.5, 38.5, 31.7, 21.5. **HRMS (ESI)** calcd for $C_{17}H_{20}NO$ $[M+H]^+$ 254.1539, found 254.1540.

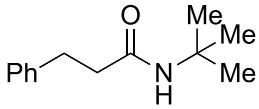

3q CAS: 58680-45-8

N-(*tert*-butyl)-3-phenylpropanamide (**3q**)

3q, white solid, 90% yield. **^{1}H NMR ($CDCl_3$, 400 MHz)** δ 7.30−7.26 (m, 2H), 7.22−7.18 (m, 3H), 5.12 (br s, 1H), 2.94 (t, *J* = 7.6 Hz, 2H), 2.38 (t, *J* = 7.6 Hz, 2H), 1.28 (s, 9H); **^{13}C NMR ($CDCl_3$, 100 MHz)** δ 171.3, 141.0, 128.4, 126.1, 51.1, 39.5, 31.8, 28.7, (one aromatic carbon signal overlapping). **HRMS (ESI)** calcd for $C_{13}H_{20}NO$ $[M+H]^+$ 206.1539, found 206.1543.

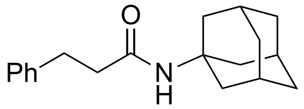

3r CAS: 109605-71-2

N-tricyclo[3.3.1.1^{3,7}]dec-1-ylbenzenepropanamide (**3r**)

3r, white solid, 88% yield. **^{1}H NMR ($CDCl_3$, 400 MHz)** δ 7.30−7.26 (m, 2H), 7.22−7.17 (m, 3H), 4.98 (br s, 1H), 2.93 (t, *J* = 7.6 Hz, 2H), 2.37 (t, *J* = 7.6 Hz, 2H), 2.04 (br s, 3H), 1.92 (br d, *J* = 2.9 Hz, 6H), 1.65 (br t, *J* = 3.0 Hz, 6H); **^{13}C NMR ($CDCl_3$, 100 MHz)** δ 171.1, 141.0, 128.4, 126.1, 51.7, 41.5, 39.5, 36.3, 31.8, 29.3, (one aromatic carbon signal overlapping). **HRMS (ESI)** calcd for $C_{19}H_{26}NO$ $[M+H]^+$ 284.2009, found 284.2009.

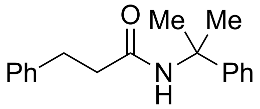

3s CAS: 107507-61-9

3-Phenyl-*N*-(2-phenylpropan-2-yl)propenamide (**3s**)

3s, white solid, 72% yield. **^{1}H NMR (CDCl$_3$, 400 MHz)** δ 7.32–7.17 (m, 10H), 5.64 (br s, 1H), 2.93 (t, *J* = 7.5 Hz, 2H), 2.45 (t, *J* = 7.5 Hz, 2H), 1.62 (s, 6H); **^{13}C NMR (CDCl$_3$, 100 MHz)** δ 170.9, 146.7, 140.9, 128.5, 128.3, 126.5, 126.2, 124.6, 55.8, 39.1, 31.6, 28.9, (one aromatic carbon signal overlapping). **HRMS (ESI)** calcd for $C_{18}H_{22}NO$ $[M+H]^+$ 268.1696, found 268.1696.

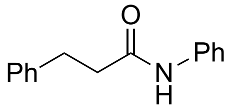

3t CAS: 3271-81-6

N,3-diphenylpropanamide (**3t**)

3t, pale yellow solid, 86% yield. **^{1}H NMR (CDCl$_3$, 400 MHz)** δ 7.42 (d, *J* = 7.9 Hz, 2H), 7.38 (br s, 1H), 7.29–7.17 (m, 7H), 7.07 (t, *J* = 7.4 Hz, 1H), 3.02 (t, *J* = 7.6 Hz, 2H), 2.62 (t, *J* = 7.6 Hz, 2H); **^{13}C NMR (CDCl$_3$, 100 MHz)** δ 170.5, 140.6, 137.7, 128.9, 128.6, 128.3, 126.3, 124.3, 120.0, 39.3, 31.5. **HRMS (ESI)** calcd for $C_{15}H_{16}NO$ $[M+H]^+$ 226.1226, found 226.1227.

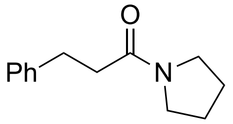

3u CAS: 151647-54-0

3-Phenyl-1-(1-pyrrolidinyl)-1-propanone (**3u**)

3u, colourless oil, 83% yield. **^{1}H NMR (CDCl$_3$, 400 MHz)** δ 7.31–7.17 (m, 5H), 3.46 (t, *J* = 6.7 Hz, 2H), 3.28 (t, *J* = 6.7 Hz, 2H), 2.99 (t, *J* = 7.9 Hz, 2H), 2.56 (t, *J* = 7.9 Hz, 2H), 1.92–1.78 (m, 4H); **^{13}C NMR (CDCl$_3$, 100 MHz)** δ 170.6, 141.5, 128.4, 128.3, 126.0, 46.4, 45.5, 36.7, 31.1, 26.0, 24.3. **HRMS (ESI)** calcd for $C_{13}H_{18}NO$ $[M+H]^+$ 204.1383, found 204.1383.

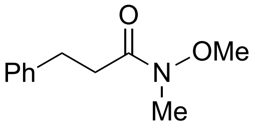

3v CAS: 170646-96-5

N-methoxy-*N*-methyl-3-phenylpropanamide (**3v**)

3v, colourless oil, 81% yield. **^{1}H NMR ($CDCl_3$, 400 MHz)** δ 7.32−7.18 (m, 5H), 3.61 (s, 3H), 3.18 (s, 3H), 2.98–2.94 (m, 2H), 2.74 (t, J = 7.8 Hz, 2H); **^{13}C NMR ($CDCl_3$, 100 MHz)** δ 173.6, 141.3, 128.4, 126.1, 61.2, 33.8, 32.1, 30.7, (one aromatic carbon signal overlapping). **HRMS (ESI)** calcd for $C_{11}H_{16}NO_2$ $[M+H]^+$ 194.1176, found 194.1179.

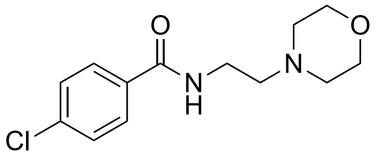

3w CAS: 71320-77-9

4-Chloro-*N*-(2-morpholinoethyl)benzamide (**3w**)

3w, white solid, 90% yield. **^{1}H NMR ($CDCl_3$, 400 MHz)** δ 7.72 (d, J = 8.5 Hz, 2H), 7.40 (d, J = 8.5 Hz, 2H), 6.84 (br s, 1H), 3.72 (t, J = 4.7 Hz, 4H), 3.56–3.51 (m, 2H), 2.60 (t, J = 6.0 Hz, 2H), 2.50 (t, J = 4.4 Hz, 4H); **^{13}C NMR ($CDCl_3$, 100 MHz)** δ 166.3, 137.5, 132.9, 128.7, 128.3, 66.9, 56.7, 53.2, 36.0. **HRMS (ESI)** calcd for $C_{13}H_{18}ClN_2O_2$ $[M+H]^+$ 269.1051, found 269.1051.

8. A typical procedure for transesterification of tertiary amide 1a to 4 by Cu(II)-catalyzed N–C amide bond cleavage (Table 3)

Scheme S5

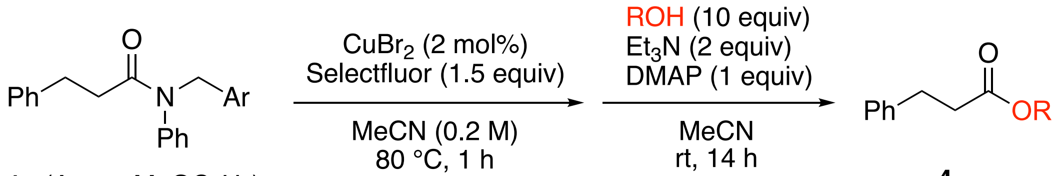

1a (Ar = *p*-MeOC$_6$H$_4$)

4

$CuBr_2$ (0.9 mg, 4 µmmol, 2 mol% of Cu), Selectfluor (107 mg, 0.30 mmol), and *N*-(4-methoxybenzyl)-*N*-phenyl-3-phenylpropanamide (**1a**) (69.1 mg, 0.20 mmol), were placed in an oven dried 10 mL Schlenk tube. The tube was evacuated and then re-filled with argon gas for 3 cycles. MeCN (1.0 mL) were added successively. The tube was placed in a preheated oil bath at 80 °C and stirred for 1 h equipped with argon balloon. Subsequently, corresponding alcohol (2.0 mmol), DMAP (24 mg, 0.20 mmol) and Et_3N (56 µL, 0.40 mmol) were added in sequence to the reaction mixture at 0 ˚C and the reaction was stirred at rt for 14 h equipped with argon balloon. Volatiles were removed on a rotary evaporator, and the crude mixtures was purified by silica gel flash chromatography with hexane and ethyl acetate as eluents.

Transesterification of tertiary amide **1w** and **1x** to **4a** (in table 2) was conducted in a same manner as described above, gave **4a** in 67% and 70% yield, repectively.

9. Characterization data of transesterification products 4

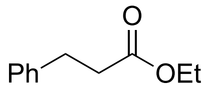

4a CAS: 2021-28-5

Ethyl 3-phenylpropanoate (**4a**)

4a, colourless oil, 86% yield. **^{1}H NMR ($CDCl_3$, 400 MHz)** δ 7.30–7.17 (m, 2H), 7.21–7.17 (m, 3H), 4.12 (q, J = 7.2 Hz, 2H), 2.95 (t, J = 7.7 Hz, 2H), 2.62 (t, J = 7.7 Hz, 2H), 1.23 (t, J = 7.2 Hz, 3H); **^{13}C NMR ($CDCl_3$, 100 MHz)** δ 172.9, 140.6, 128.4, 128.3, 126.2, 60.4, 35.9, 30.9, 14.2. **HRMS (ESI)** calcd for $C_{11}H_{14}O_2Na$ $[M+Na]^+$ 201.0886, found 201.0889.

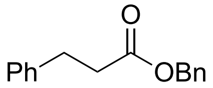

4b CAS: 22767-96-0

Benzyl 3-phenylpropanoate (**4b**)

4b, colourless oil, 90% yield. **^{1}H NMR ($CDCl_3$, 400 MHz)** δ 7.36–7.16 (m, 10H), 5.10 (s, 2H), 2.96 (t, J = 7.6 Hz, 2H), 2.67 (t, J = 7.6 Hz, 2H); **^{13}C NMR ($CDCl_3$, 100 MHz)** δ 172.7, 140.4, 135.9, 128.51, 128.47, 128.3, 128.2, 126.2, 66.2, 35.8, 30.9, (one aromatic carbon signal overlapping). **HRMS (ESI)** calcd for $C_{16}H_{16}O_2Na$ $[M+Na]^+$ 263.1043, found 263.1049.

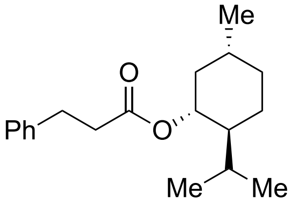

4c CAS: 101023-15-8

(1*R*,2*S*,5*R*)-2-isopropyl-5-methylcyclohexyl 3-phenylpropanoate (**4c**)

4c, colourless oil, 84% yield. **^{1}H NMR ($CDCl_3$, 400 MHz)** δ 7.29–7.25 (m, 2H), 7.21–7.17 (m, 3H), 4.67 (td, J = 10.9, 4.4 Hz, 1H), 2.97–2.93 (m, 2H), 2.61 (dd, J = 8.2 Hz, 6.6 Hz, 2H), 1.96–1.91 (m, 1H), 1.76–1.61 (m, 3H), 1.53–1.39 (m, 1H), 1.37–1.29 (m, 1H), 1.08–0.97 (m, 1H), 0.96–0.81 (m, 8H), 0.70 (d, J = 7.0 Hz, 3H); **^{13}C NMR ($CDCl_3$, 100 MHz)** δ 172.5, 140.5, 128.4, 128.3, 126.2, 74.1, 46.9, 40.8, 36.2, 34.2, 31.3, 31.0, 26.1, 23.3, 22.0, 20.7, 16.2. **HRMS (ESI)** calcd for $C_{19}H_{28}O_2Na$ $[M+Na]^+$ 311.1982, found 311.1983.

10. Synthesis of unsymmetrical ketone 5 from tertiary amide 1a (Scheme 1)

Scheme S6

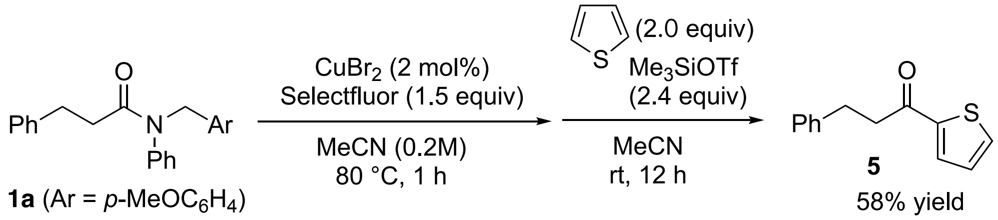

$CuBr_2$ (0.9 mg, 4 μmmol, 2 mol% of Cu), Selectfluor (106.3 mg, 0.30 mmol), and *N*-(4-methoxybenzyl)-*N*-phenyl-3-phenylpropanamide (**1a**) (69.1 mg, 0.20 mmol), were placed in an oven dried 10 mL Schlenk tube. The tube was evacuated and then re-filled with argon gas for 3 cycles. MeCN (1.0 mL) were added successively. The tube was placed in a preheated oil bath at 80 °C and stirred for 1 h equipped with argon balloon. Subsequently, thiophene (32 μL, 0.4 mmol), Me_3SiOTf (88 μL, 0.48 mmol), and Et_3N (56 μL, 0.40 mmol) were added in sequence to the reaction mixture at 0 ˚C and the reaction was stirred at rt for 12 h with argon balloon. Volatiles were removed on a rotary evaporator, and the crude mixtures was purified by silica gel flash chromatography with hexane and ethyl acetate as eluents.

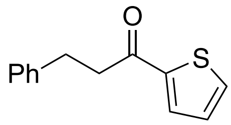

5 CAS: 40027-94-9

3-Phenyl-1-(thiophen-2-yl)propan-1-one (**5**)

5, colourless oil, 58% yield. **^{1}H NMR ($CDCl_3$, 400 MHz)** δ 7.68 (dd, *J* = 3.8 Hz, 1.1 Hz, 1H), 7.62 (dd, *J* = 4.9 Hz, 1.1 Hz, 1H), 7.32–7.18 (m, 5H), 7.11 (dd, *J* = 4.9 Hz, 3.8 Hz, 1H), 3.25–3.21 (m, 2H), 3.09–3.05 (m, 2H); **^{13}C NMR ($CDCl_3$, 100 MHz)** δ 192.1, 144.1, 141.0, 133.6, 131.8, 128.5, 128.4, 128.1, 126.2, 41.1, 30.3. **HRMS (ESI)** calcd for $C_{13}H_{13}OS$ $[M+H]^+$ 217.0682, found 217.0684.

11. Procedure for synthesis of dipeptides 6, *dl*-8, 11, and 13

Scheme S7

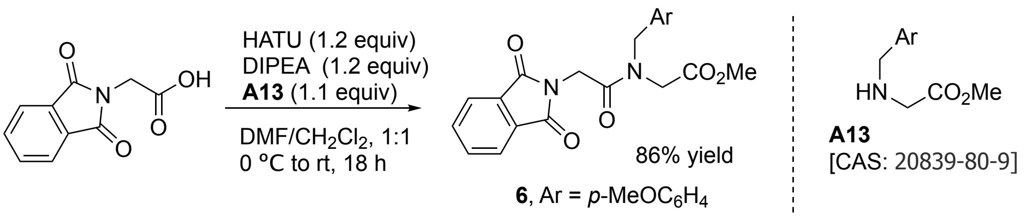

To a solution of *N*-phthaloyl-glycine (616 mg, 3 mmol) and HATU [CAS: 148893-10-1] (1.37 g, 3.6 mmol) in DMF (3 mL) was added **A13**[9] (691 mg, 3.3 mmol) (pre-dissolved in 3 mL of CH_2Cl_2) followed by *N*,*N*-diisopropylethylamine (DIPEA) (0.63 mL, 3.6 mmol) dropwise at 0 ˚C, and the mixture was stirred at rt for 18 h under argon. After adding water, the reaction mixture was extracted with ethyl acetate, and the extracts were washed by 1M aq. HCl, saturated $NaHCO_3$ aq. solution, and brine, dried over Na_2SO_4 and filtered. Evaporation of the solvent followed by flash column chromatography on silica gel using hexane and ethyl acetate as eluents gave dipeptide **6** in 86% yield as white solid.

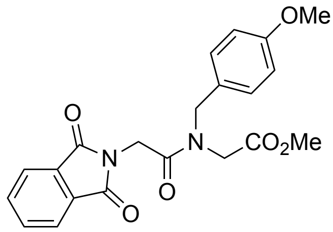

6

Methyl *N*-(2-(1,3-dioxoisoindolin-2-yl)acetyl)-*N*-(4-methoxybenzyl)glycinate (**6**)

6, white solid, 86% yield (1.02 g). **^{1}H NMR ($CDCl_3$, 400 MHz)** observed mixture of rotamers (A:B = 70:30), NMR data reported are for mixture of rotamers; δ 7.89–7.85 (m, 2H, A & B), 7.74–7.71 (m, 2H, A & B), 7.25 (d, *J* = 8.6 Hz, 1.4H, A), 7.17 (d, *J* = 8.6 Hz, 0.6H, B), 6.94 (d, *J* = 8.6 Hz, 1.4H, A), 6.85 (d, *J* = 8.6 Hz, 0.6H, B), 4.66 (s, 1.4H, A), 4.65 (s, 1.4H, A), 4.59 (s, 0.6H, B), 4.49 (s, 0.6H, B), 4.04 (s, 1.4H, A), 4.02 (s, 0.6H, B), 3.82 (s, 2.1H, A), 3.79 (s, 0.9H, B), 3.76 (s, 0.9H, B), 3.69 (s, 2.1H, A); **^{13}C NMR ($CDCl_3$, 100 MHz)** NMR data reported are for mixture of rotamers. No assignments of rotamers, the observed peaks are summarized as follow, δ 169.2, 168.9, 167.8, 166.6, 166.3, 159.5, 159.3, 134.0, 132.2, 130.1, 128.2, 127.7, 126.5, 123.5, 123.4, 114.5, 114.1, 55.3, 55.2, 52.6, 52.1, 50.9, 49.9, 47.6, 46.9, 39.1, 39.0, (one

[9] **A13** was prepared according to the literature: T. Conroy, M. Manohar, Y. Gong, S. M. Wilkinson, M. Webster, B. P. Lieberman, S. D. Banister, T. A. Reekie, R. H. Mach, L. M. Rendina, M. Kassiou, *Org. Biomol. Chem.* **2016**, *14*, 9388–9405.

acyl carbon signal overlapping for rotamer B, one aromatic carbon signal overlapping for both rotamers A & B). **HRMS (ESI)** calcd for $C_{21}H_{21}N_2O_6$ $[M+H]^+$ 397.1394, found 397.1395.

Scheme S8

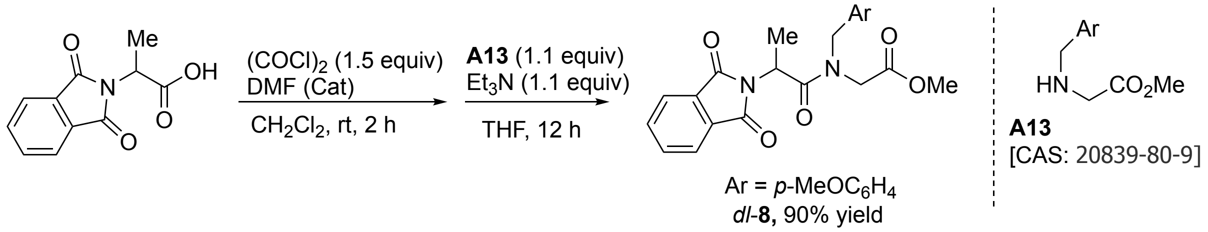

To a solution of DL-*N*-phthaloyl-alanine (657 mg, 3 mmol) in CH_2Cl_2 (5 mL) was added two drops of DMF followed by oxalyl chloride (0.39 mL, 4.5 mmol) dropwise at 0 ˚C, and the mixture was stirred at rt for 2 h under argon. After removing the volatiles by rotary evaporator, the residuals were dissolved in THF (3 mL) and **A13** (691 mg, 3.3 mmol) (pre-dissolved in 3 mL of THF), and Et_3N (0.46 mL, 3.3 mmol) was added in sequence at 0 ˚C. The solution was stirred at rt for 12 h under argon. After adding water, the reaction mixture was extracted with ethyl acetate, and the extracts were washed by saturated $NaHCO_3$ aq. solution, water, and brine, dried over Na_2SO_4 and filtered. Evaporation of the solvent followed by flash column chromatography on silica gel using hexane and ethyl acetate as eluents gave dipeptide *dl*-**8** as white solid in 90% yield.

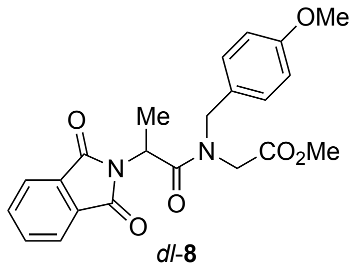

dl-**8**

Methyl *N*-(2-(1,3-dioxoisoindolin-2-yl)propanoyl)-*N*-(4-methoxybenzyl)glycinate (*dl*-**8**)

dl-**8**, white solid, 90% yield (1.11 g). **^{1}H NMR ($CDCl_3$, 400 MHz)** observed mixture of rotamers (A:B = 77:23), NMR data reported are for mixture of rotamers; δ 7.87–7.85 (m, 0.46H, B), 7.75–7.73 (m, 0.46H, B), 7.65–7.59 (m, 3.1H, A), 7.16 (d, J = 8.4 Hz, 0.46H, B), 6.89 (d, J = 8.6 Hz, 1.5H, A), 6.82 (d, J = 8.4 Hz, 0.46H, B), 6.55 (d, J = 8.6 Hz, 1.5H, A), 5.34–5.28 (m, 0.77H, A), 5.11–5.02 (m, 0.23H, B), 4.96 (d, J = 13.9 Hz, 0.23H, B), 4.68 (d, J = 17.3 Hz, 0.77H, A), 4.53 (d, J = 17.1 Hz, 0.77H, A), 4.40 (d, J = 17.3 Hz, 0.77H, A), 4.26 (d, J = 12.7 Hz, 0.23H, A), 3.93 (d, J = 18.4 Hz, 0.23H, B), 3.83 (d, J = 18.6 Hz, 0.23H, B), 3.78 (s, 0.69H, B), 3.72 (s, 2.3H, A), 3.64 (d, J = 17.1 Hz, 0.77H, A), 3.60 (s, 2.3H, A), 3.28 (s, 0.69H, B), 1.69 (d, J = 7.1 Hz, 3H, A & B); **^{13}C NMR ($CDCl_3$, 100 MHz)** NMR data reported are for

major rotamer A. δ 170.2, 169.4, 167.2, 158.6, 133.6, 131.6, 126.8, 123.1, 114.0, 55.0, 52.1, 50.9, 48.1, 46.3, 15.6, (one aromatic carbon signal overlapping). **HRMS (ESI)** calcd for $C_{22}H_{23}N_2O_6$ $[M+H]^+$ 411.1551, found 411.1551.

Scheme S9

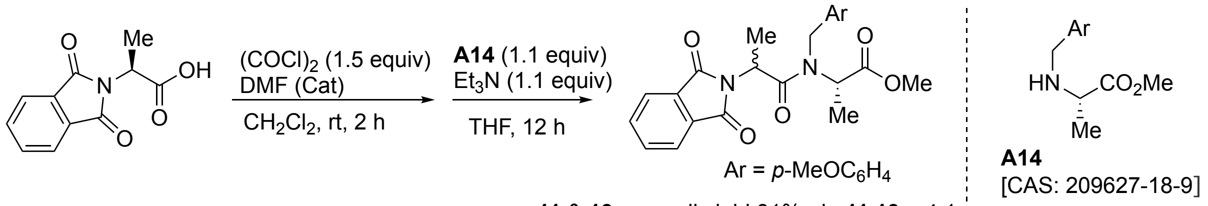

11 & **13**, over all yield 81%, dr, **11**:**13** = 4:1

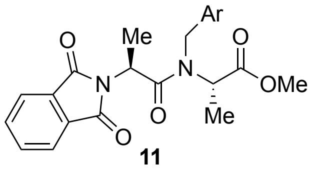

11
Ar = *p*-MeOC$_6$H$_4$

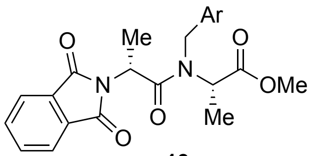

13
Ar = *p*-MeOC$_6$H$_4$

To a solution of *N*-phthaloyl-L-alanine[10] (1.31 g, 6 mmol) in CH_2Cl_2 (10 mL) was added two drops of DMF followed by oxalyl chloride (0.77 mL, 9 mmol) dropwise at 0 ˚C, and the mixture was stirred at rt for 2 h under argon. After removing the volatiles by rotary evaporator, the residuals were dissolved in THF (6 mL) and **A14**[11] (1.47 g, 6.6 mmol) (pre-dissolved in 6 mL of THF), and Et_3N (0.92 mL, 6.6 mmol) was added in sequence at 0 ˚C, and the solution was stirred at rt for 12 h under argon. After adding water, the reaction mixture was extracted with ethyl acetate, and the extracts were washed by saturated $NaHCO_3$ aq. solution, water, and brine, dried over Na_2SO_4 and filtered. Evaporation of the solvent followed by flash column chromatography on silica gel using hexane and ethyl acetate as eluents gave dipeptides **11** and **13** as a mixture in a ratio of ca. 4:1 in total 81% yield (2.06 g).

The mixture of **11** and **13** were further partially separated by flash column chromatography on silica gel using CH_2Cl_2 and ethyl acetate as eluents (CH_2Cl_2: ethyl acetate = 50:1 to 10:1). More **13** enriched eluents was flushed out and collected firstly, followed by the mixture, and then more **11** enriched portion was collected. The above mentioned column chromatography purified **11** and **13** were further recrystallized by hexane and CH_2Cl_2 in −10 °C fridge for two times. The characterization data for **11** and **13** are reported as follow:

[10] G. Liao, X.-S. Yin, K. Chen, Q. Zhang, S.-Q. Zhang, B.-F. Shi, *Nat Commun.* **2016**, *7*, 12901.
[11] **A14** was prepared following the literature method: J. L. Baeza, G. Gerona-Navarro, J. Pérez de Vega, M. T. García-López, R. González-Muníz, M. Martín-Martínez, *J. Org. Chem.* **2008**, *73*, 1704–1715.

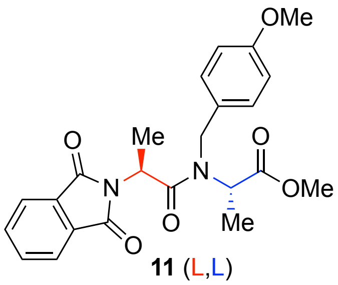

11 (L,L)

Methyl *N*-((*S*)-2-(1,3-dioxoisoindolin-2-yl)propanoyl)-*N*-(4-methoxybenzyl)-*L*-alaninate (**11**)
11, white solid, 99% ee, dr > 20:1; The ee was measured by HPLC (Daicel Chiralpak IC column), column oven: 40 ˚C, hexane/2-propanol/$CHCl_3$ = 80/10/10, flow 1.0 mL/min, 280 nm, t_{minor} = 42.5 min, t_{major} = 57.8 min. $[\alpha]^{26}_{D}$ –117 (*c* 1.10, $CHCl_3$). **^{1}H NMR ($CDCl_3$, 400 MHz)** observed mixture of rotamers (A:B = 85:15), NMR data reported are for mixture of rotamers: δ 7.88–7.84 (m, 0.3H, B), 7.76–7.71 (m, 0.3H, B), 7.62–7.57 (m, 3.4H, A), 7.16 (d, *J* = 8.1 Hz, 0.3H, B), 7.05 (d, *J* = 8.6 Hz, 1.7H, A), 6.78 (d, *J* = 8.1 Hz, 0.3H, B), 6.49 (d, *J* = 8.6 Hz, 1.7H, A), 5.19 (q, *J* = 7.0 Hz, 1H, A & B), 4.66–4.36 (m, 3H, A & B), 3.76 (s, 3H, A & B), 3.56 (s, 2.55H, A), 2.88 (s, 0.45H, B), 1.74–1.71 (m, 0.45H, B), 1.65 (d, *J* = 7.0 Hz, 2.55H, A), 1.40 (d, *J* = 7.2 Hz, 3H, A & B); **^{13}C NMR ($CDCl_3$, 100 MHz)** NMR data reported are for major rotamer A: δ 171.8, 170.2, 167.0, 158.3, 133.4, 131.6, 128.2, 126.7, 122.9, 113.7, 55.7, 54.9, 52.2, 49.1, 46.6, 15.7, 14.1. **HRMS (ESI)** calcd for $C_{23}H_{25}N_2O_6$ $[M+H]^+$ 425.1707, found 425.1707.

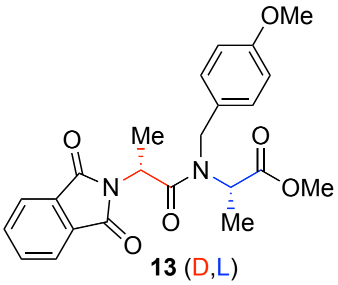

13 (D,L)

Methyl *N*-((*R*)-2-(1,3-dioxoisoindolin-2-yl)propanoyl)-*N*-(4-methoxybenzyl)-*L*-alaninate (**13**)
13, white solid, 95% ee, dr > 20:1; The ee was measured by HPLC (Daicel Chiralpak IC column), column oven: 40 ˚C, hexane/2-propanol/$CHCl_3$ = 80/10/10, flow 1.0 mL/min, 280 nm, t_{major} = 55.4 min, t_{minor} = 64.3 min. $[\alpha]^{27}_{D}$ –1.31 (*c* 1.10, $CHCl_3$). **^{1}H NMR ($CDCl_3$, 400 MHz)** observed mixture of rotamers (A:B = 85:15), NMR data reported are for major rotamer A: δ 7.73–7.63 (m, 4H), 7.08 (d, *J* = 8.2 Hz, 2H), 6.66 (d, *J* = 8.2 Hz, 2H), 5.20 (q, *J* = 7.1 Hz, 1H), 4.61 (d, *J* = 16.9 Hz, 1H), 4.48 (d, *J* = 16.9 Hz, 1H), 3.94 (q, *J* = 7.0 Hz, 1H), 3.69 (s, 3H), 3.67 (s, 3H), 1.67 (d, *J* = 7.1 Hz, 3H), 1.51 (d, *J* = 7.0 Hz, 3H); **^{13}C NMR ($CDCl_3$, 100 MHz)** NMR data reported are for major rotamer A: δ 171.6, 170.1, 167.4, 158.8, 133.7, 131.7, 127.7,

127.5, 123.1, 113.9, 56.9, 55.1, 52.2, 51.9, 47.3, 15.4, 14.7. **HRMS (ESI)** calcd for $C_{23}H_{25}N_2O_6$ $[M+H]^+$ 425.1707, found 425.1707.

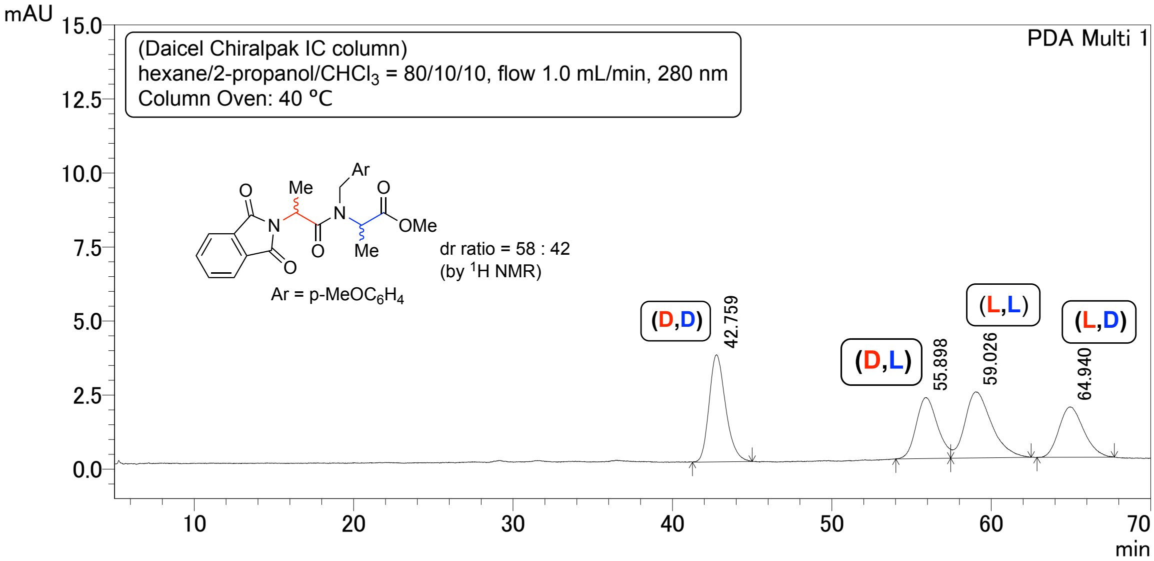

ピークテーブル

PDA Ch1 280nm 4nm

ピーク#	保持時間	面積	面積%
1	42.759	260649	29.124
2	55.898	190498	21.286
3	59.026	260162	29.070
4	64.940	183651	20.521
合計		894960	100.000

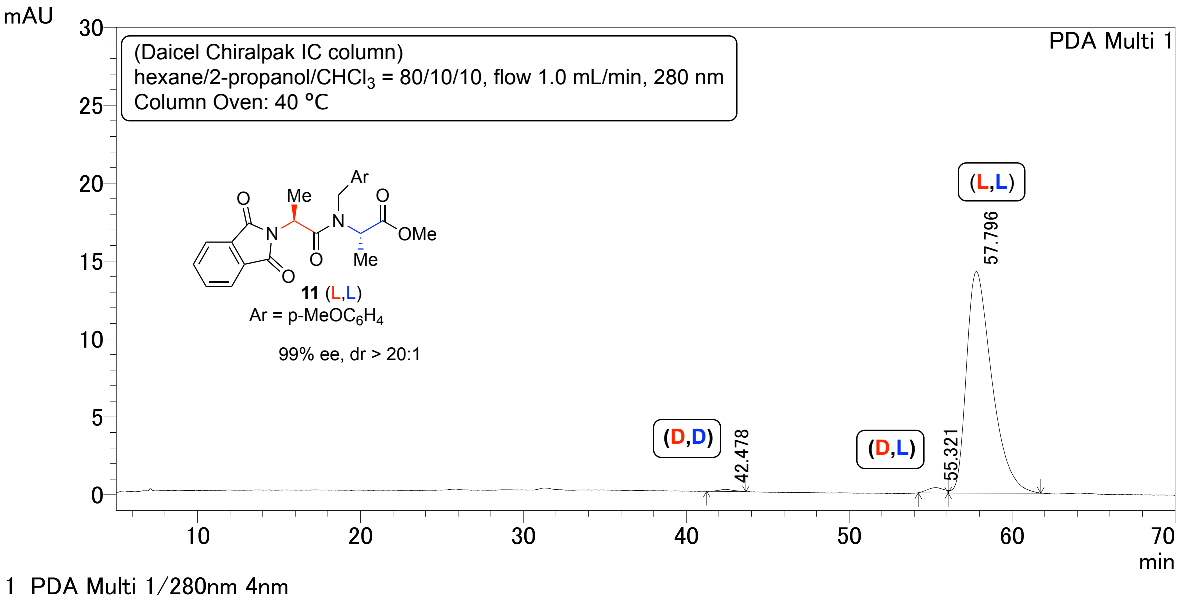

1 PDA Multi 1/280nm 4nm

PDA Ch1 280nm 4nm

ピーク#	保持時間	面積	面積%
1	42.478	7782	0.495
2	55.321	24203	1.539
3	57.796	1540719	97.966
合計		1572704	100.000

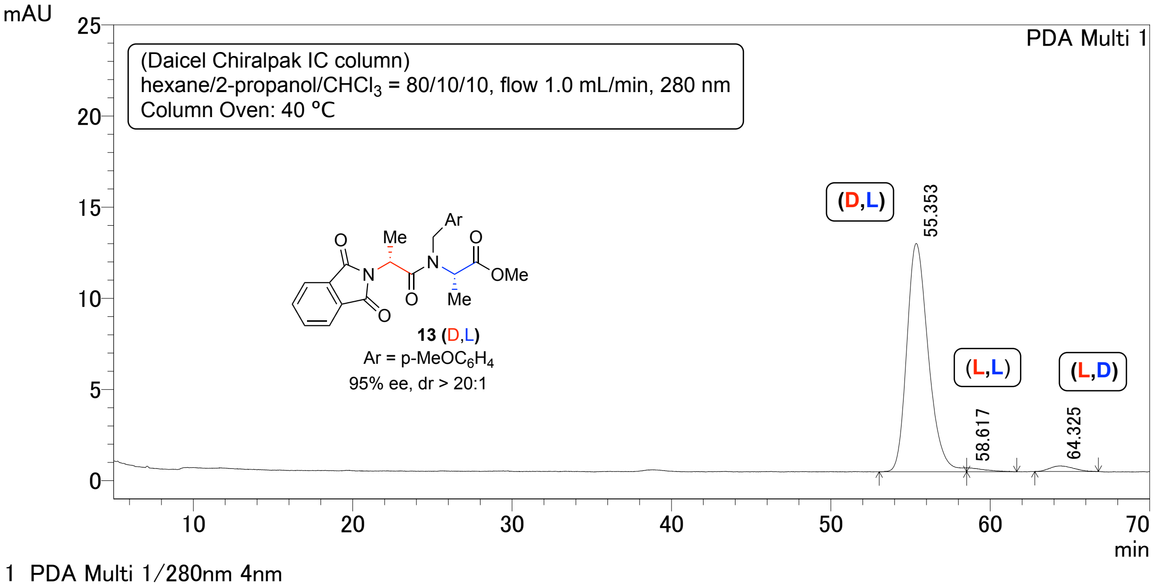

1 PDA Multi 1/280nm 4nm

PDA Ch1 280nm 4nm

ピーク#	保持時間	面積	面積%
1	55.353	1173658	96.034
2	58.617	16671	1.364
3	64.325	31802	2.602
合計		1222131	100.000

12. Procedure for transformation of dipeptides 6, *dl*-8, 11, and 13 to corresponding new dipeptides 7, 10, 12, and 14

Scheme S10

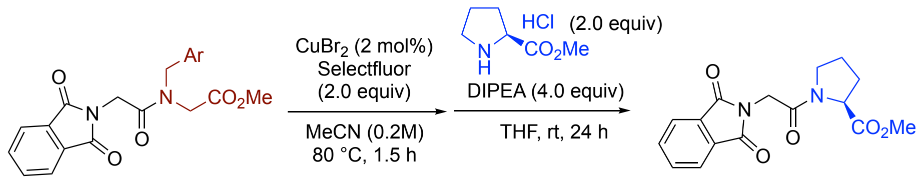

6 (Ar = *p*-MeOC$_6$H$_4$) **7**, 53% yield

$CuBr_2$ (0.9 mg, 4 μmmol, 2 mol% of Cu), Selectfluor (142 mg, 0.40 mmol), and dipeptide **6** (79.2 mg, 0.20 mmol), were placed in an oven dried 10 mL Schlenk tube. The tube was evacuated and then re-filled with argon gas for 3 cycles. MeCN (1.0 mL) were added successively. The tube was placed in a preheated oil bath at 80 °C and stirred for 1.5 h equipped with argon balloon. The reaction was cool down to rt, the mixture was passed through a short pad of silica gel with CH_2Cl_2 as eluent. Volatiles were removed on a rotary evaporator, and the residuals were dissolved in THF (2 mL) and L-proline methyl ester •HCl [CAS: 2133-40-6] (66.2 mg, 0.4 mmol) and DIPEA (56 μL, 0.8 mmol) was added in sequence to the reaction mixture at 0 ˚C. The reaction was stirred at rt for 24 h with argon balloon. Volatiles were removed on a rotary evaporator, and the crude mixtures was purified by silica gel flash chromatography with CH_2Cl_2 and ethyl acetate as eluents. Dipeptide **7** was obtained as white solid in 53% yield.

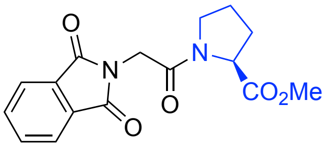

7

Methyl (2-(1,3-dioxoisoindolin-2-yl)acetyl)-*L*-prolinate (**7**)

7, white solid, 53% yield. **^{1}H NMR (CDCl$_3$, 400 MHz)** observed mixture of rotamers (A:B = 90:10), NMR data reported are for major rotamer A: δ 7.88–7.83 (m, 2H), 7.74–7.69 (m, 2H), 4.61–4.51 (m, 1H), 4.56 (d, *J* = 16.4 Hz, 1H), 4.40 (d, *J* = 16.4 Hz, 1H), 3.80–3.73 (m, 1H), 3.69 (s, 3H), 3.66–3.60 (m, 1H), 2.33–1.88 (m, 4H); **^{13}C NMR (CDCl$_3$, 100 MHz)** NMR data reported are for major rotamer A: δ 172.1, 167.8, 164.4, 134.0, 132.2, 123.4, 59.1, 52.2, 46.2, 39.6, 28.9, 24.8. **HRMS (ESI)** calcd for $C_{16}H_{17}N_2O_5$ [M+H]$^+$ 317.1132, found 317.1132.

Scheme S11

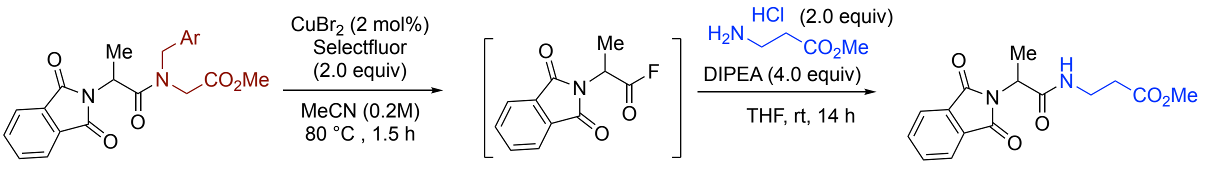

The reaction for synthesis of dipeptide **10** from *dl*-**8** was conducted in a same manner as described for dipeptide **7**. Dipeptide **10** was obtained as a colourless oil in 74% yield

The work up for the determination of NMR yield of compound **9**: After the first step reaction at 80 °C for 1.5 h under argon, the reaction was cooled down to rt, and the mixture was passed through a short pad of silica gel with ethyl acetate as eluent. Volatiles were removed on a rotary evaporator, and the crude mixture was subjected to NMR analysis. The yield of **9** in the crude mixture was determined by ^{19}F NMR using 4,4'-difluorobenzophenone as internal standard.

Figure S3. ^{19}F NMR chart of crude mixture. Illustration for determination of NMR yield of **9**

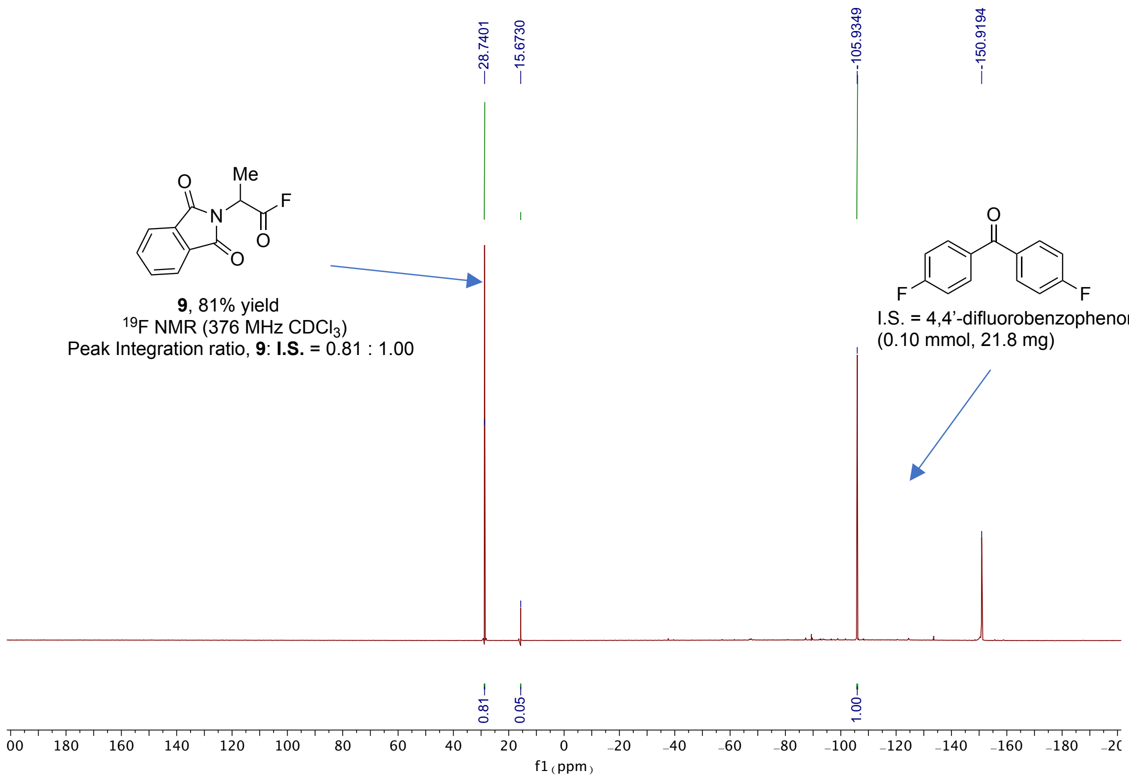

The peak observed at δ 15.7 is tentatively assigned as *p*-MeOC$_6$H$_4$(CO)F based on ^{1}H & ^{19}F NMR analysis of above crude mixture compared to our previous isolated authentic sample NMR data.[12]

[12] T. Yoshii, S. Tsuzuki, S. Sakurai, R. Sakamoto, J. Jiang, M. Hatanaka, A. Matsumoto, K. Maruoka, *Chem. Sci.* **2020**, *11*, 5772-5778.

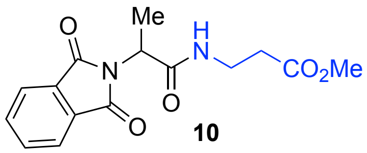

Methyl 3-(2-(1,3-dioxoisoindolin-2-yl)propanamido)propanoate (**10**)

10, colourless oil, 74% yield. **^{1}H NMR ($CDCl_3$, 400 MHz)** δ 7.88–7.83 (m, 2H), 7.76–7.72 (m, 2H), 6.67 (app s, 1H), 4.89 (q, *J* = 7.4 Hz, 1H), 3.65 (s, 3H), 3.61–3.44 (m, 2H), 2.62–2.49 (m, 2H), 1.67 (d, *J* = 7.4 Hz, 3H); **^{13}C NMR ($CDCl_3$, 100 MHz)** δ 173.0, 169.0, 167.6, 134.2, 131.8, 123.4, 51.7, 49.1, 35.2, 33.4, 15.1. **HRMS (ESI)** calcd for $C_{15}H_{17}N_2O_5$ $[M+H]^+$ 305.1132, found 305.1132.

Scheme S12

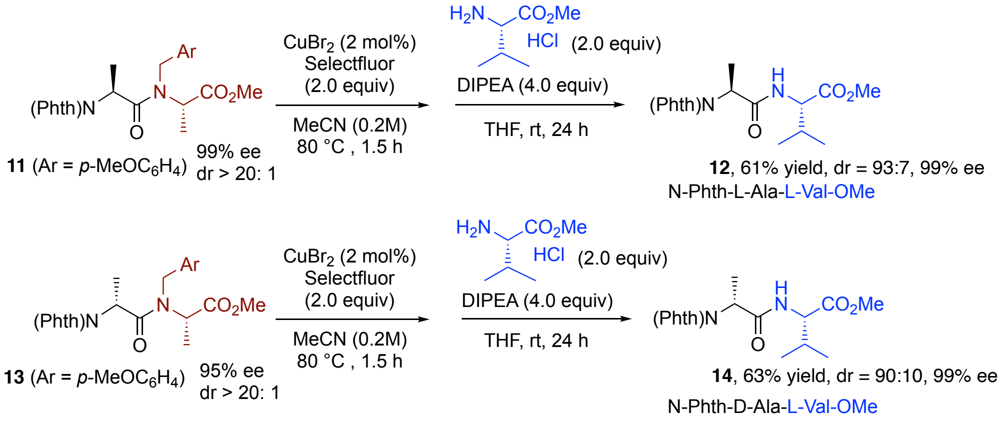

The reaction for synthesis of dipeptides **12** and **14** from dipeptides **11** and **13**, respectively, were conducted in a same manner as described for dipeptide **7**.

The reaction work up for the determination of dr ratio of **12** and **14**:

After the second step reaction at rt for 24 h, water was added to the reaction solution, and the mixture was extracted with ethyl acetate. The extracts were washed by 1M HCl aq. solution, and brine, dried over Na_2SO_4 and filtered. Volatiles were removed on a rotary evaporator, and the crude mixture was subjected to ^{1}H NMR analysis.

^{1}H NMR chart of crude reaction mixture – For illustration of determination of dr ratio for dipeptides **12** and **14**.

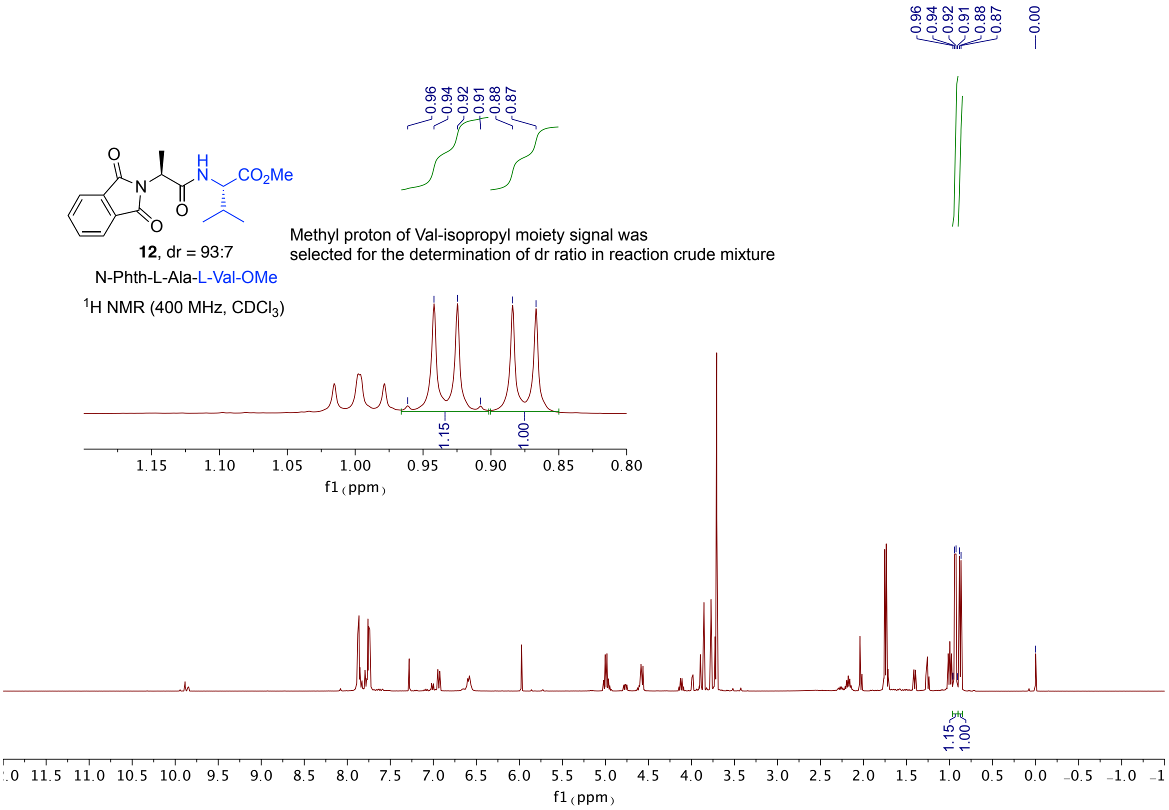

^{1}H NMR analysis of crude reaction mixture of dipeptide **14**

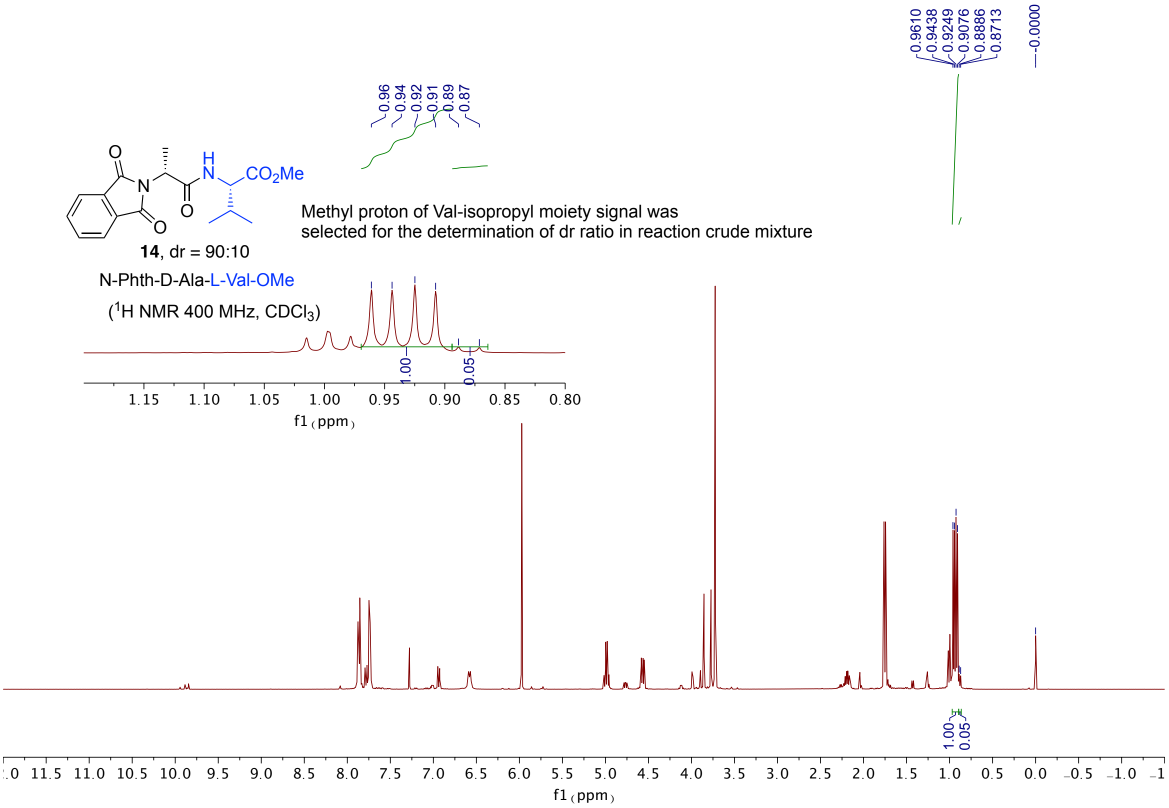

Characterization data of **12** and **14**

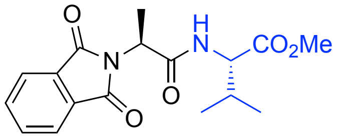

12

N-Phth-L-Ala-L-Val-OMe

<u>Methyl ((*S*)-2-(1,3-dioxoisoindolin-2-yl)propanoyl)-*L*-valinate</u> (**12**)

12, white solid, 61% yield; 99% ee, dr = 93:7; The ee was measured by HPLC (Daicel Chiralpak IC column), column oven: 40 ˚C, hexane/2-propanol/$CHCl_3$ = 80/10/10, flow 1.0 mL/min, 240 nm, t_{major} = 19.8 min, t_{minor} = 28.0 min; $[\alpha]^{28}_D$ +47.7 (*c* 1.00, $CHCl_3$). **^{1}H NMR ($CDCl_3$, 400 MHz)** δ 7.90–7.85 (m, 2H), 7.78–7.73 (m, 2H), 6.58 (d, *J* = 8.5 Hz, 1H), 5.00 (q, *J* = 7.4 Hz, 1H), 4.58 (dd, *J* = 8.5 Hz, 4.7Hz, 1H), 3.70 (s, 3H), 2.23–2.12 (m, 1H), 1.75 (d, *J* = 7.4 Hz, 3H), 0.93 (d, *J* = 6.9 Hz, 3H), 0.87 (d, *J* = 6.9 Hz, 3H); **^{13}C NMR ($CDCl_3$, 100 MHz)** δ 172.2, 169.1, 167.7, 134.3, 131.8, 123.5, 57.3, 52.1, 49.5, 31.3, 18.8, 17.6, 15.3. **HRMS (ESI)** calcd for $C_{17}H_{21}N_2O_5$ $[M+H]^+$ 333.1445, found 333.1446.

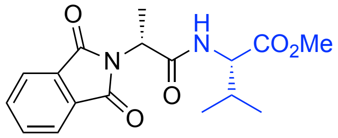

14

N-Phth-D-Ala-L-Val-OMe

<u>Methyl ((*R*)-2-(1,3-dioxoisoindolin-2-yl)propanoyl)-*L*-valinate</u> (**14**)

14, white solid, 63% yield. 99% ee, dr = 90:10; The ee was measured by HPLC (Daicel Chiralpak IC column), column oven: 40 ˚C, hexane/2-propanol/$CHCl_3$ = 80/10/10, flow 1.0 mL/min, 240 nm, t_{major} = 24.3 min, t_{minor} = 41.2 min; $[\alpha]^{28}_D$ +0.34 (*c* 1.00, $CHCl_3$). **^{1}H NMR ($CDCl_3$, 400 MHz)** δ 7.89–7.84 (m, 2H), 7.76–7.72 (m, 2H), 6.58 (d, *J* = 8.6 Hz, 1H), 4.99 (q, *J* = 7.4 Hz, 1H), 4.57 (dd, *J* = 8.6 Hz, 4.8 Hz, 1H), 3.72 (s, 3H), 2.24–2.13 (m, 1H), 1.75 (d, *J* = 7.4 Hz, 3H), 0.95 (d, *J* = 6.9 Hz, 3H), 0.92 (d, *J* = 6.9 Hz, 3H); **^{13}C NMR ($CDCl_3$, 100 MHz)** δ 172.2, 168.9, 167.7, 134.2, 131.8, 123.5, 57.4, 52.1, 49.3, 31.3, 18.8, 17.7, 15.3. **HRMS (ESI)** calcd for $C_{17}H_{21}N_2O_5$ $[M+H]^+$ 333.1445, found 333.1447.

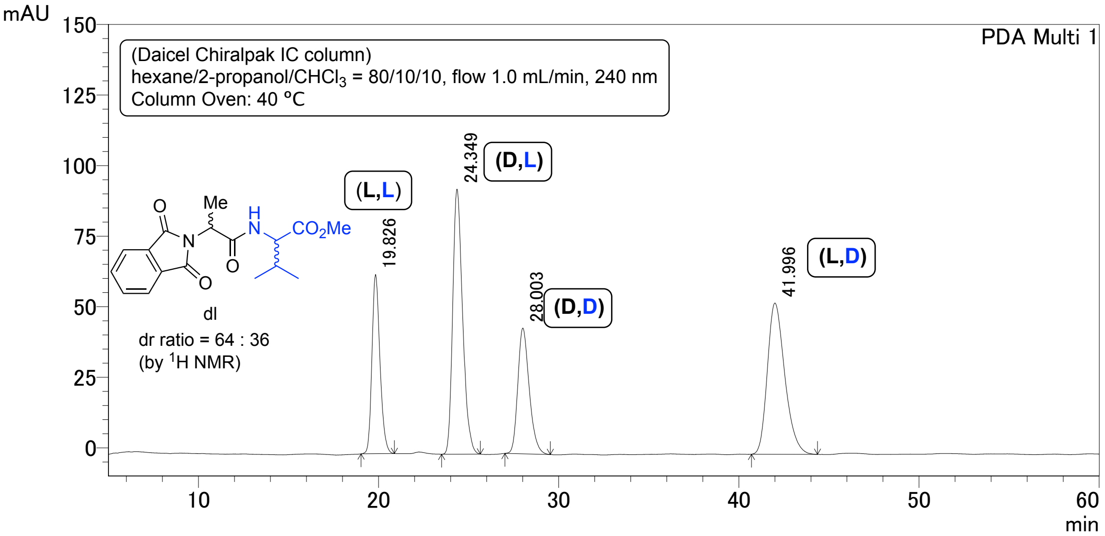

1 PDA Multi 1/240nm 4nm

PDA Ch1 240nm 4nm

ピーク#	保持時間	面積	面積%
1	19.826	1959888	17.637
2	24.349	3586124	32.272
3	28.003	1963015	17.665
4	41.996	3603288	32.426
合計		11112314	100.000

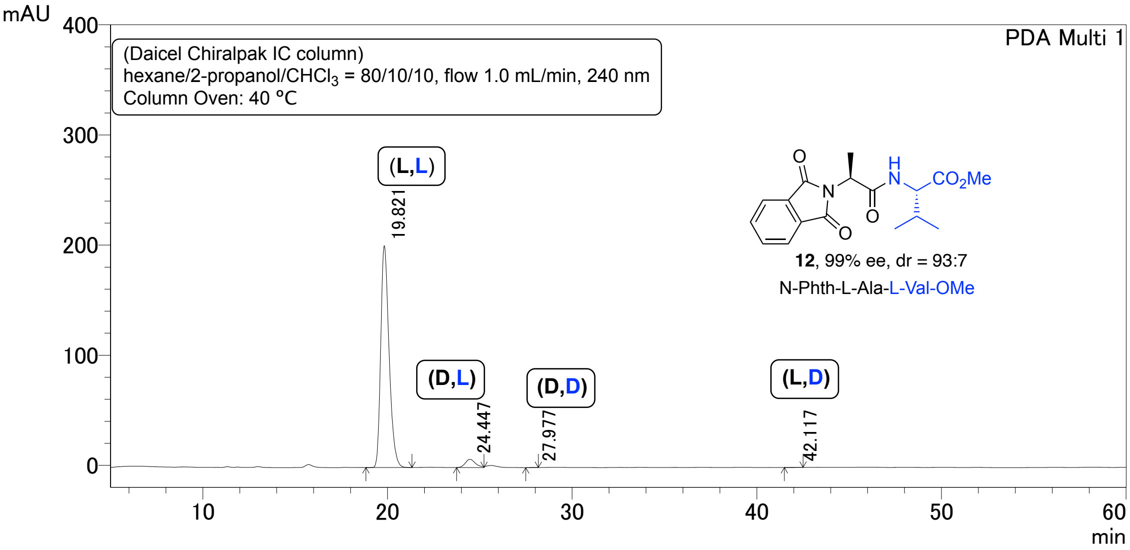

1 PDA Multi 1/240nm 4nm

ﾋﾟｰｸﾃｰﾌﾞﾙ

PDA Ch1 240nm 4nm

ピーク#	保持時間	面積	面積%
1	19.821	6294703	95.602
2	24.447	287678	4.369
3	27.977	345	0.005
4	42.117	1530	0.023
合計		6584256	100.000

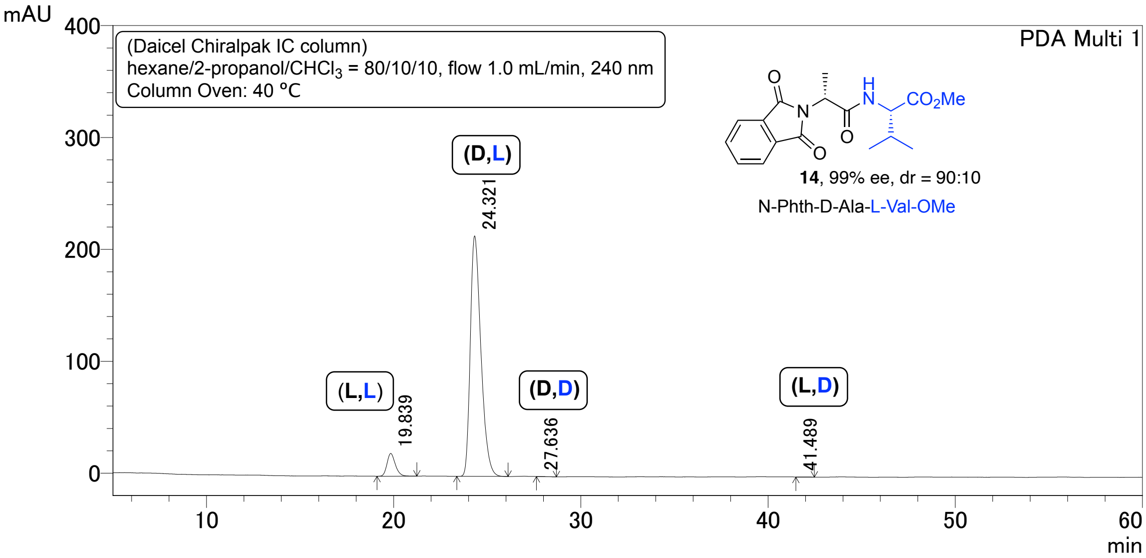

1 PDA Multi 1/240nm 4nm

PDA Ch1 240nm 4nm

ピーク#	保持時間	面積	面積%
1	19.839	625804	6.893
2	24.321	8445343	93.019
3	27.636	3515	0.039
4	41.489	4472	0.049
合計		9079134	100.000

13. Experiments for Scheme 4

Radical scavenger experiment (Scheme 4a)

Scheme S13

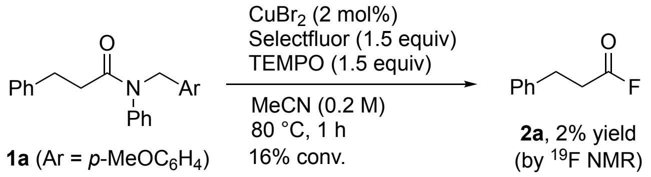

$CuBr_2$ (0.9 mg, 4 μmmol, 2 mol% of Cu), Selectfluor (106.3 mg, 0.30 mmol), *N*-(4-methoxybenzyl)-*N*-phenyl-3-phenylpropanamide (**1a**) (69.1 mg, 0.20 mmol), and TEMPO (46.9 mg, 0.30 mmol) were placed in an oven dried 10 mL Schlenk tube. The tube was evacuated and then re-filled with argon gas for 3 cycles. MeCN (1.0 mL) was added successively. The tube was placed in a preheated oil bath at 80 °C and stirred for 1 h equipped with argon balloon. The reaction was cooled down to rt, and the mixture was passed through a short pad of silica gel with ethyl acetate as eluent. Volatiles were removed on a rotary evaporator, and the crude mixture was subjected to NMR analysis. Conversion of **1a** and yield of **2a** in the crude mixture was determined by ^{1}H and ^{19}F NMR using $Cl_2CHCHCl_2$ and 4,4'-difluorobenzophenone as internal standard, respectively.

Photoirradiation experiments (Scheme 4b)

Scheme S14

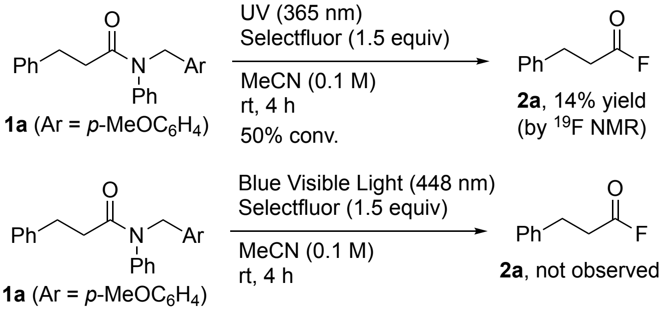

Selectfluor (106.3 mg, 0.30 mmol), and *N*-(4-methoxybenzyl)-*N*-phenyl-3-phenylpropanamide (**1a**) (69.1 mg, 0.20 mmol) were placed in an oven dried 10 mL reaction tube shielded with aluminium foil and with an internal light source (Techno Sigma-PER-AMP, 365 nm or 448 nm). The tube was evacuated and then re-filled with argon gas for 3 cycles. MeCN (2.0 mL) was added successively. After confirming the light source tip soaked with reaction solution, the tube was placed in a water bath (EYELA reactor) at rt and stirred for 4 h under the irradiation of UV light (365 nm) or blue visible light (448 nm) under protection with argon balloon. The mixture was passed through a short pad of silica gel with ethyl acetate as eluent. Volatiles were removed on a rotary evaporator, and the crude mixture was subjected to NMR analysis. Conversion of **1a** and yield of **2a** in the crude mixture was determined by ^{1}H and ^{19}F NMR using $Cl_2CHCHCl_2$ and 4,4'-difluorobenzophenone as internal standard, respectively.

Pictures: illustration for the reaction setup

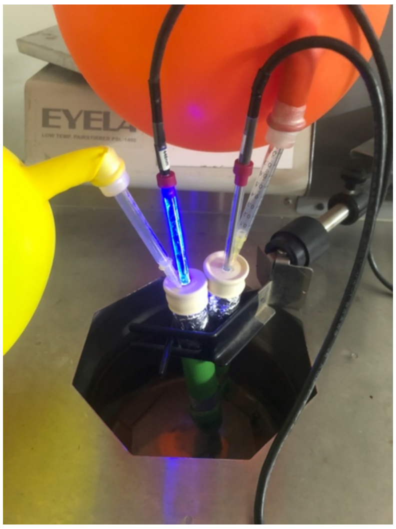

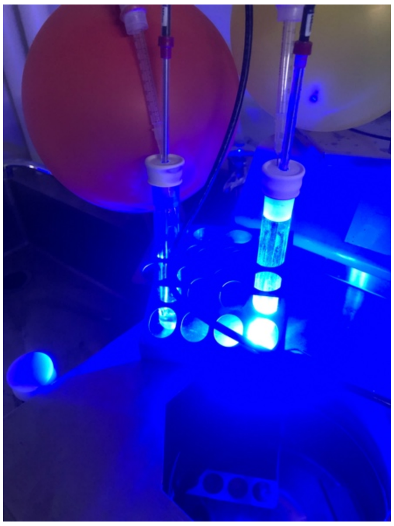

Experimental procedure for Scheme 4c

Synthesis of **15**

Tertiary amide **15** was prepared using method A (as described for the synthesis of **1a**). Secondary amine 2-((4-methoxybenzyl)amino)ethan-1-ol (**A15**)[13] [CAS: 64834-63-5] was prepared according to the literature, and it was used for the synthesis of **15**.

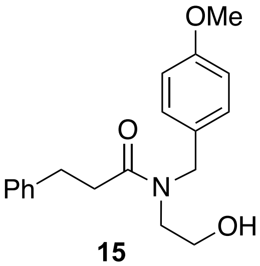

15

N-(2-hydroxyethyl)-*N*-(4-methoxybenzyl)-3-phenylpropanamide (**15**)

15, colourless oil, yield 78% (1.23 g). **^{1}H NMR ($CDCl_3$, 400 MHz)** observed mixture of rotamers (A:B = 70:30), NMR data are reported for mixture of rotamers: δ 7.29–7.15 (m, 5H, A & B), 7.12 (d, *J* = 8.7 Hz, 0.6H, B), 6.98 (d, *J* = 8.7 Hz, 1.4H, A), 6.86–6.80 (m, 2H, A & B), 4.58 (s, 0.6H, B), 4.42 (s, 1.4H, A), 3.78 (s, 2.1H, A), 3.77 (s, 0.9H, B), 3.69 (t, *J* = 5.2 Hz, 1.4H, A), 3.65–3.55 (m, 0.6H, B), 3.60 (br s, 0.7H, A), 3.51 (t, *J* = 5.2 Hz, 1.4H, A), 3.32 (t, *J* = 5.6 Hz, 0.6H, B), 3.03–2.97 (m, 2H, A & B), 2.90 (br s, 0.3H, B), 2.77 (t, *J* = 7.8 Hz, 0.6H, B), 2.69 (t, *J* = 7.8 Hz, 1.4H, A); **^{13}C NMR ($CDCl_3$, 100 MHz)** No assignments of rotamers, the observed peaks are summarized as follow, δ 174.7, 173.0, 159.1, 158.8, 141.2, 140.9, 129.7, 129.3, 128.44, 128.39, 128.0, 127.5, 126.2, 126.0, 114.2, 113.9, 61.9, 59.9, 55.2, 55.2, 51.9, 49.6, 48.6, 48.0, 35.1, 35.0, 31.5, 31.4, (one aromatic carbon signal overlapping for both rotamers A & B). **HRMS (ESI)** calcd for $C_{19}H_{24}NO_3$ $[M+H]^+$ 314.1751, found 314.1752.

[13] S. Xia, Y. Song, X. Li, H. Li, L.-N. He, *Molecules*, **2018**, *23*, 3033.

Synthesis of **17**

Scheme S15

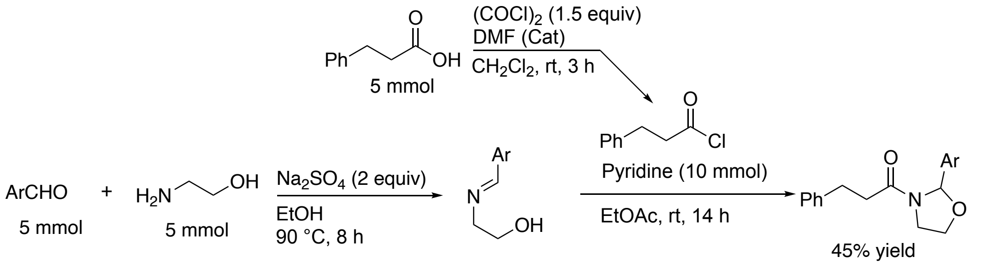

17 (Ar = *p*-MeOC$_6$H$_4$)

Compound **17** was prepared with a modified procedure according to the literature[14].

A solution of *p*-methoxybenzaldyhyde (0.68 g, 5 mmol), 2-aminoethanol (0.31 g, 5 mmol), and Na_2SO_4 (1.42 g, 10 mmol) in ethanol (30 mL) was refluxed at 90 °C for 8 h under argon balloon protection. The solution was filtered and volatiles were removed under vacuum condition to gave the imine as yellowish oil, which was directly used for subsequent step without any further purification.

To a solution of 3-phenylpropanoic acid (0.75 g, 5 mmol) in CH_2Cl_2 (5 mL) was added two drops of DMF followed by oxalyl chloride (0.64 mL, 7.5 mmol) dropwise at 0 ˚C, and the mixture was stirred at rt for 3 h under argon. After removing the volatiles by rotary evaporator, the residuals were dissolved in ethyl acetate (5 mL) and added to a solution of above freshly prepared imine and pyridine (0.81 ml, 10 mmol) in ethyl acetate (5 mL) at 0 ˚C. The mixture was stirred at rt for 14 h. The solution was then filtered and evaporation of the solvent followed by flash column chromatography on silica gel using hexane and ethyl acetate as eluents gave **17** as colourless oil in 45% yield.

[14] M. Nechab, D. N. Kumar, C. Philouze, C. Einhorn, J. Einhorn, *Angew. Chem. Int. Ed.* **2007**, *46*, 3080–3083; *Angew. Chem.* **2007**, *119*, 3140–3143.

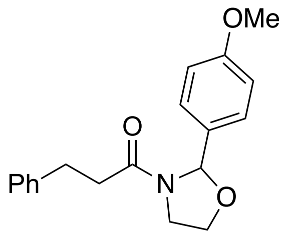

17

1-(2-(4-Methoxyphenyl)oxazolidin-3-yl)-3-phenylpropan-1-one (**17**)

17, colourless oil, yield 45% (0.70 g). **^{1}H NMR ($CDCl_3$, 400 MHz)** observed mixture of rotamers (A:B = 55:45), NMR data are reported for mixture of rotamers; δ 7.30–7.13 (m, 5.9H, A & B), 7.01 (d, J = 7.0 Hz, 1.1H, A), 6.89–6.84 (m, 2H, A & B), 6.29 (s, 0.45H, B), 5.90 (s, 0.55H, A), 4.08–3.90 (m, 2.55H, A & B), 3.80 (s, 1.65H, A), 3.78 (s, 1.35H, B), 3.68–3.50 (m, 1.45H, A & B), 3.05–2.87 (m, 1.45H, A & B), 2.75 (ddd, J = 14.8 Hz, 9.0 Hz, 6.2 Hz, 0.55H, A), 2.68–2.60 (m, 0.9H, B), 2.38 (ddd, J = 15.1 Hz, 8.6 Hz, 6.4 Hz, 0.55H, A), 2.25 (ddd, J = 15.4 Hz, 9.2 Hz, 6.2 Hz, 0.55H, A); **^{13}C NMR ($CDCl_3$, 100 MHz)** No assignments of rotamers, the observed peaks are summarized as follow, δ 169.9(A & B overlapping), 160.3, 159.7, 140.9, 140.8, 130.8, 130.4, 128.5, 128.4, 128.3, 128.2, 127.9, 127.8, 126.2, 126.0, 114.2, 113.6, 89.0, 88.8, 65.5, 64.5, 55.2, 55.2, 45.3, 44.9, 37.1, 36.7, 30.9, 30.8. **HRMS (ESI)** calcd for $C_{19}H_{21}NO_3Na$ $[M+Na]^+$ 334.1414, found 334.1420.

Scheme S16

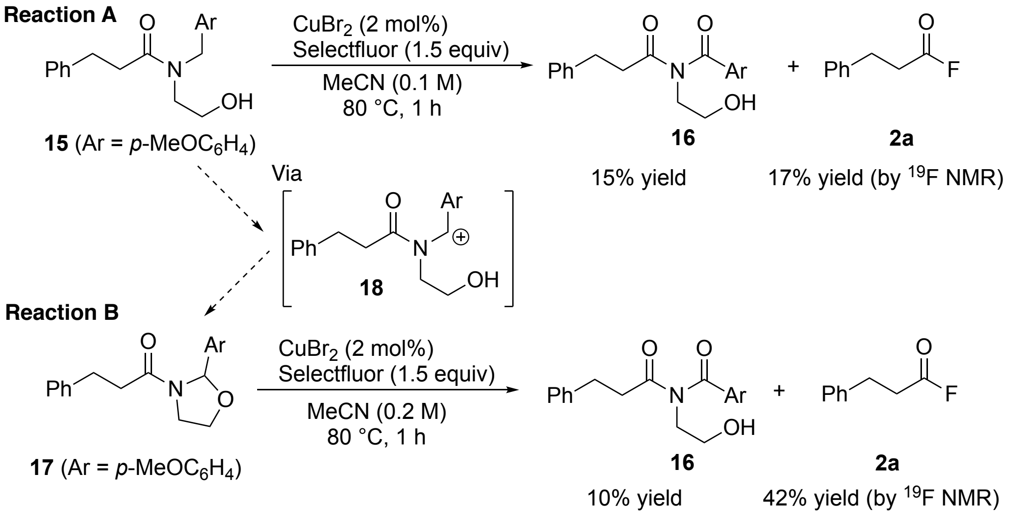

Procedure for reaction A

$CuBr_2$ (0.9 mg, 4 µmmol, 2 mol% of Cu), Selectfluor (106.3 mg, 0.30 mmol), and **15** (62.7 mg, 0.20 mmol), were placed in an oven dried 10 mL Schlenk tube. The tube was evacuated and then re-filled with argon gas for 3 cycles. MeCN (2.0 mL) was added successively. The tube was placed in a preheated oil bath at 80 °C and stirred for 1 h equipped with argon balloon. The reaction was cooled down to rt, and the mixture was passed through a short pad of silica gel with ethyl acetate as eluent. Volatiles were removed on a rotary evaporator, and the crude mixture was subjected to NMR analysis. After the yield of **2a** in the crude mixture was determined by ^{19}F NMR using 4,4'-difluorobenzophenone as internal standard, the crude mixture was subjected for flash column chromatography on silica gel using hexane and ethyl acetate as eluents to isolate the imide **16** as white solid in 15% yield and 17% NMR yield of acyl fluoride **2a** was realized. In reaction A, no production of **17** was observed.

Procedure for reaction B

Reaction B of compound **17** with Selectfluor and $CuBr_2$ was conducted in a same procedure as described for reaction A except for using 1 mL of MeCN as reaction solvent. The reaction B gave imide **16** in 10% yield and acyl fluoride **2a** in 42% NMR yield.

Interpretation of reactions A and B

These results suggest compound **17** may be in-situ formed and converted in the course of reaction **A**, probably via the cation intermediate **18** by favourable intramolecular reaction with its hydroxyl moiety. As shown in reaction B, the resulted **17** can be further converted to imide **16** and acyl fluoride **2a**, respectively (same products in reaction A).

Attempts on the identification and isolation of imide type products with other substrates, for example, *N*-(4-methoxybenzyl)-*N*-phenyl-3-phenylpropanamide (**1a**) and *N*-cyclohexyl-*N*-(4-methoxybenzyl)-3-phenylpropanamide (**1v**) were unsuccessful under the same reaction condition of reaction B.

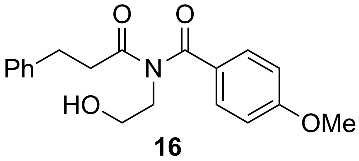

16

N-(2-hydroxyethyl)-4-methoxy-*N*-(3-phenylpropanoyl)benzamide (**16**)

16, white solid. NMR data reported are for major rotamer A. **^{1}H NMR ($CDCl_3$, 400 MHz)** mixture of rotamers (A:B = 90:10), δ. 7.95 (d, *J* = 9.0 Hz, 2H), 7.28–7.13 (m, 5H), 6.92 (d, *J* = 9.0 Hz, 2H), 5.82 (br s, 1H), 4.31 (t, *J* = 5.2 Hz, 2H), 3.60 (s, 3H), 3.60 (q, *J* = 5.6 Hz, 2H), 2.96 (t, *J* = 7.7 Hz, 2H), 2.96 (t, *J* = 7.7 Hz, 2H); **^{13}C NMR ($CDCl_3$, 100 MHz)** δ 172.3, 166.4, 163.5, 140.7, 131.7, 128.5, 128.3, 126.2, 122.0, 113.6, 63.4, 55.4, 38.9, 38.4, 31.6. HRMS (ESI) calcd for $C_{19}H_{22}NO_4$ $[M+H]^+$ 328.1543, found 328.1537.

14. Study of deuterium kinetic isotope effect (KIE experiments)

Synthesis of **1a**-d_2

4-Methoxybenzenemethan-d_2-ol [CAS: 35693-15-3][15] was prepared according to literature method. The reaction procedure shown below was modified according to the literature[16] for the synthesis of *p*-methoxy-*N*-phenylbenzylamine from *p*-methoxybenzyl alcohol.

Scheme S17

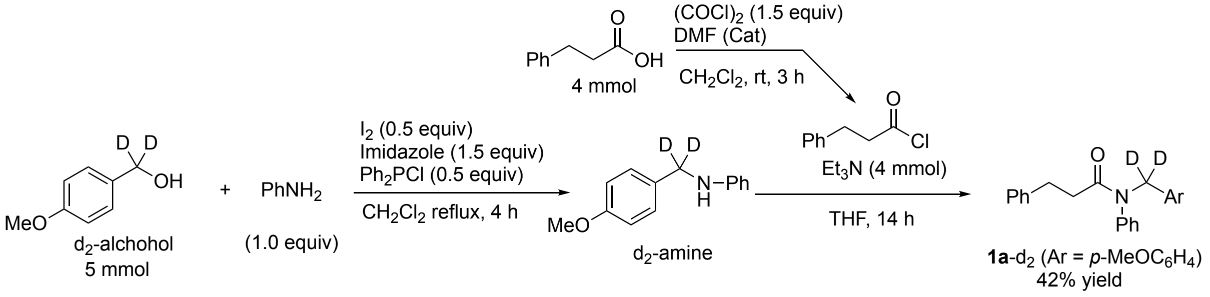

To a mixture of chlorodiphenylphosphine (552 mg, 2.5 mmol), imidazole (511 mg, 7.5 mmol), and iodine (635 mg, 2.5 mmol) in CH_2Cl_2 was added 4-methoxybenzenemethan-d_2-ol [CAS: 35693-15-3] followed by aniline (466 mg, 5 mmol), and the mixture was refluxed for 4 h. The solution was washed with saturated aq. Na_2CO_3, sodium thiosulfate, and brine. The extracts was dried over Na_2SO_4 and filtered. Evaporation of the solvent followed by flash column chromatography on silica gel using hexane and ethyl acetate as eluents gave the crude d_2-amine as inseparable mixture as colourless oil, which was applied to the subsequent step without further purification.

To a solution of 3-phenylpropanoic acid (600 mg, 4 mmol) in CH_2Cl_2 (5 mL) was added two drops of DMF followed by oxalyl chloride (0.52 mL, 6 mmol) dropwise at 0 ˚C, and the mixture was stirred at rt for 3 h under argon. After removing the volatiles by rotary evaporator, the residuals were dissolved in THF (5 mL), and the crude d_2-amine (pre-dissolved in 4 mL of THF) and Et_3N (0.56 mL, 4 mmol) were added in sequence at 0 ˚C. The solution was stirred at rt for 14 h under argon. After adding water, the reaction mixture was extracted with ethyl acetate, and the extracts were washed by Na_2CO_3 aq. solution, and brine, dried over Na_2SO_4 and filtered. Evaporation of the solvent followed by flash column chromatography on silica gel using hexane and ethyl acetate as eluents gave **1a**-d_2 as colourless oil in 42% yield (730 mg) (D>99%).

---

[15] R. K. Zhang, K. Chen, X. Huang, L. Wohlschlager, H. Renata, F. H. Arnold, *Nature*, **2019**, *565*, 67–72.
[16] N. Nowrouzi, M. Z. Jonaghani, *Can. J. Chem.* **2012**, *90*, 498–509.

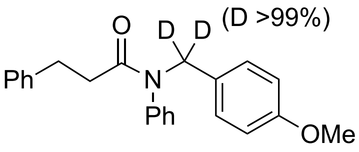

1a-d2

N-((4-methoxyphenyl)methyl-*d*2)-*N*,3-diphenylpropanamide (**1a**-d2)

1a-d2, colourless oil, 42% yield (0.73 g). **^{1}H NMR (CDCl$_3$, 400 MHz)** δ 7.27–7.13 (m, 6H), 7.07–7.03 (m, 4H), 6.78–6.74 (m, 4H), 3.76 (s, 3H), 2.93 (t, *J* = 7.7 Hz, 2H), 2.34 (t, *J* = 7.7 Hz, 2H); **^{13}C NMR (CDCl$_3$, 100 MHz)** δ 171.8, 158.8, 142.1, 141.1, 130.1, 129.5, 129.4, 128.4, 128.3, 127.8, 126.0, 113.6, 55.1, 51.8(C-D coupling not observed), 36.1, 31.7, (one aromatic carbon signal overlapping). **HRMS (ESI)** calcd for $C_{22}H_{23}D_2NO_2$ [M+H]$^+$ 348.1927, found 348.1921.

NMR chart for the illustration of D incorporation in 1a-d2 (D>99%)

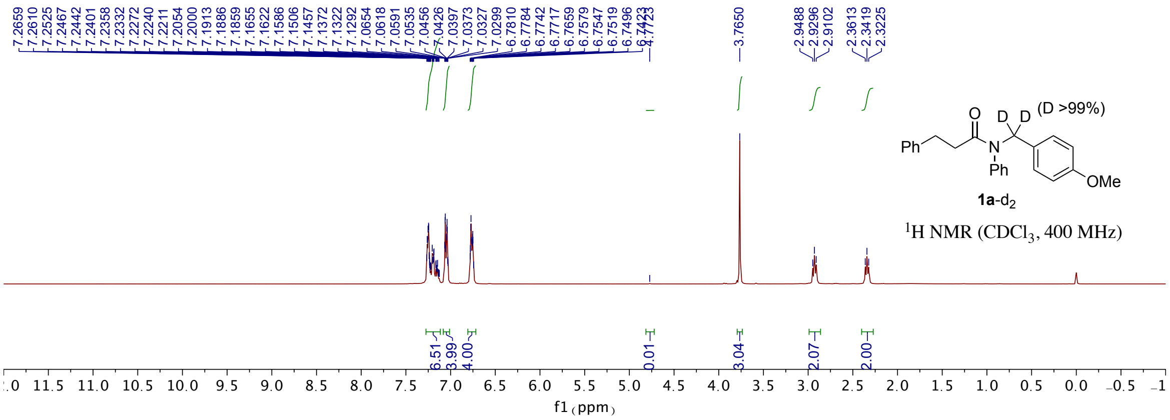

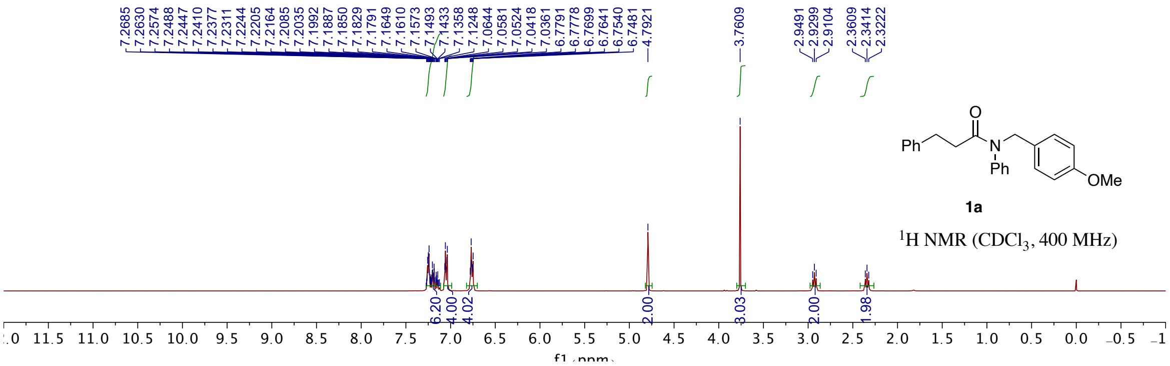

Intermolecular competitive reaction

Scheme S18

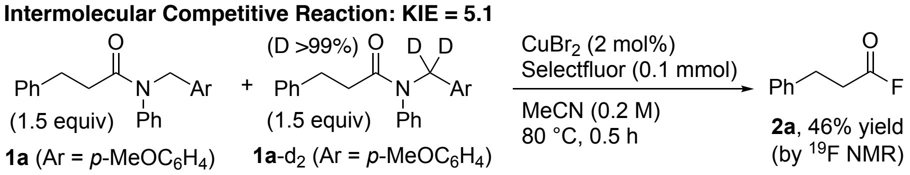

$CuBr_2$ (0.45 mg, 2 μmmol, 2 mol% of Cu), Selectfluor (35.4 mg, 0.10 mmol), *N*-(4-methoxybenzyl)-*N*-phenyl-3-phenylpropanamide (**1a**) (51.8 mg, 0.15 mmol), and **1a**-d_2 (52.1 mg, 0.15 mmol) were placed in an oven dried 10 mL Schlenk tube. The tube was evacuated and then re-filled with argon gas for 3 cycles. MeCN (0.5 mL) was added successively. The tube was placed in a preheated oil bath at 80 °C and stirred there for 0.5 h equipped with argon balloon. The reaction mixture was cooled down to rt, and the mixture was passed through a short pad of silica gel with ethyl acetate as eluent. Volatiles were removed on a rotary evaporator, and the crude mixture was subjected to NMR analysis. Conversion of **1a** and **1a**-d_2, and yield of **2a** in the crude mixture was determined by ^1H and ^{19}F NMR using $Cl_2CHCHCl_2$ and 4,4'-difluorobenzophenone as internal standard, respectively.

KIE was calculated based on the following equation[17,18].

$$KIE_{Cal} = \frac{\ln(1-F)}{\ln[(1-F)\,R/R_0]}$$

F: fractional conversion of reactants.
R/R_0: The proportion of a major isotopic component in recovered material compared to the original starting material.

$KIE_{H/D}$ was calculated based on crude ^1H NMR analysis as shown in the following charts.

$F = 0.25$

R = (**1a**-d_2)/(**1a**-d_2+ **1a**) = 0.63 (R: molar ratio)

$R_0 = 0.5$ (R_0: molar ratio)

$R/R_0 = 1.26$

$KIE_{H/D} = 5.1$

[17] L. Melander, W. H., Saunders, Jr. in *Reactions Rates of Isotopic Molecules*, Wiley, New York, **1980**, pp 95–102.
[18] D. A. Singleton, A. A. Thomas, *J. Am. Chem. Soc.* **1995**, *117*, 9357–9358.

The ^{1}H and ^{19}F NMR chart of reaction crude mixture analysis are shown as follow:

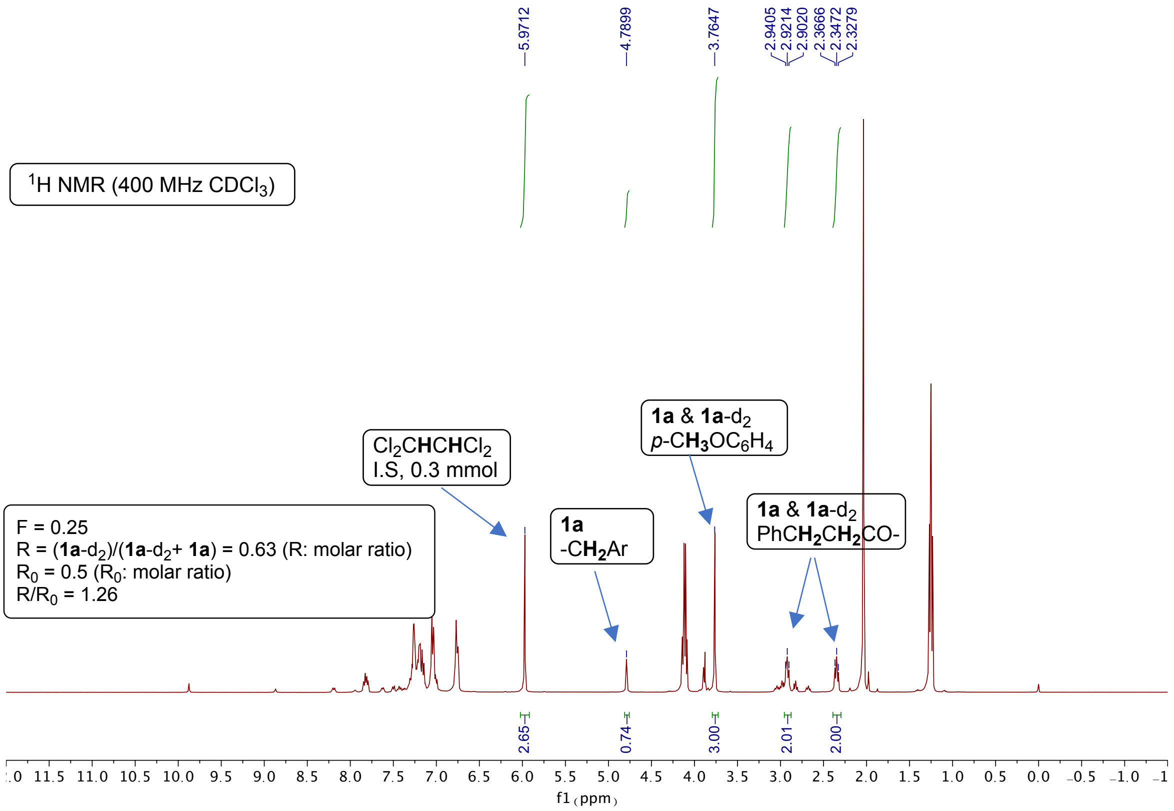

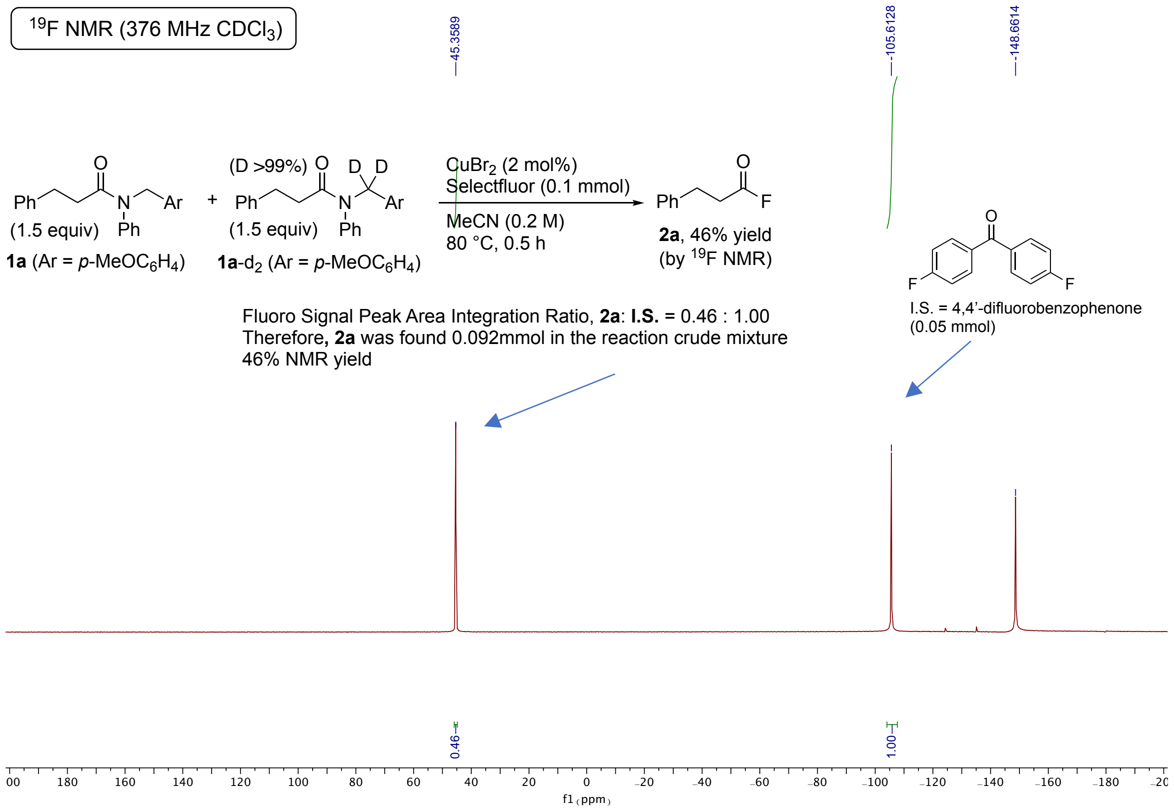

KIE study parallel reaction

Scheme S19

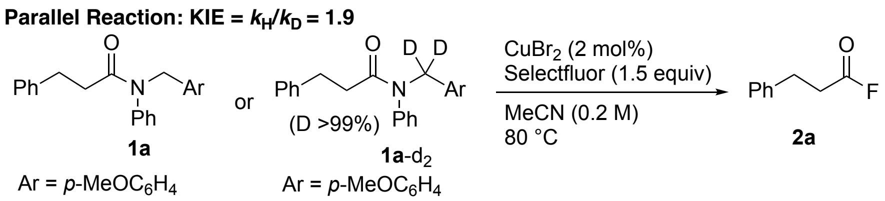

$CuBr_2$ (0.45 mg, 2 μmol, 2 mol% of Cu), Selectfluor (53.1 mg, 0.15 mmol), and *N*-(4-methoxybenzyl)-*N*-phenyl-3-phenylpropanamide (**1a**) (34.6 mg, 0.20 mmol), were placed in an oven dried 10 mL Schlenk tube. The tube was evacuated and then re-filled with argon gas for 3 cycles. MeCN (0.5 mL) was added successively. The tube was placed in a preheated oil bath at 80 °C and stirred for 4 min equipped with argon balloon. The reaction tube was immediately removed from oil bath and ethyl acetate (2 ml) was quickly added. The mixture was passed through a short pad of silica gel with ethyl acetate as eluent. Volatiles were removed on a rotary evaporator, and the crude mixture was subjected to NMR analysis. Yield of **2a** in the crude mixture was determined by ^{19}F NMR using 4,4'-difluorobenzophenone as internal standard.

The above motioned reaction was also conducted for 1 min, 2 min, and 3 min of reaction time, respectively, to complete the time dependent curve of yield of **2a** using **1a** as substrate.

Reaction of **1a**-d2 followed the same procedure as described above. The reactions were conducted for 2 min, 3 min, 4 min, and 5 min reaction time, respectively, to complete the time dependent curve of yield of **2a** using **1a**-d2 as substrate.

KIE = k_H/k_D = 22.9/11.8 = 1.94

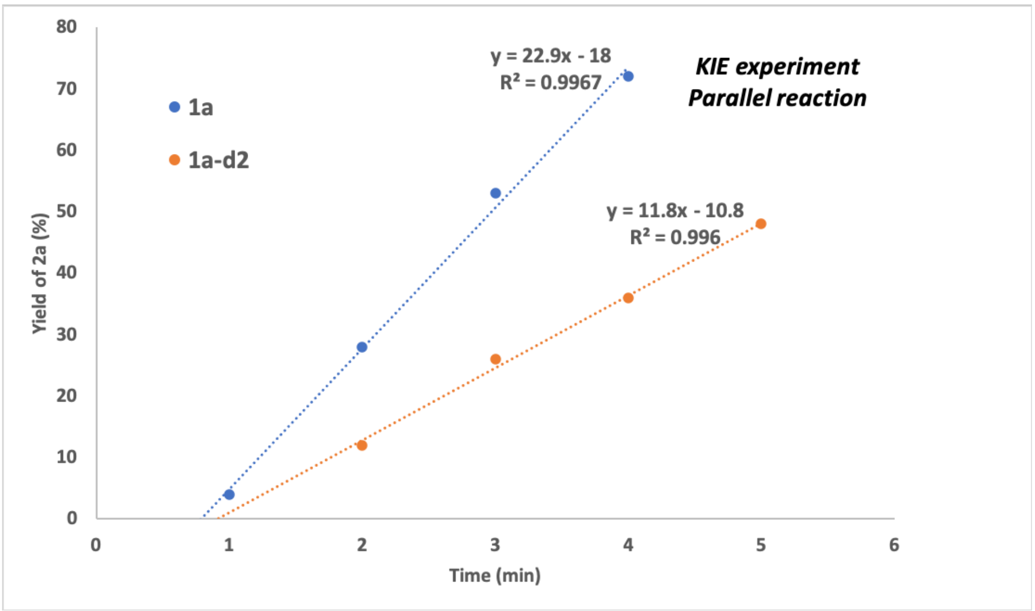

Raw data for ^{19}F NMR charts for the above mentioned reactions are shown as follow:

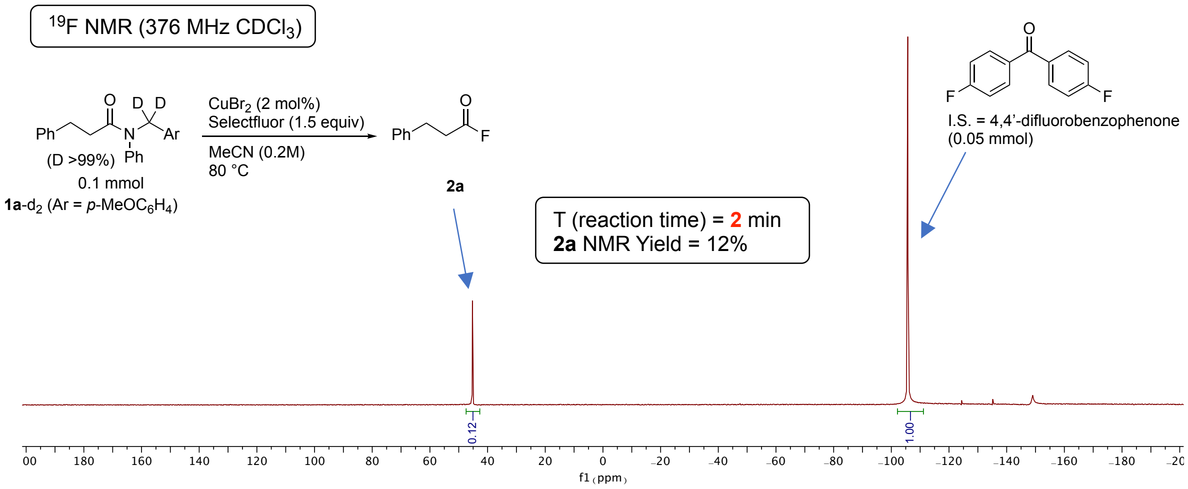

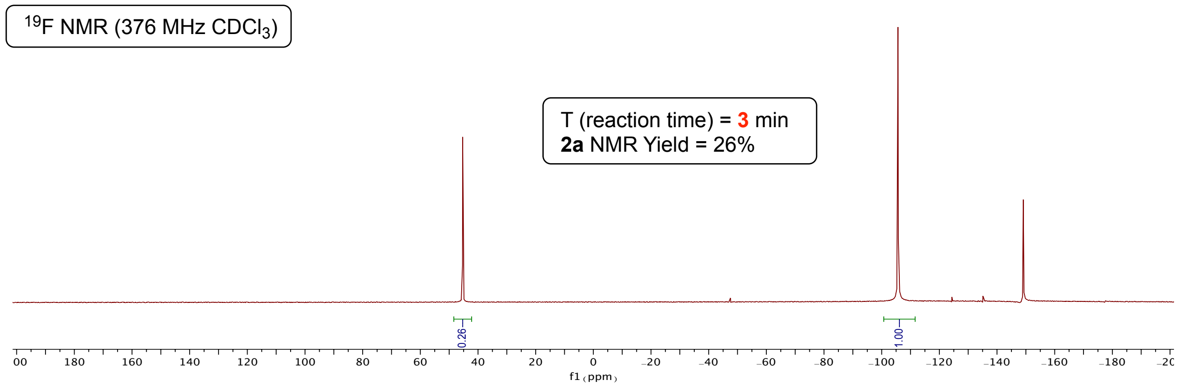

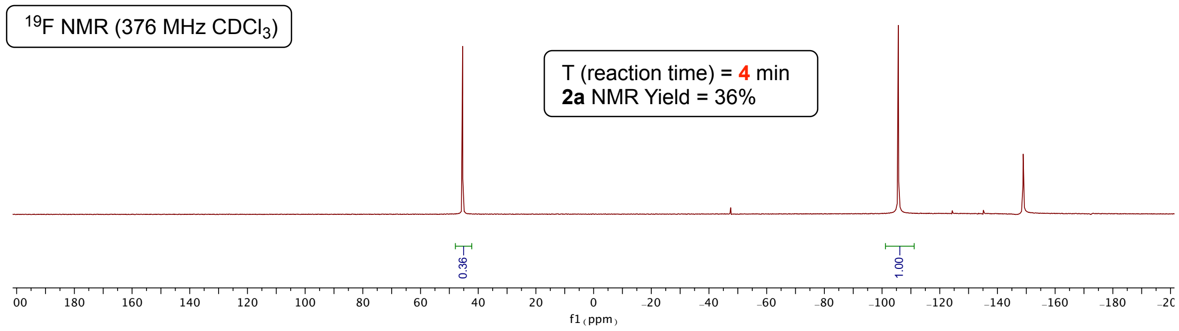

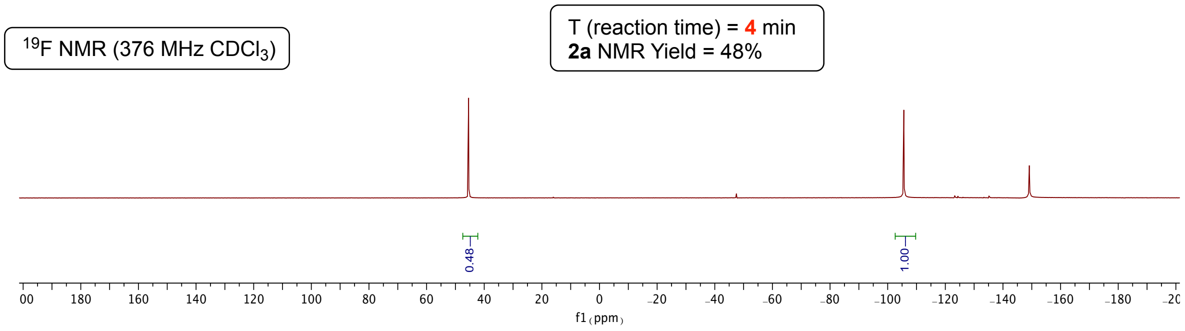

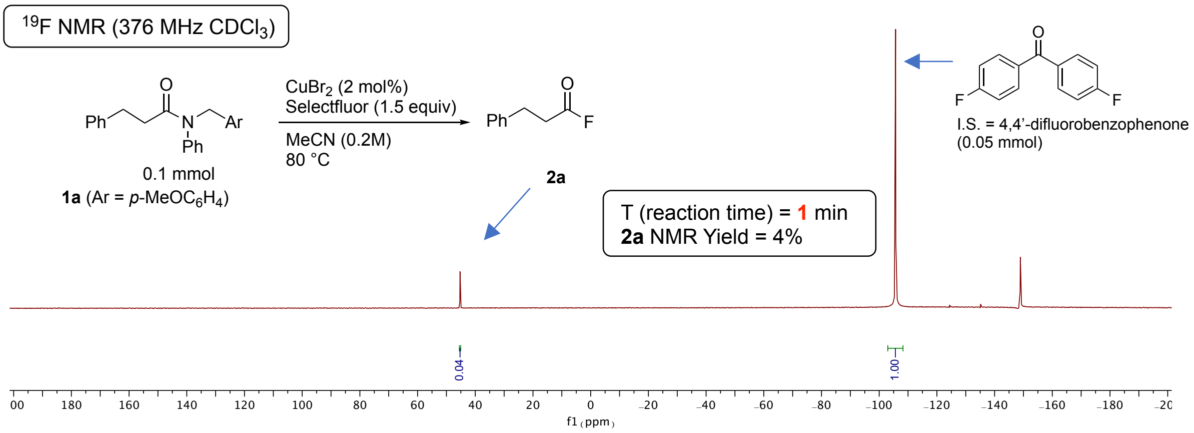
19F NMR (376 MHz CDCl3)
CuBr2 (2 mol%)
Selectfluor (1.5 equiv)
MeCN (0.2M)
80 °C
Ph
N
Ar
Ph
O
F
0.1 mmol
1a (Ar = p-MeOC6H4)
2a
I.S. = 4,4'-difluorobenzophenone
(0.05 mmol)
T (reaction time) = 1 min
2a NMR Yield = 4%
0.04
1.00
f1 (ppm)

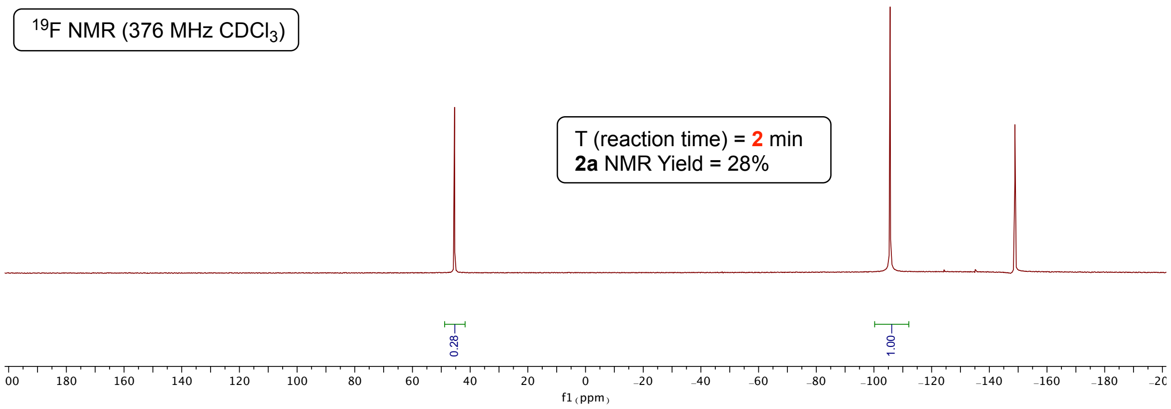
19F NMR (376 MHz CDCl3)
T (reaction time) = 2 min
2a NMR Yield = 28%
0.28
1.00
f1 (ppm)

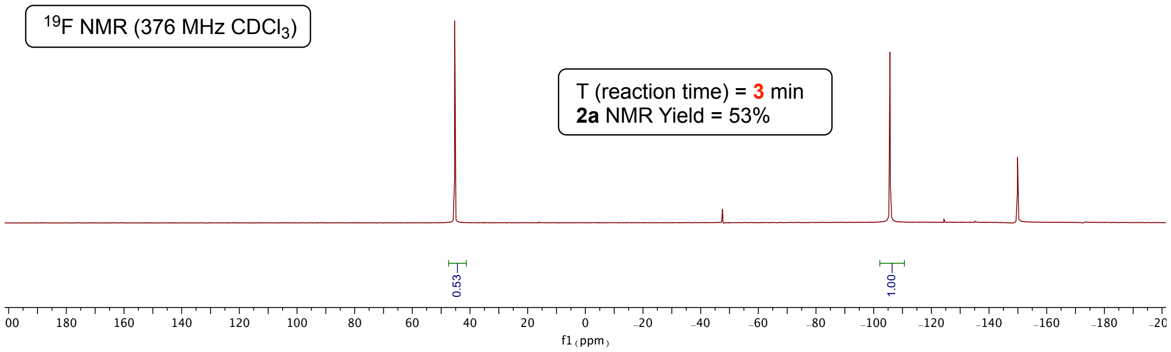
19F NMR (376 MHz CDCl3)
T (reaction time) = 3 min
2a NMR Yield = 53%
0.53
1.00
f1 (ppm)

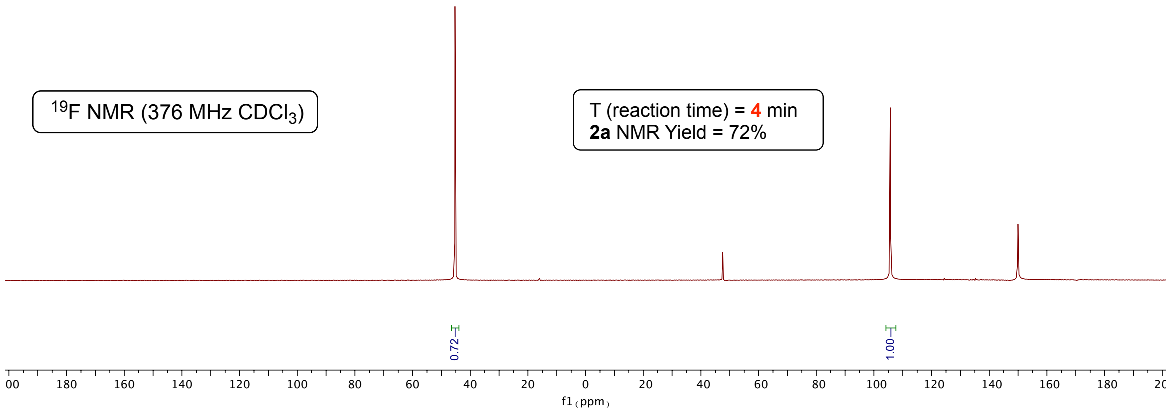
19F NMR (376 MHz CDCl3)
T (reaction time) = 4 min
2a NMR Yield = 72%
0.72
1.00
f1 (ppm)

15. References

[1] M. R. Jafari, H. Yu, J. M. Wickware, Y.-S. Lin, R. Derda, *Org. Biomol. Chem.* **2018**, *16*, 7588–7594.

[2] X. Zhao, S. Liang, X. Fan, T. Yang, W. Yu, *Org. Lett.* **2019**, *21*, 1559–1563.

[3] R. F. A. Gomes, J. A. S. Coelho, C. A. M. Afonso, *ChemSusChem* **2019**, *12*, 420–425.

[4] B. Hu, Y. Li, W. Dong, K. Ren, X. Xie, J. Wan, Z. Zhang, *Chem. Commun.* **2016**, *52*, 3709–3712.

[5] F. Sanchez-Cantalejo, J. D. Priest, P. W. Davies, *Chem. Eur. J.* **2018**, *24*, 17215–17219.

[6] M. Yudasaka, D. Shimbo, T. Maruyama, N. Tada, A. Itoh, *Org. Lett.* **2019**, *21*, 1098−1102.

[7] A. Firsov, A. Sapegin, M. Krasavin, *Eur. J. Org. Chem.* **2019**, 5242–5246.

[8] Q. W. Song, B. Yu, X.-D. Li, R. Ma, Z.-F. Diao, R.-G. Li, W. Li, L.-N. He, *Green Chem.* **2014**, *16*, 1633–1638

[9] T. Conroy, M. Manohar, Y. Gong, S. M. Wilkinson, M. Webster, B. P. Lieberman, S. D. Banister, T. A. Reekie, R. H. Mach, L. M. Rendina, M. Kassiou, *Org. Biomol. Chem.* **2016**, *14*, 9388–9405.

[10] G. Liao, X.-S. Yin, K. Chen, Q. Zhang, S.-Q. Zhang, B.-F. Shi, *Nat Commun.* **2016**, *7*, 12901.

[11] J. L. Baeza, G. Gerona-Navarro, J. Pérez de Vega, M. T. García-López, R. González-Muníz, M. Martín-Martínez, *J. Org. Chem.* **2008**, *73*, 1704–1715.

[12] T. Yoshii, S. Tsuzuki, S. Sakurai, R. Sakamoto, J. Jiang, M. Hatanaka, A. Matsumoto, K. Maruoka, *Chem. Sci.* **2020**, *11*, 5772-5778.

[13] S. Xia, Y. Song, X. Li, H. Li, L.-N. He, *Molecules*, **2018**, *23*, 3033.

[14] M. Nechab, D. N. Kumar, C. Philouze, C. Einhorn, J. Einhorn, *Angew. Chem. Int. Ed.* **2007**, *46*, 3080−3083; *Angew. Chem.* **2007**, *119*, 3140−3143.

[15] R. K. Zhang, K. Chen, X. Huang, L. Wohlschlager, H. Renata, F. H. Arnold, *Nature*, **2019**, *565*, 67–72.

[16] N. Nowrouzi, M. Z. Jonaghani, *Can. J. Chem.* **2012**, *90*, 498–509.

[17] L. Melander, W. H., Saunders, Jr. in *Reactions Rates of Isotopic Molecules*, Wiley, New York, **1980**, pp 95–102.

[18] D. A. Singleton, A. A. Thomas, *J. Am. Chem. Soc.* **1995**, *117*, 9357–9358.

16. NMR Charts

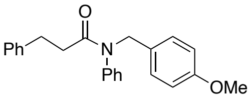

1a

^{1}H NMR ($CDCl_3$, 400 MHz)

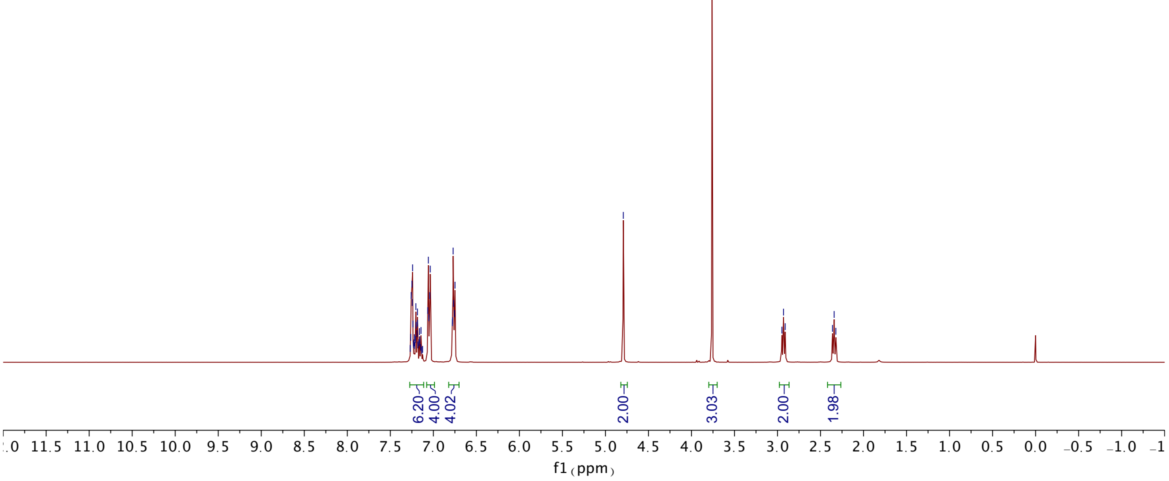

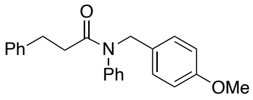

1a

^{13}C NMR ($CDCl_3$, 100 MHz)

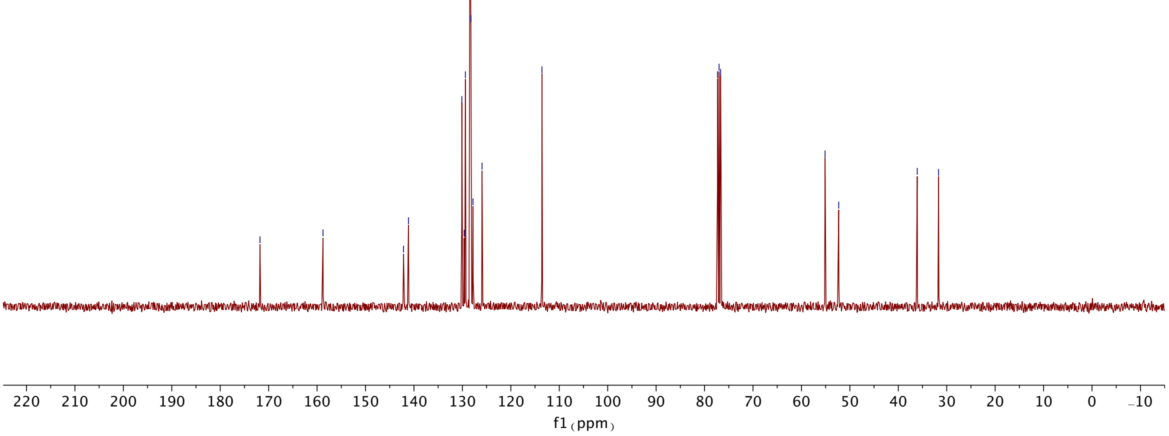

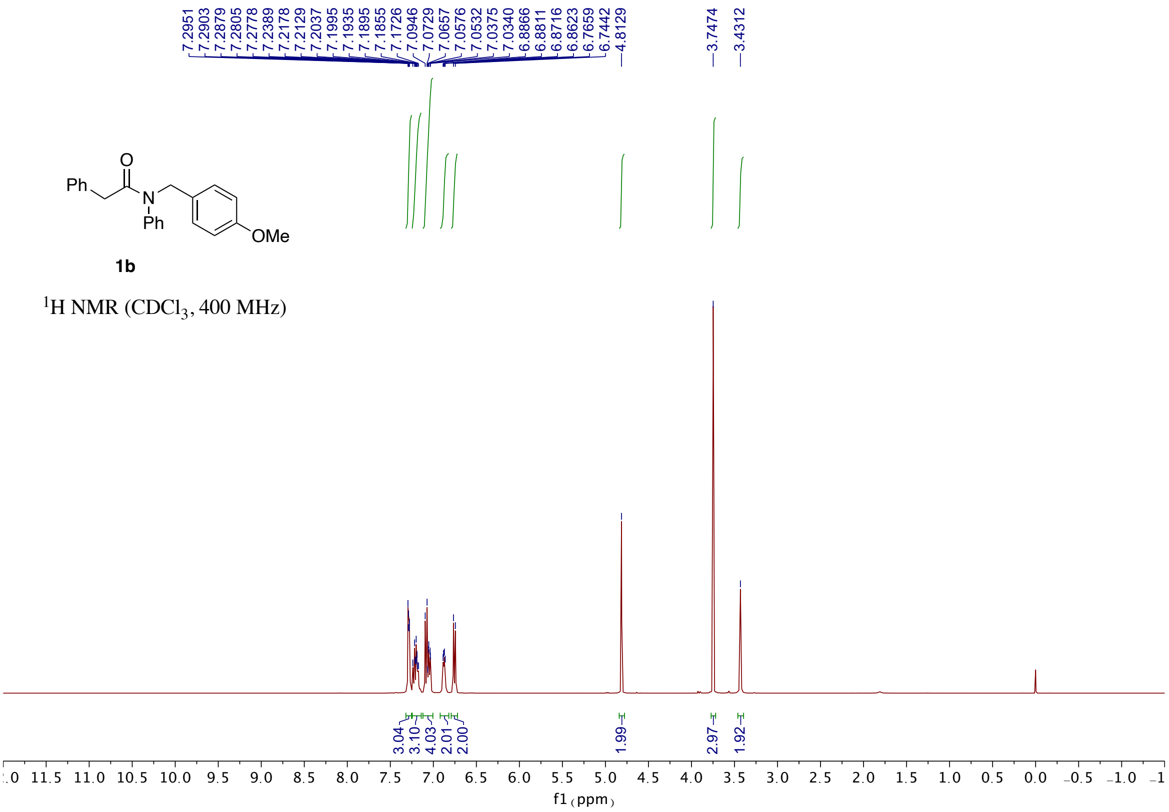

1b

^{1}H NMR ($CDCl_3$, 400 MHz)

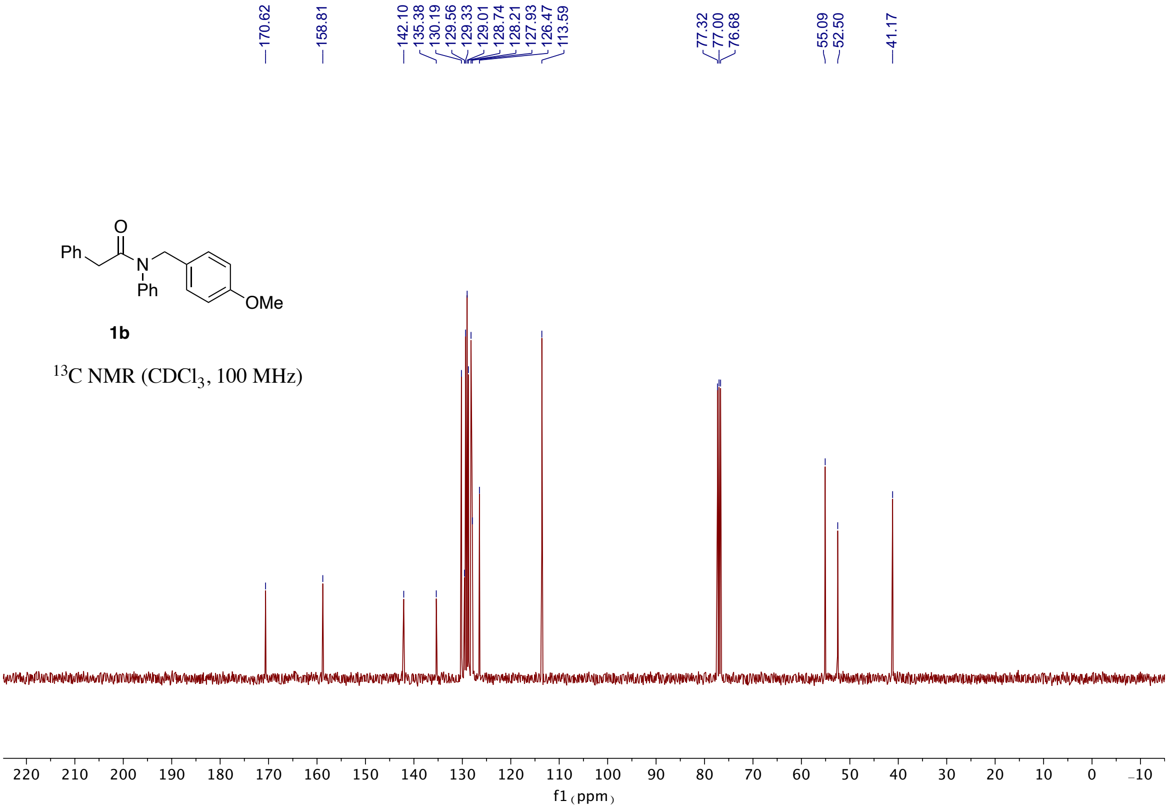

1b

^{13}C NMR ($CDCl_3$, 100 MHz)

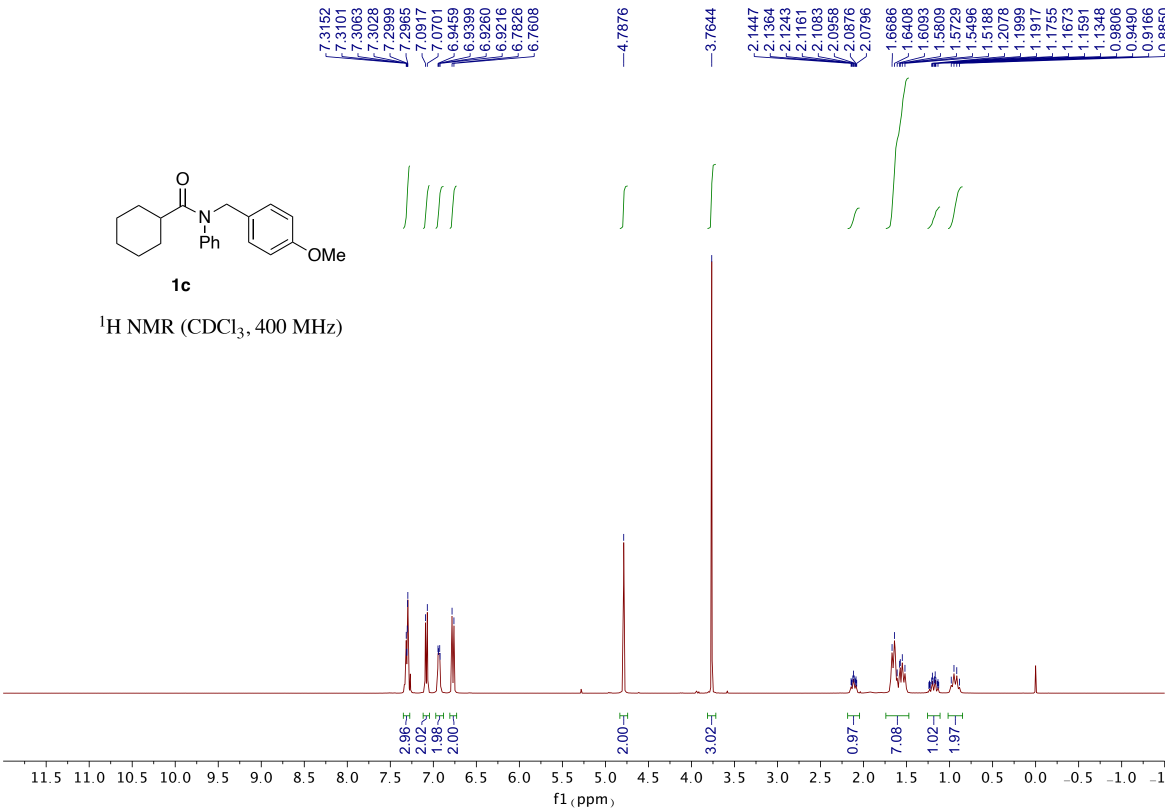
7.3152
7.3101
7.3063
7.3028
7.2999
7.2965
7.0917
7.0701
6.9459
6.9399
6.9260
6.9216
6.7826
6.7608
4.7876
3.7644
2.1447
2.1364
2.1243
2.1161
2.1083
2.0958
2.0876
2.0796
1.6686
1.6408
1.6093
1.5809
1.5729
1.5496
1.5188
1.2078
1.1999
1.1917
1.1755
1.1673
1.1591
1.1348
0.9806
0.9490
0.9166
O
N
Ph
OMe
1c
^{1}H NMR ($CDCl_3$, 400 MHz)
2.96
2.02
1.98
2.00
2.00
3.02
0.97
7.08
1.02
1.97
f1 (ppm)

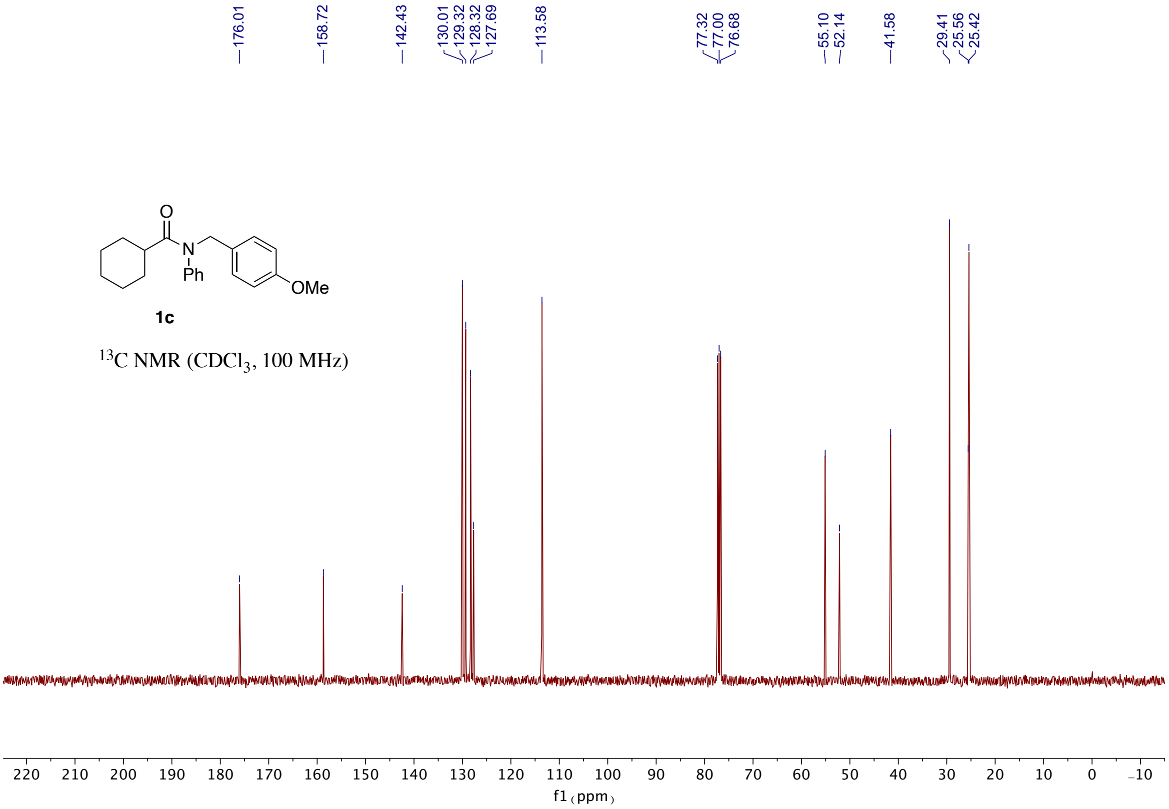
176.01
158.72
142.43
130.01
129.32
128.32
127.69
113.58
77.32
77.00
76.68
55.10
52.14
41.58
29.41
25.56
25.42
O
N
Ph
OMe
1c
^{13}C NMR ($CDCl_3$, 100 MHz)
f1 (ppm)

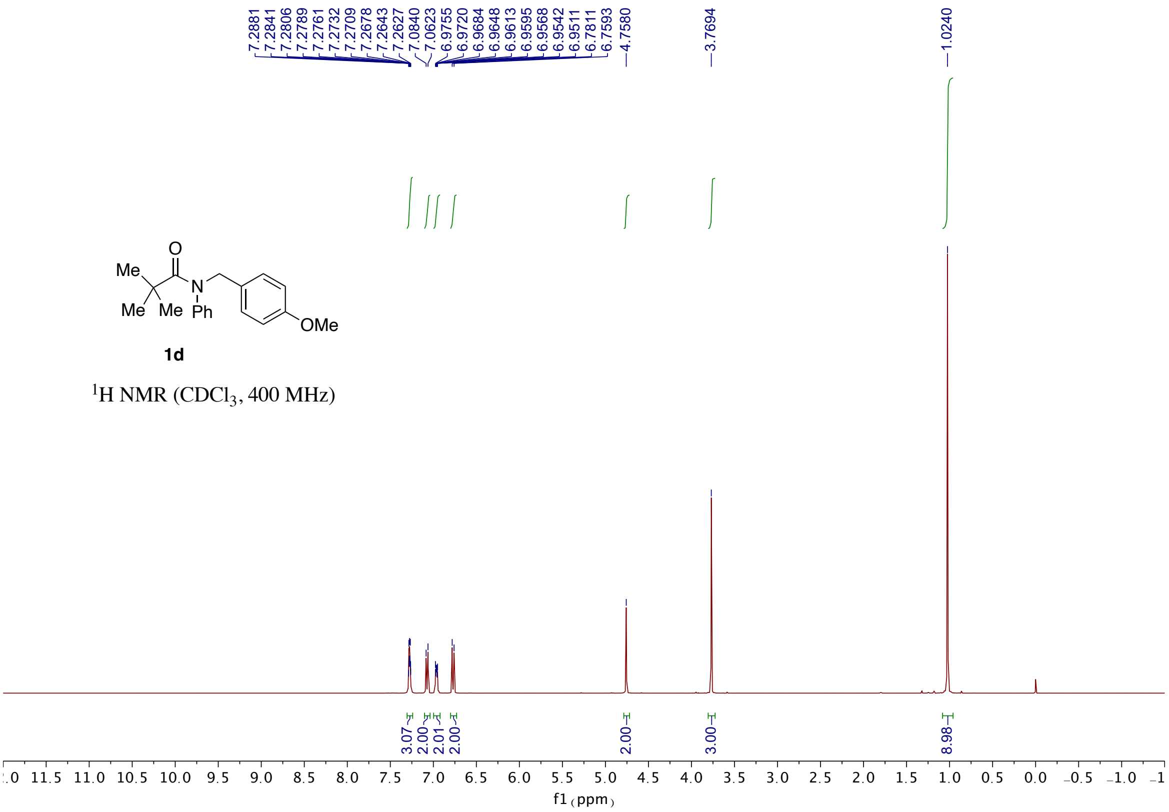
7.2881
7.2841
7.2806
7.2789
7.2761
7.2732
7.2709
7.2678
7.2643
7.2627
7.0840
7.0623
6.9755
6.9720
6.9684
6.9648
6.9613
6.9595
6.9568
6.9542
6.9511
6.7811
6.7593
4.7580
3.7694
1.0240
Me
O
N
Me
Me
Ph
OMe
1d
1H NMR (CDCl3, 400 MHz)
3.07
2.00
2.01
2.00
2.00
3.00
8.98
f1 (ppm)

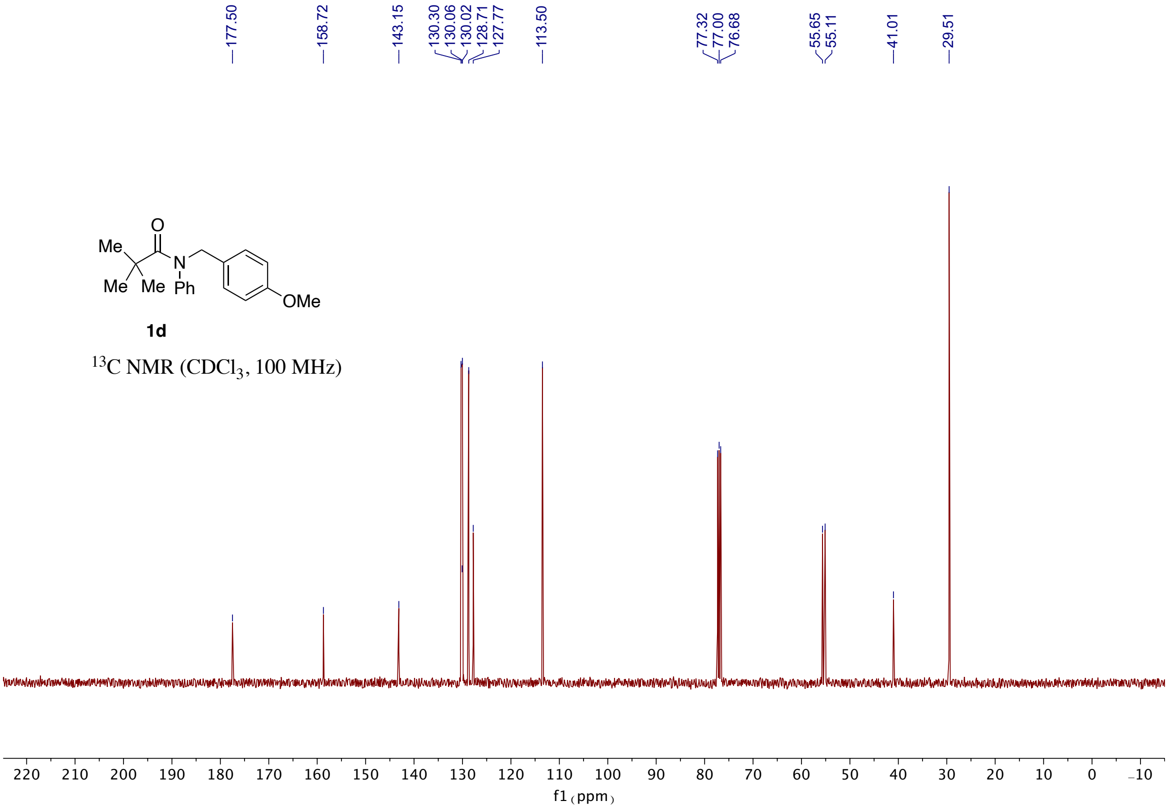
177.50
158.72
143.15
130.30
130.06
130.02
128.71
127.77
113.50
77.32
77.00
76.68
55.65
55.11
41.01
29.51
Me
O
N
Me
Me
Ph
OMe
1d
13C NMR (CDCl3, 100 MHz)
f1 (ppm)

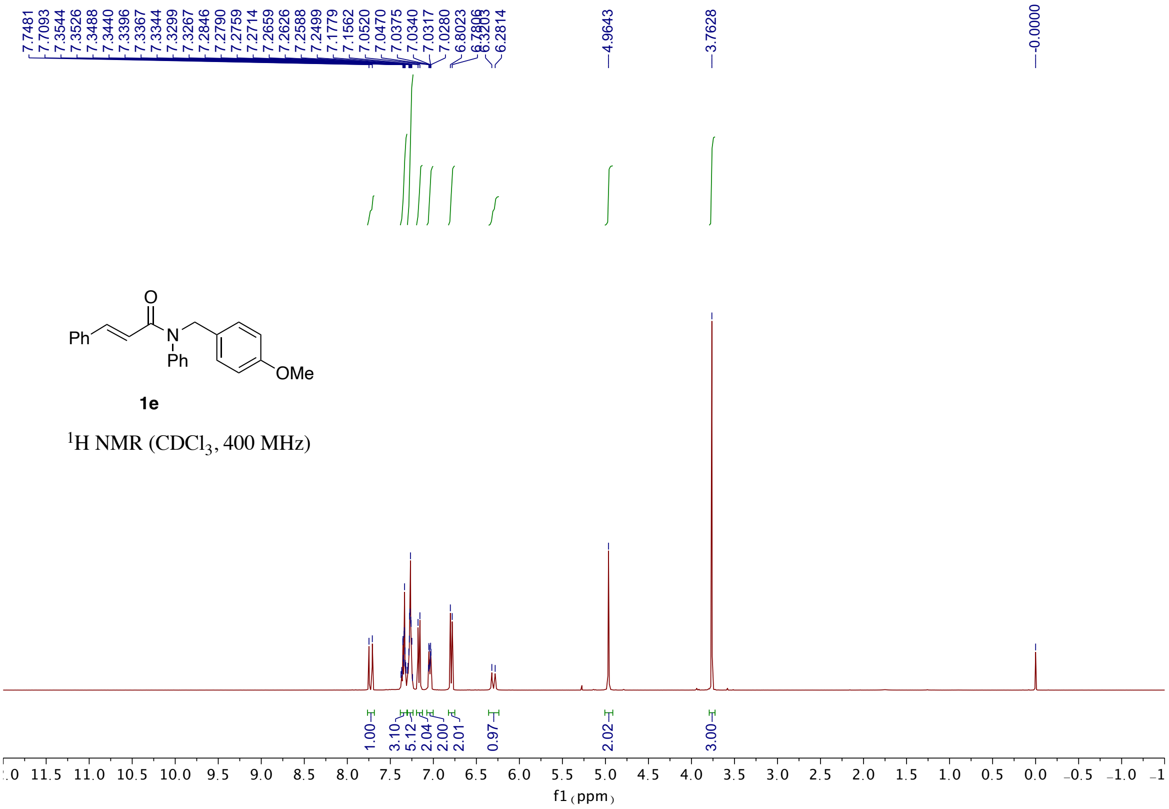
7.7481 7.7093 7.3544 7.3526 7.3488 7.3440 7.3396 7.3367 7.3344 7.3299 7.3267 7.2846 7.2790 7.2759 7.2714 7.2659 7.2626 7.2588 7.2499 7.1779 7.1562 7.0520 7.0470 7.0375 7.0340 7.0317 7.0280 6.8023 6.2814
4.9643
3.7628
-0.0000
O
Ph
N
Ph
OMe
1e
^{1}H NMR ($CDCl_3$, 400 MHz)
1.00 3.10 5.12 2.04 2.00 2.01 0.97 2.02 3.00
f1 (ppm)

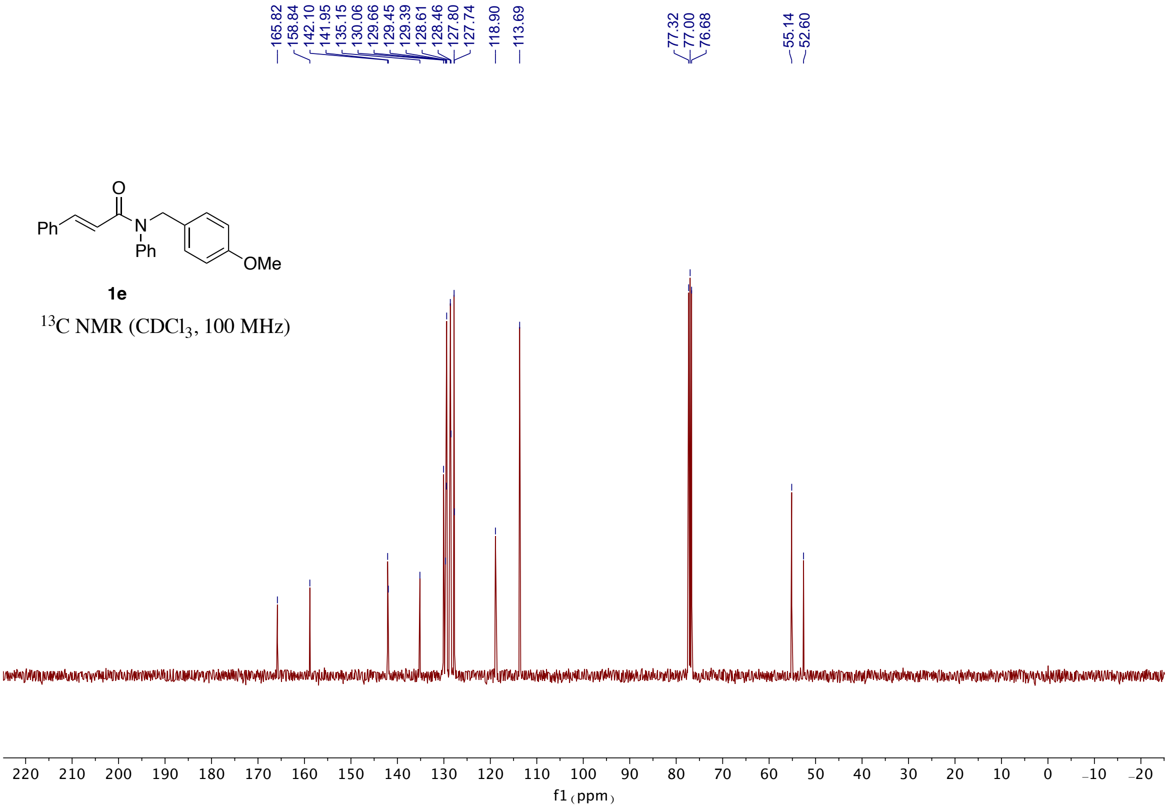
165.82 158.84 142.10 141.95 135.15 130.06 129.66 129.45 129.39 128.61 128.46 127.80 127.74 118.90 113.69
77.32 77.00 76.68
55.14 52.60
O
Ph
N
Ph
OMe
1e
^{13}C NMR ($CDCl_3$, 100 MHz)
f1 (ppm)

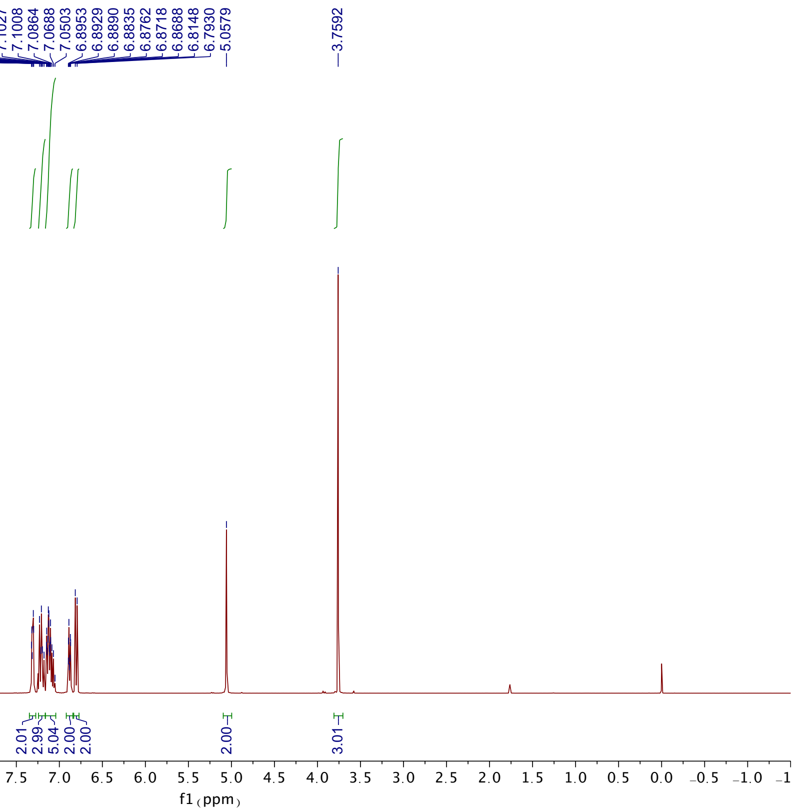

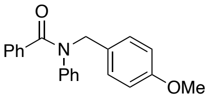

1f

^{1}H NMR (CDCl$_3$, 400 MHz)

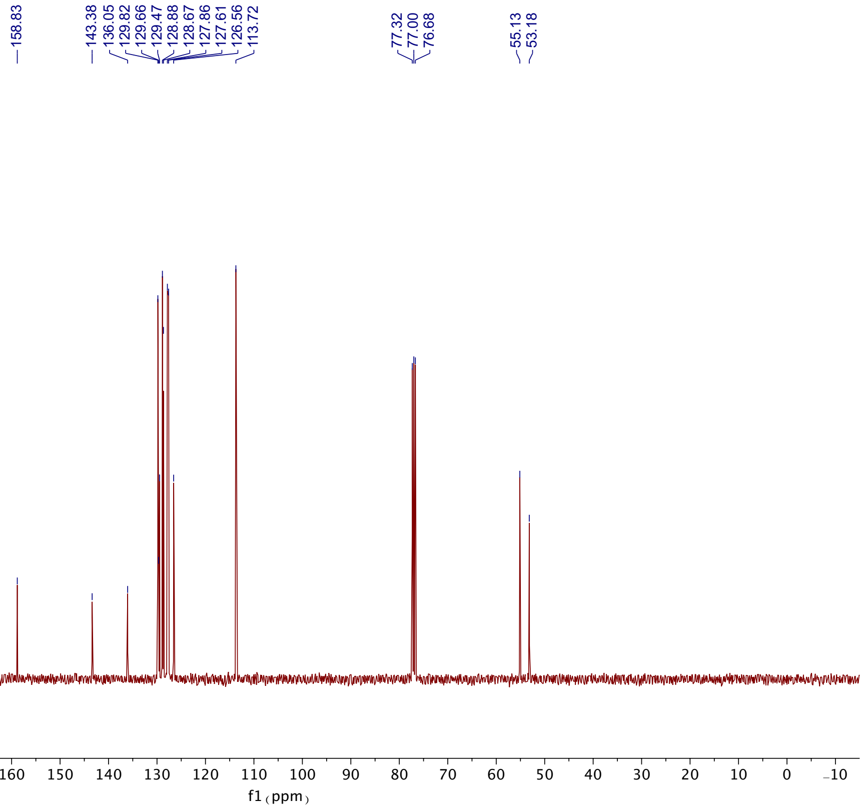

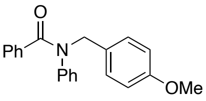

1f

^{13}C NMR (CDCl$_3$, 100 MHz)

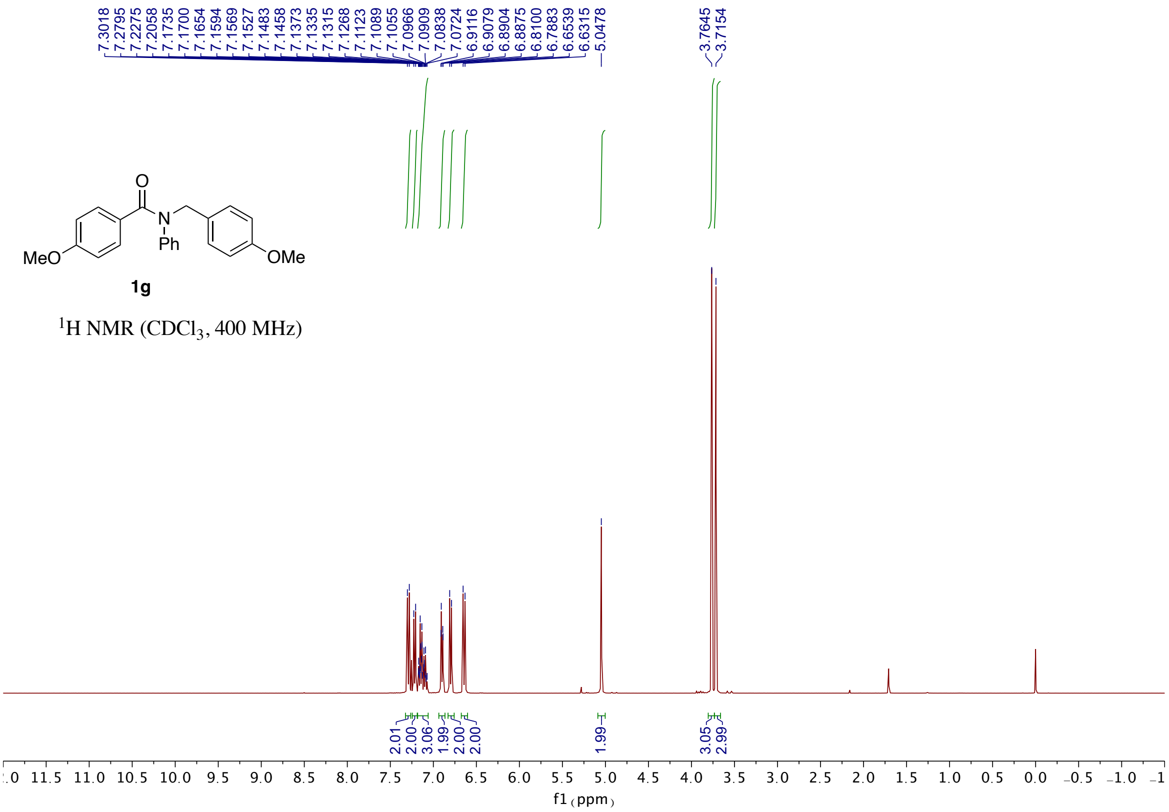

1g

^{1}H NMR ($CDCl_3$, 400 MHz)

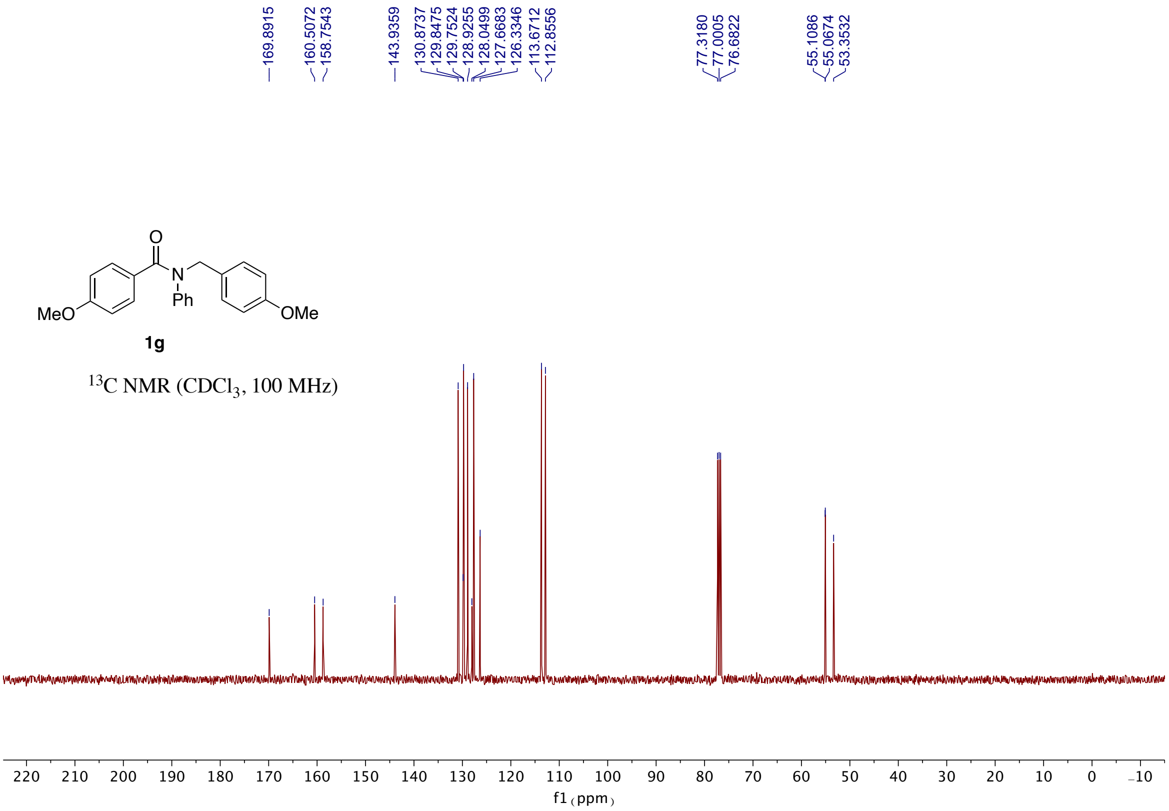

1g

^{13}C NMR ($CDCl_3$, 100 MHz)

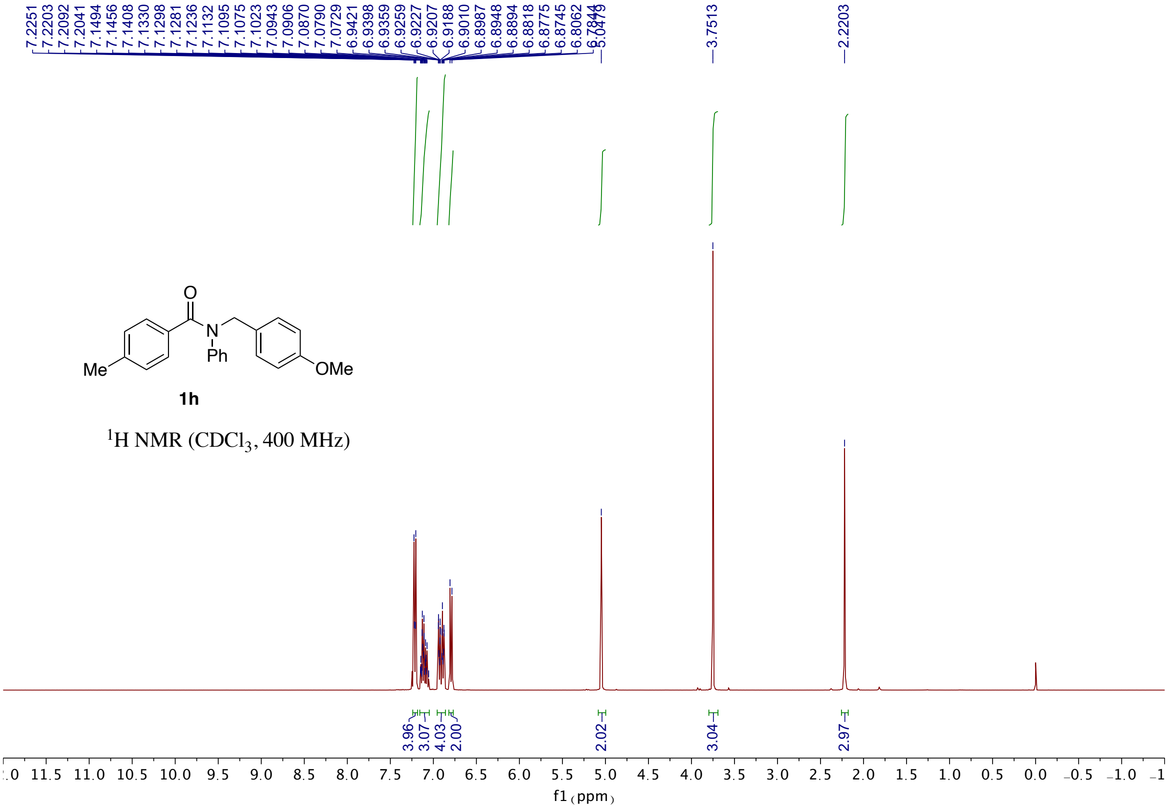
7.2251
7.2203
7.2092
7.2041
7.1494
7.1456
7.1408
7.1330
7.1298
7.1281
7.1236
7.1132
7.1095
7.1075
7.1023
7.0943
7.0906
7.0870
7.0790
7.0729
6.9421
6.9398
6.9359
6.9259
6.9227
6.9207
6.9188
6.9010
6.8987
6.8948
6.8894
6.8818
6.8775
6.8745
6.8062
6.7844
5.0479
3.7513
2.2203
O
N
Ph
Me
OMe
1h
^{1}H NMR ($CDCl_3$, 400 MHz)
3.96
3.07
4.03
2.00
2.02
3.04
2.97
f1 (ppm)

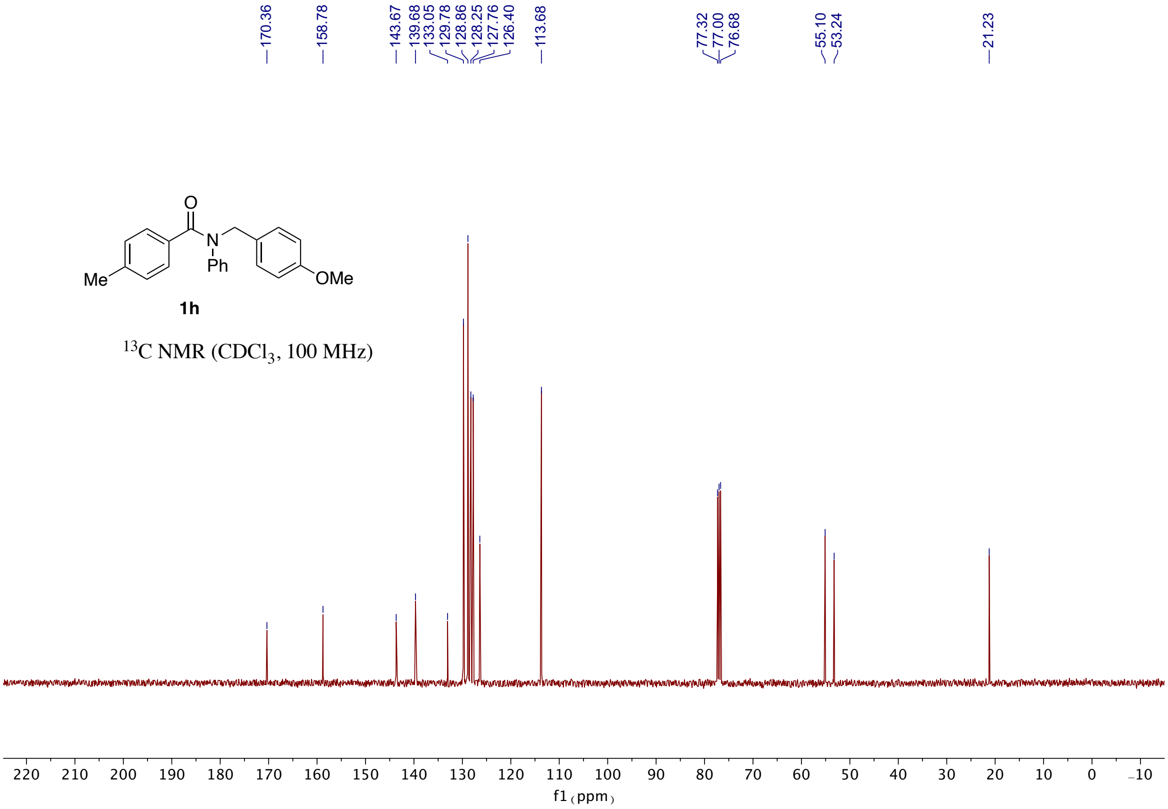
170.36
158.78
143.67
139.68
133.05
129.78
128.86
128.25
127.76
126.40
113.68
77.32
77.00
76.68
55.10
53.24
21.23
O
N
Ph
Me
OMe
1h
^{13}C NMR ($CDCl_3$, 100 MHz)
f1 (ppm)

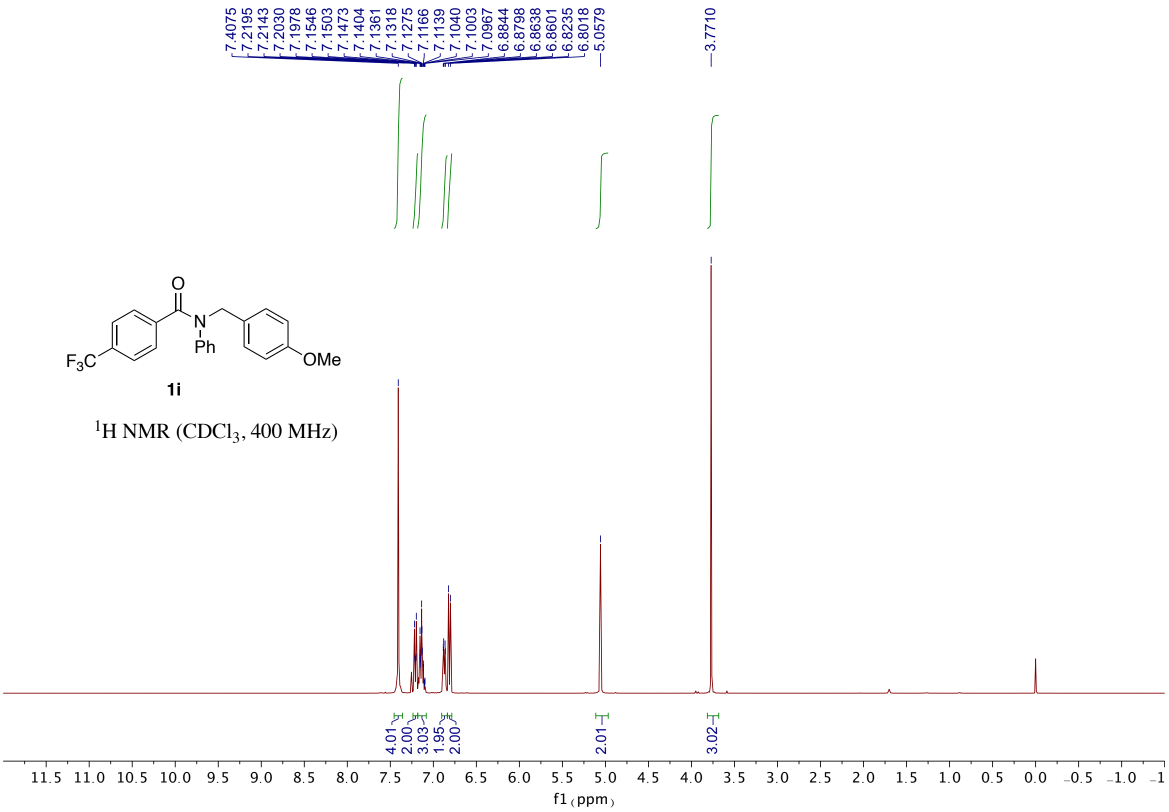

1i

^{1}H NMR ($CDCl_3$, 400 MHz)

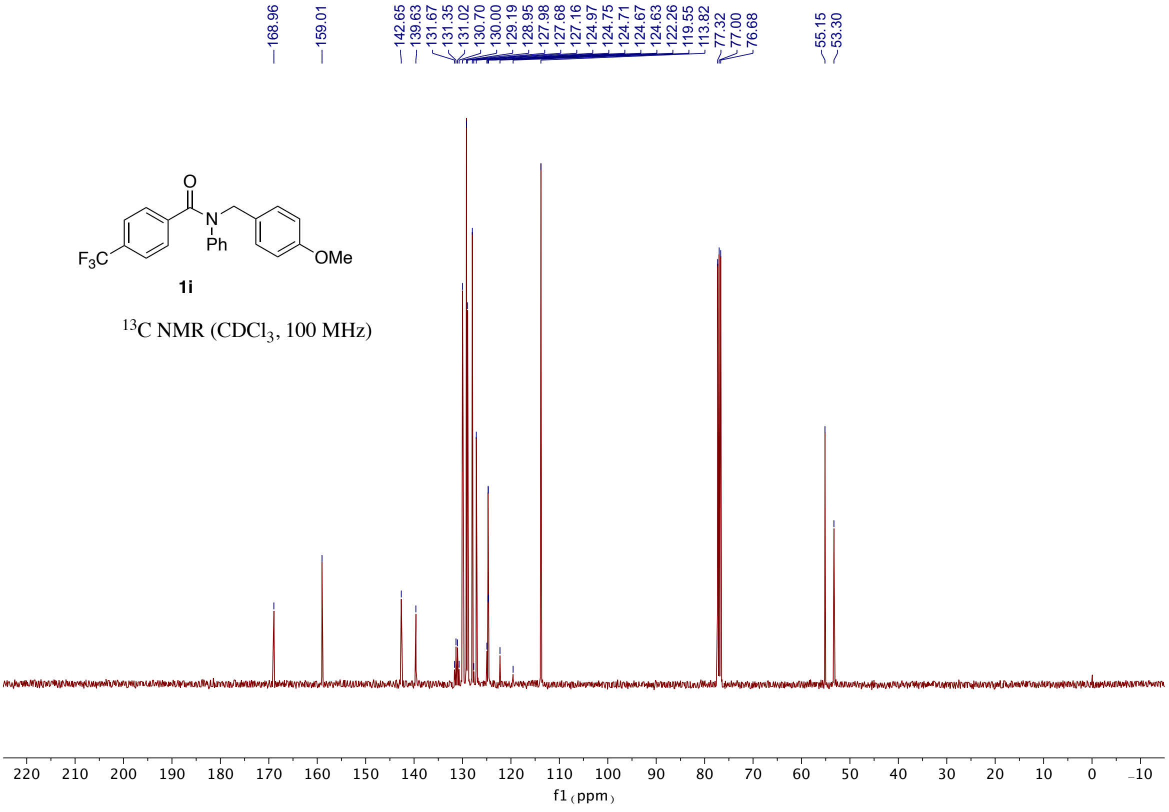

1i

^{13}C NMR ($CDCl_3$, 100 MHz)

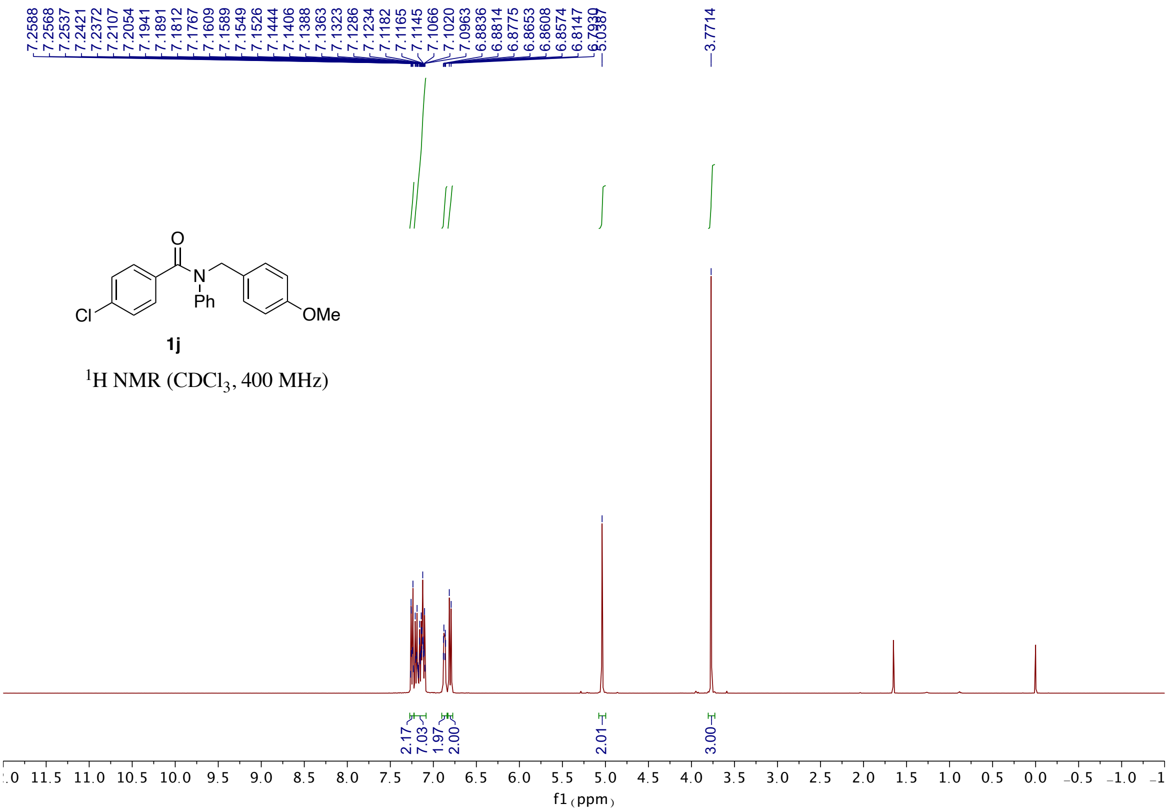

1j

^{1}H NMR (CDCl$_3$, 400 MHz)

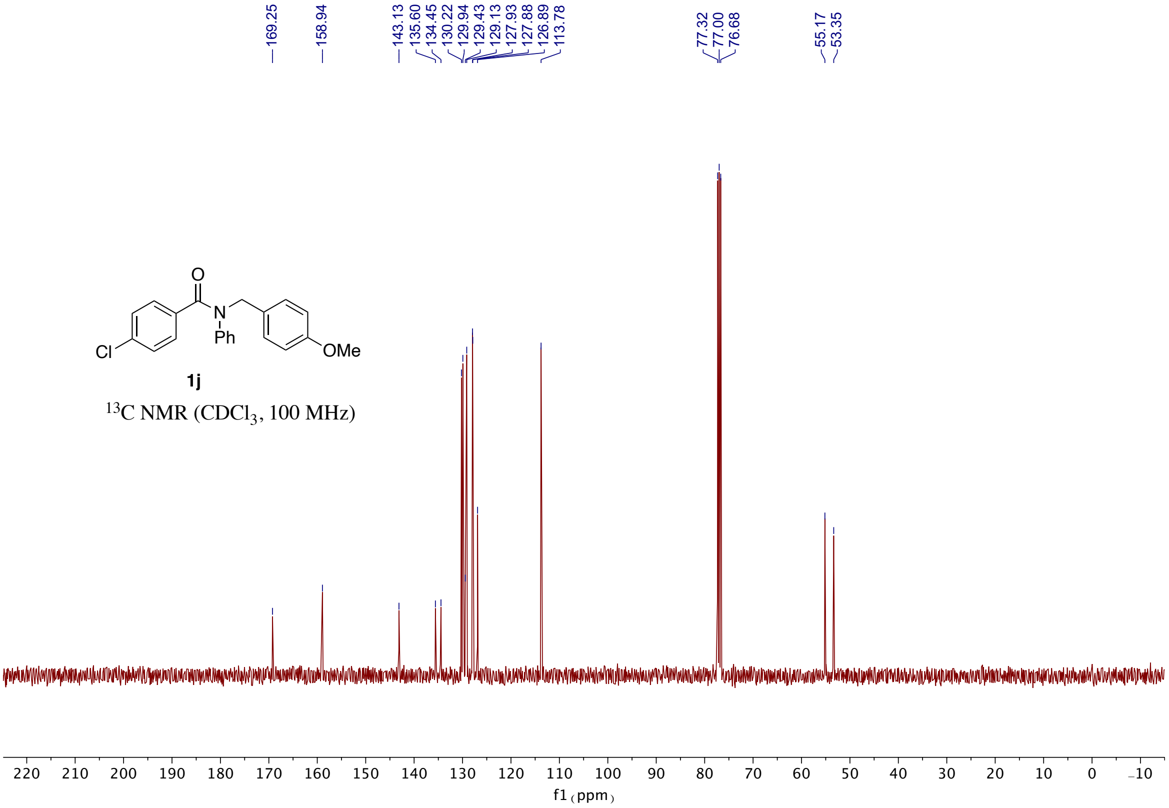

1j

^{13}C NMR (CDCl$_3$, 100 MHz)

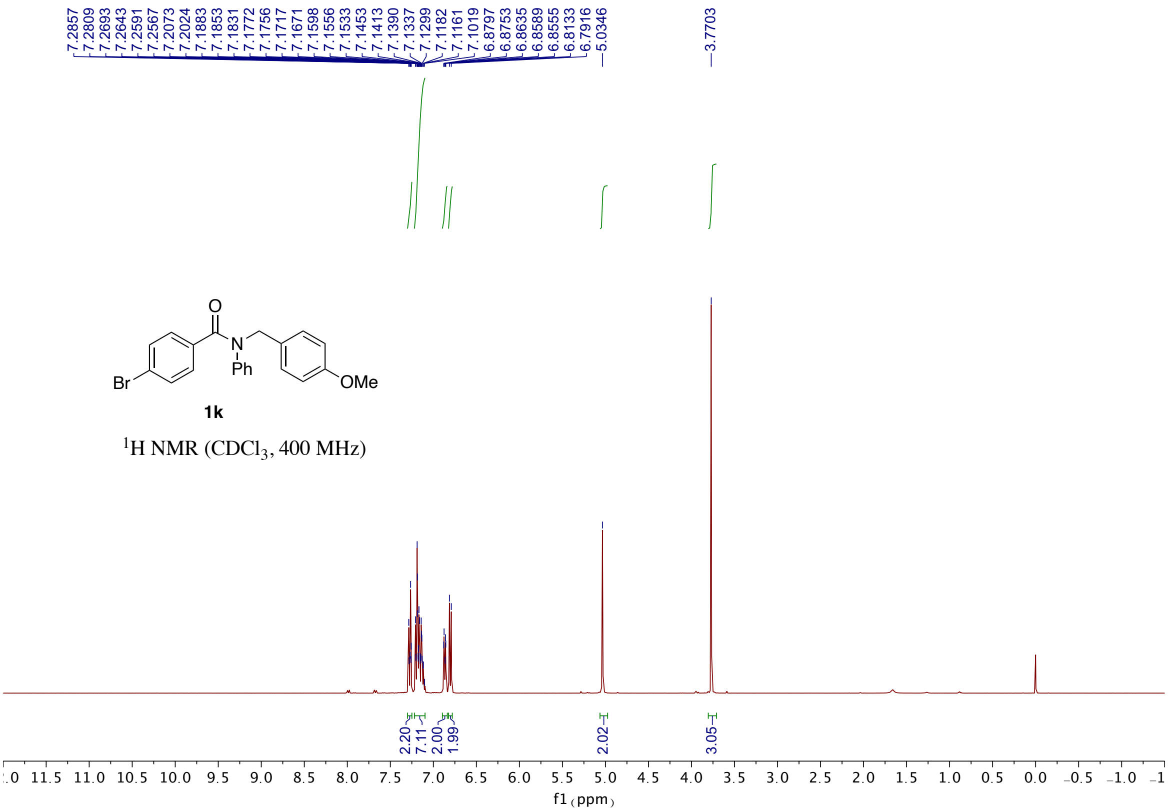
7.2857 7.2809 7.2693 7.2643 7.2591 7.2567 7.2073 7.2024 7.1883 7.1853 7.1831 7.1772 7.1756 7.1717 7.1671 7.1598 7.1556 7.1533 7.1453 7.1413 7.1390 7.1337 7.1299 7.1182 7.1161 7.1019 6.8797 6.8753 6.8635 6.8589 6.8555 6.8133 6.7916 5.0346
3.7703
O
N
Ph
Br
OMe
1k
1H NMR (CDCl3, 400 MHz)
2.20 7.11 2.00 1.99
2.02
3.05
f1 (ppm)

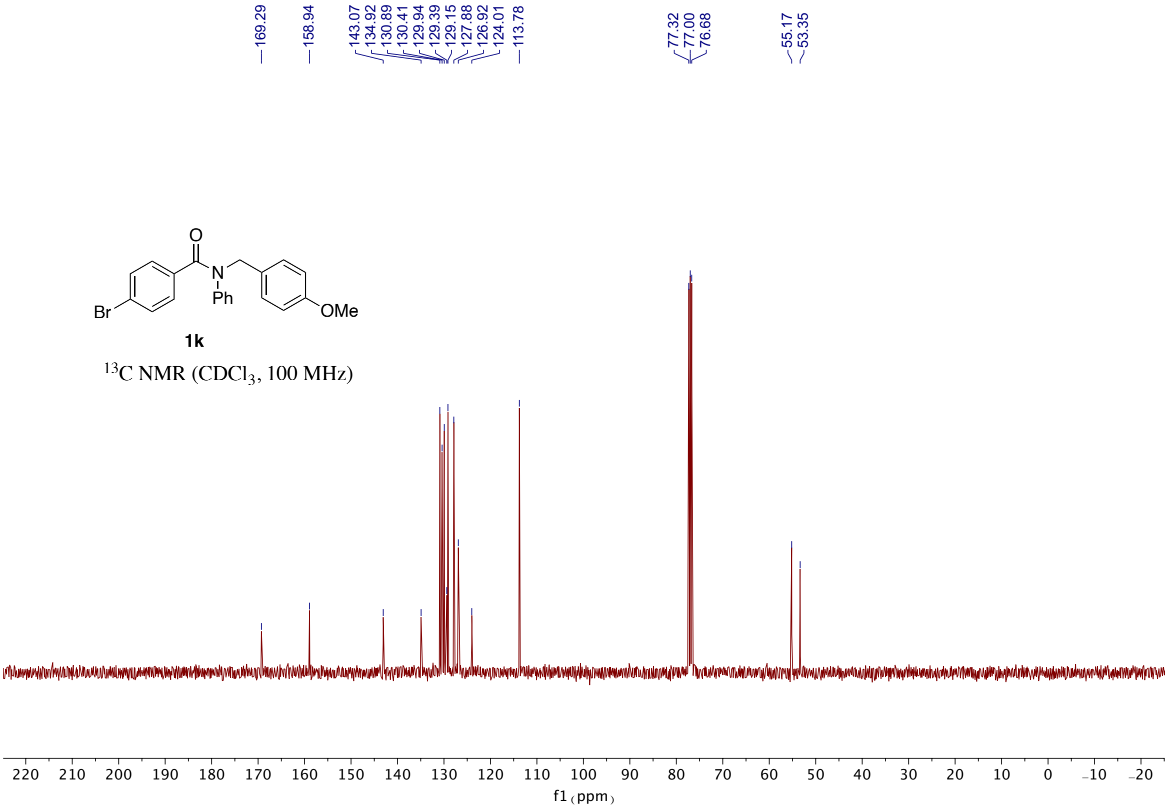
169.29
158.94
143.07 134.92 130.89 130.41 129.94 129.39 129.15 127.88 126.92 124.01
113.78
77.32 77.00 76.68
55.17 53.35
O
N
Ph
Br
OMe
1k
13C NMR (CDCl3, 100 MHz)
f1 (ppm)

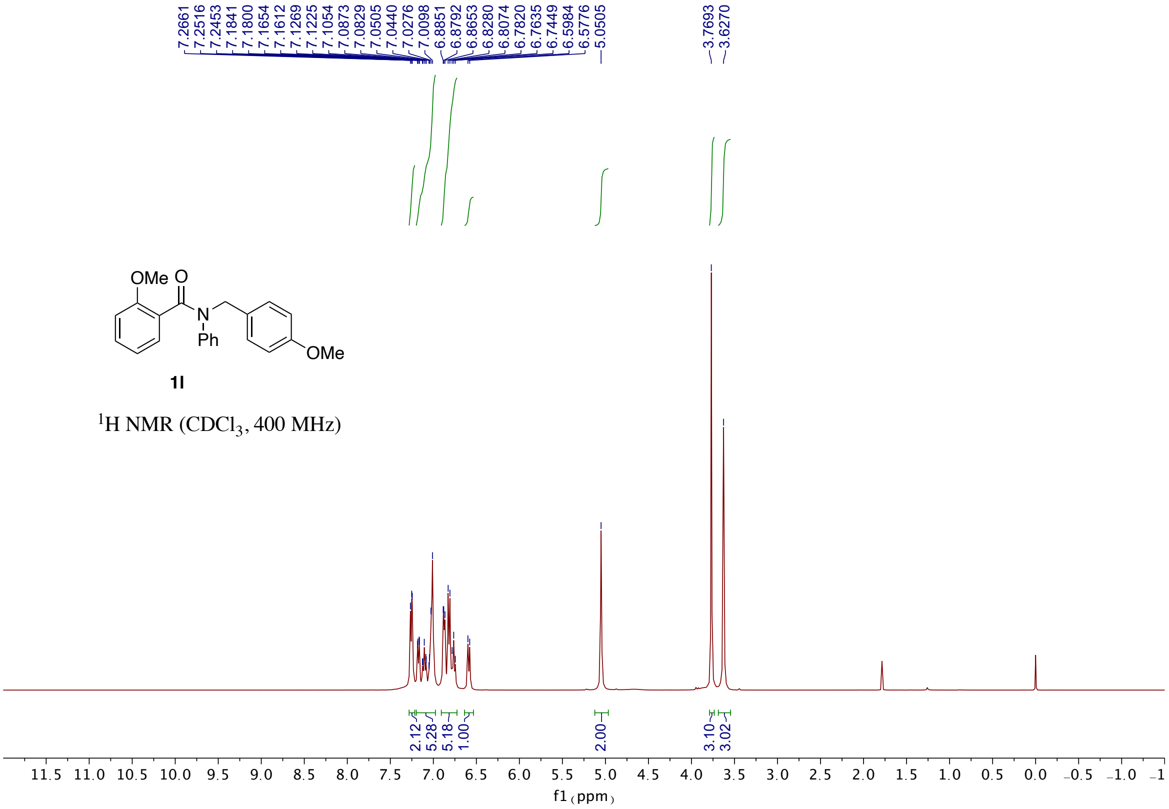

1l

^{1}H NMR ($CDCl_3$, 400 MHz)

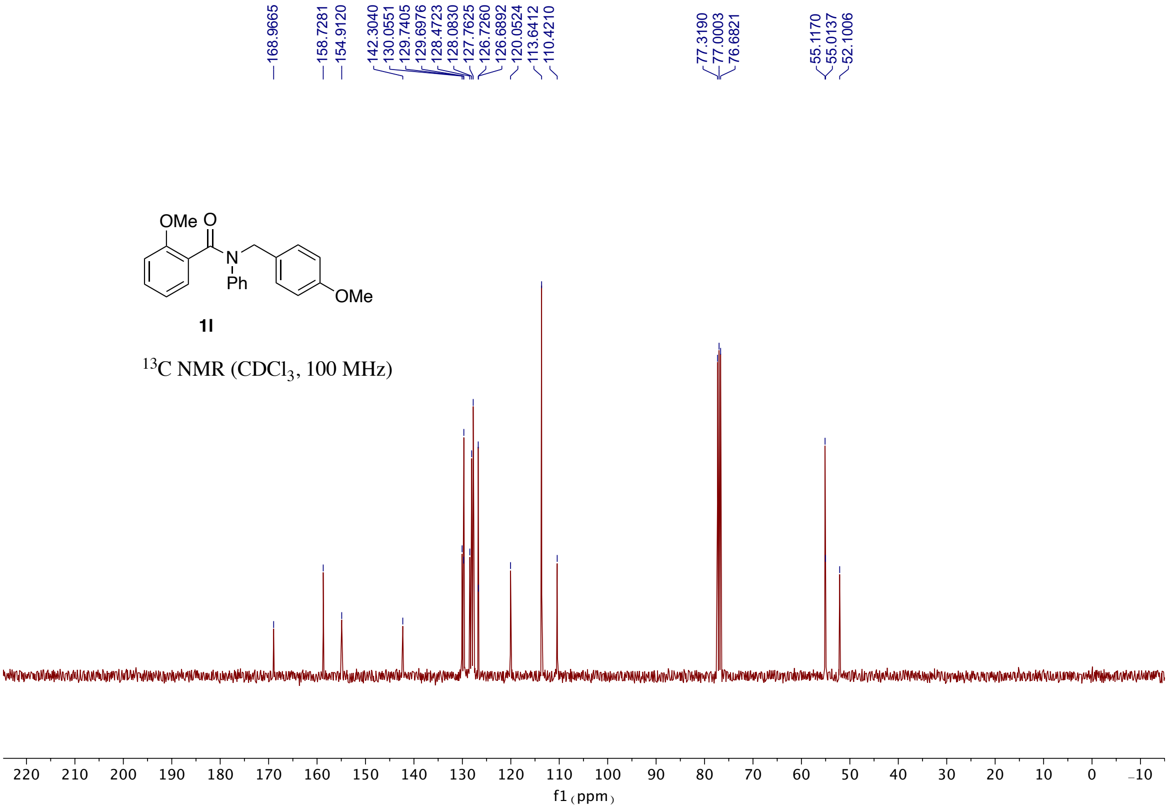

1l

^{13}C NMR ($CDCl_3$, 100 MHz)

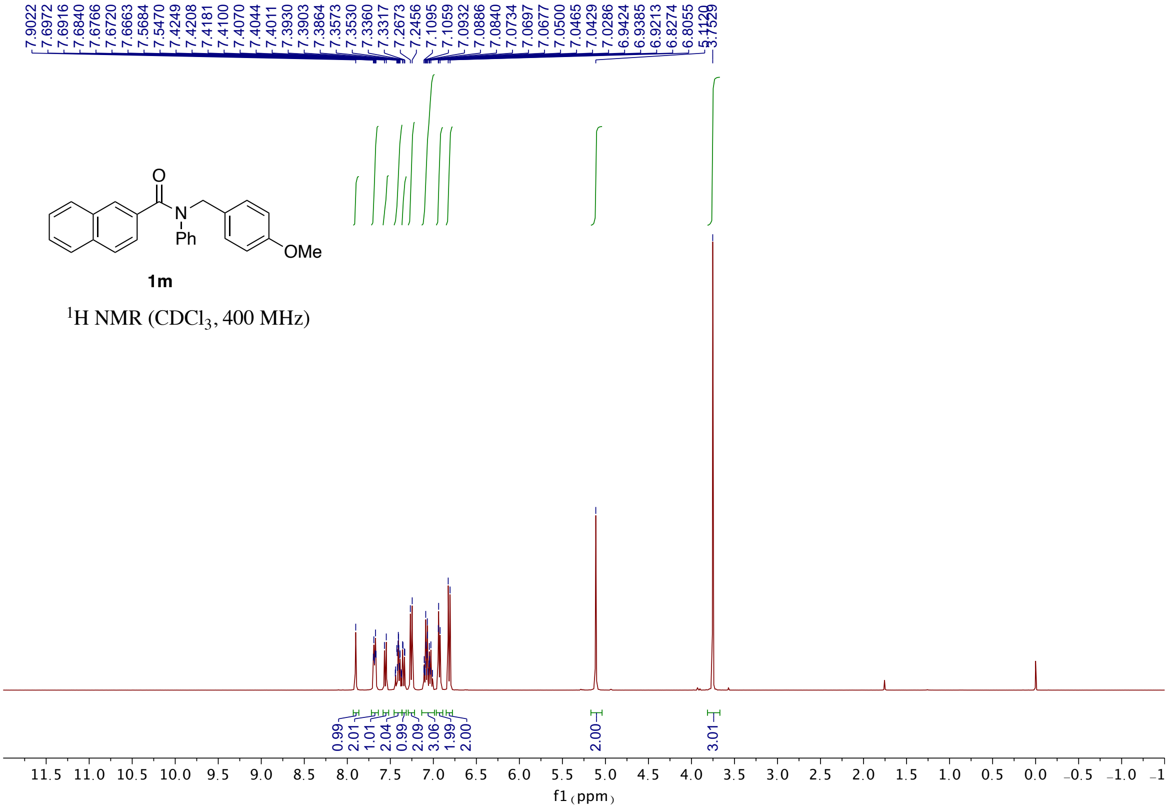

1m

^{1}H NMR ($CDCl_3$, 400 MHz)

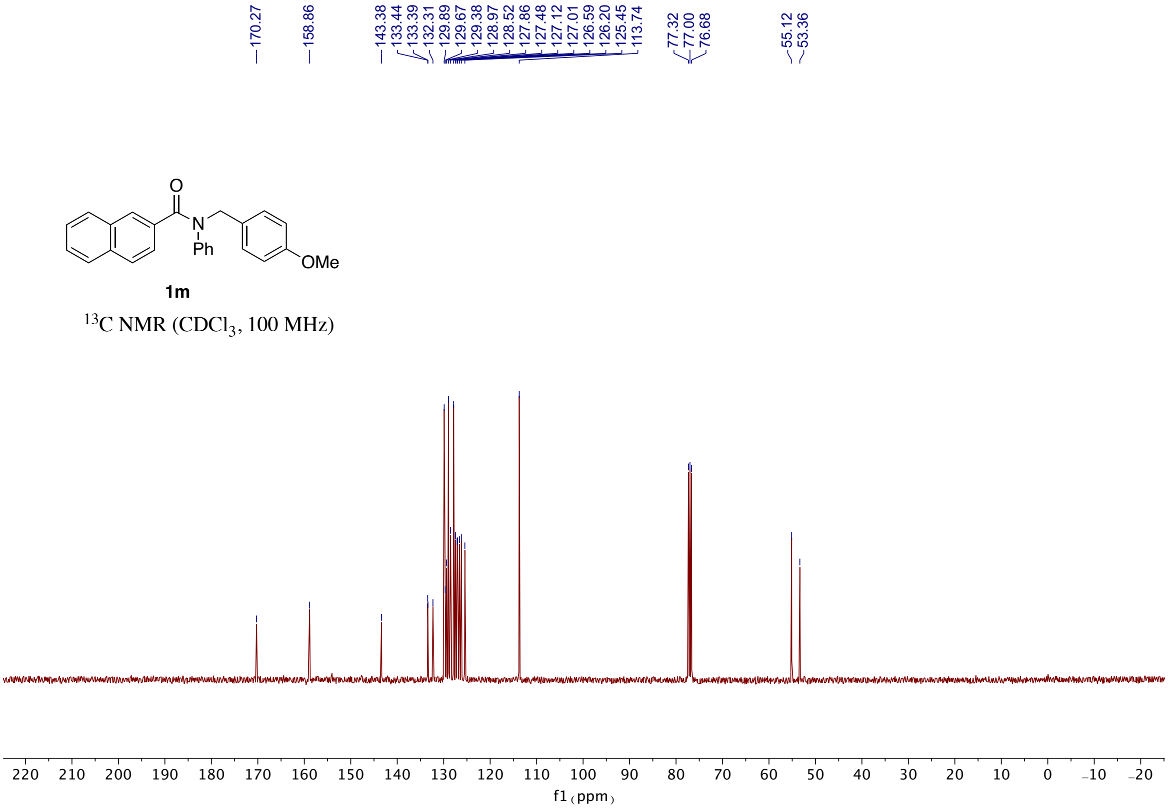

1m

^{13}C NMR ($CDCl_3$, 100 MHz)

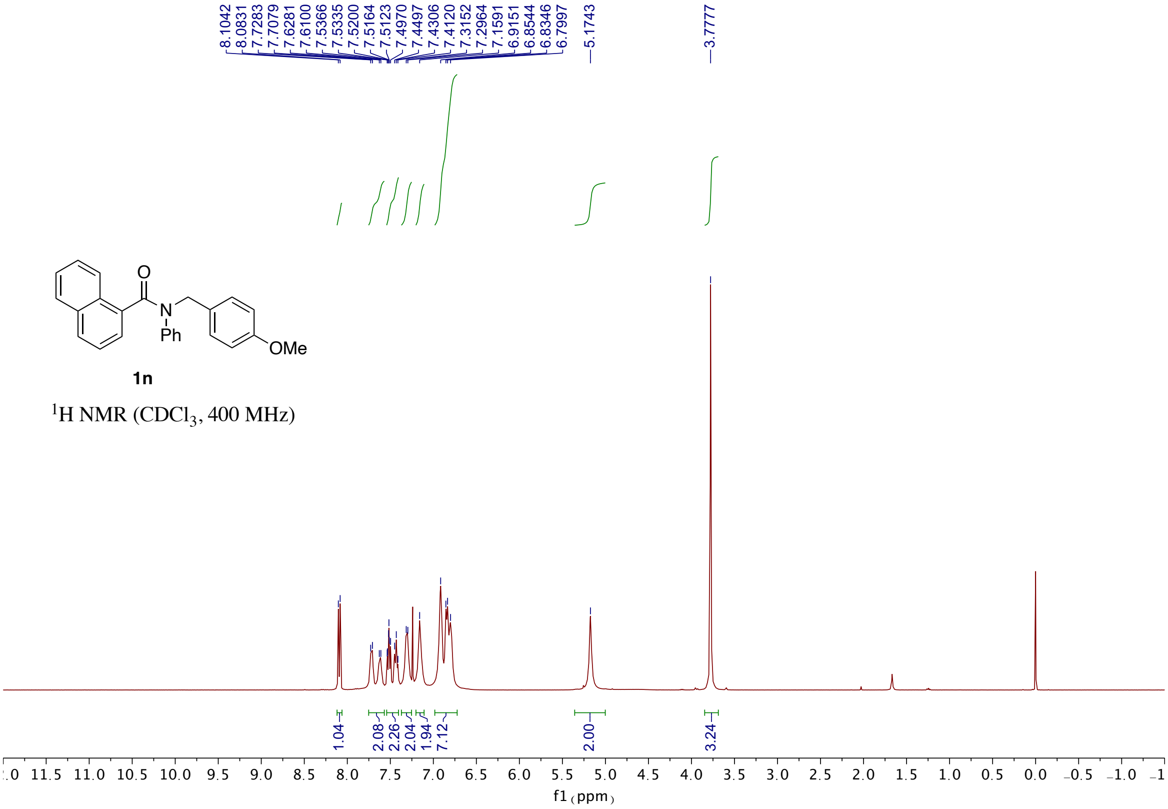

1n

^{1}H NMR ($CDCl_3$, 400 MHz)

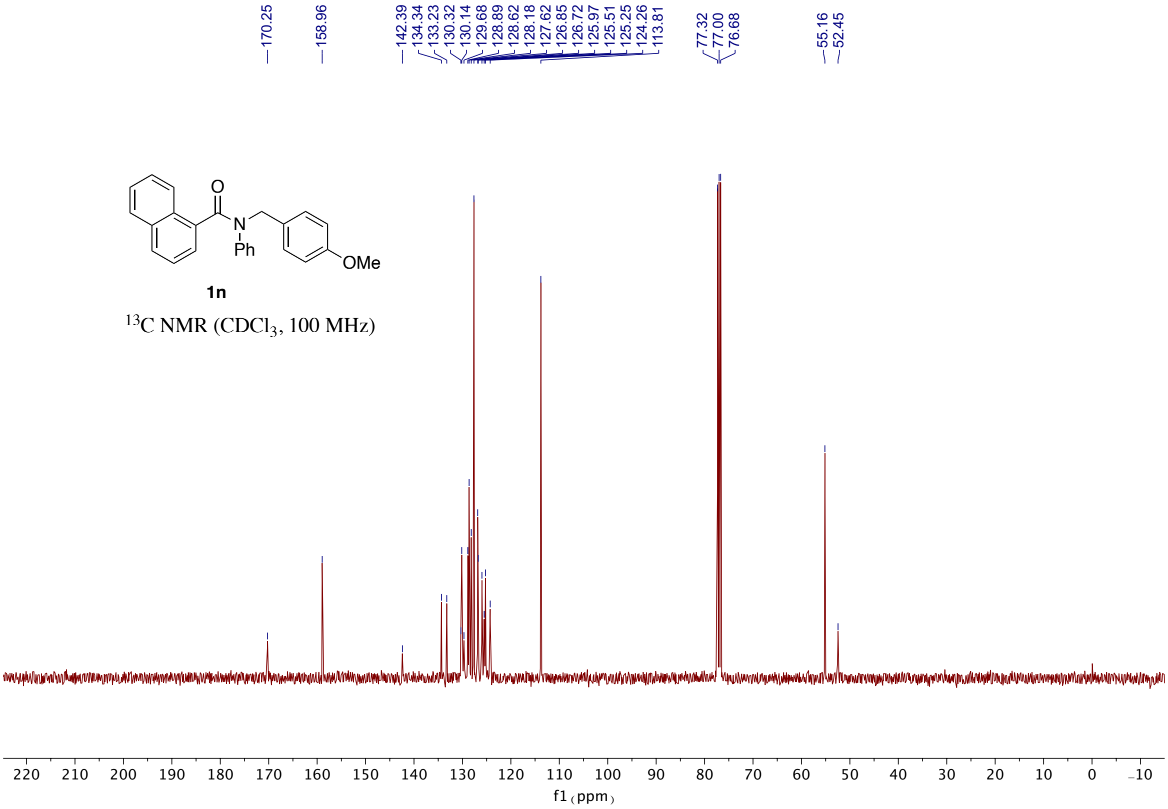

1n

^{13}C NMR ($CDCl_3$, 100 MHz)

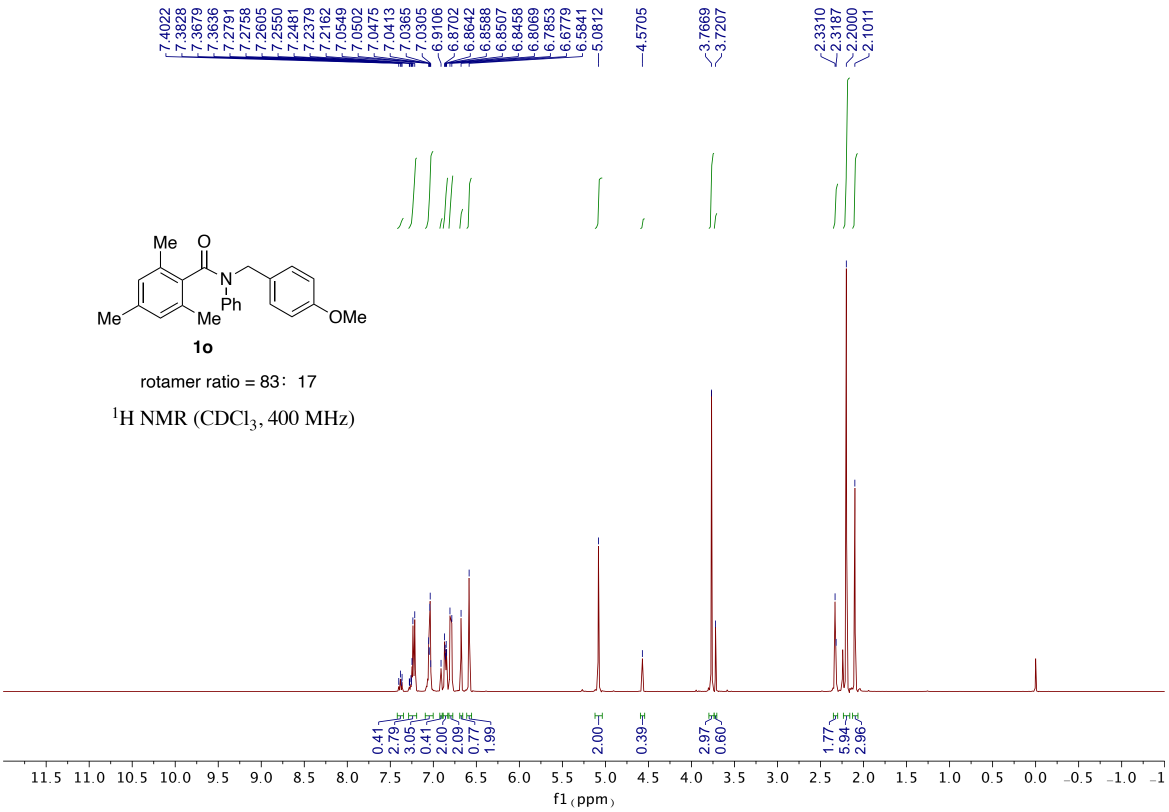
7.4022
7.3828
7.3679
7.3636
7.2791
7.2758
7.2605
7.2550
7.2481
7.2379
7.2162
7.0549
7.0502
7.0475
7.0413
7.0365
7.0305
6.9106
6.8702
6.8642
6.8588
6.8507
6.8458
6.8069
6.7853
6.6779
6.5841
5.0812
4.5705
3.7669
3.7207
2.3310
2.3187
2.2000
2.1011
Me
O
N
Ph
Me
Me
OMe
1o
rotamer ratio = 83： 17
^{1}H NMR (CDCl$_3$, 400 MHz)
0.41
2.79
3.05
0.41
2.00
2.09
0.77
1.99
2.00
0.39
2.97
0.60
1.77
5.94
2.96
f1 (ppm)

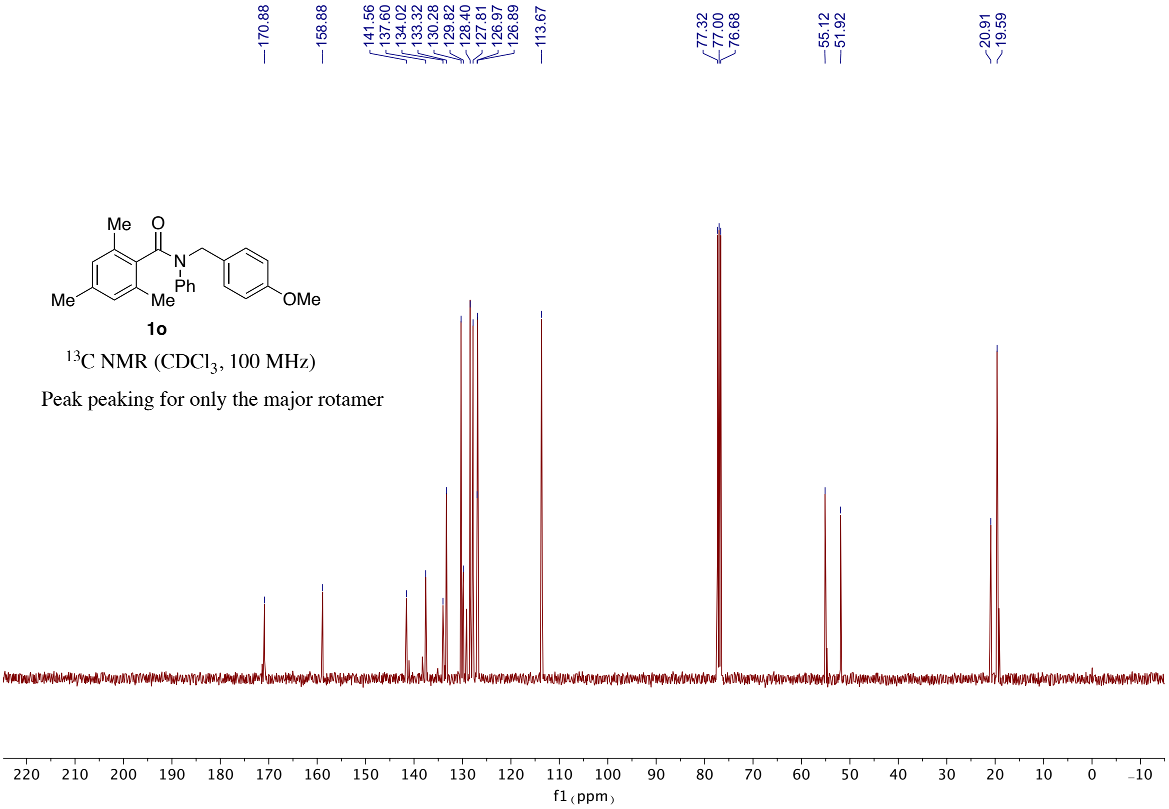
170.88
158.88
141.56
137.60
134.02
133.32
130.28
129.82
128.40
127.81
126.97
126.89
113.67
77.32
77.00
76.68
55.12
51.92
20.91
19.59
Me
O
N
Ph
Me
Me
OMe
1o
^{13}C NMR (CDCl$_3$, 100 MHz)
Peak peaking for only the major rotamer
f1 (ppm)

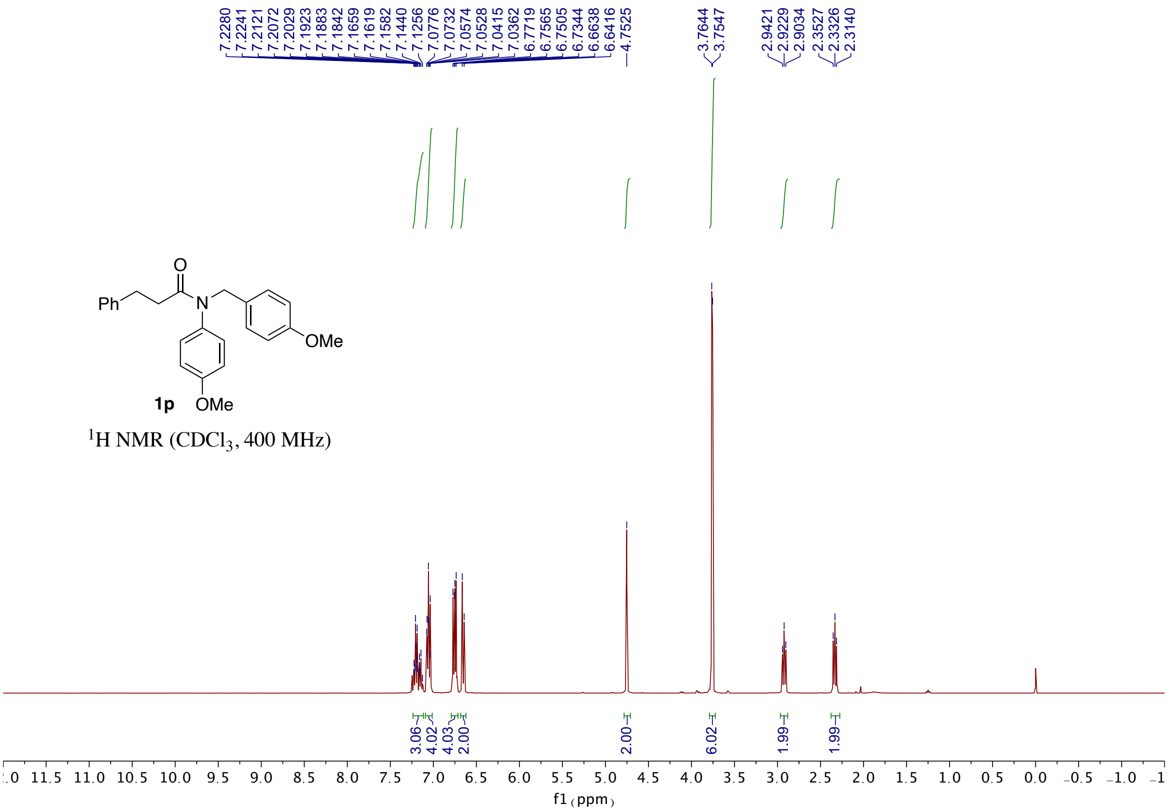
7.2280
7.2241
7.2121
7.2072
7.2029
7.1923
7.1883
7.1842
7.1659
7.1619
7.1582
7.1440
7.1256
7.0776
7.0732
7.0574
7.0528
7.0415
7.0362
6.7719
6.7565
6.7505
6.7344
6.6638
6.6416
4.7525
3.7644
3.7547
2.9421
2.9229
2.9034
2.3527
2.3326
2.3140
Ph
O
N
OMe
1p
OMe
^{1}H NMR ($CDCl_3$, 400 MHz)
3.06
4.02
4.03
2.00
2.00
6.02
1.99
1.99
f1 (ppm)

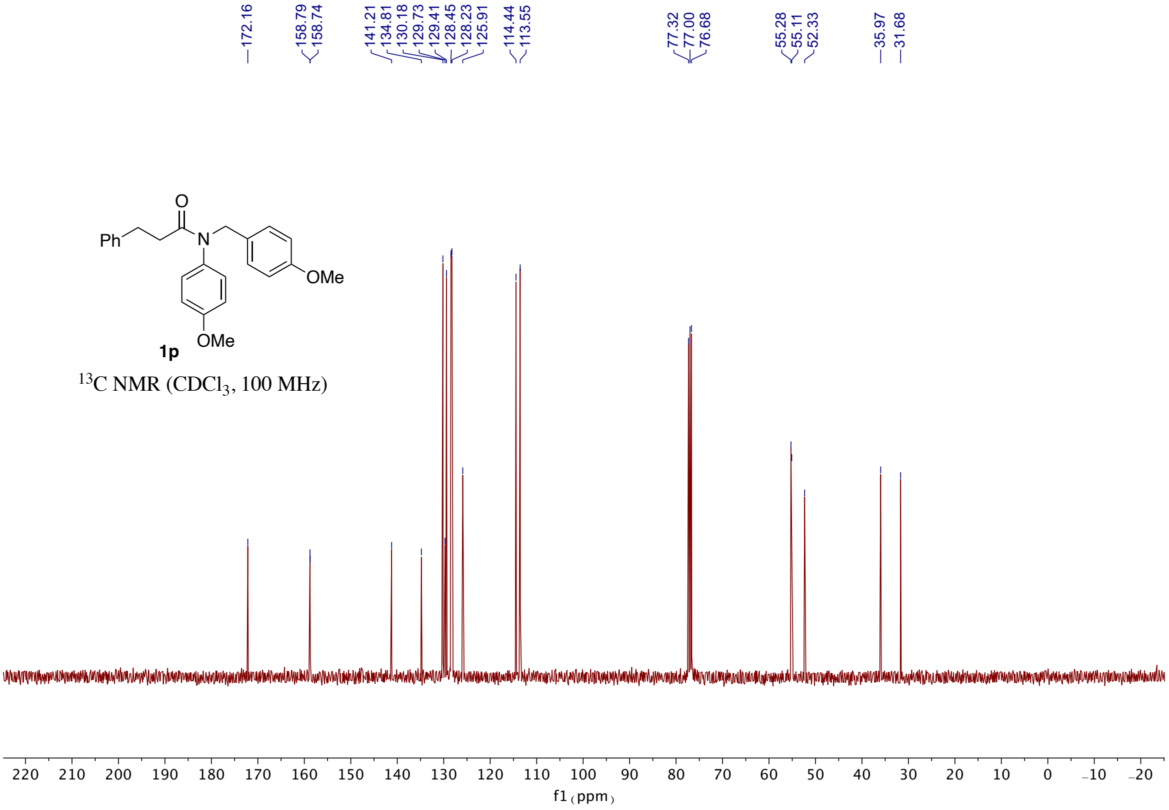
172.16
158.79
158.74
141.21
134.81
130.18
129.73
129.41
128.45
128.23
125.91
114.44
113.55
77.32
77.00
76.68
55.28
55.11
52.33
35.97
31.68
Ph
O
N
OMe
1p
OMe
^{13}C NMR ($CDCl_3$, 100 MHz)
f1 (ppm)

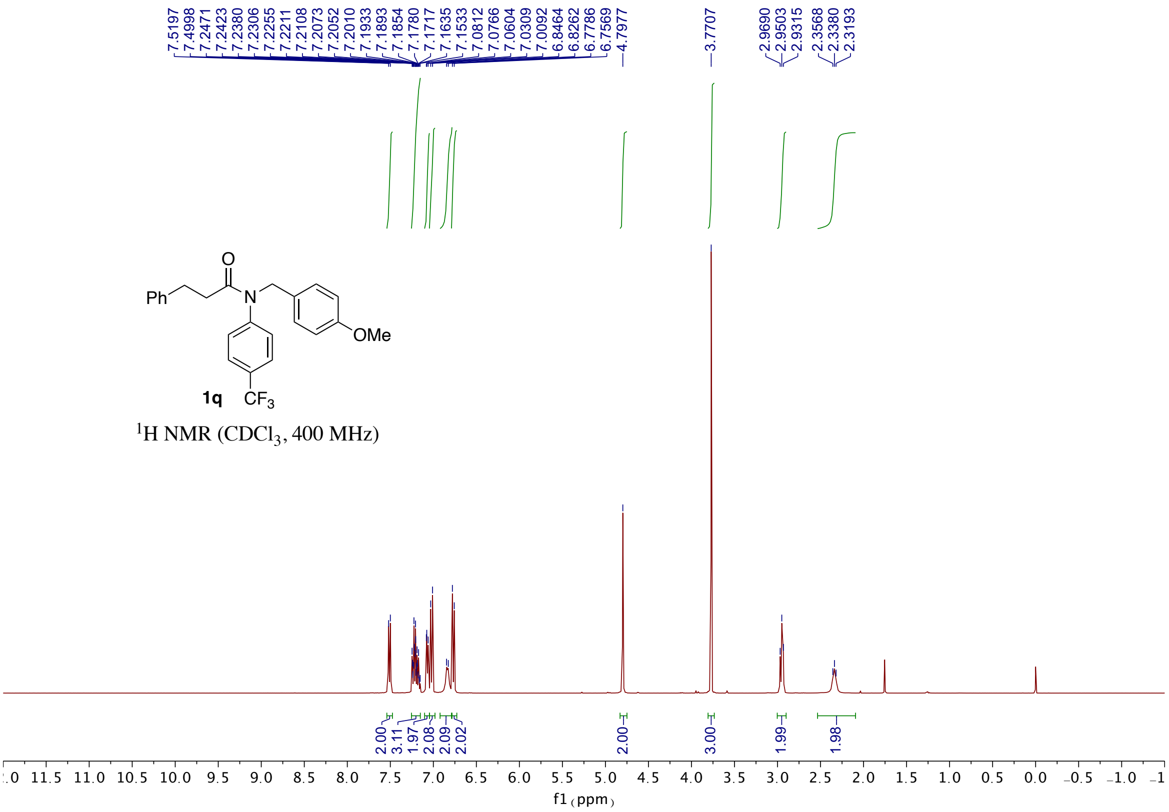

1q

^{1}H NMR ($CDCl_3$, 400 MHz)

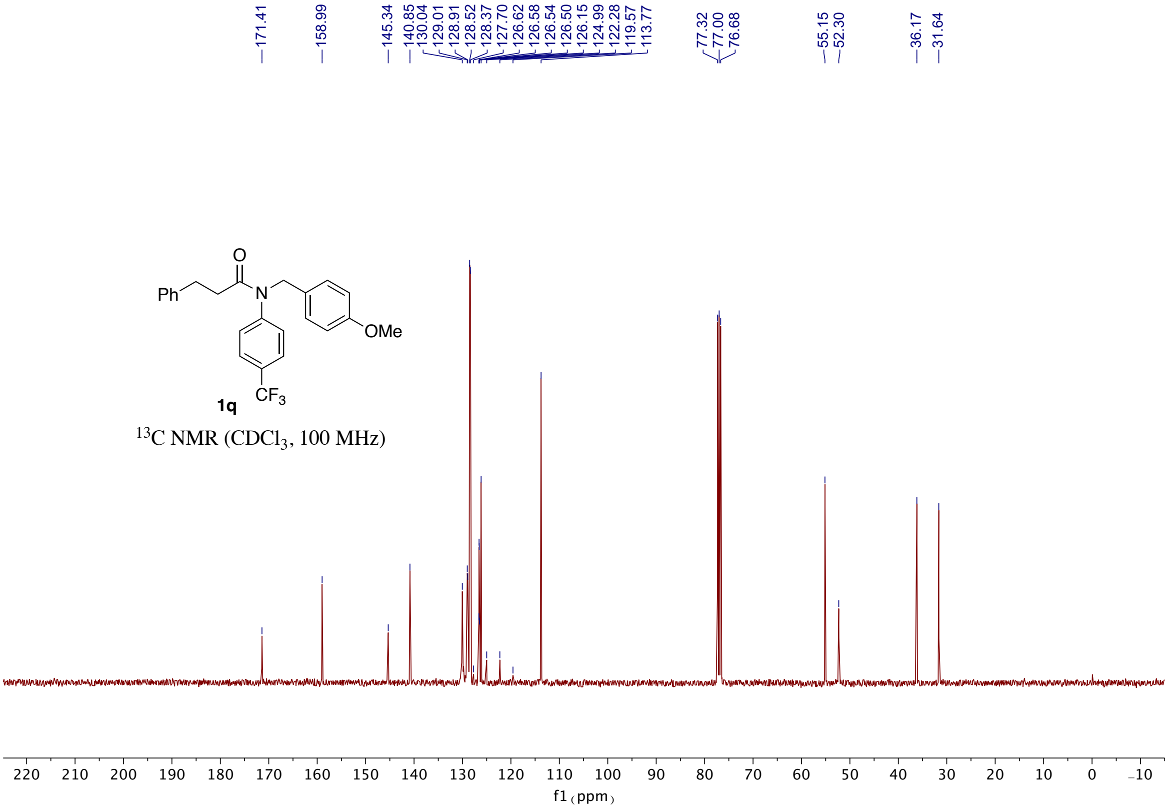

1q

^{13}C NMR ($CDCl_3$, 100 MHz)

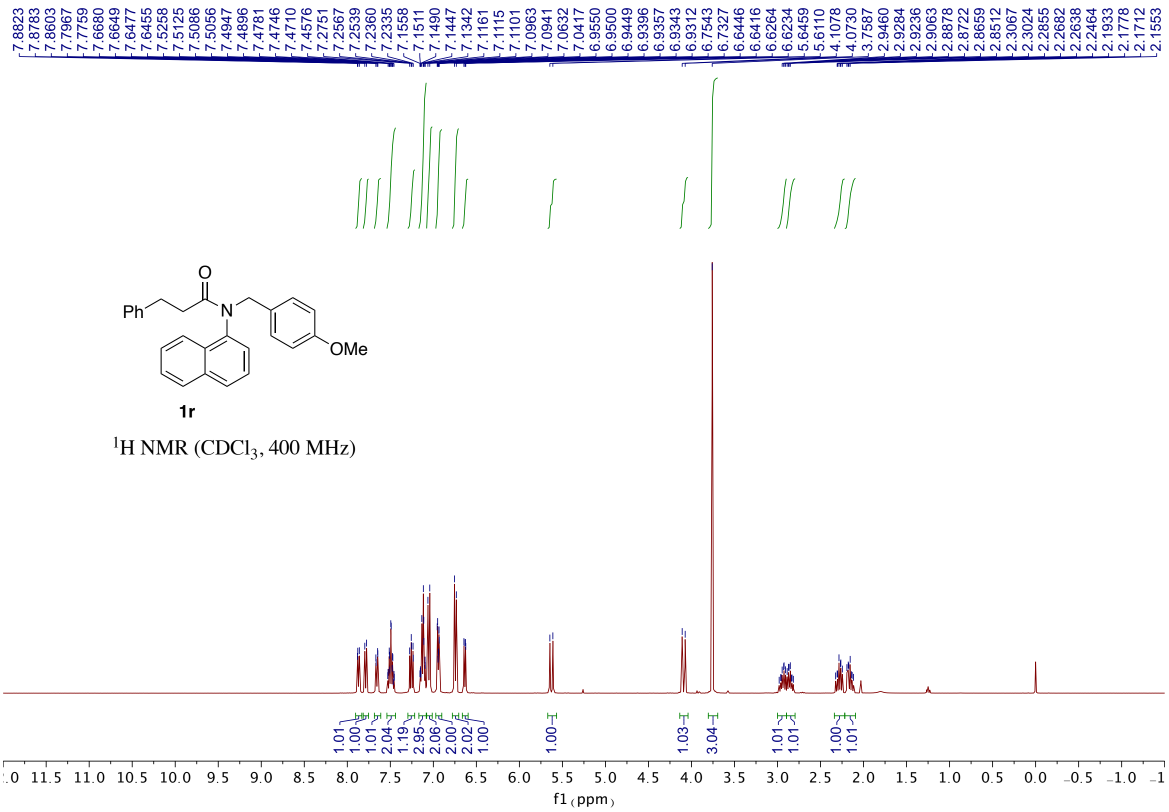

1r

^{1}H NMR ($CDCl_3$, 400 MHz)

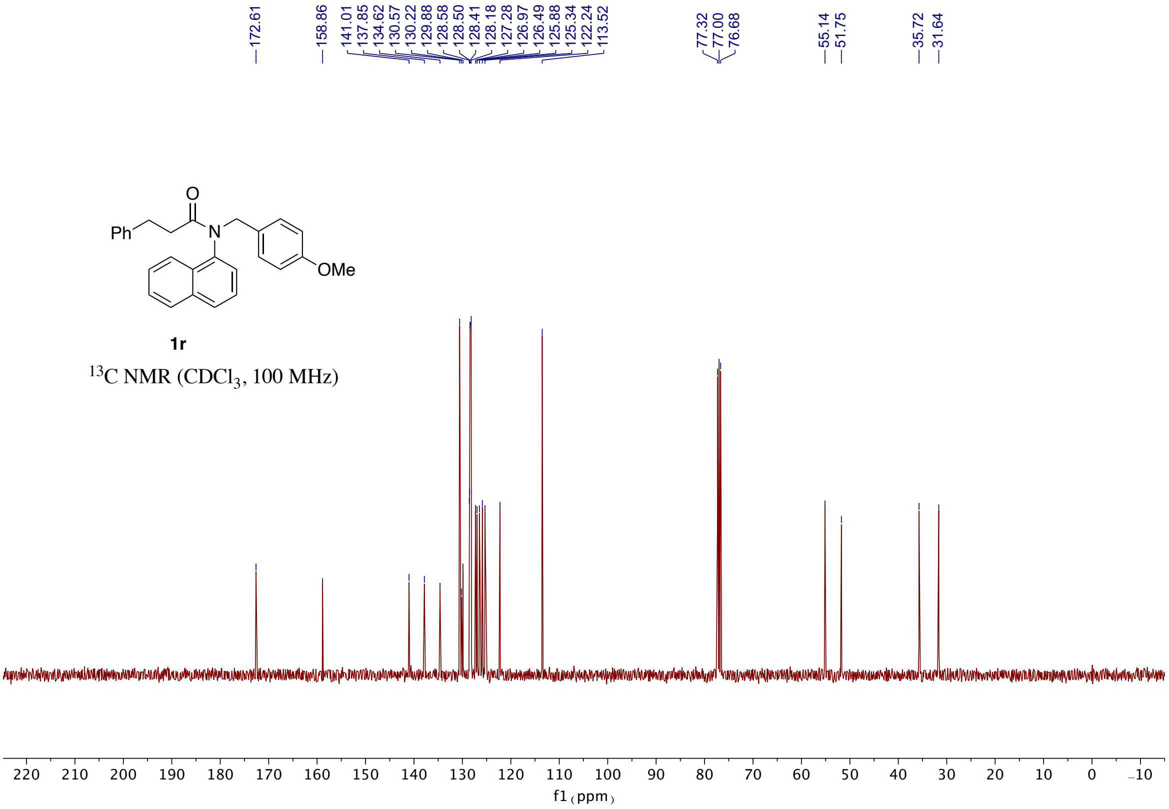

1r

^{13}C NMR ($CDCl_3$, 100 MHz)

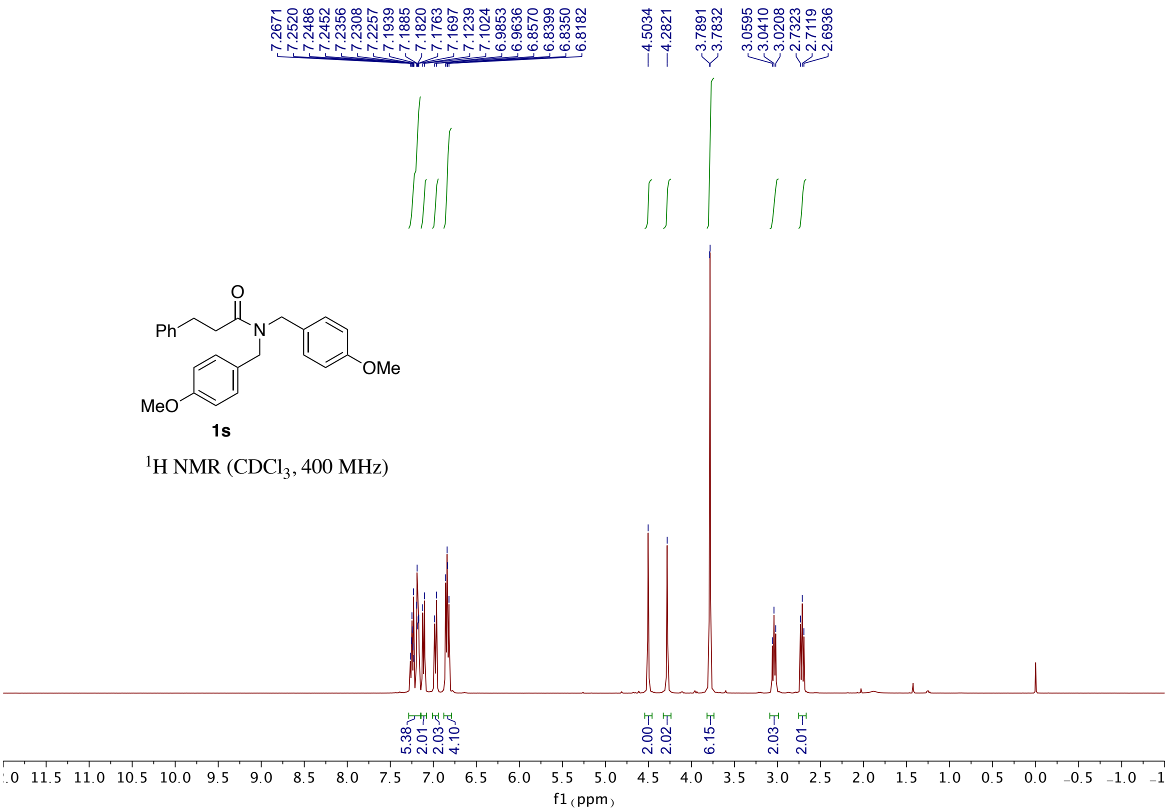

^{1}H NMR ($CDCl_3$, 400 MHz)

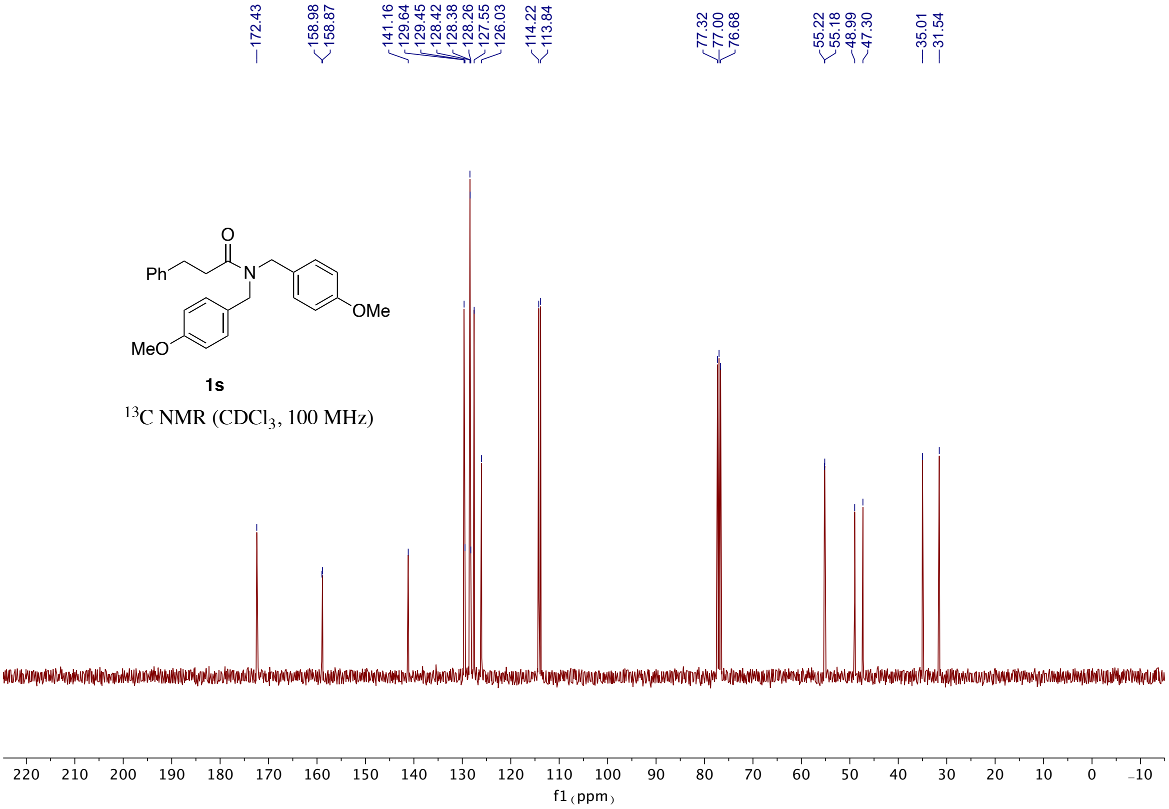

^{13}C NMR ($CDCl_3$, 100 MHz)

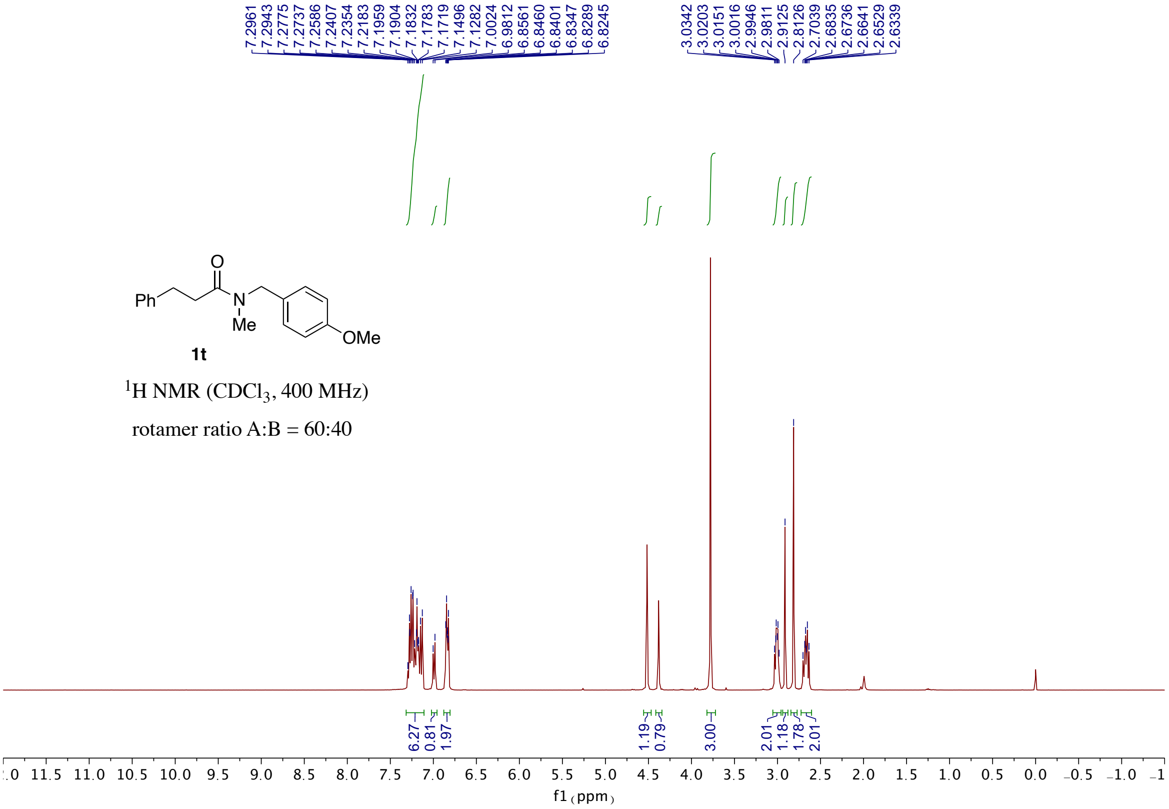
7.2961
7.2943
7.2775
7.2737
7.2586
7.2407
7.2354
7.2183
7.1959
7.1904
7.1832
7.1783
7.1719
7.1496
7.1282
7.0024
6.9812
6.8561
6.8460
6.8401
6.8347
6.8289
6.8245
3.0342
3.0203
3.0151
3.0016
2.9946
2.9811
2.9125
2.8126
2.7039
2.6835
2.6736
2.6641
2.6529
2.6339
Ph
O
N
Me
OMe
1t
^{1}H NMR (CDCl$_3$, 400 MHz)
rotamer ratio A:B = 60:40
6.27
0.81
1.97
1.19
0.79
3.00
2.01
1.18
1.78
2.01
f1 (ppm)

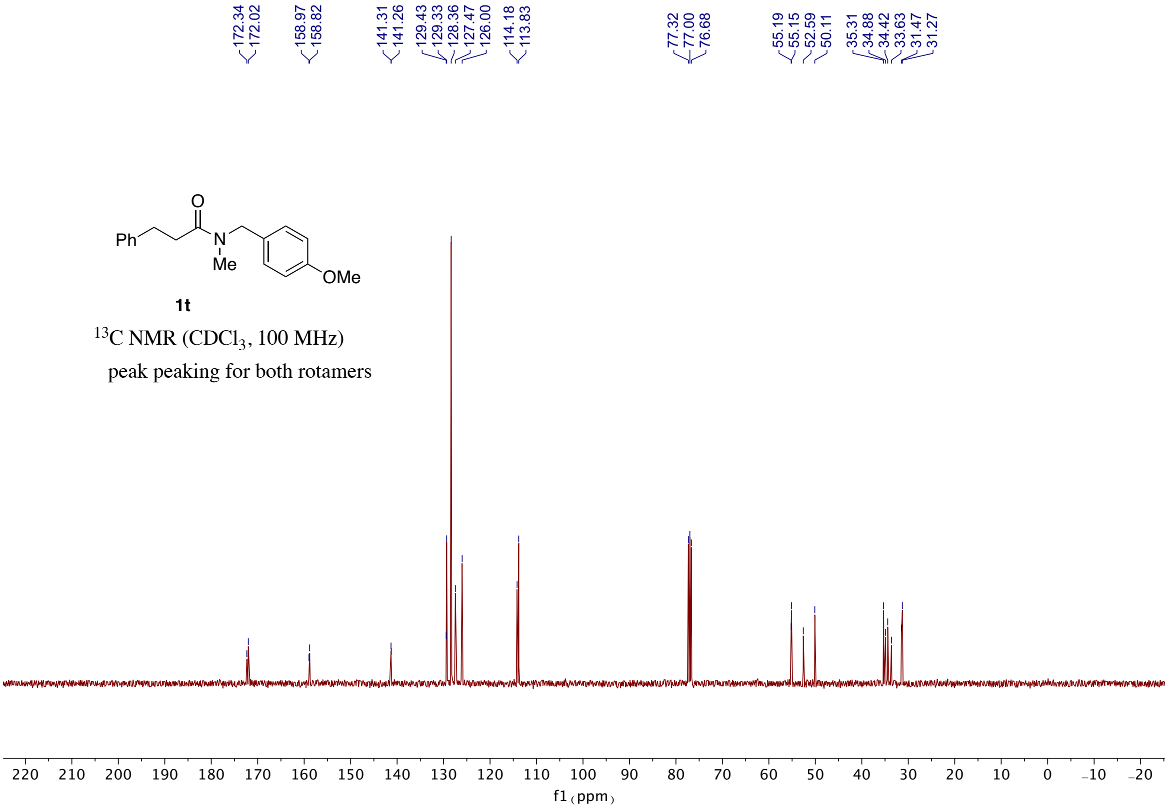
172.34
172.02
158.97
158.82
141.31
141.26
129.43
129.33
128.36
127.47
126.00
114.18
113.83
77.32
77.00
76.68
55.19
55.15
52.59
50.11
35.31
34.88
34.42
33.63
31.47
31.27
Ph
O
N
Me
OMe
1t
^{13}C NMR (CDCl$_3$, 100 MHz)
peak peaking for both rotamers
f1 (ppm)

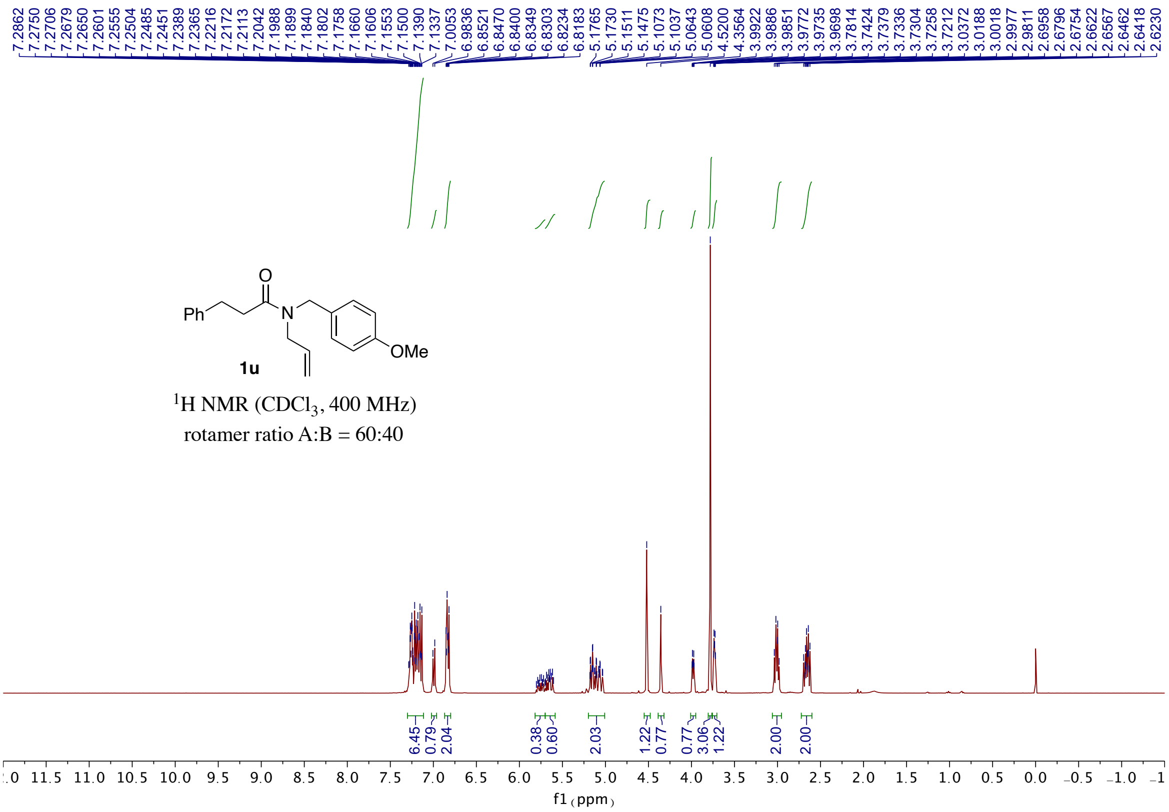
1u
^{1}H NMR ($CDCl_3$, 400 MHz)
rotamer ratio A:B = 60:40
f1 (ppm)

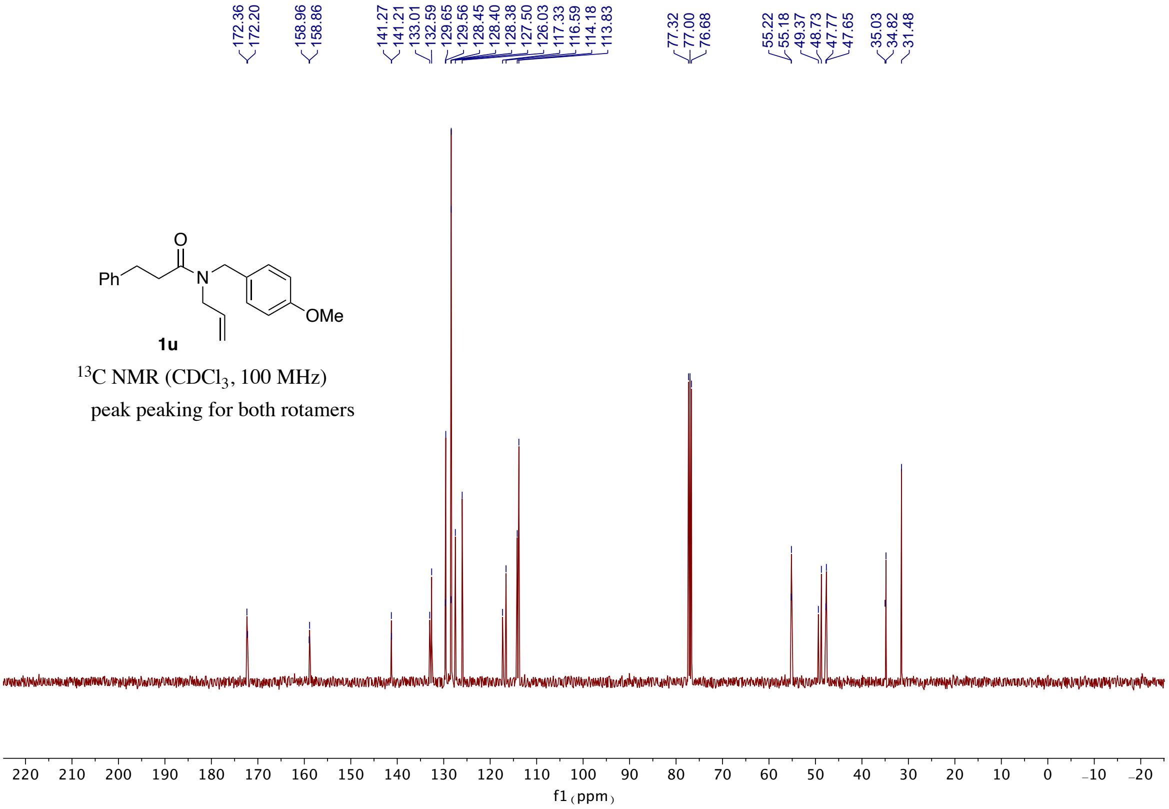
1u
^{13}C NMR ($CDCl_3$, 100 MHz)
peak peaking for both rotamers
f1 (ppm)

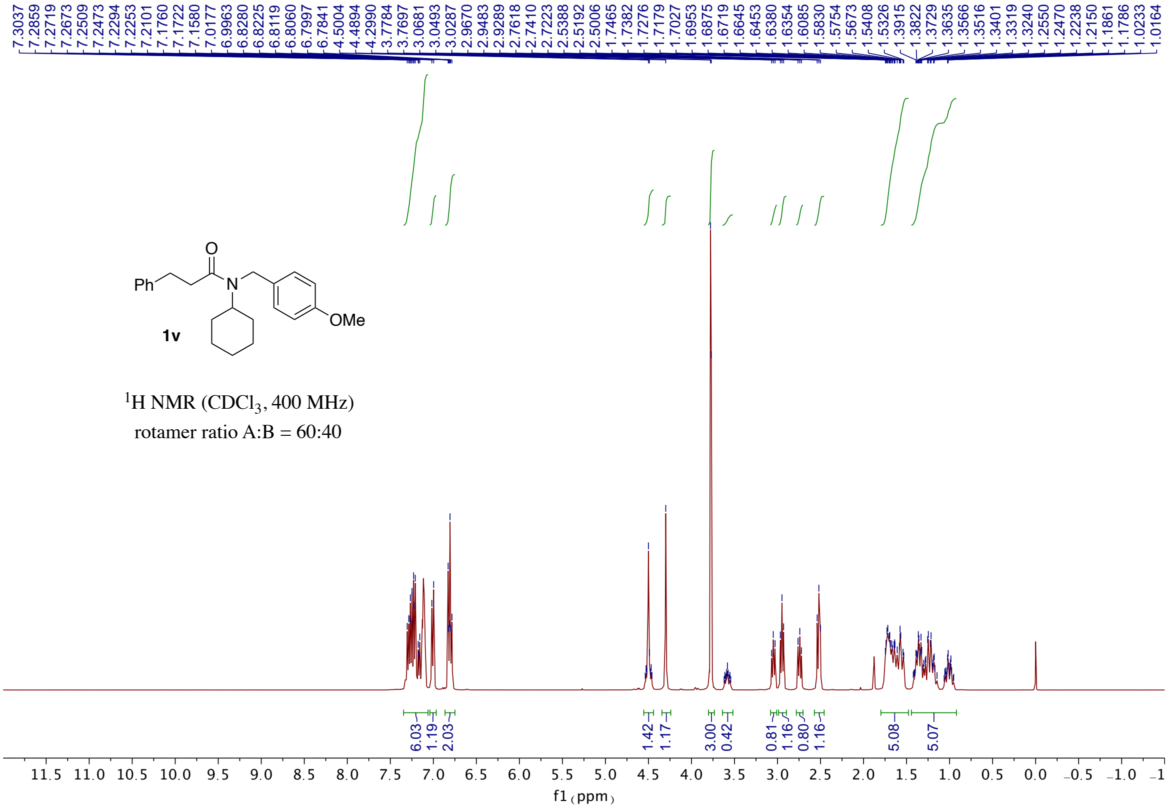
7.3037 7.2859 7.2719 7.2673 7.2509 7.2473 7.2294 7.2253 7.2101 7.1760 7.1722 7.1580 7.0177 6.9963 6.8280 6.8225 6.8119 6.8060 6.7997 6.7841 4.5004 4.4894 4.2990 3.7784 3.7697 3.0681 3.0493 3.0287 2.9670 2.9483 2.9289 2.7618 2.7410 2.7223 2.5388 2.5192 2.5006 1.7465 1.7382 1.7276 1.7179 1.7027 1.6953 1.6875 1.6719 1.6645 1.6453 1.6380 1.6354 1.6085 1.5830 1.5754 1.5673 1.5408 1.5326 1.3915 1.3822 1.3729 1.3635 1.3566 1.3516 1.3401 1.3319 1.3240 1.2550 1.2470 1.2238 1.2150 1.1861 1.1786 1.0233 1.0164
O
Ph
N
OMe
1v
^{1}H NMR (CDCl$_3$, 400 MHz)
rotamer ratio A:B = 60:40
6.03 1.19 2.03 1.42 1.17 3.00 0.42 0.81 1.16 0.80 1.16 5.08 5.07
f1 (ppm)

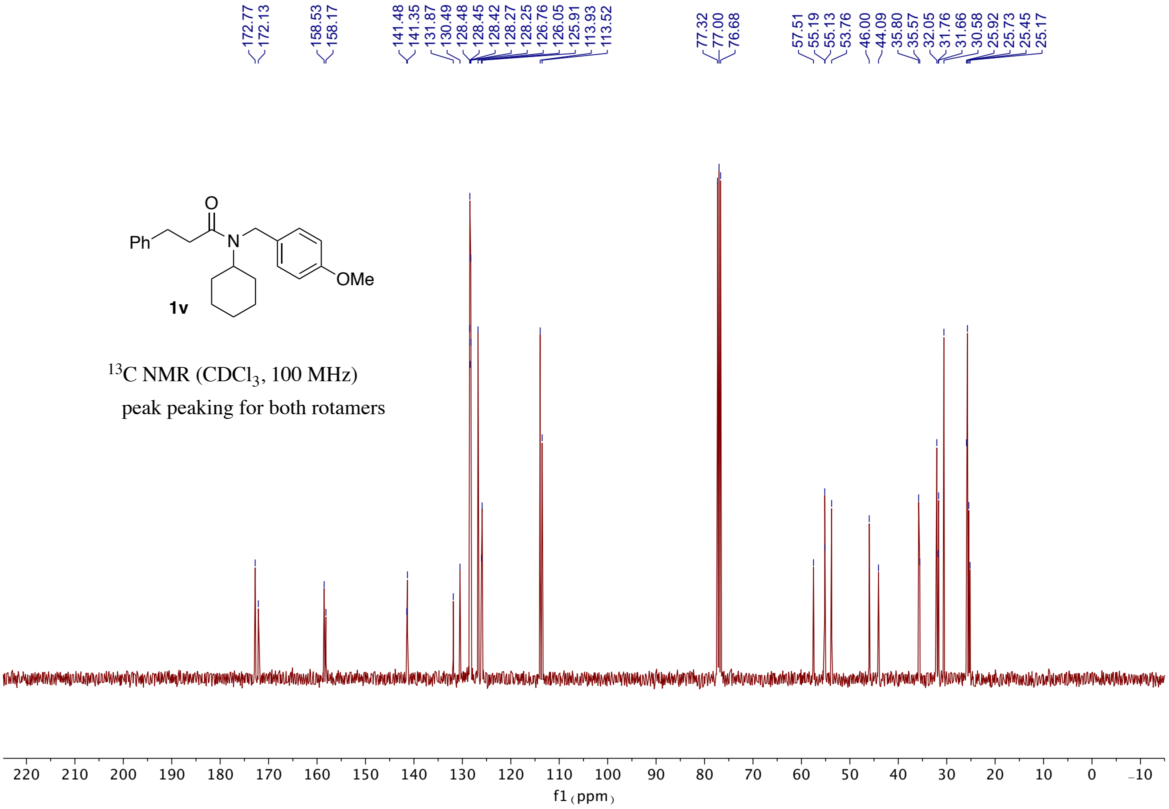
172.77 172.13 158.53 158.17 141.48 141.35 131.87 130.49 128.48 128.45 128.42 128.27 128.25 126.76 126.05 125.91 113.93 113.52 77.32 77.00 76.68 57.51 55.19 55.13 53.76 46.00 44.09 35.80 35.57 32.05 31.76 31.66 30.58 25.92 25.73 25.45 25.17
O
Ph
N
OMe
1v
^{13}C NMR (CDCl$_3$, 100 MHz)
peak peaking for both rotamers
f1 (ppm)

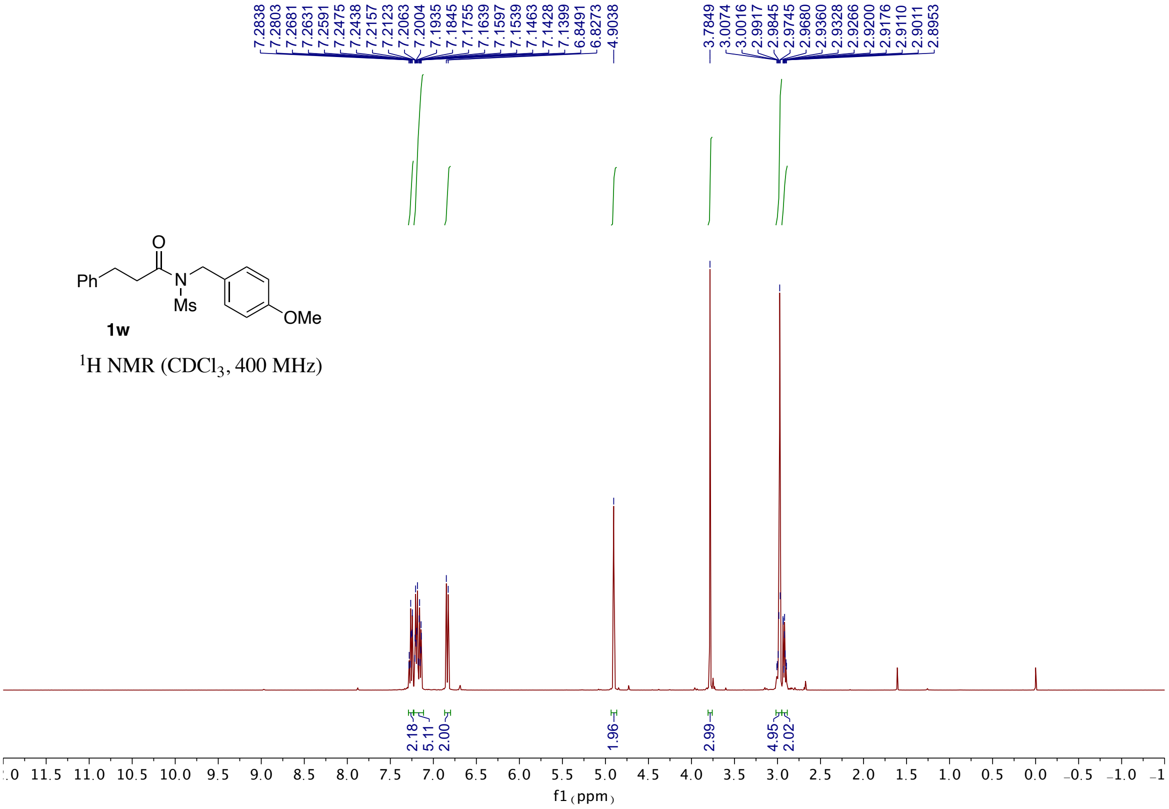
7.2838
7.2803
7.2681
7.2631
7.2591
7.2475
7.2438
7.2157
7.2123
7.2063
7.2004
7.1935
7.1845
7.1755
7.1639
7.1597
7.1539
7.1463
7.1428
7.1399
6.8491
6.8273
4.9038
3.7849
3.0074
3.0016
2.9917
2.9845
2.9745
2.9680
2.9360
2.9328
2.9266
2.9200
2.9176
2.9110
2.9011
2.8953
Ph
O
N
Ms
OMe
1w
^{1}H NMR ($CDCl_3$, 400 MHz)
2.18
5.11
2.00
1.96
2.99
4.95
2.02
f1 (ppm)

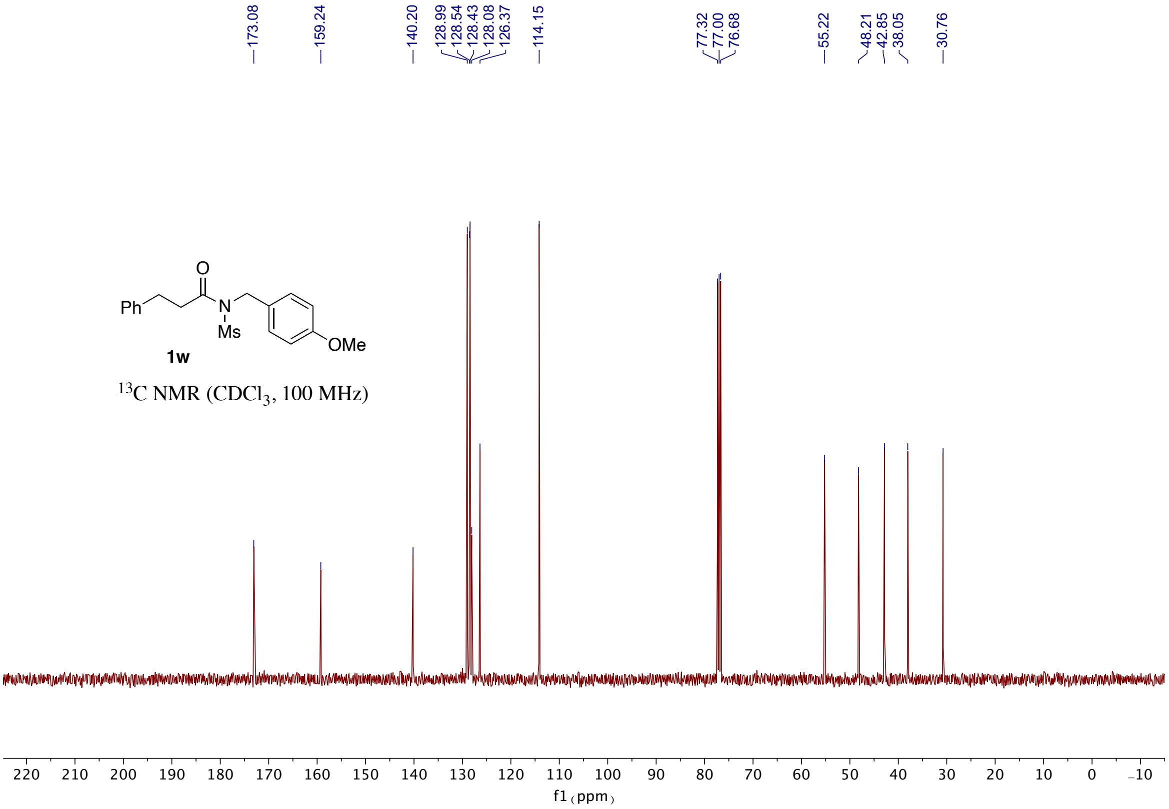
173.08
159.24
140.20
128.99
128.54
128.43
128.08
126.37
114.15
77.32
77.00
76.68
55.22
48.21
42.85
38.05
30.76
Ph
O
N
Ms
OMe
1w
^{13}C NMR ($CDCl_3$, 100 MHz)
f1 (ppm)

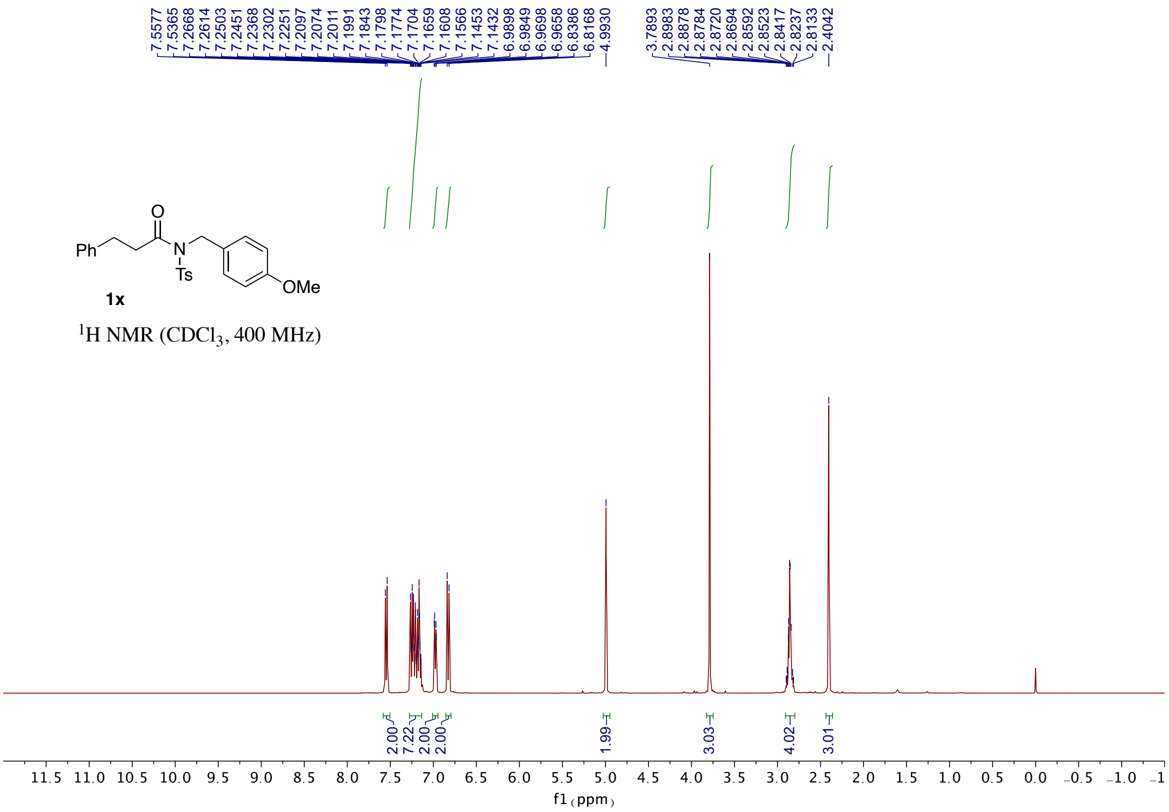

1x

^{1}H NMR ($CDCl_3$, 400 MHz)

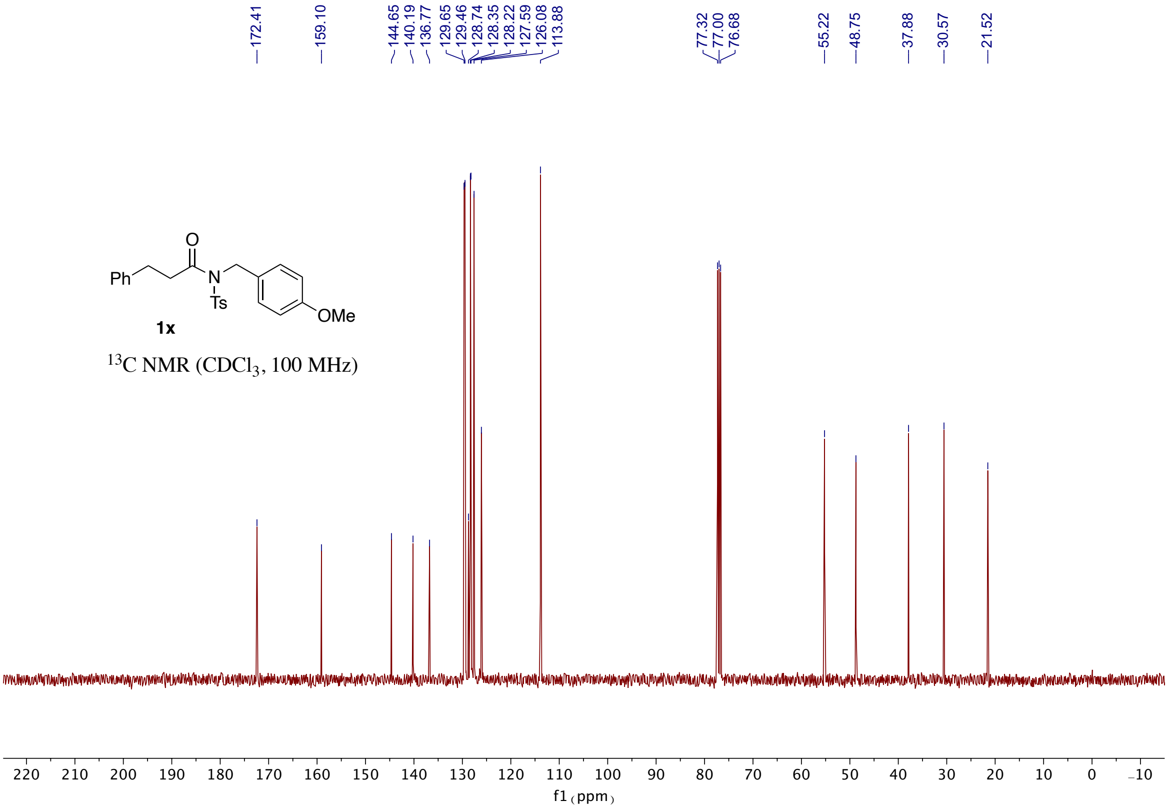

1x

^{13}C NMR ($CDCl_3$, 100 MHz)

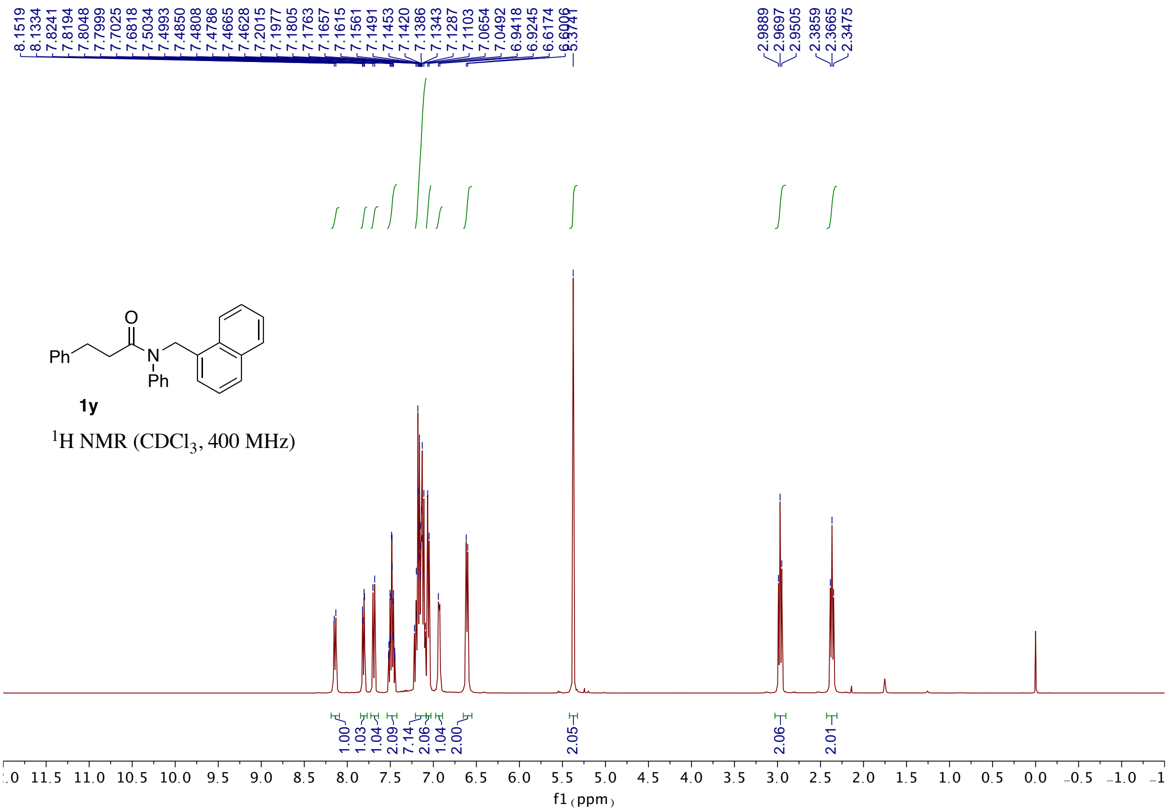
8.1519
8.1334
7.8241
7.8194
7.8048
7.7999
7.7025
7.6818
7.5034
7.4993
7.4850
7.4808
7.4786
7.4665
7.4628
7.2015
7.1977
7.1805
7.1763
7.1657
7.1615
7.1561
7.1491
7.1453
7.1420
7.1386
7.1343
7.1287
7.1103
7.0654
7.0492
6.9418
6.9245
6.6174
6.6006
5.3741
2.9889
2.9697
2.9505
2.3859
2.3665
2.3475
Ph
O
N
Ph
1y
^{1}H NMR (CDCl$_3$, 400 MHz)
1.00
1.03
1.04
2.09
7.14
2.06
1.04
2.00
2.05
2.06
2.01
f1 (ppm)

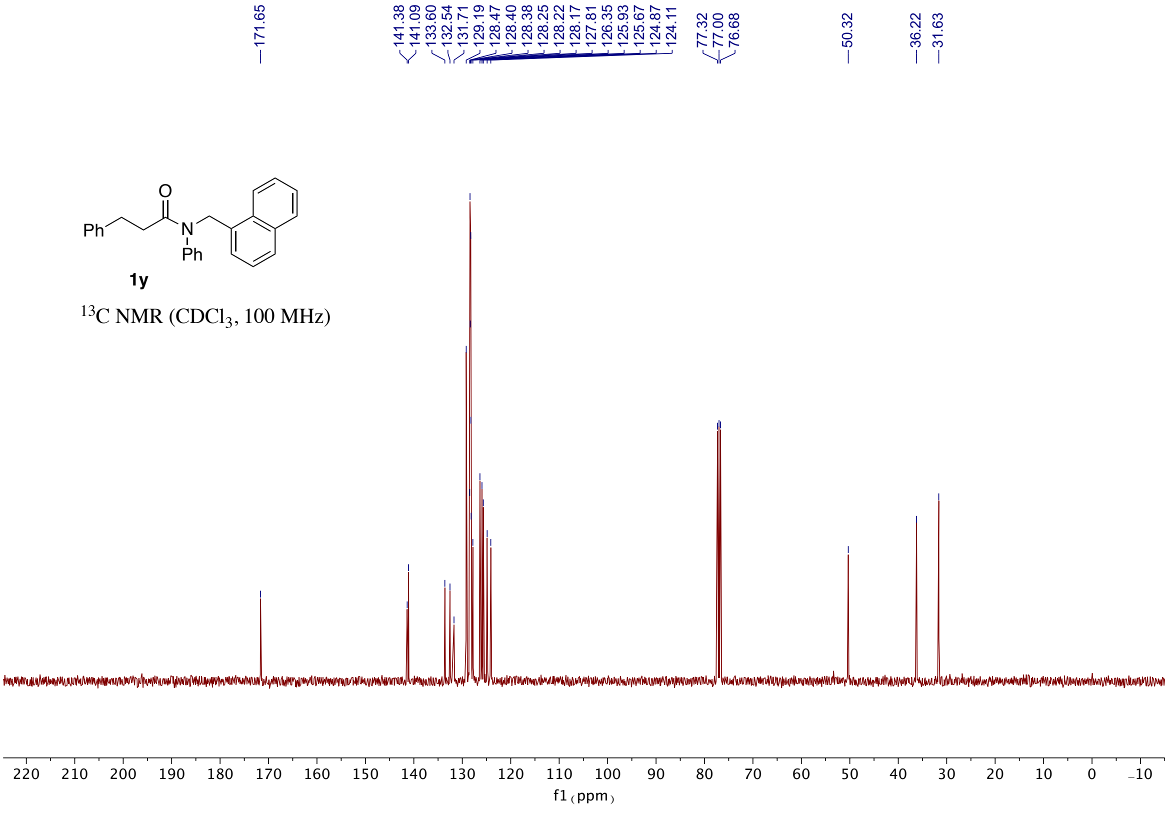
171.65
141.38
141.09
133.60
132.54
131.71
129.19
128.47
128.40
128.38
128.25
128.22
128.17
127.81
126.35
125.93
125.67
124.87
124.11
77.32
77.00
76.68
50.32
36.22
31.63
Ph
O
N
Ph
1y
^{13}C NMR (CDCl$_3$, 100 MHz)
f1 (ppm)

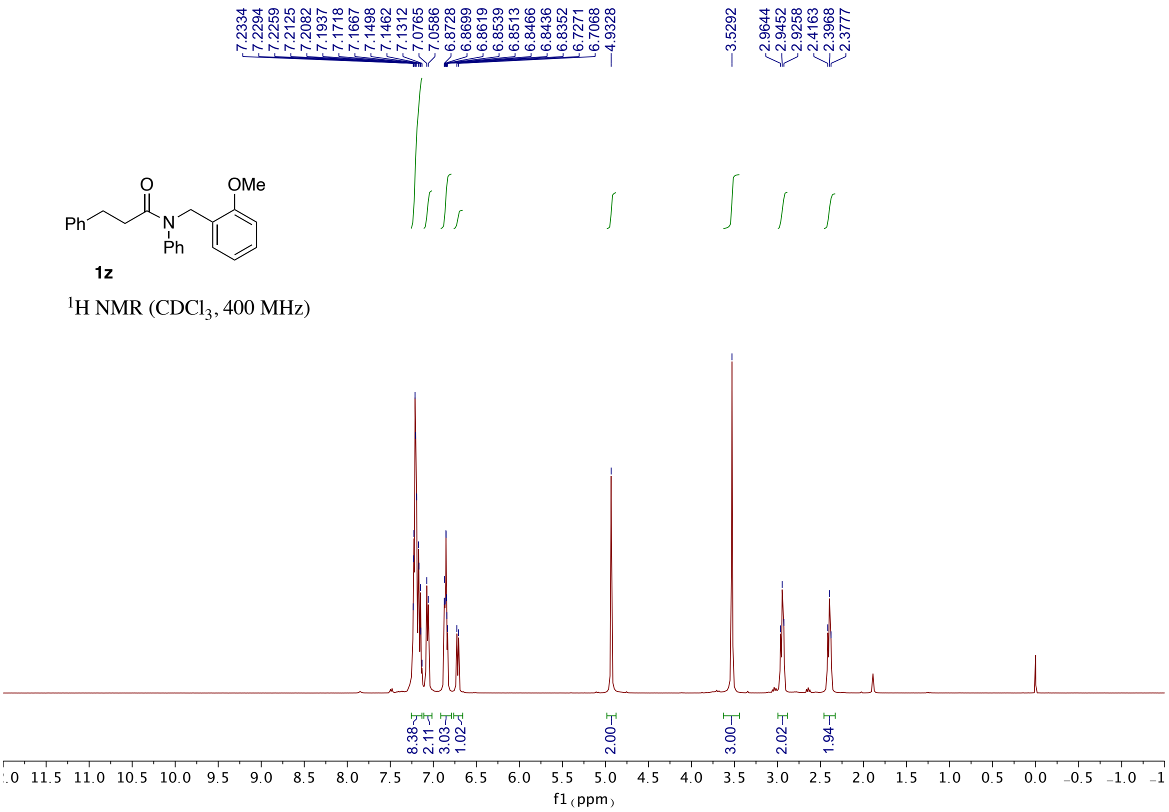
7.2334
7.2294
7.2259
7.2125
7.2082
7.1937
7.1718
7.1667
7.1498
7.1462
7.1312
7.0765
7.0586
6.8728
6.8699
6.8619
6.8539
6.8513
6.8466
6.8436
6.8352
6.7271
6.7068
4.9328
3.5292
2.9644
2.9452
2.9258
2.4163
2.3968
2.3777
O
OMe
Ph
N
Ph
1z
^{1}H NMR ($CDCl_3$, 400 MHz)
8.38
2.11
3.03
1.02
2.00
3.00
2.02
1.94
f1 (ppm)

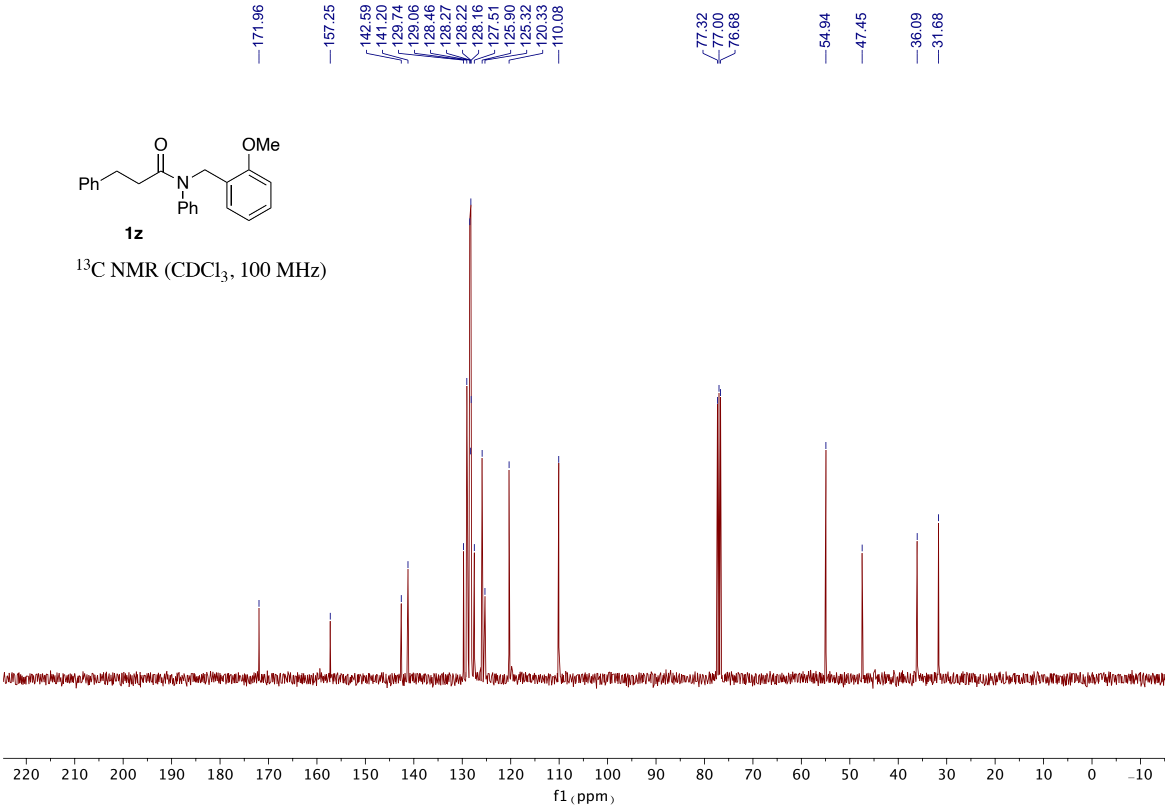
171.96
157.25
142.59
141.20
129.74
129.06
128.46
128.27
128.22
128.16
127.51
125.90
125.32
120.33
110.08
77.32
77.00
76.68
54.94
47.45
36.09
31.68
O
OMe
Ph
N
Ph
1z
^{13}C NMR ($CDCl_3$, 100 MHz)
f1 (ppm)

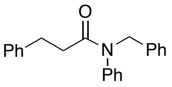

1aa

^{1}H NMR ($CDCl_3$, 400 MHz)

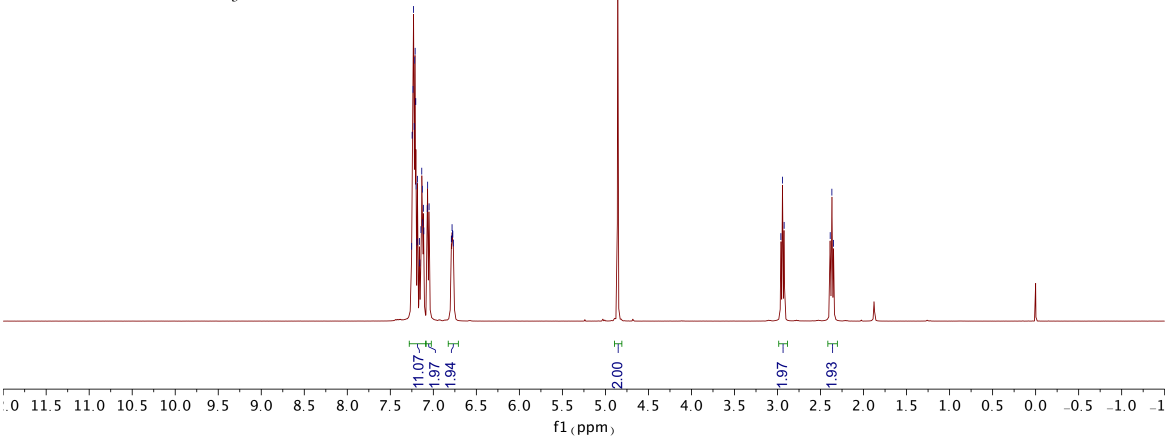

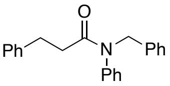

1aa

^{13}C NMR ($CDCl_3$, 100 MHz)

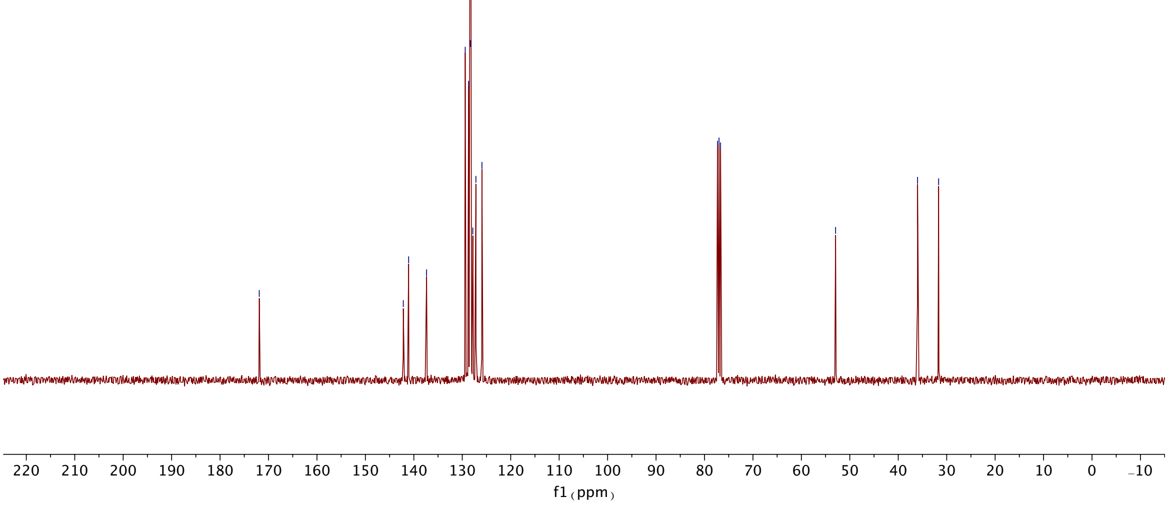

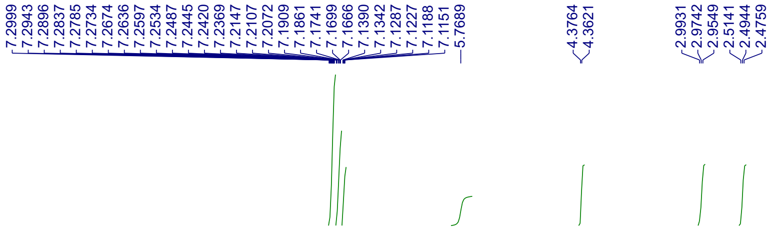

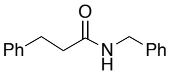

3a

^{1}H NMR ($CDCl_3$, 400 MHz)

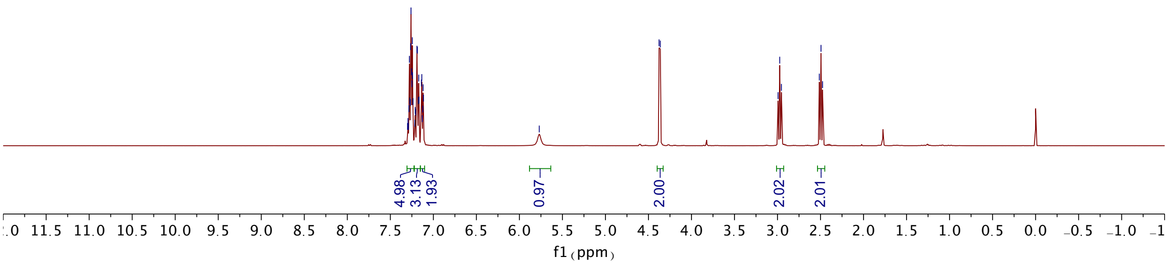

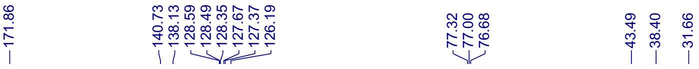

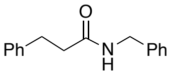

3a

^{13}C NMR ($CDCl_3$, 100 MHz)

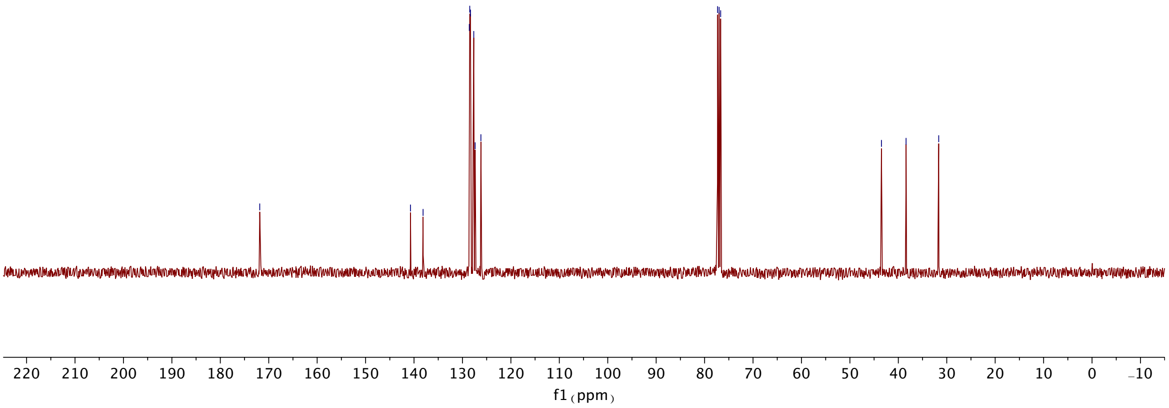

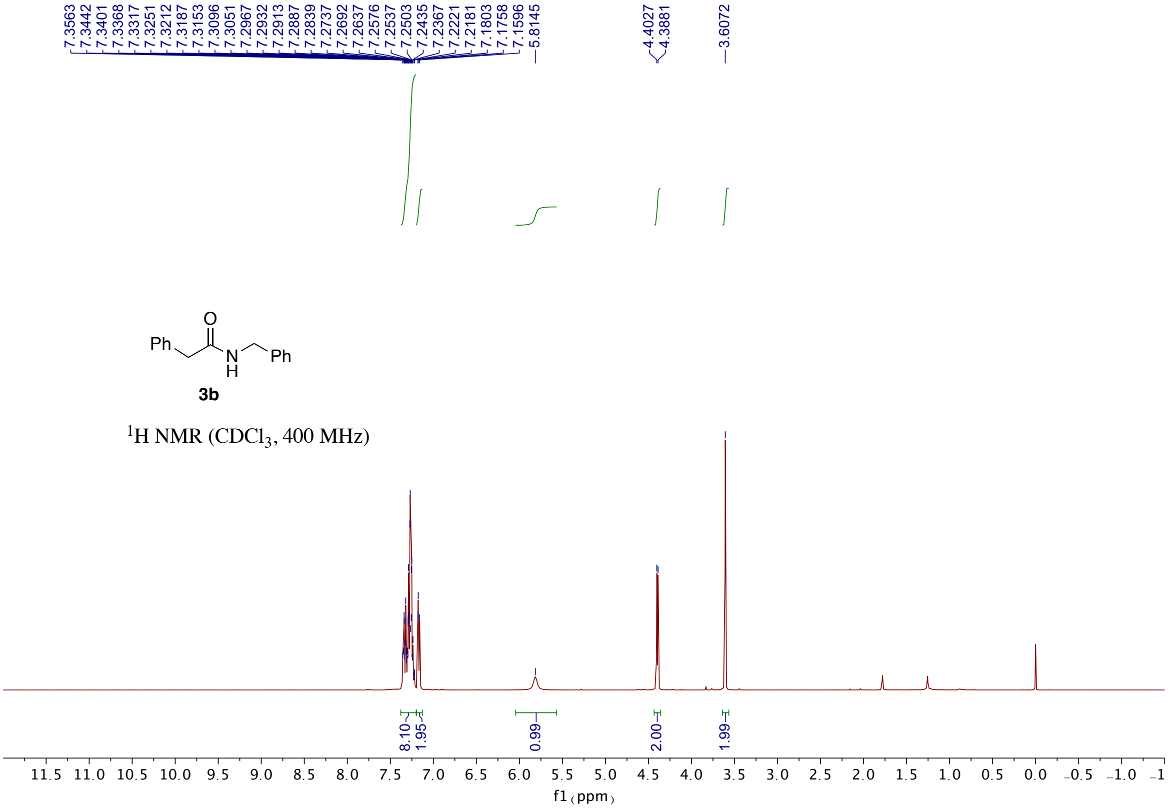

3b

^{1}H NMR ($CDCl_3$, 400 MHz)

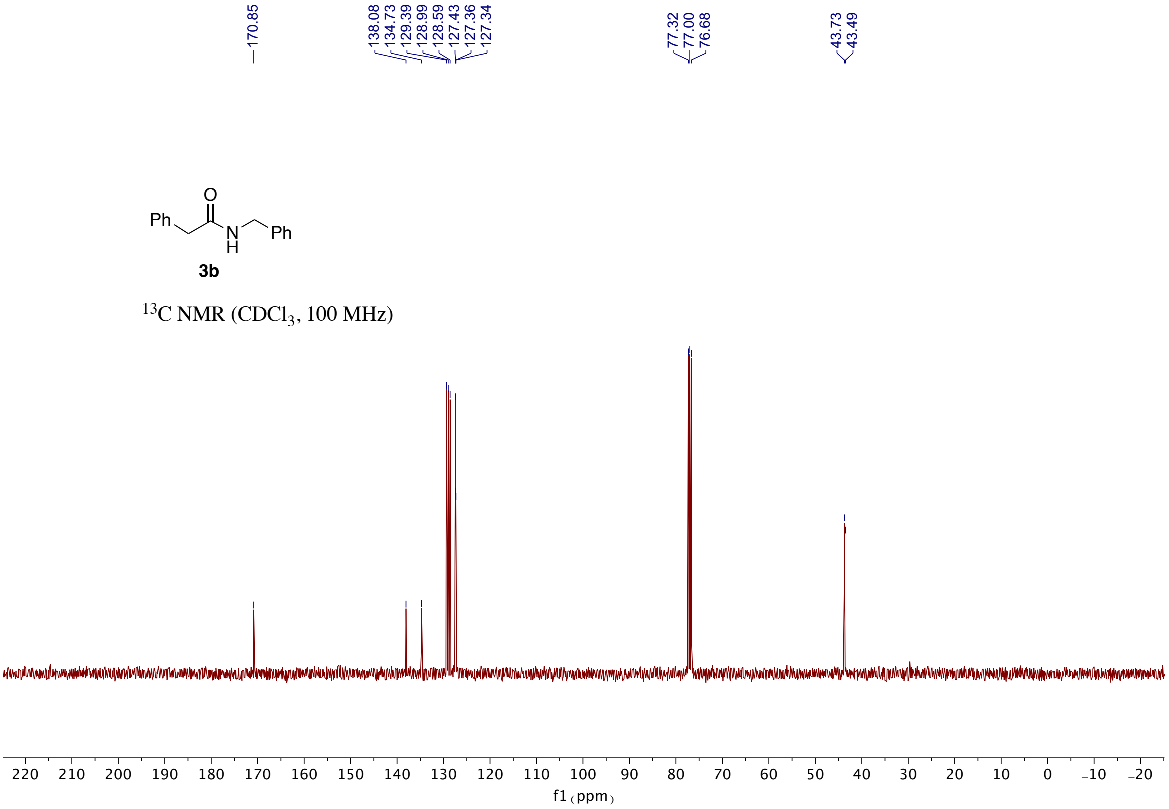

3b

^{13}C NMR ($CDCl_3$, 100 MHz)

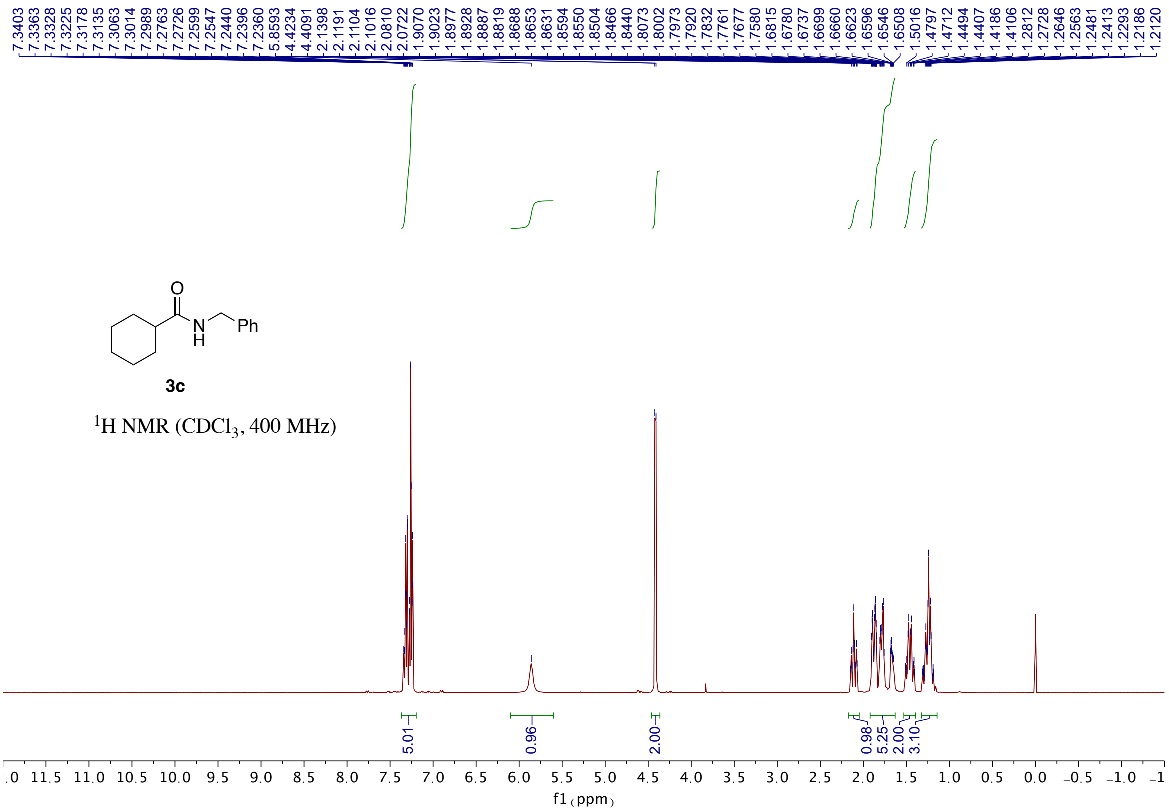

3c

^{1}H NMR ($CDCl_3$, 400 MHz)

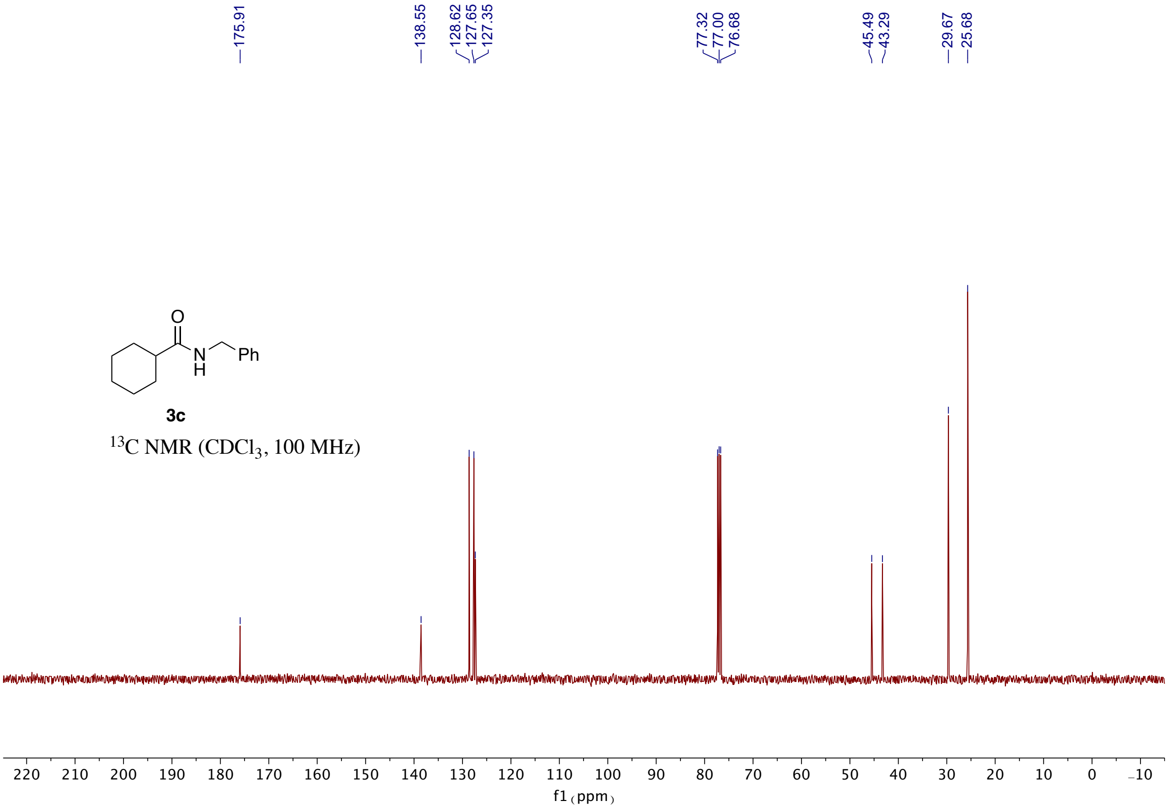

3c

^{13}C NMR ($CDCl_3$, 100 MHz)

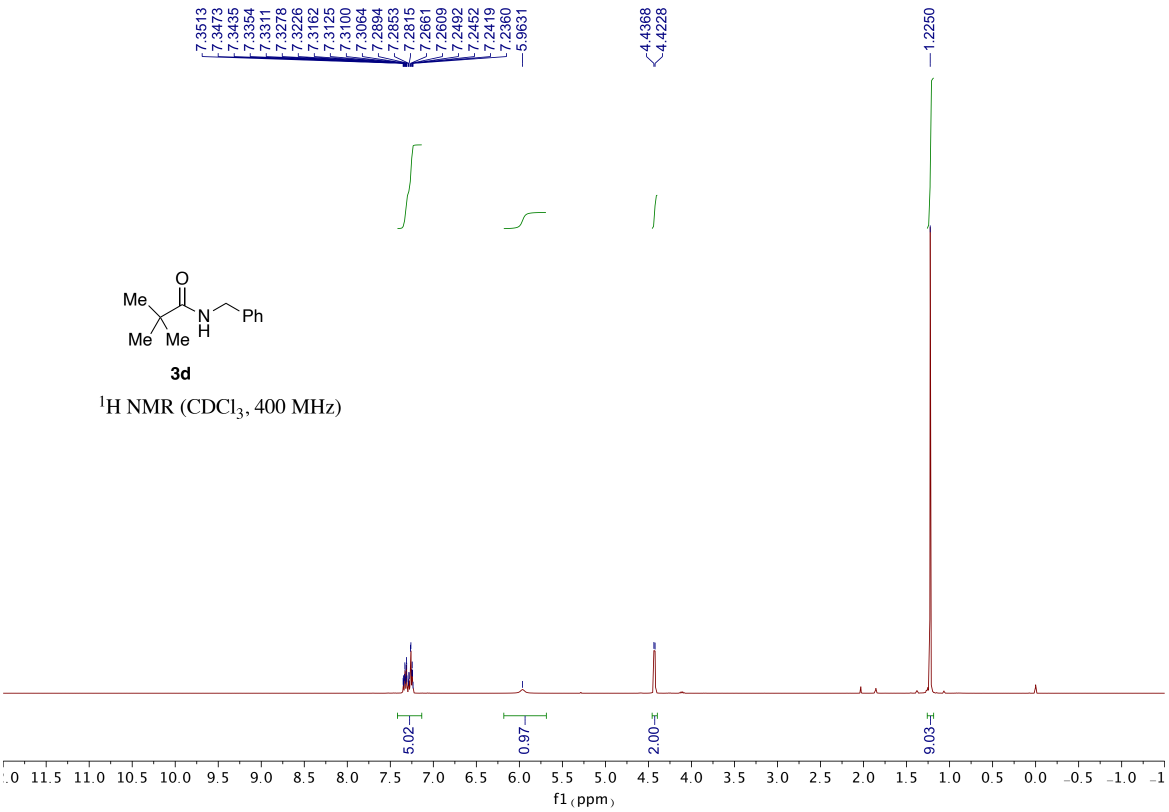

3d

^{1}H NMR ($CDCl_3$, 400 MHz)

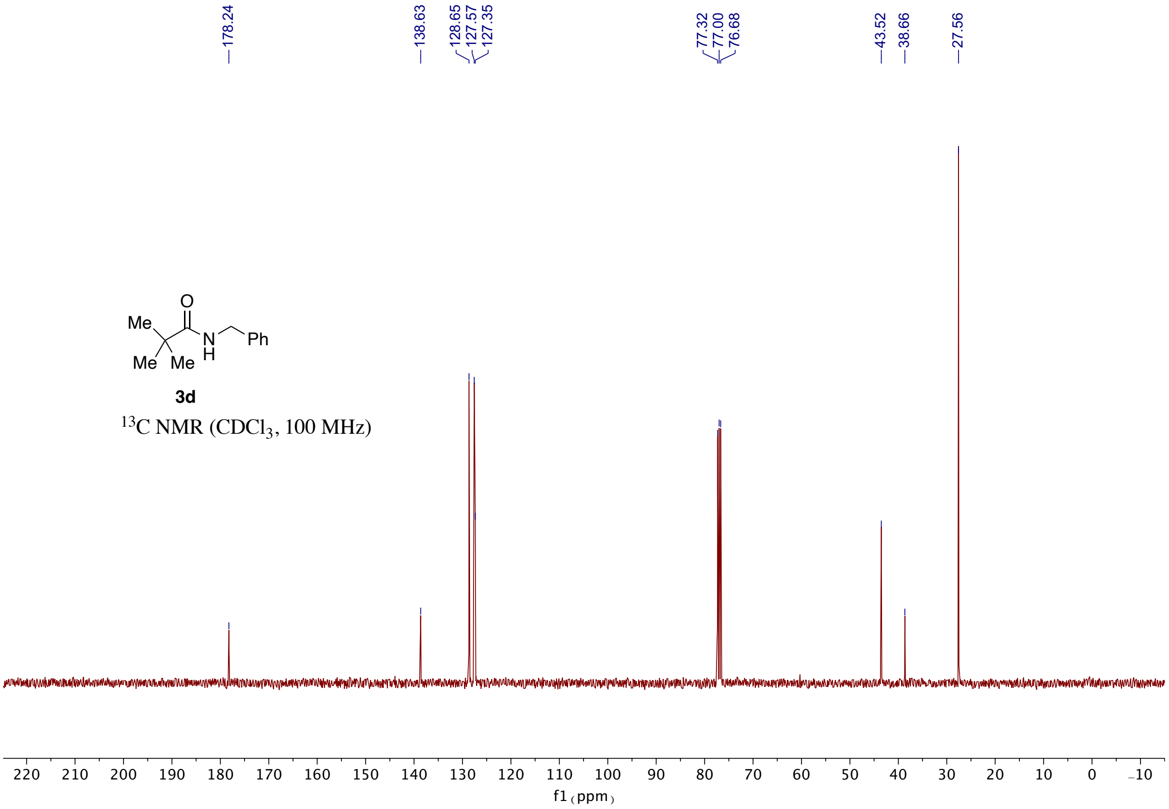

3d

^{13}C NMR ($CDCl_3$, 100 MHz)

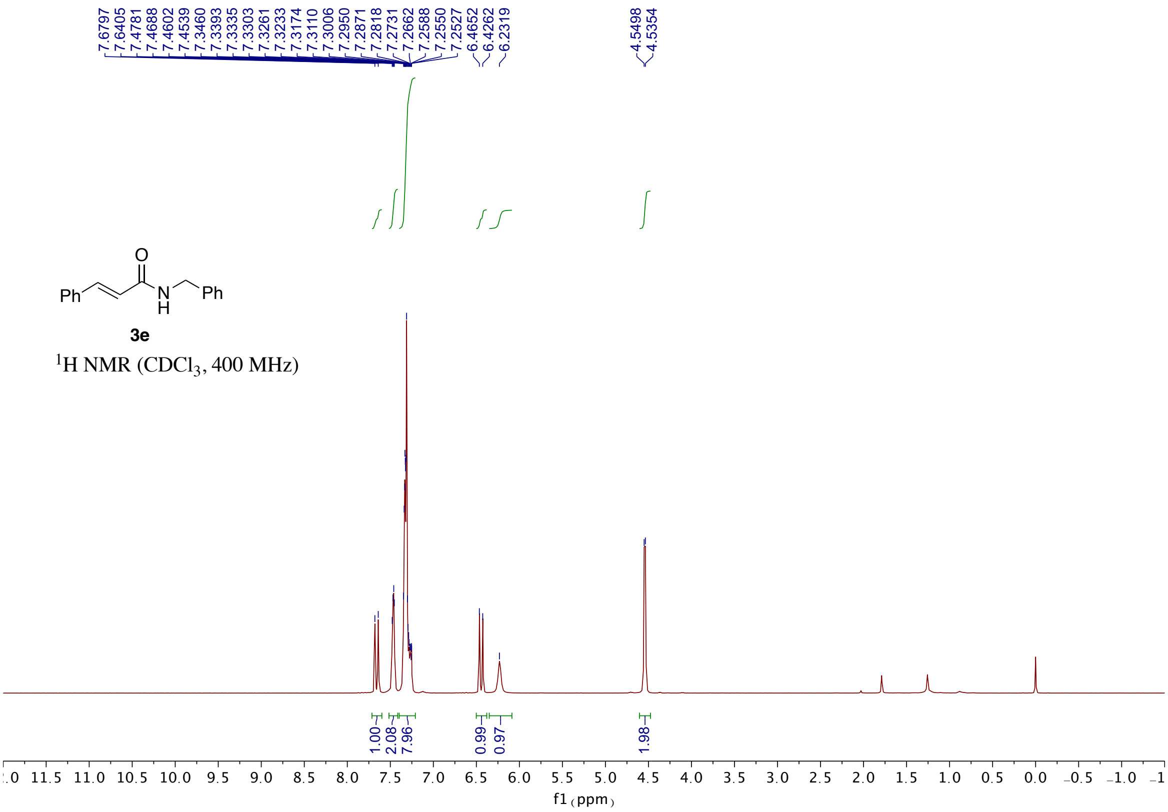

7.6797
7.6405
7.4781
7.4688
7.4602
7.4539
7.3460
7.3393
7.3335
7.3303
7.3261
7.3233
7.3174
7.3110
7.3006
7.2950
7.2871
7.2818
7.2731
7.2662
7.2588
7.2550
7.2527
6.4652
6.4262
6.2319
4.5498
4.5354
Ph
O
N
H
Ph
3e
1H NMR (CDCl3, 400 MHz)
1.00
2.08
7.96
0.99
0.97
1.98
f1 (ppm)

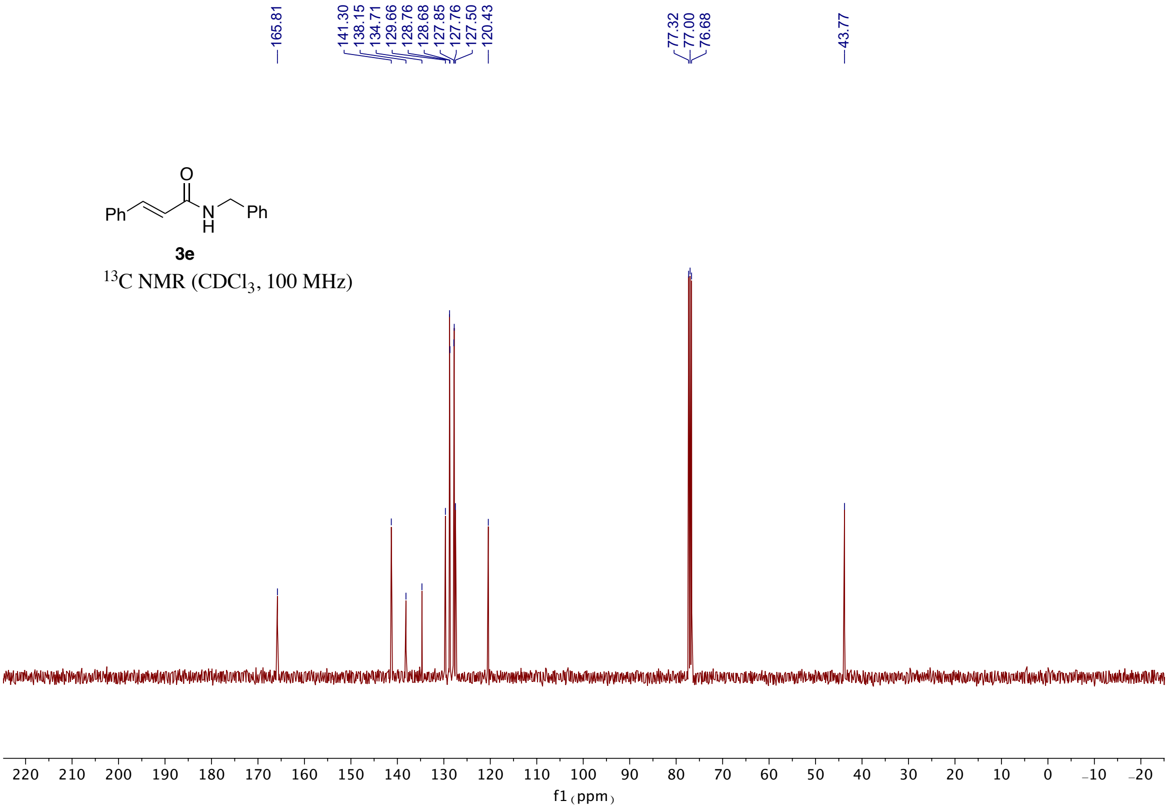

165.81
141.30
138.15
134.71
129.66
128.76
128.68
127.85
127.76
127.50
120.43
77.32
77.00
76.68
43.77
Ph
O
N
H
Ph
3e
13C NMR (CDCl3, 100 MHz)
f1 (ppm)

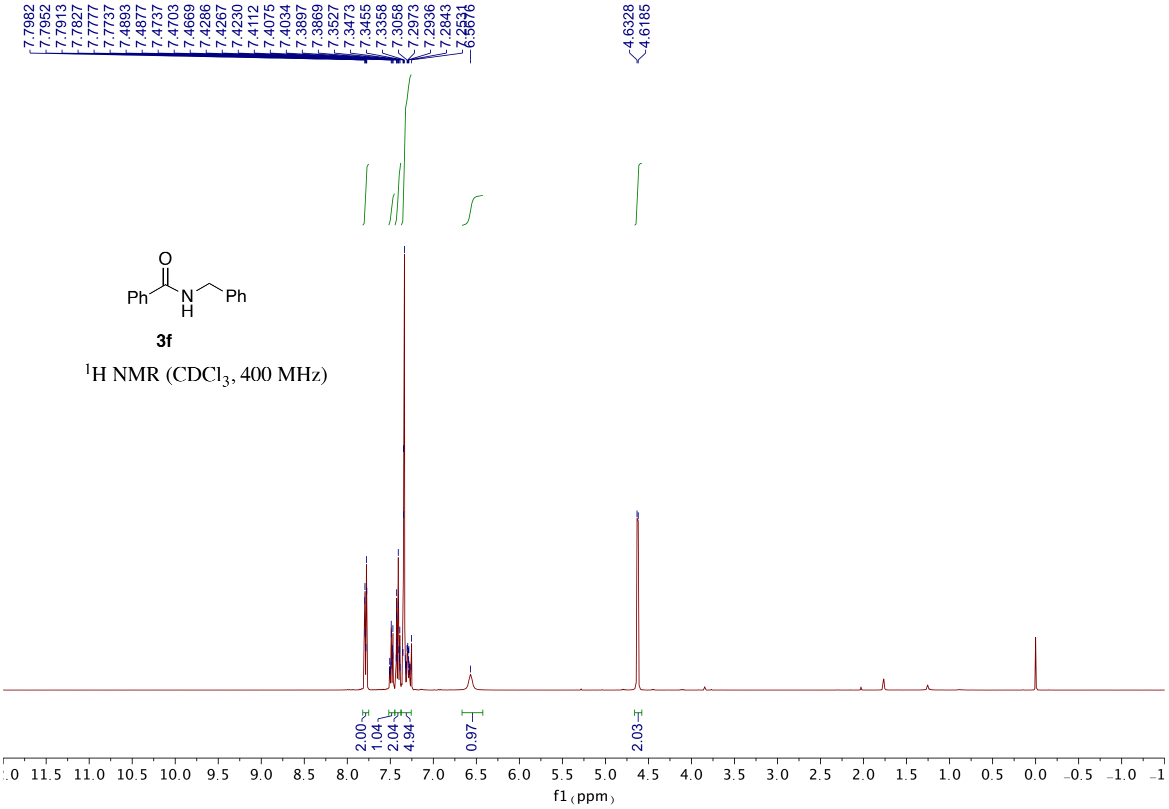
7.7982
7.7952
7.7913
7.7827
7.7777
7.7737
7.4893
7.4877
7.4737
7.4703
7.4669
7.4286
7.4267
7.4230
7.4112
7.4075
7.4034
7.3897
7.3869
7.3527
7.3473
7.3455
7.3358
7.3058
7.2973
7.2936
7.2843
4.6328
4.6185
Ph
O
N
H
Ph
3f
1H NMR (CDCl3, 400 MHz)
2.00
1.04
2.04
4.94
0.97
2.03
f1 (ppm)

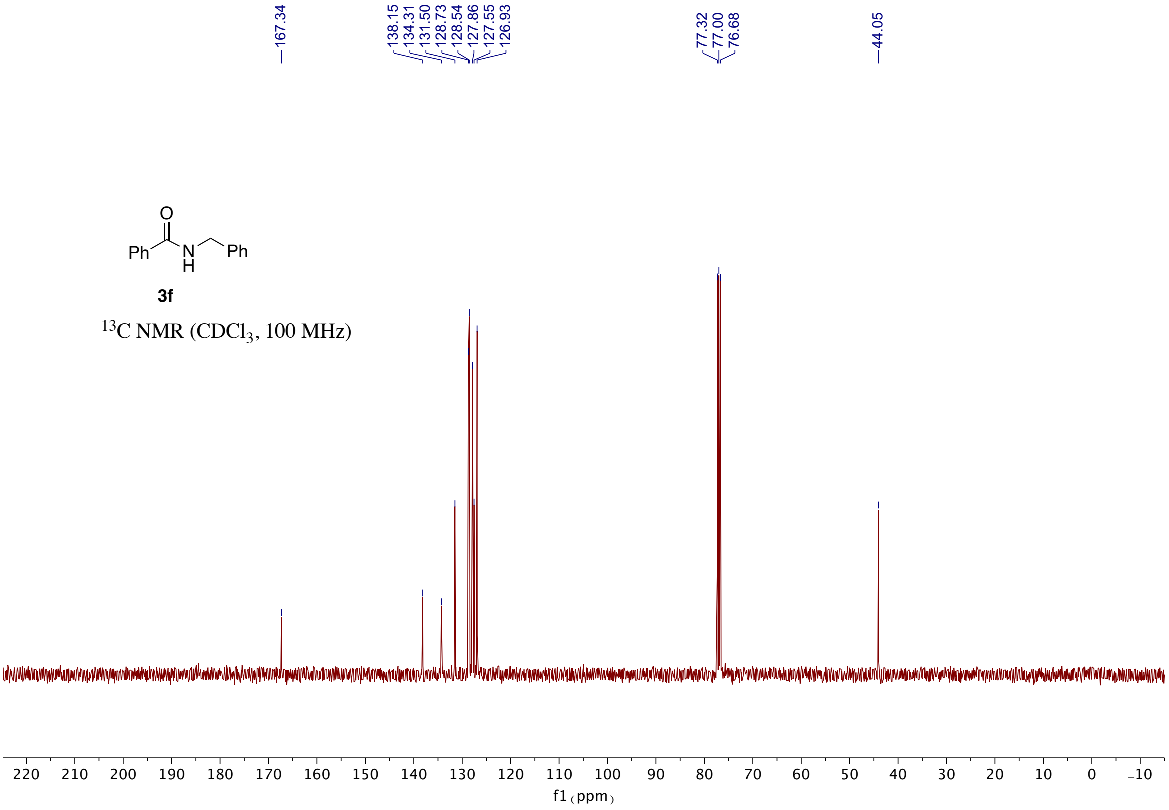
167.34
138.15
134.31
131.50
128.73
128.54
127.86
127.55
126.93
77.32
77.00
76.68
44.05
Ph
O
N
H
Ph
3f
13C NMR (CDCl3, 100 MHz)
f1 (ppm)

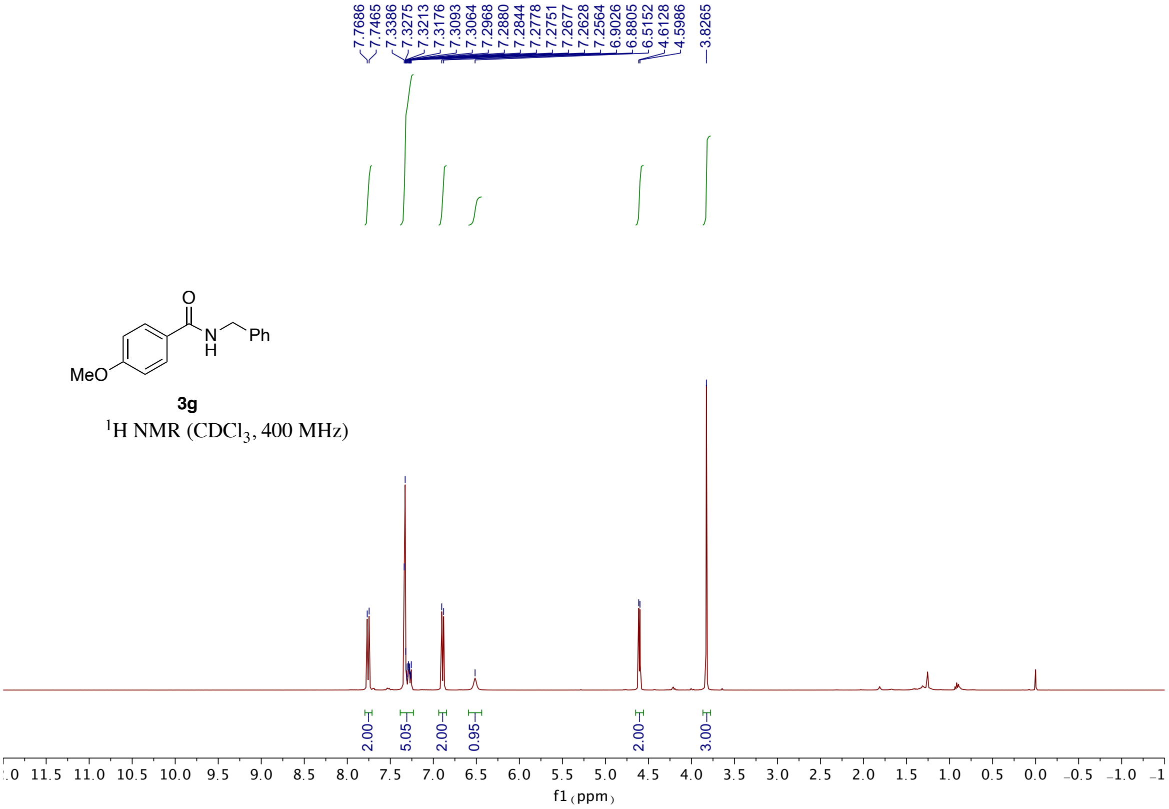
7.7686
7.7465
7.3386
7.3275
7.3213
7.3176
7.3093
7.3064
7.2968
7.2880
7.2844
7.2778
7.2751
7.2677
7.2628
7.2564
6.9026
6.8805
6.5152
4.6128
4.5986
3.8265
O
N
H
Ph
MeO
3g
^{1}H NMR ($CDCl_3$, 400 MHz)
2.00
5.05
2.00
0.95
2.00
3.00
f1 (ppm)

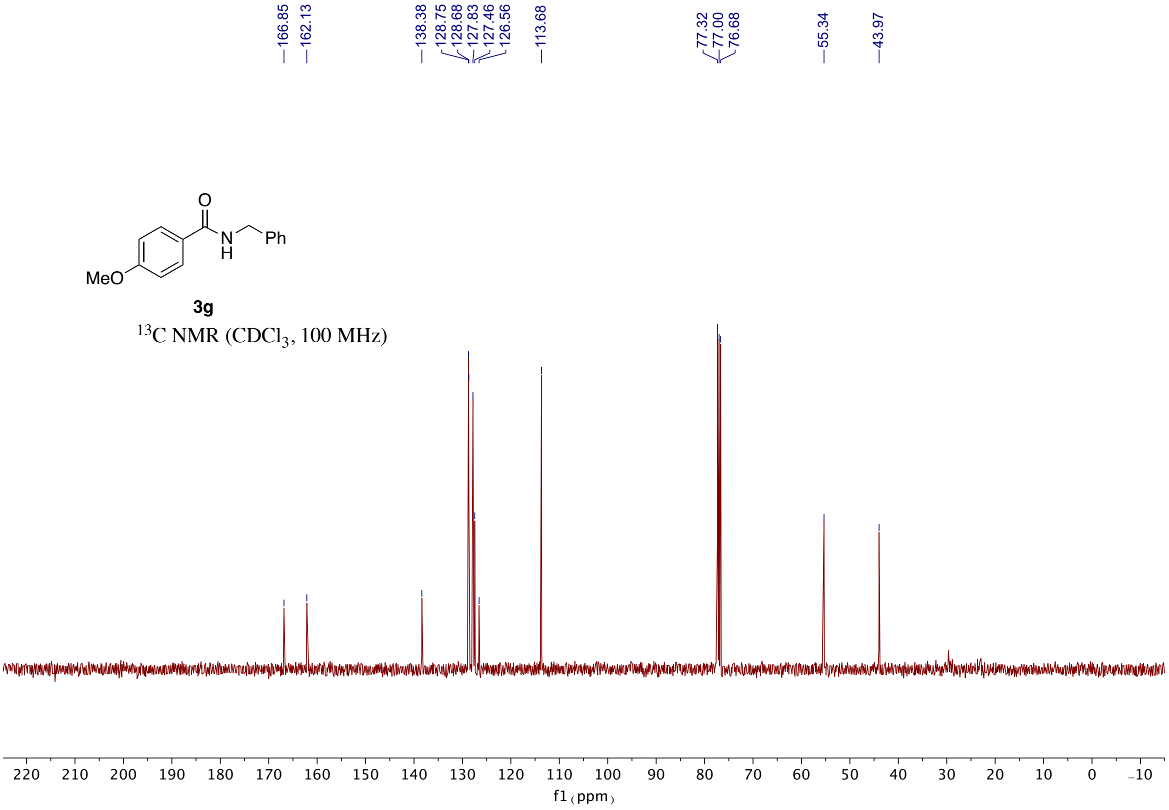
166.85
162.13
138.38
128.75
128.68
127.83
127.46
126.56
113.68
77.32
77.00
76.68
55.34
43.97
O
N
H
Ph
MeO
3g
^{13}C NMR ($CDCl_3$, 100 MHz)
f1 (ppm)

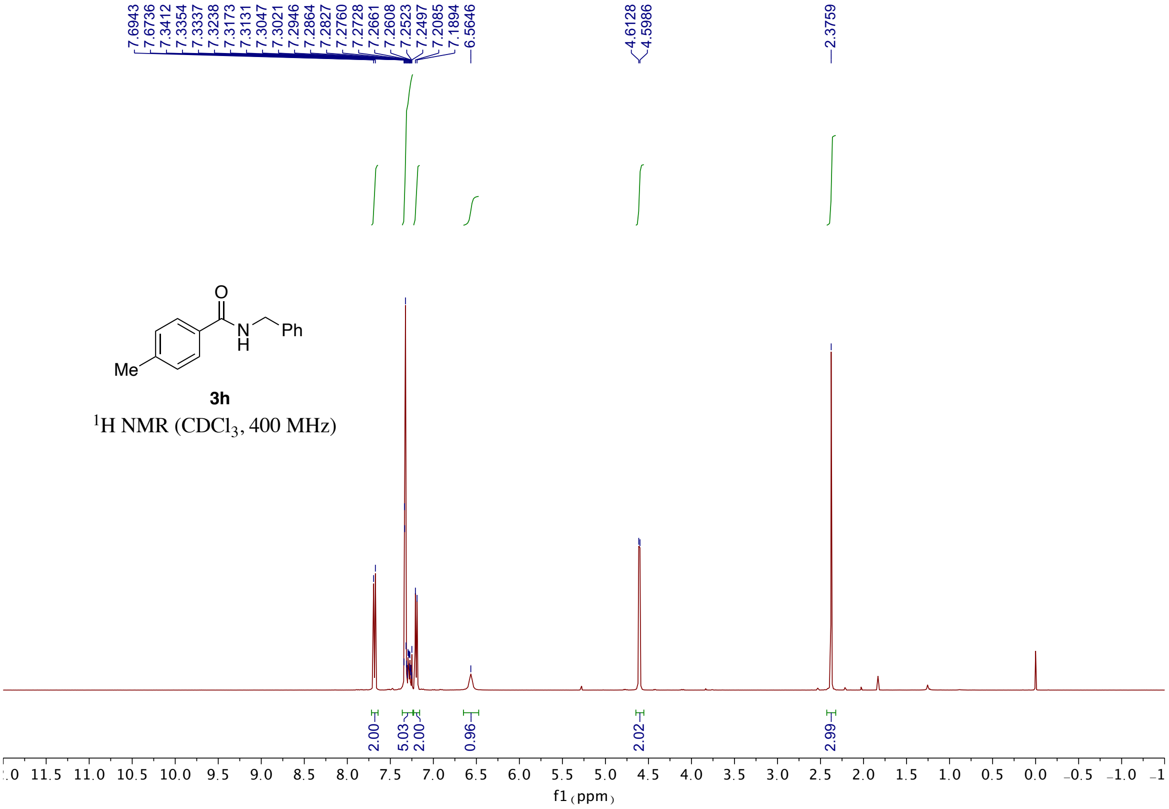

3h

^{1}H NMR ($CDCl_3$, 400 MHz)

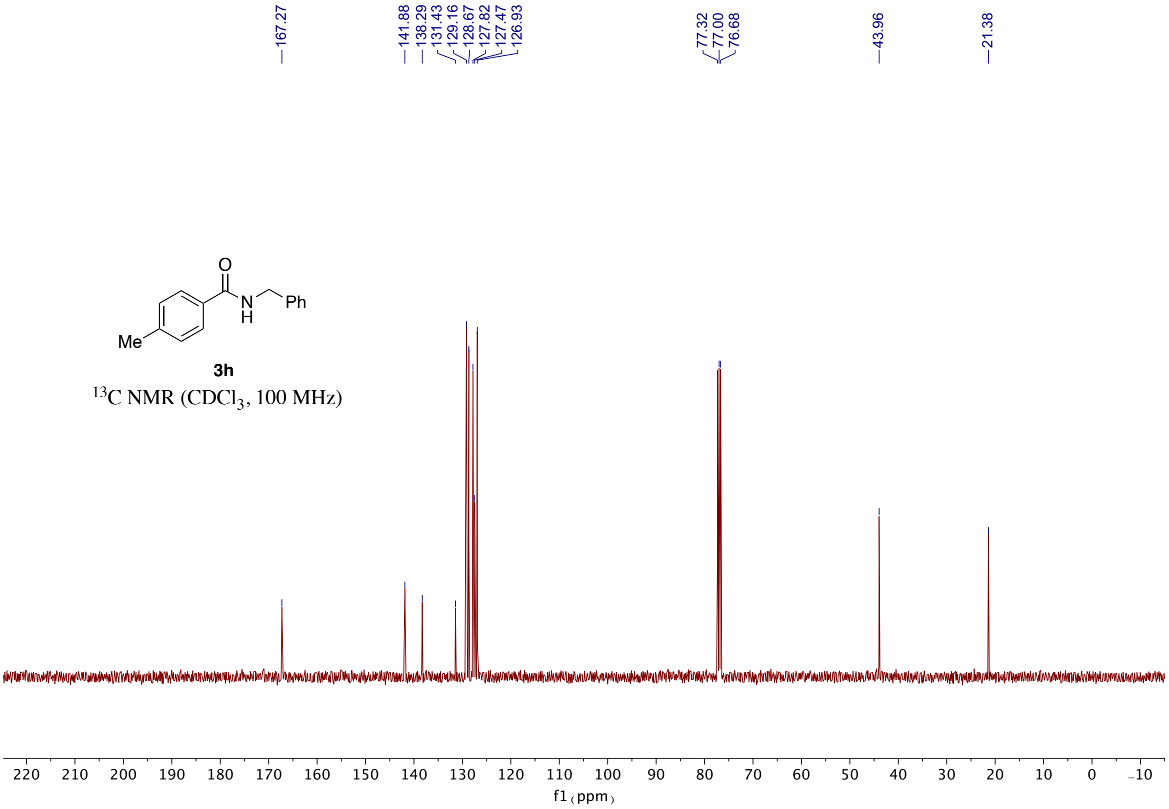

3h

^{13}C NMR ($CDCl_3$, 100 MHz)

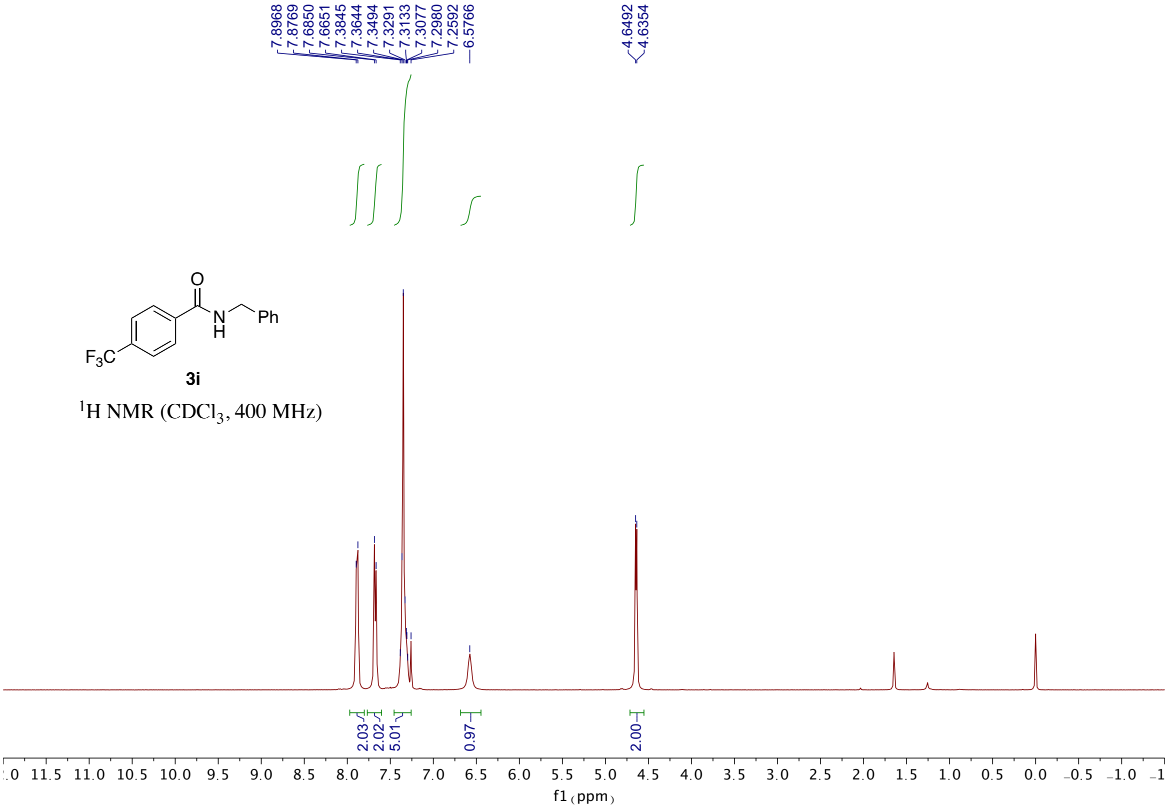

3i

^{1}H NMR ($CDCl_3$, 400 MHz)

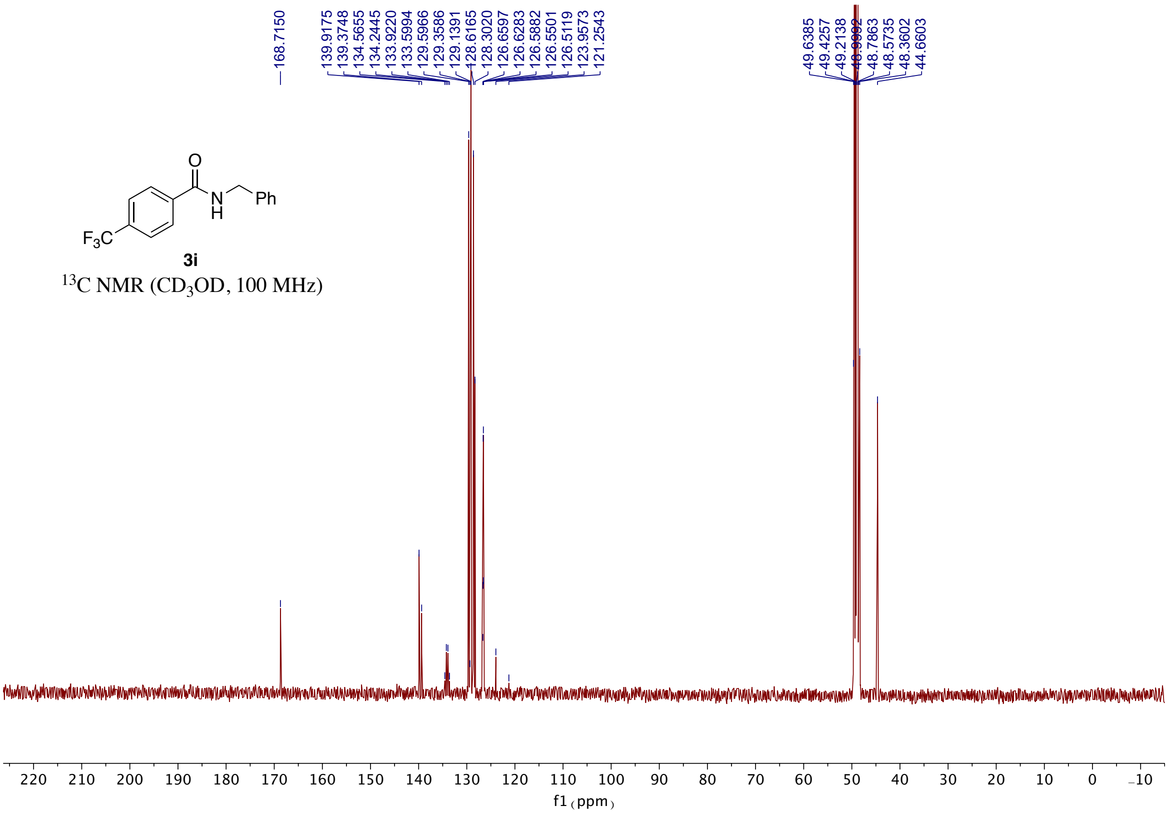

3i

^{13}C NMR (CD_3OD, 100 MHz)

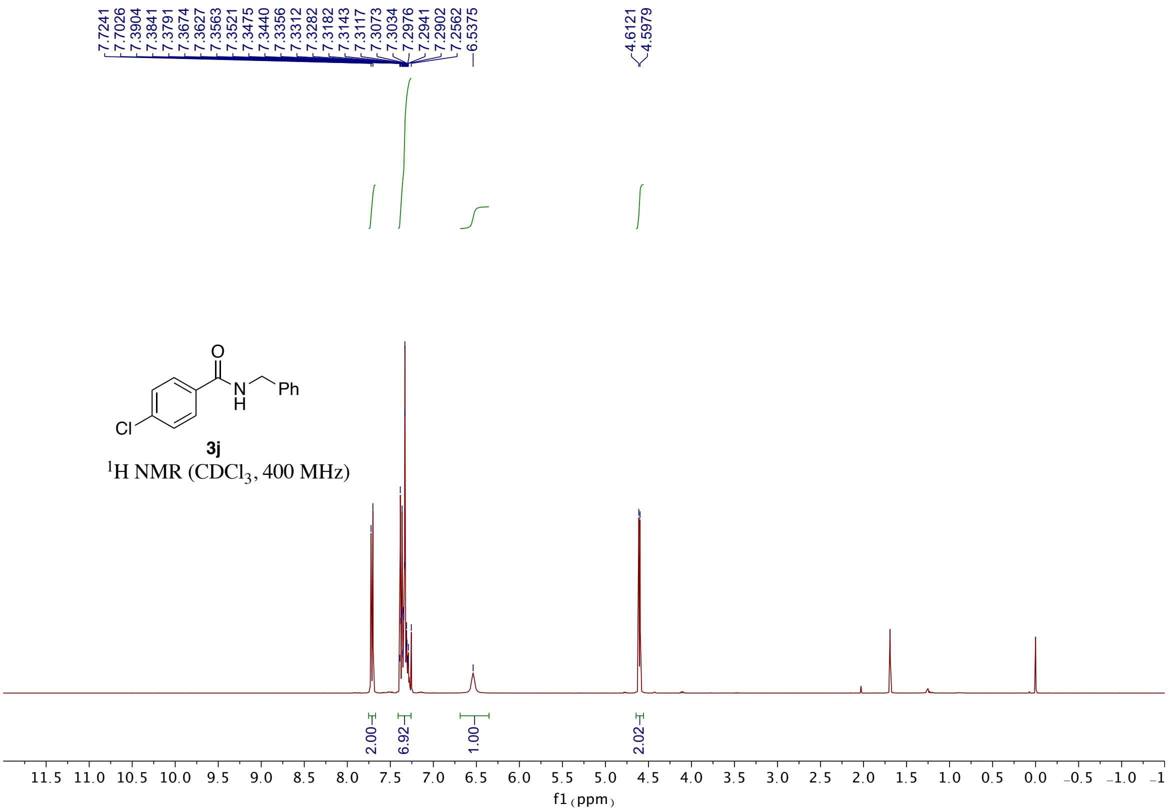
7.7241
7.7026
7.3904
7.3841
7.3791
7.3674
7.3627
7.3563
7.3521
7.3475
7.3440
7.3356
7.3312
7.3282
7.3182
7.3143
7.3117
7.3073
7.3034
7.2976
7.2941
7.2902
7.2562
6.5375
4.6121
4.5979
O
N
H
Ph
Cl
3j
1H NMR (CDCl3, 400 MHz)
2.00
6.92
1.00
2.02
f1 (ppm)

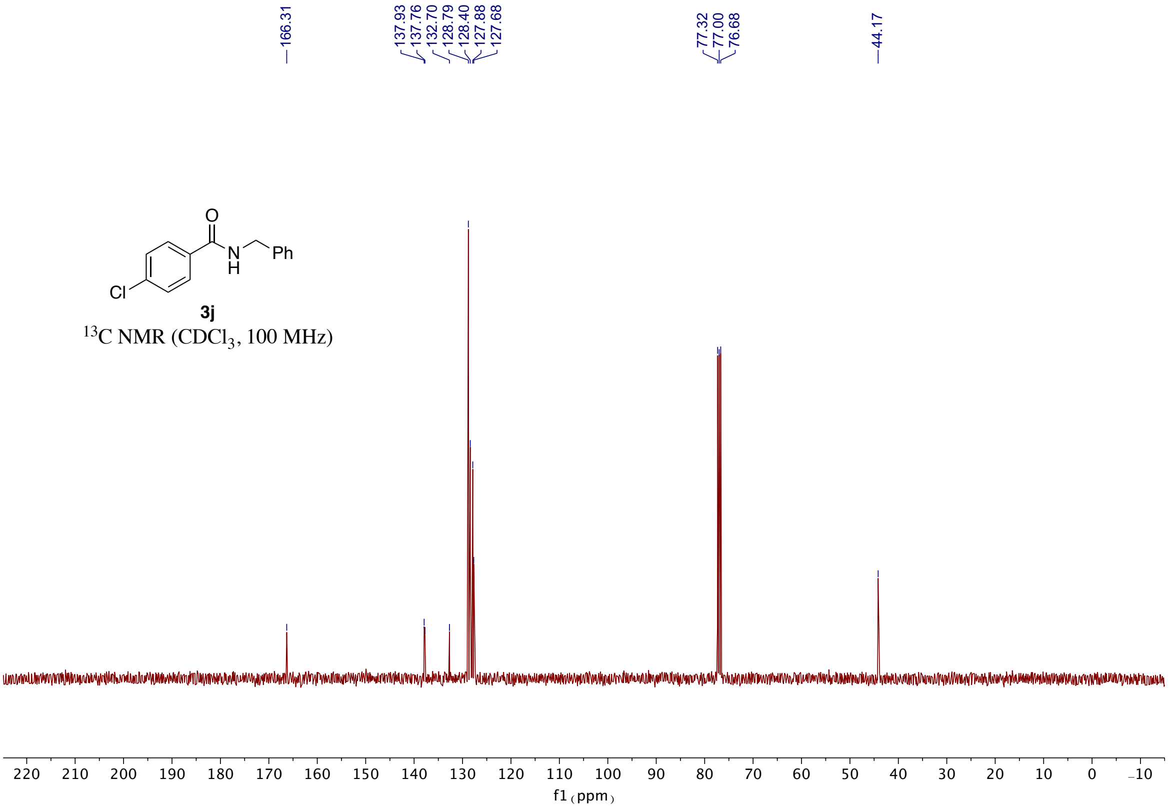
166.31
137.93
137.76
132.70
128.79
128.40
127.88
127.68
77.32
77.00
76.68
44.17
O
N
H
Ph
Cl
3j
13C NMR (CDCl3, 100 MHz)
f1 (ppm)

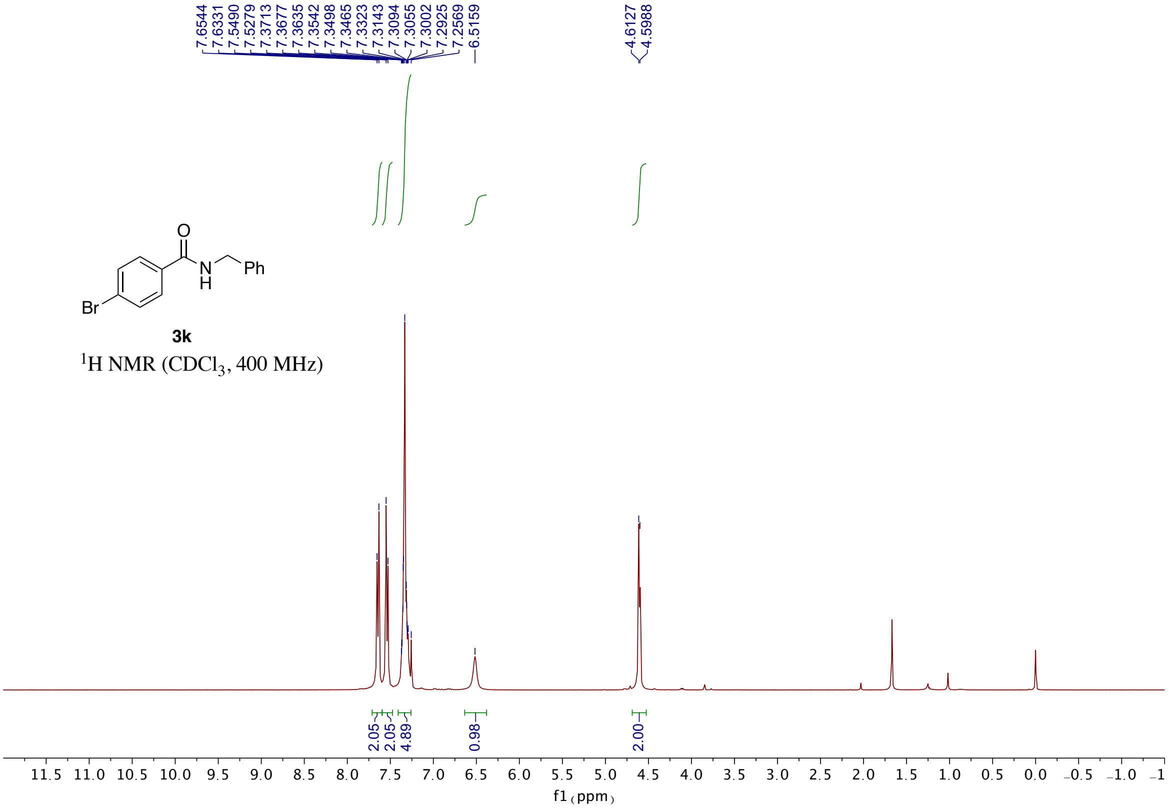

3k

^{1}H NMR ($CDCl_3$, 400 MHz)

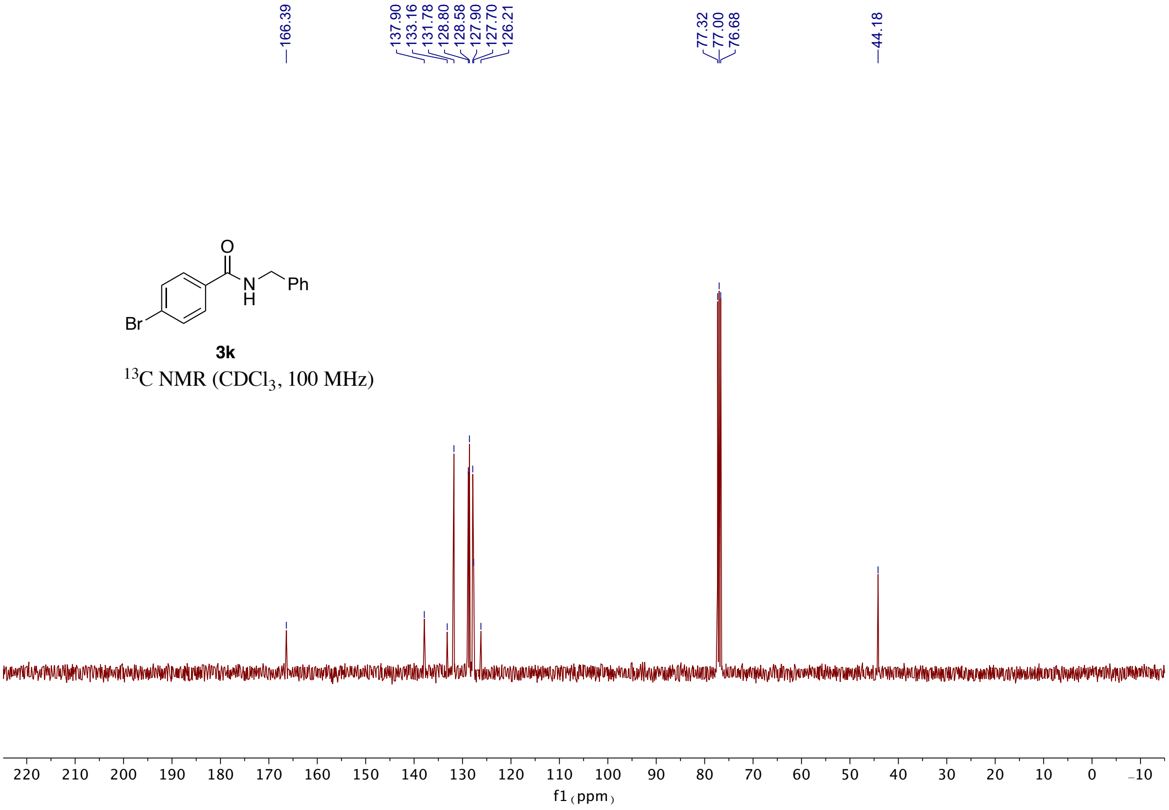

3k

^{13}C NMR ($CDCl_3$, 100 MHz)

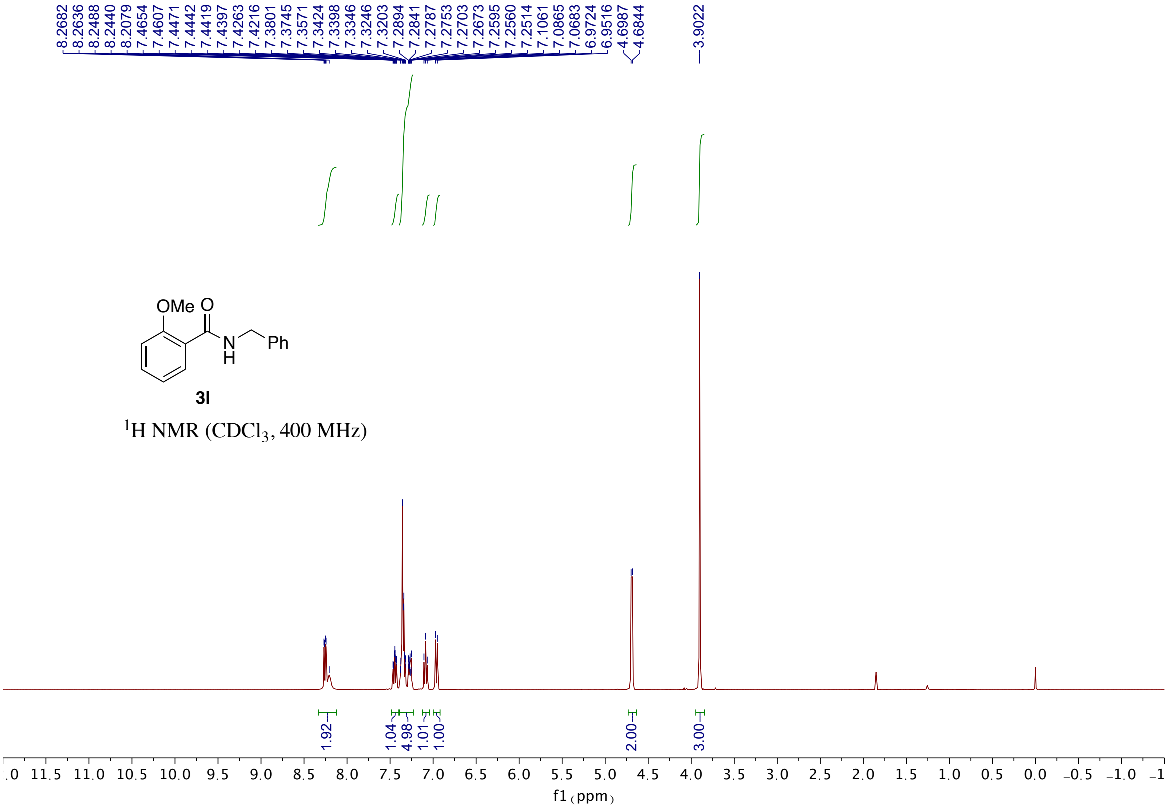

^{1}H NMR ($CDCl_3$, 400 MHz)

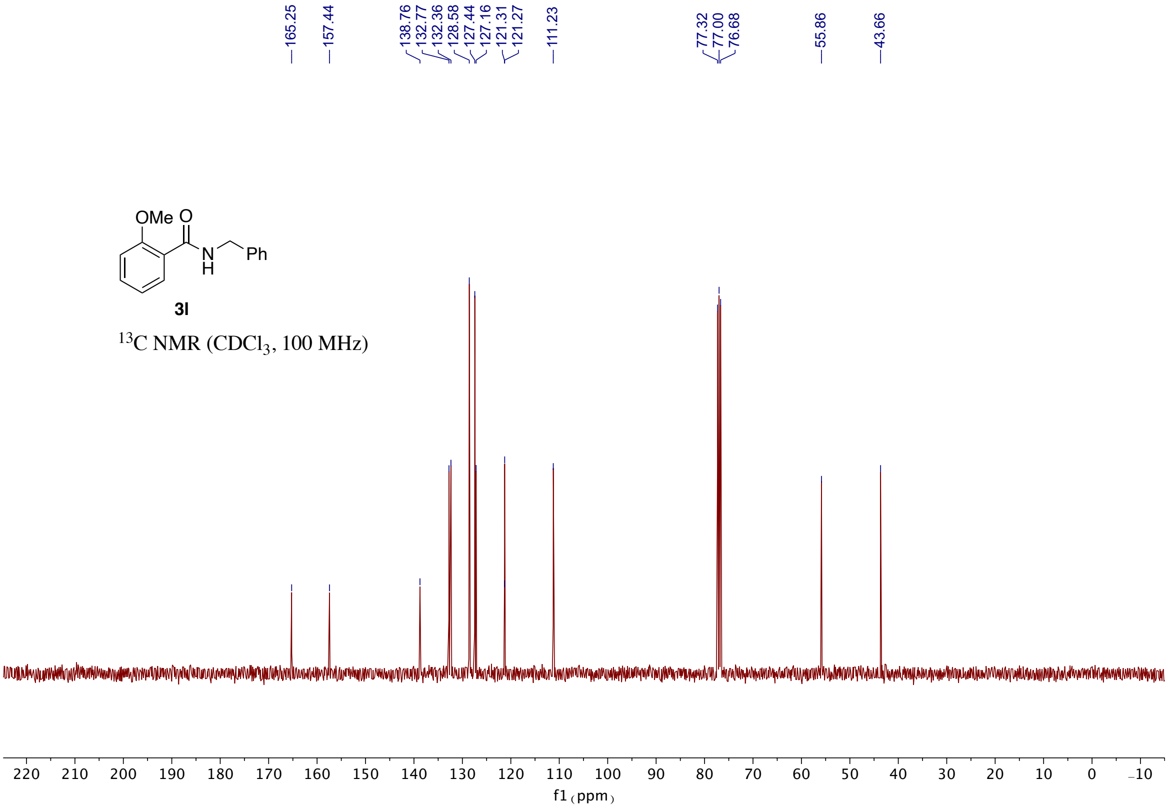

^{13}C NMR ($CDCl_3$, 100 MHz)

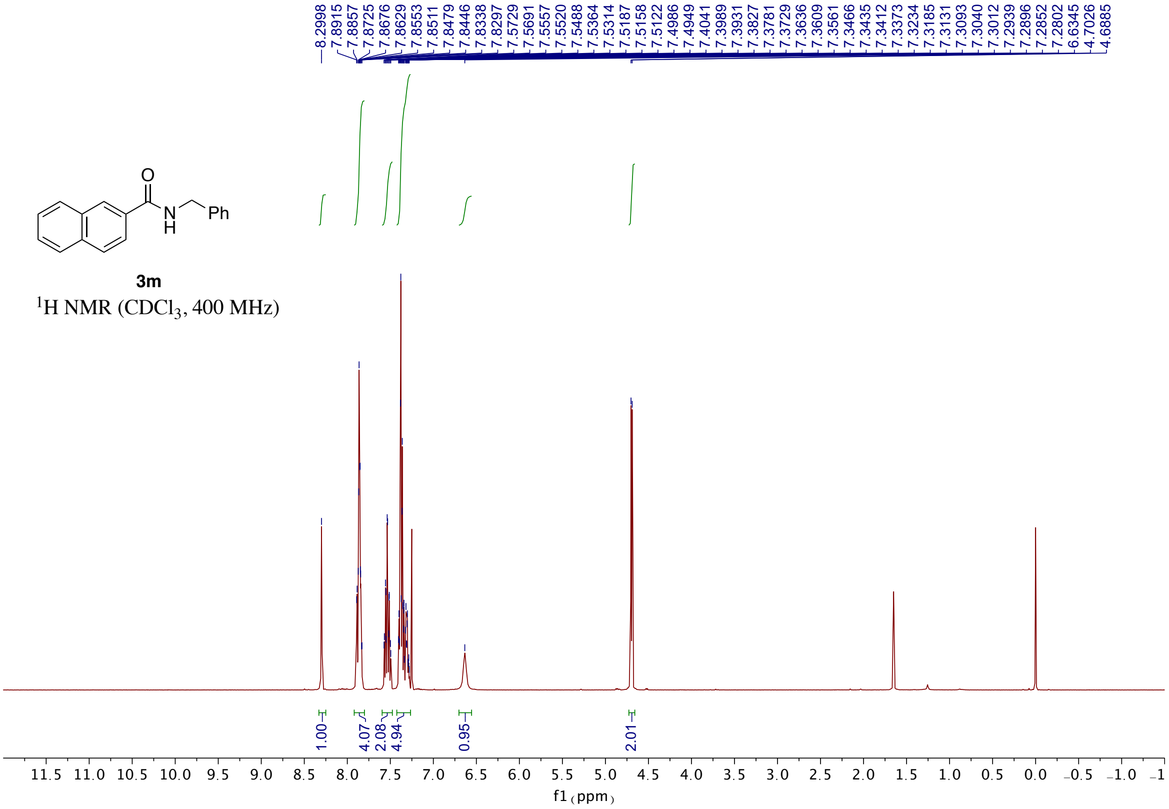

3m

^{1}H NMR ($CDCl_3$, 400 MHz)

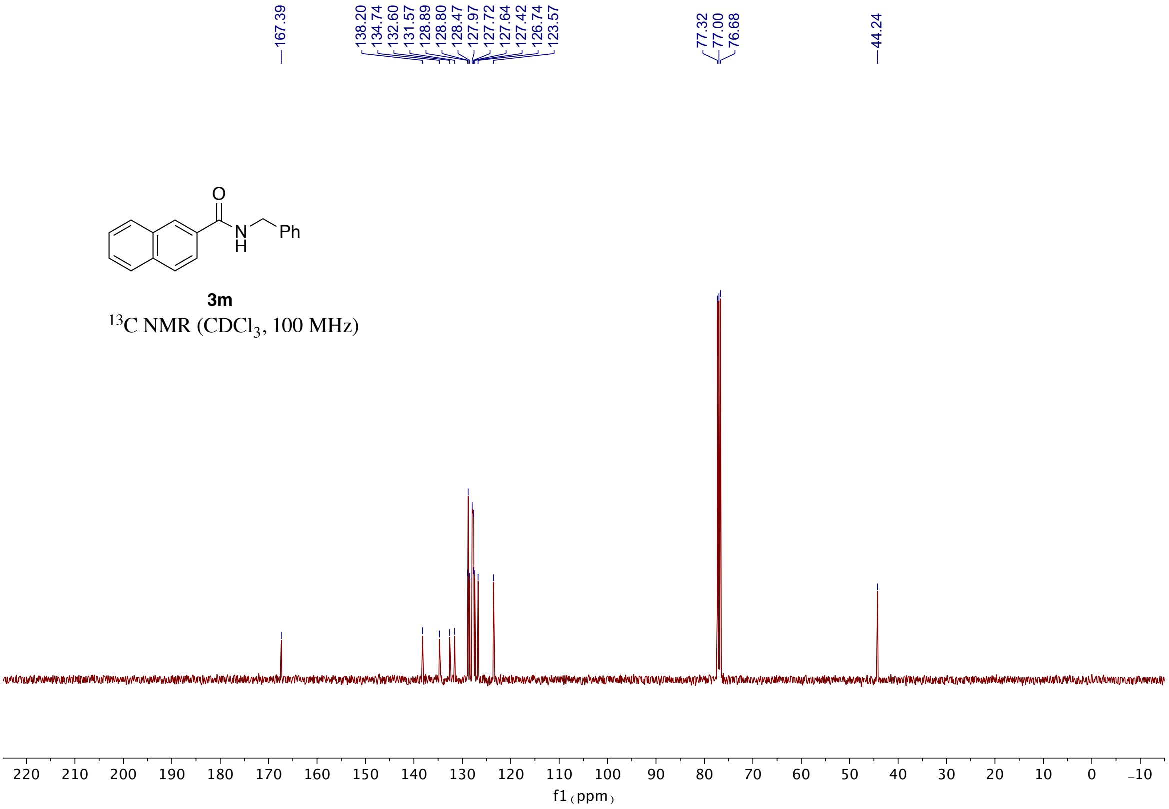

3m

^{13}C NMR ($CDCl_3$, 100 MHz)

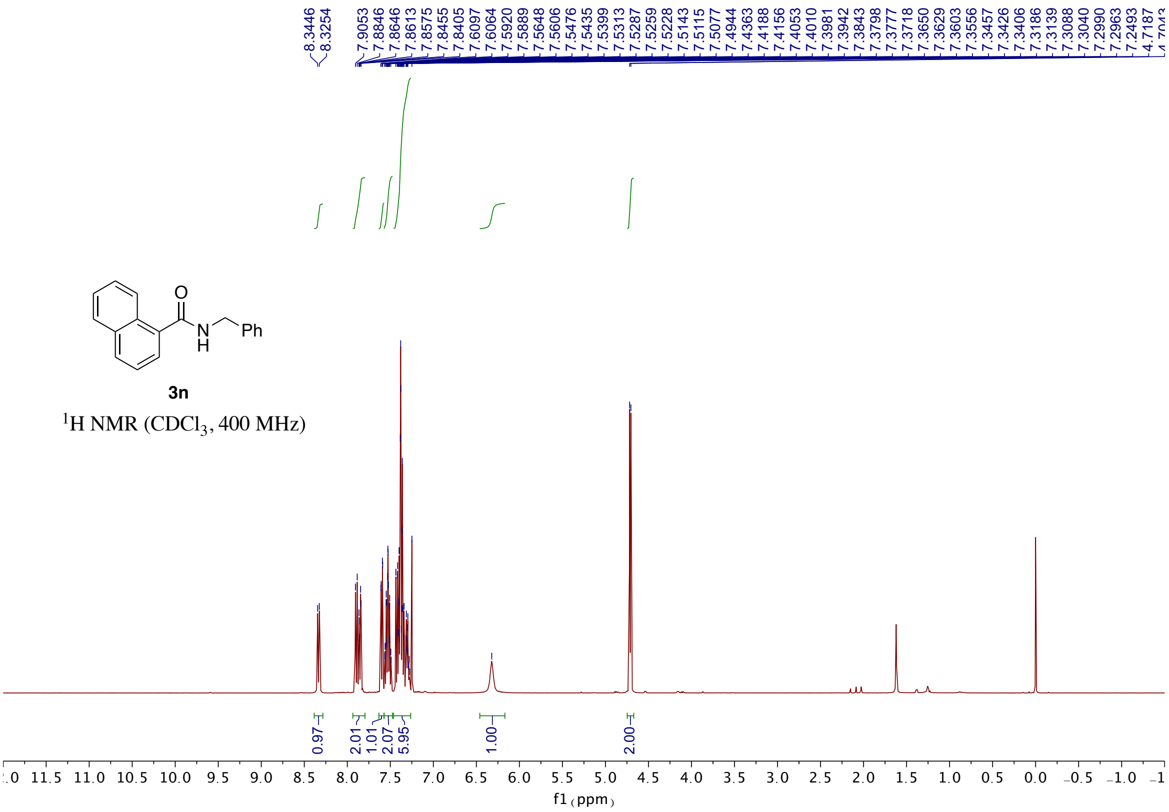
8.3446
8.3254
7.9053
7.8846
7.8646
7.8613
7.8575
7.8455
7.8405
7.6097
7.6064
7.5920
7.5889
7.5648
7.5606
7.5476
7.5435
7.5399
7.5313
7.5287
7.5259
7.5228
7.5143
7.5115
7.5077
7.4944
7.4363
7.4188
7.4156
7.4053
7.4010
7.3981
7.3942
7.3843
7.3798
7.3777
7.3718
7.3650
7.3629
7.3603
7.3556
7.3457
7.3426
7.3406
7.3186
7.3139
7.3088
7.3040
7.2990
7.2963
7.2493
4.7187
O
N
H
Ph
3n
1H NMR (CDCl3, 400 MHz)
0.97
2.01
1.01
2.07
5.95
1.00
2.00
f1 (ppm)

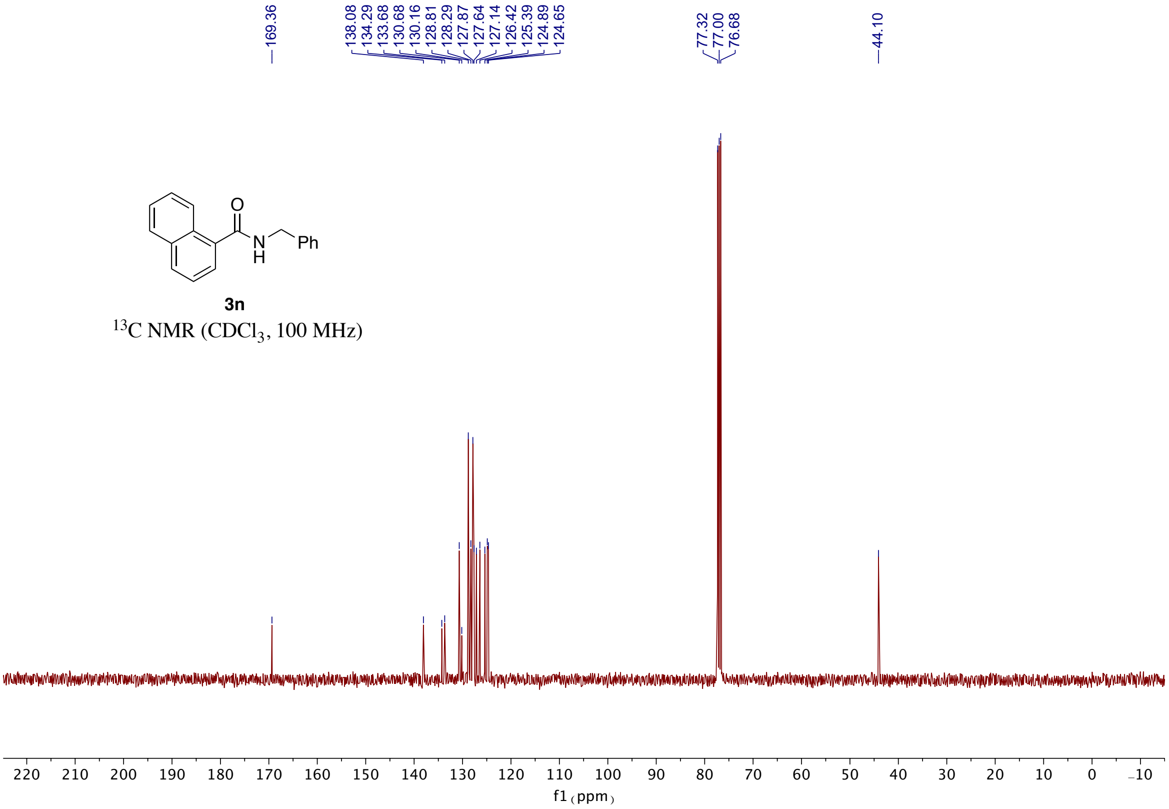
169.36
138.08
134.29
133.68
130.68
130.16
128.81
128.29
127.87
127.64
127.14
126.42
125.39
124.89
124.65
77.32
77.00
76.68
44.10
O
N
H
Ph
3n
13C NMR (CDCl3, 100 MHz)
f1 (ppm)

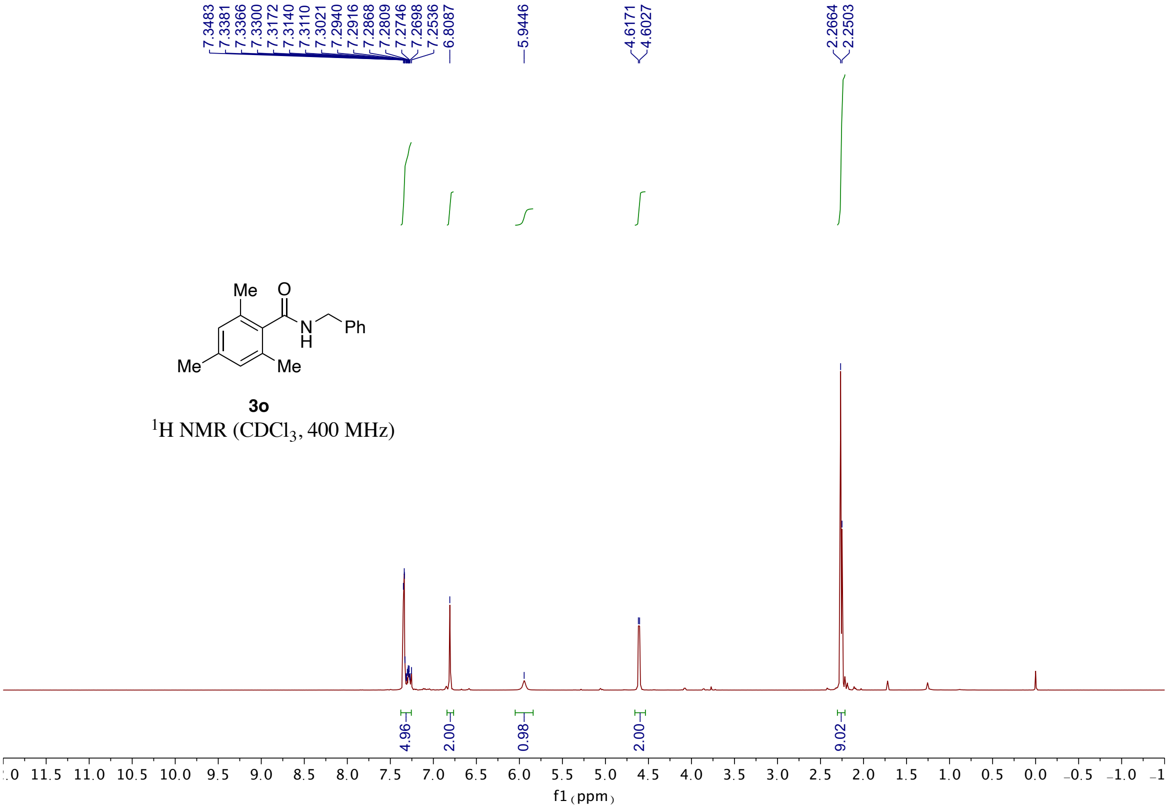

3o
^{1}H NMR ($CDCl_3$, 400 MHz)

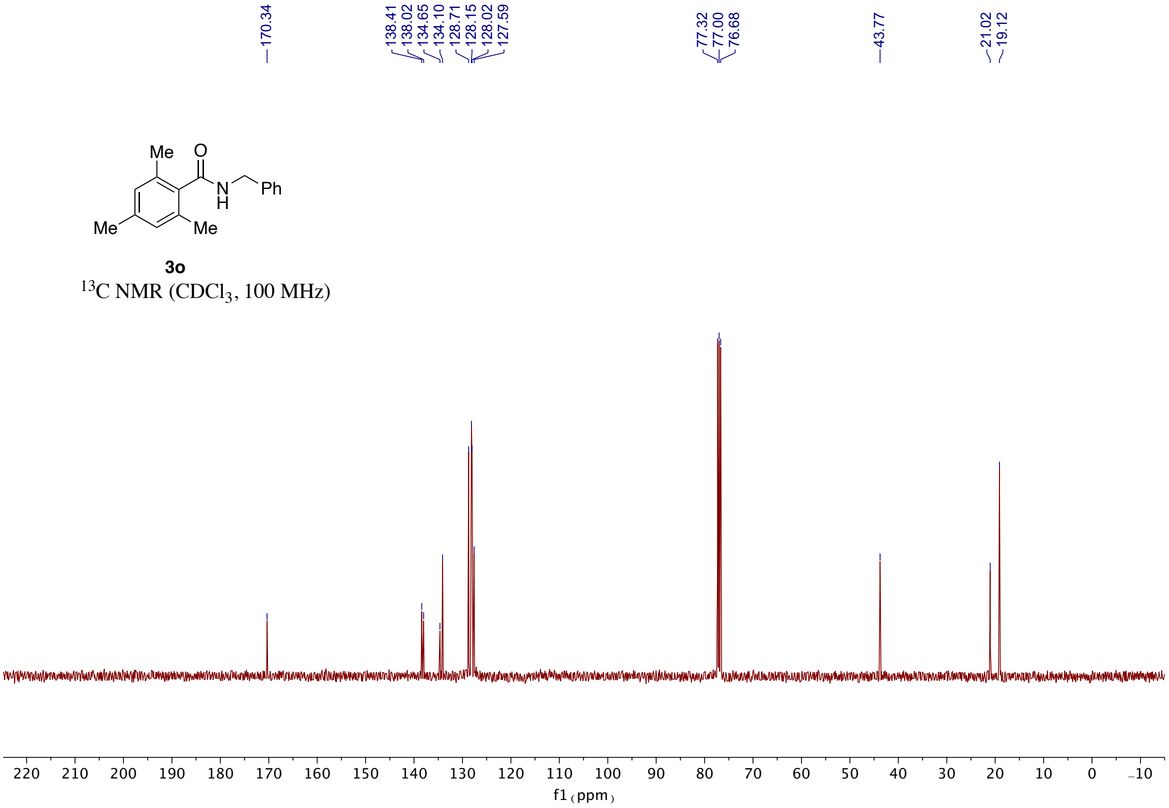

3o
^{13}C NMR ($CDCl_3$, 100 MHz)

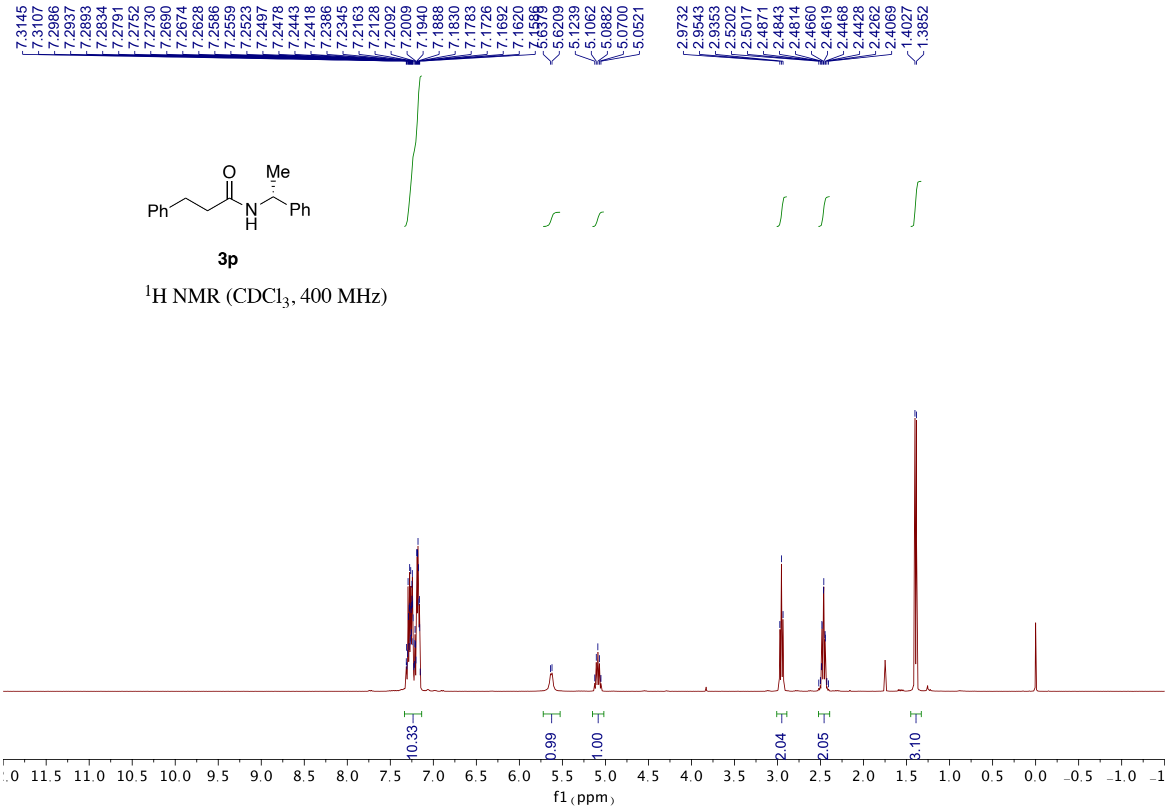

3p

^{1}H NMR ($CDCl_3$, 400 MHz)

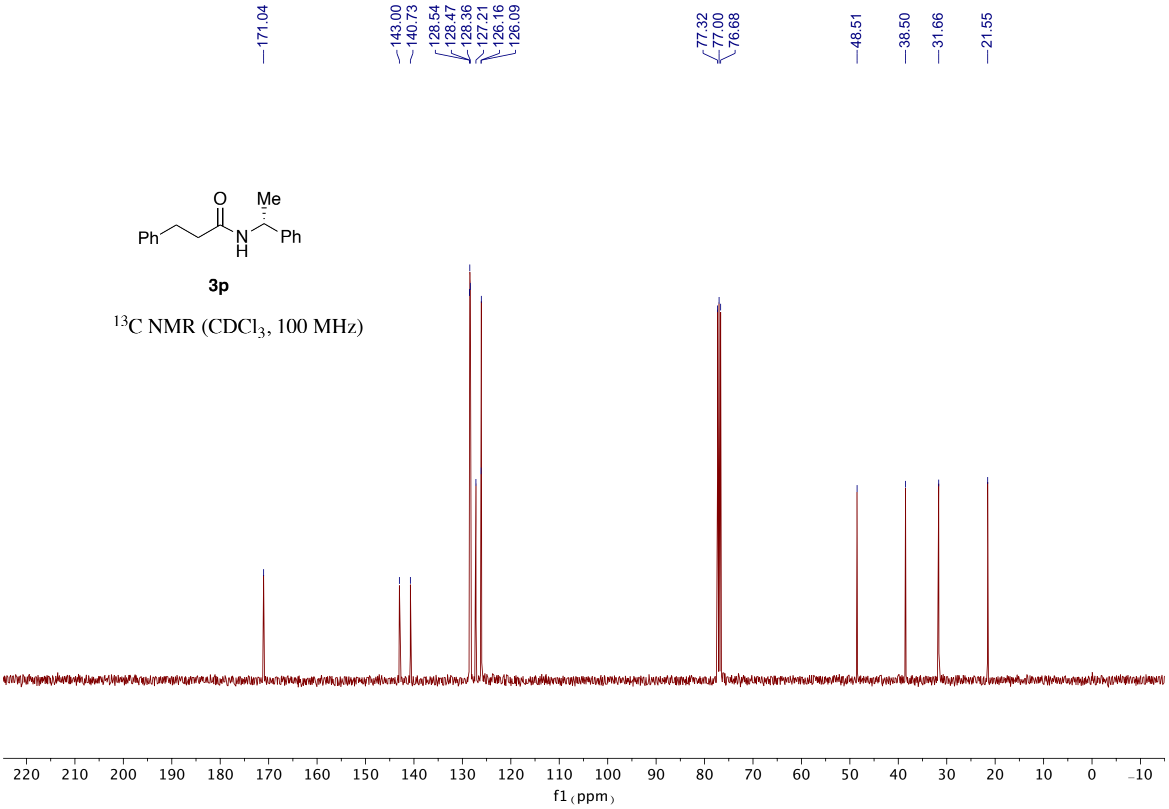

3p

^{13}C NMR ($CDCl_3$, 100 MHz)

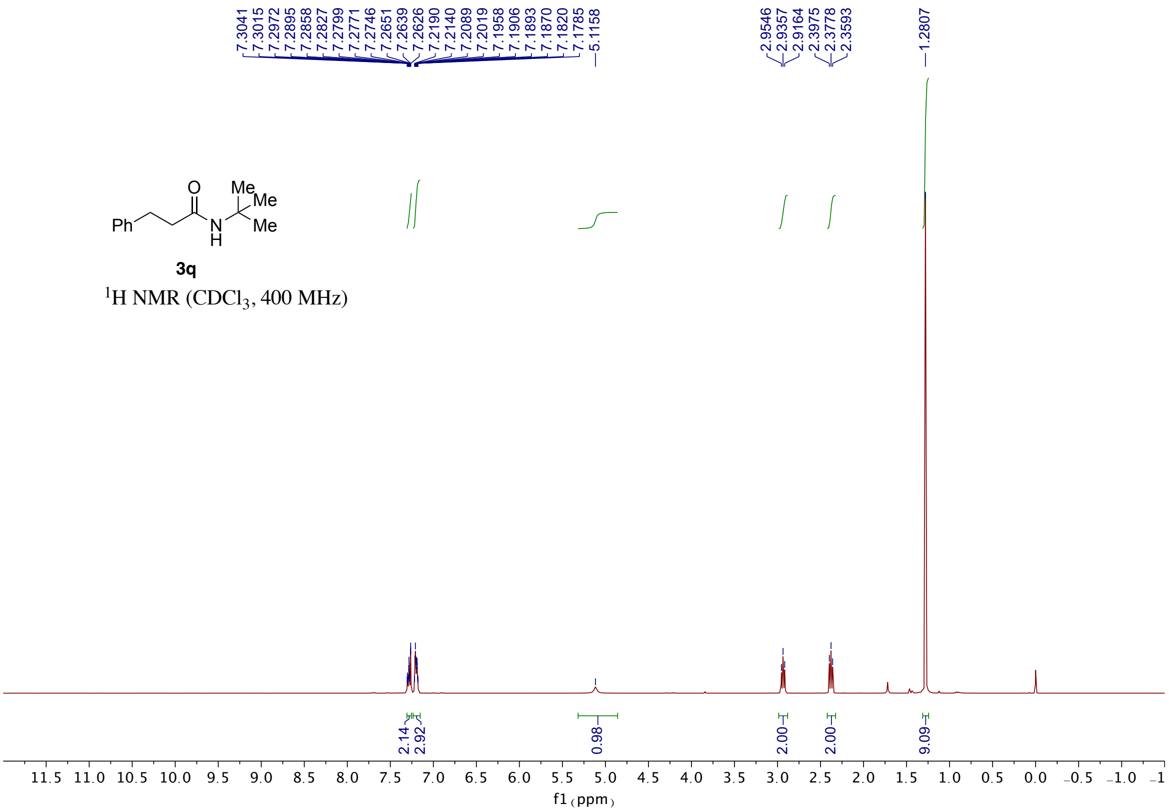
7.3041
7.3015
7.2972
7.2895
7.2858
7.2827
7.2799
7.2771
7.2746
7.2651
7.2639
7.2626
7.2190
7.2140
7.2089
7.2019
7.1958
7.1906
7.1893
7.1870
7.1820
7.1785
5.1158
2.9546
2.9357
2.9164
2.3975
2.3778
2.3593
1.2807
Ph
O
N
H
Me
Me
Me
3q
1H NMR (CDCl3, 400 MHz)
2.14
2.92
0.98
2.00
2.00
9.09
f1 (ppm)

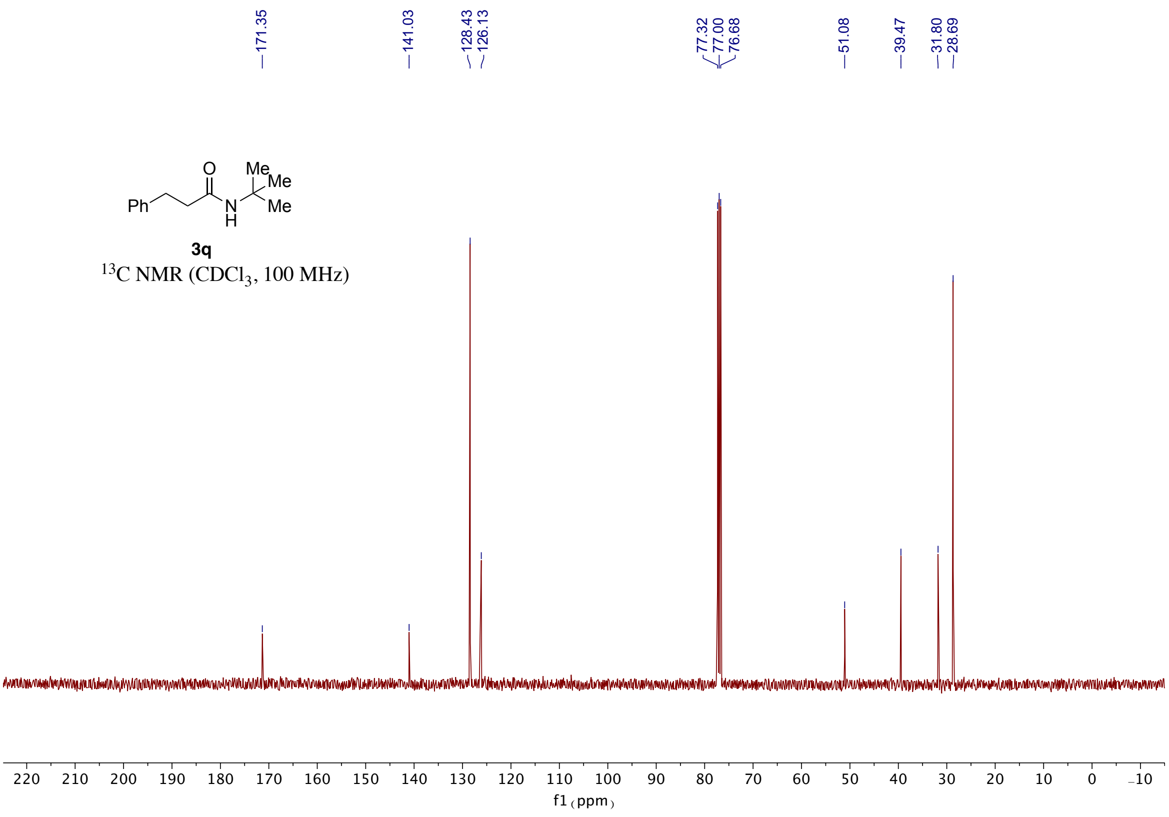
171.35
141.03
128.43
126.13
77.32
77.00
76.68
51.08
39.47
31.80
28.69
Ph
O
N
H
Me
Me
Me
3q
13C NMR (CDCl3, 100 MHz)
f1 (ppm)

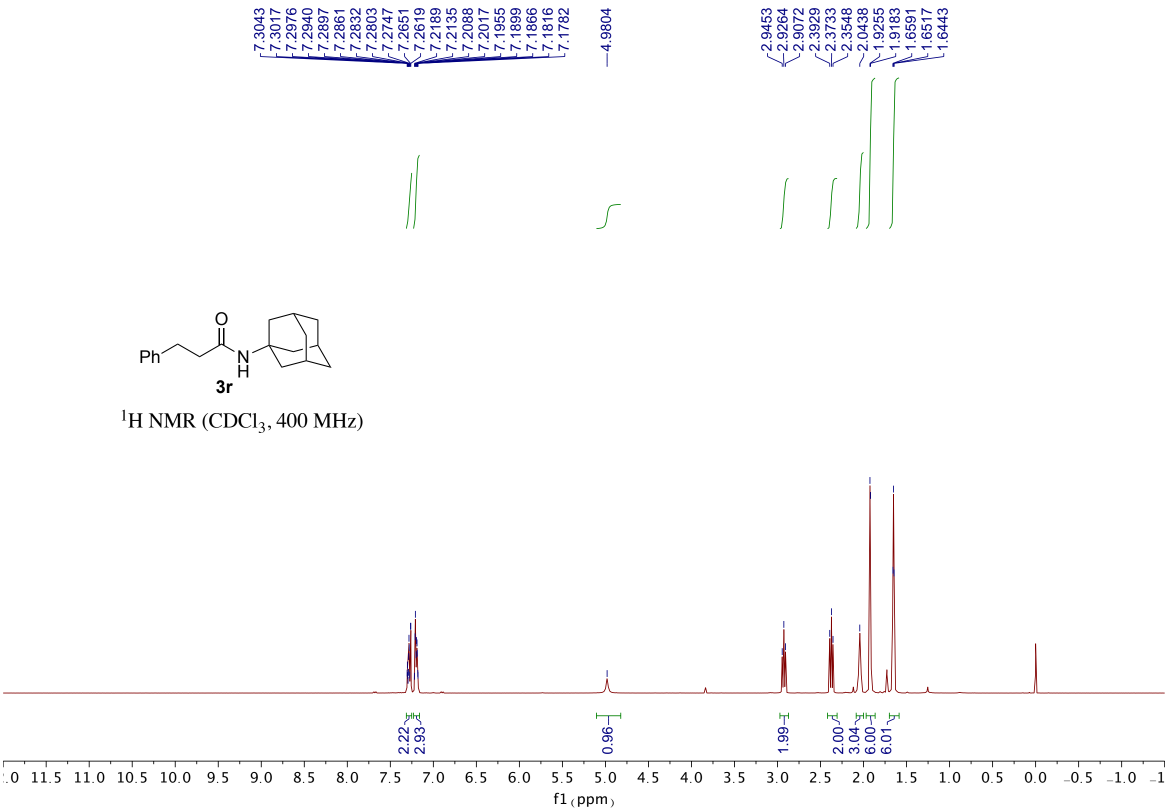
7.3043
7.3017
7.2976
7.2940
7.2897
7.2861
7.2832
7.2803
7.2747
7.2651
7.2619
7.2189
7.2135
7.2088
7.2017
7.1955
7.1899
7.1866
7.1816
7.1782
4.9804
2.9453
2.9264
2.9072
2.3929
2.3733
2.3548
2.0438
1.9255
1.9183
1.6591
1.6517
1.6443
Ph
O
N
H
3r
1H NMR (CDCl3, 400 MHz)
2.22
2.93
0.96
1.99
2.00
3.04
6.00
6.01
f1 (ppm)

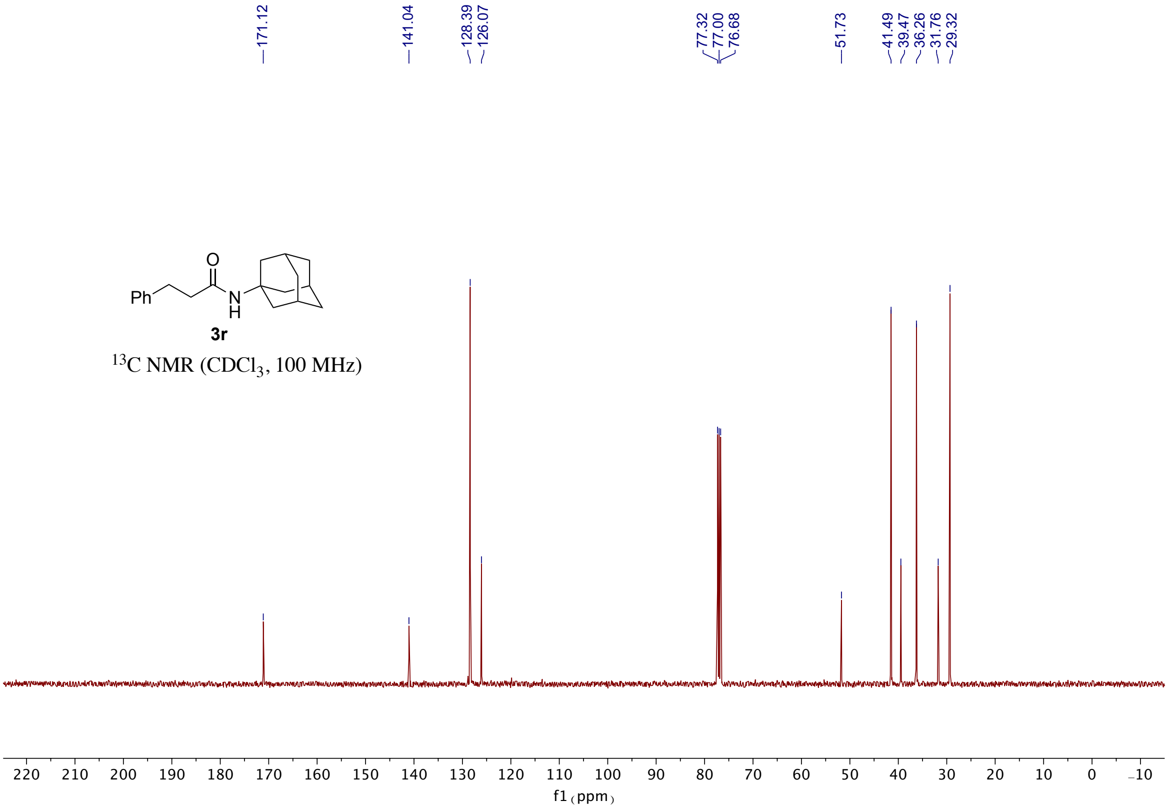
171.12
141.04
128.39
126.07
77.32
77.00
76.68
51.73
41.49
39.47
36.26
31.76
29.32
Ph
O
N
H
3r
13C NMR (CDCl3, 100 MHz)
f1 (ppm)

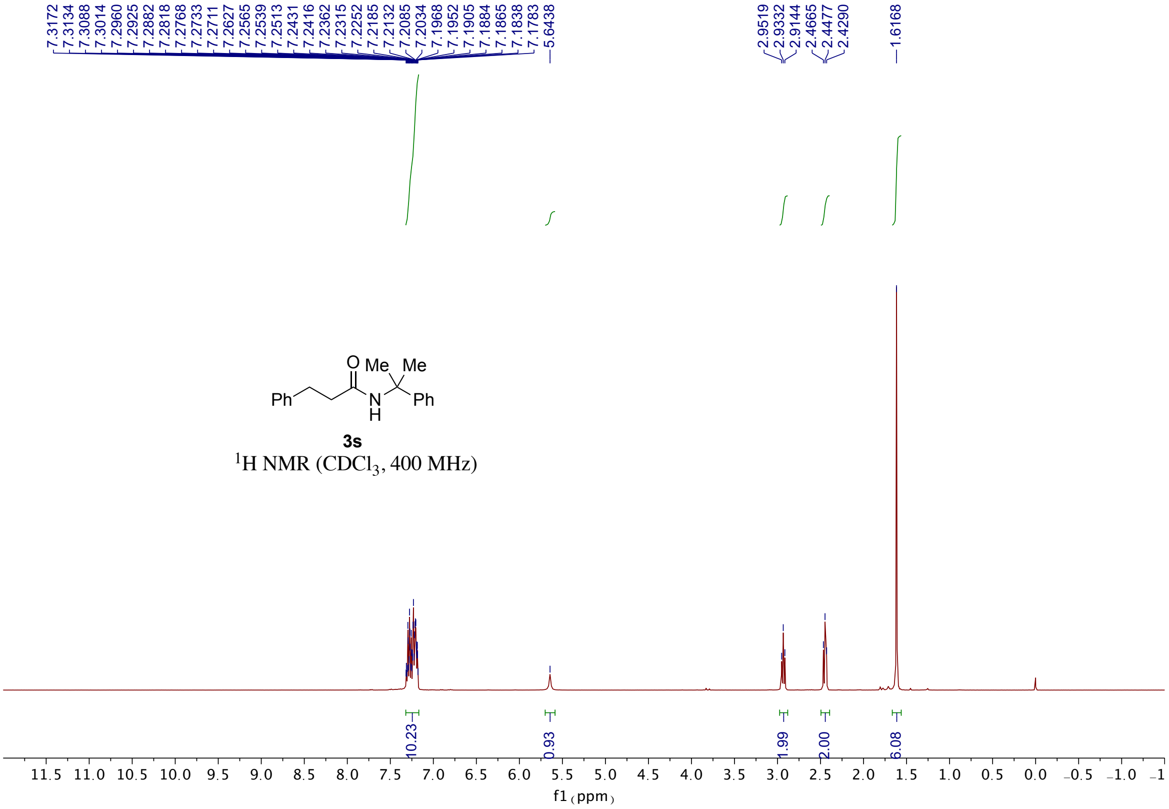
7.3172
7.3134
7.3088
7.3014
7.2960
7.2925
7.2882
7.2818
7.2768
7.2733
7.2711
7.2627
7.2565
7.2539
7.2513
7.2431
7.2416
7.2362
7.2315
7.2252
7.2185
7.2132
7.2085
7.2034
7.1968
7.1952
7.1905
7.1884
7.1865
7.1838
7.1783
5.6438
2.9519
2.9332
2.9144
2.4665
2.4477
2.4290
1.6168
O
Me
Me
Ph
N
H
Ph
3s
¹H NMR (CDCl₃, 400 MHz)
10.23
0.93
1.99
2.00
6.08
f1 (ppm)

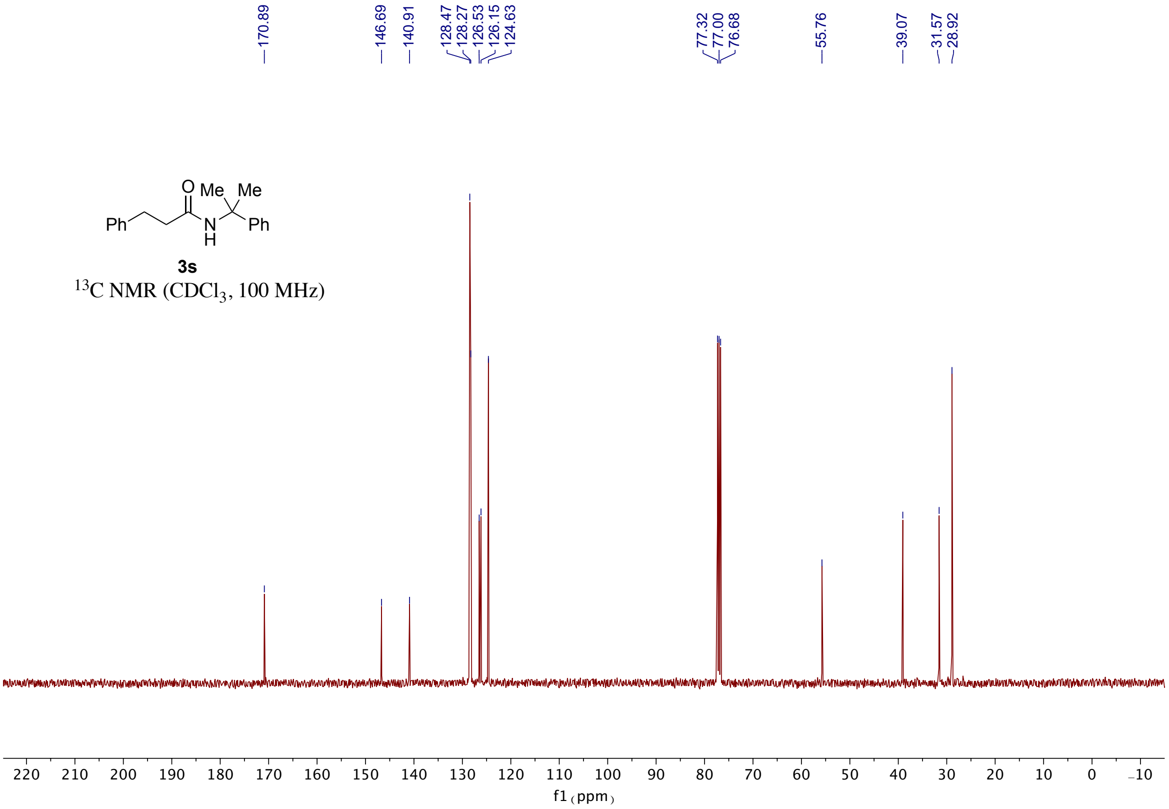
170.89
146.69
140.91
128.47
128.27
126.53
126.15
124.63
77.32
77.00
76.68
55.76
39.07
31.57
28.92
O
Me
Me
Ph
N
H
Ph
3s
¹³C NMR (CDCl₃, 100 MHz)
f1 (ppm)

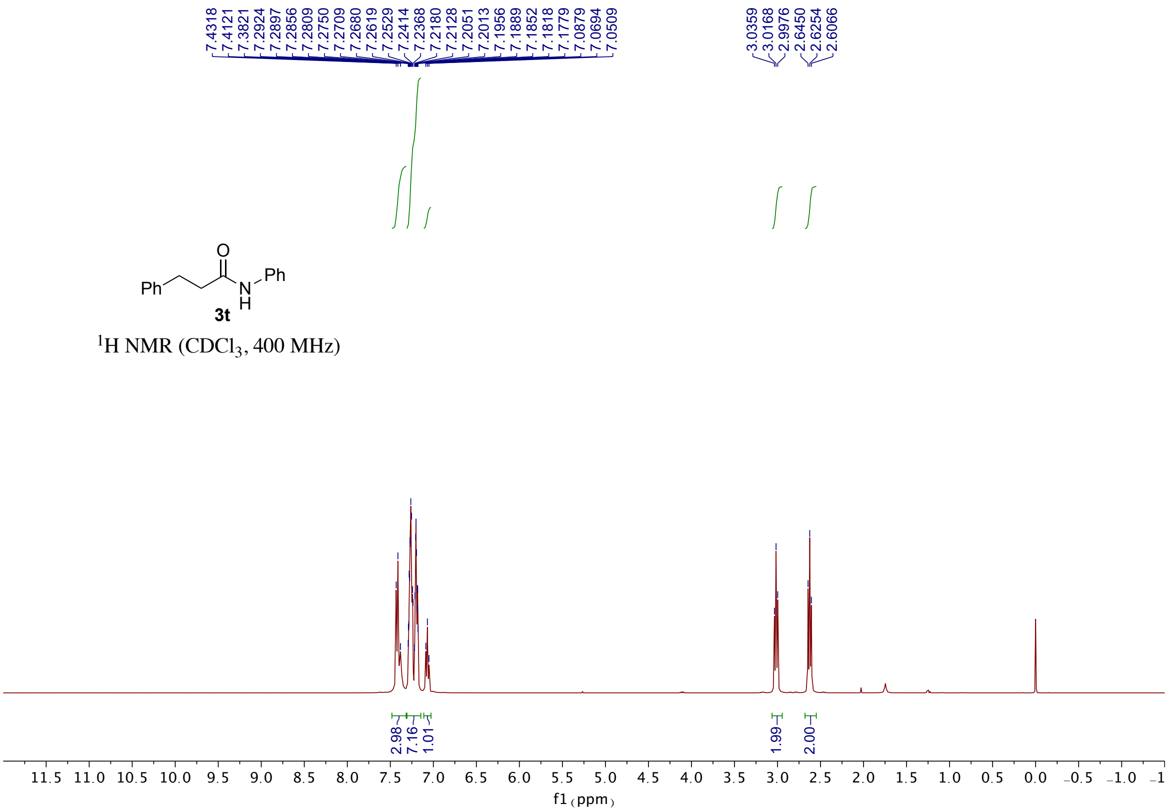

^{1}H NMR ($CDCl_3$, 400 MHz)

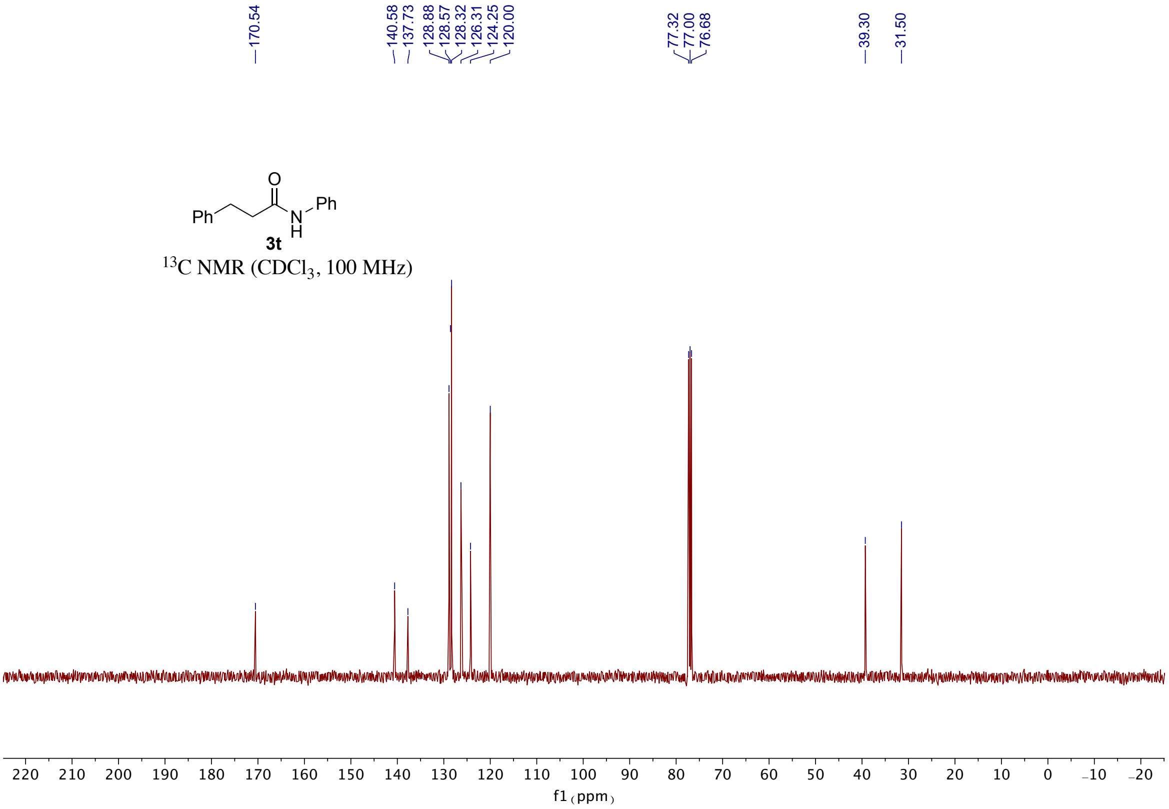

^{13}C NMR ($CDCl_3$, 100 MHz)

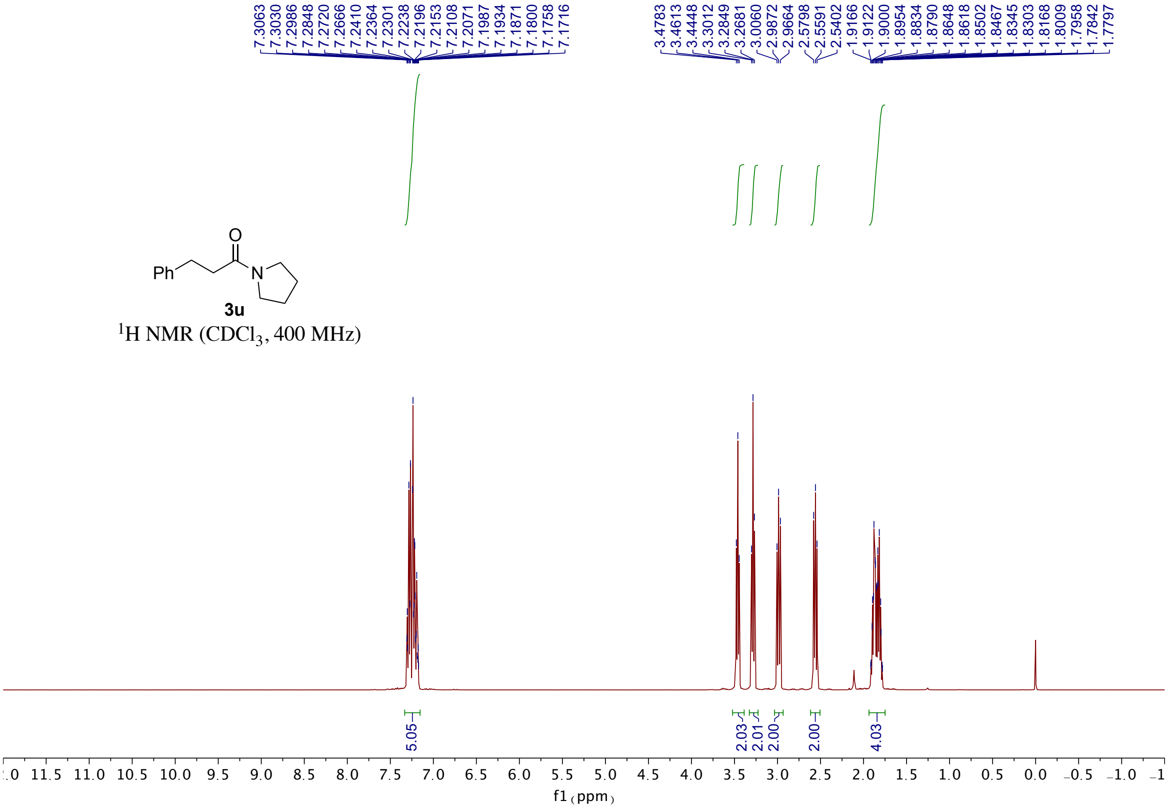

^{1}H NMR (CDCl$_3$, 400 MHz)

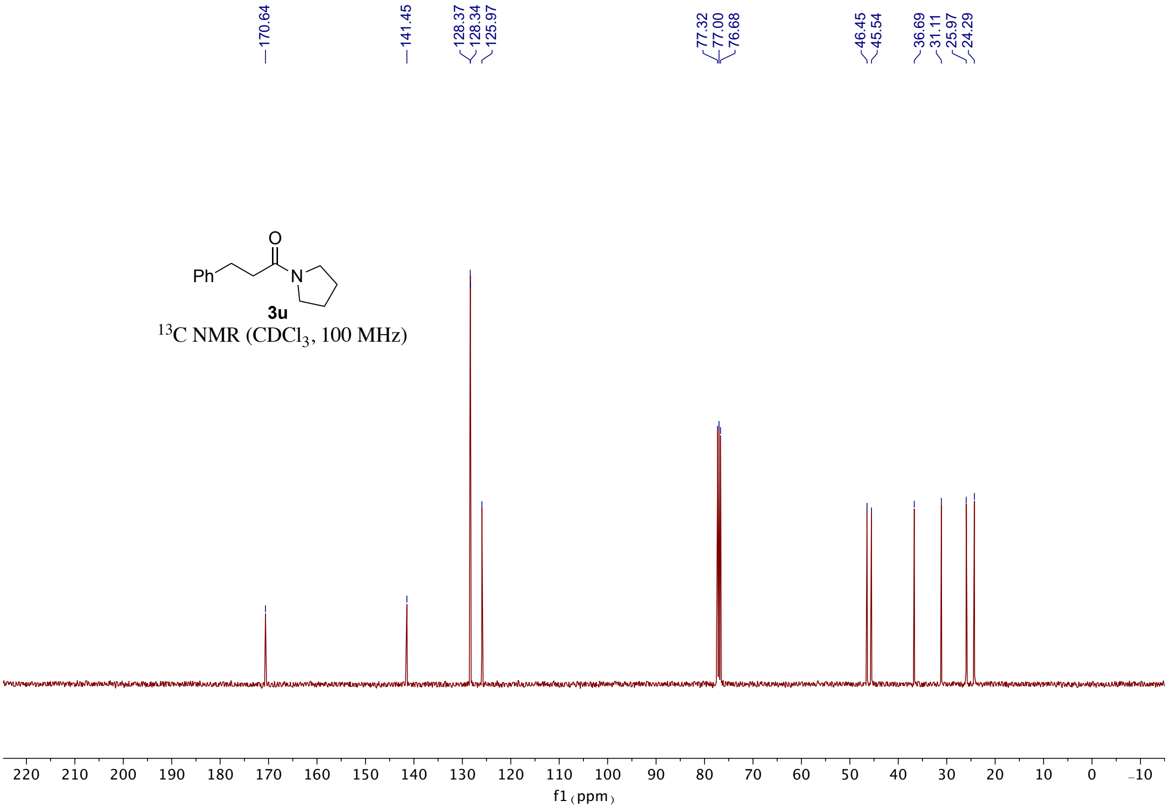

^{13}C NMR (CDCl$_3$, 100 MHz)

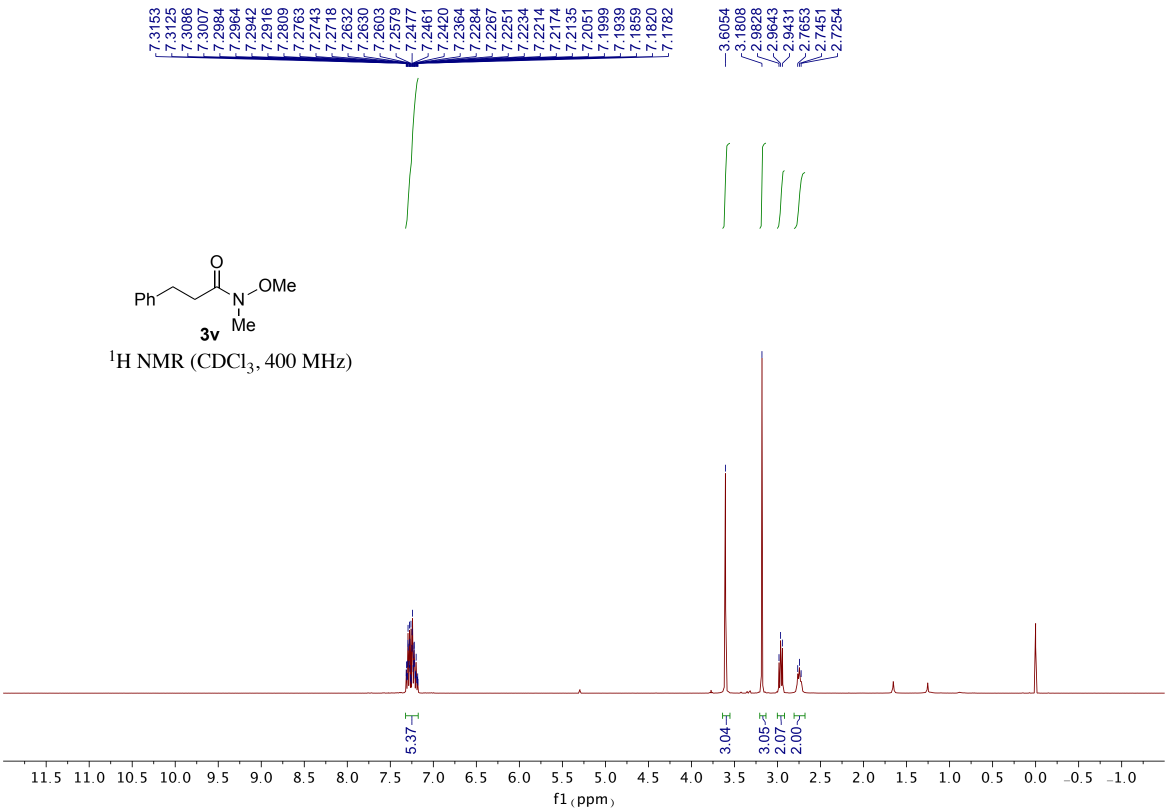
7.3153
7.3125
7.3086
7.3007
7.2984
7.2964
7.2942
7.2916
7.2809
7.2763
7.2743
7.2718
7.2632
7.2630
7.2603
7.2579
7.2477
7.2461
7.2420
7.2364
7.2284
7.2267
7.2251
7.2234
7.2214
7.2174
7.2135
7.2051
7.1999
7.1939
7.1859
7.1820
7.1782
3.6054
3.1808
2.9828
2.9643
2.9431
2.7653
2.7451
2.7254
Ph
O
N
OMe
Me
3v
^{1}H NMR (CDCl$_3$, 400 MHz)
5.37
3.04
3.05
2.07
2.00
f1 (ppm)

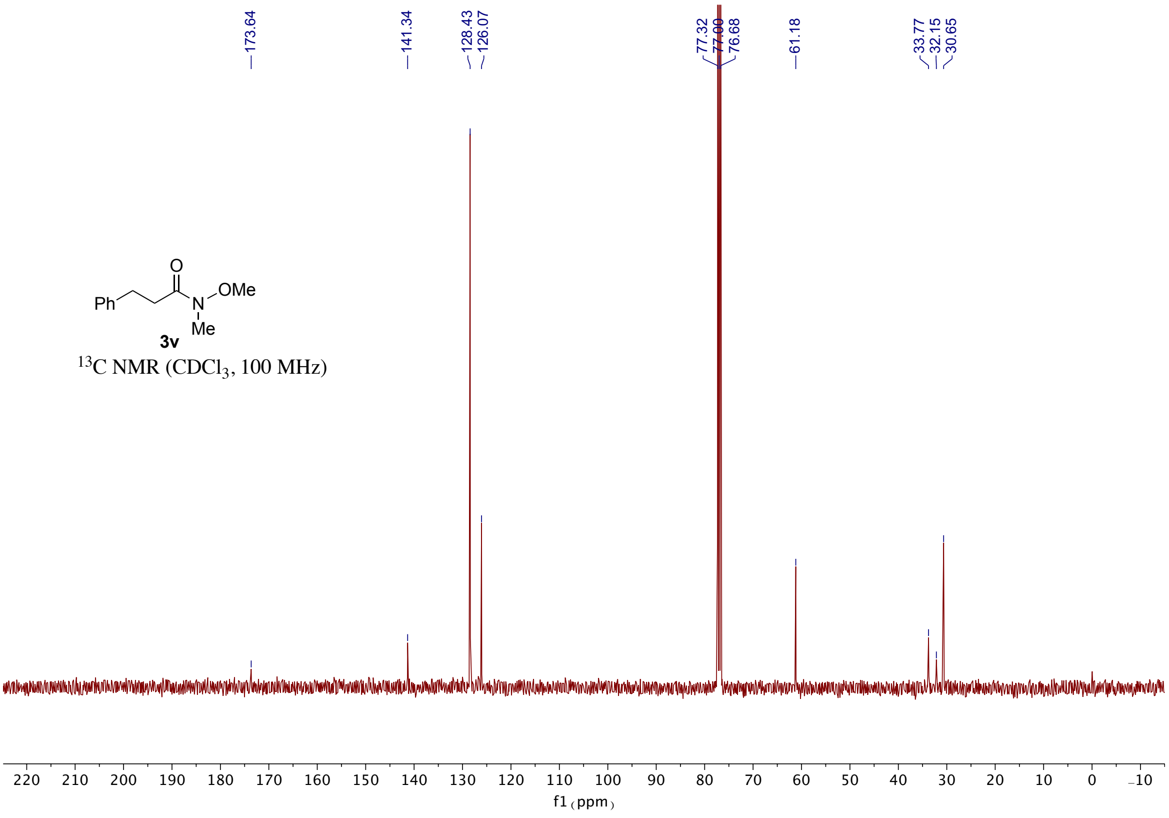
173.64
141.34
128.43
126.07
77.32
77.00
76.68
61.18
33.77
32.15
30.65
Ph
O
N
OMe
Me
3v
^{13}C NMR (CDCl$_3$, 100 MHz)
f1 (ppm)

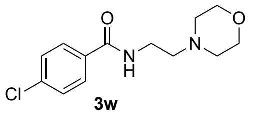

3w

^{1}H NMR ($CDCl_3$, 400 MHz)

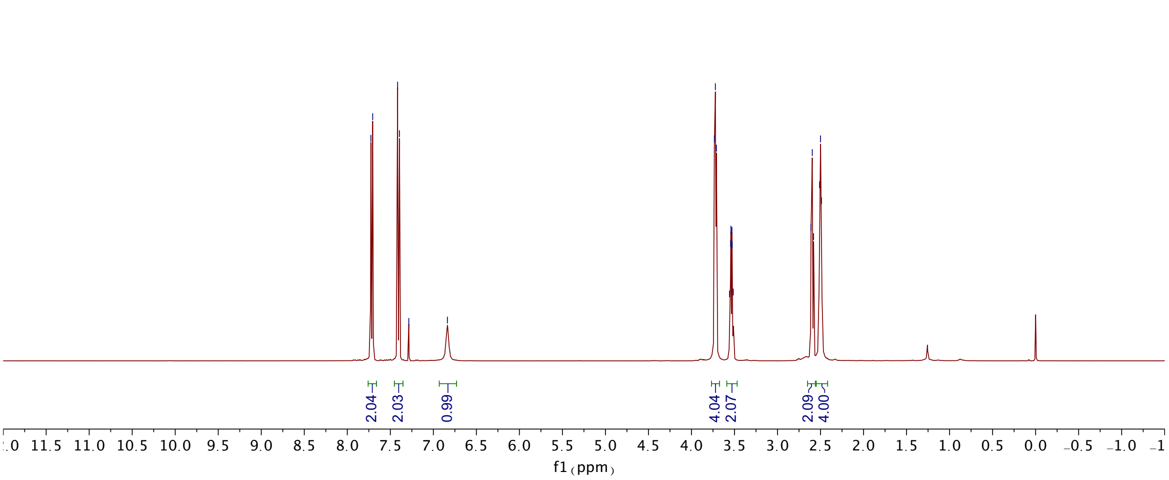

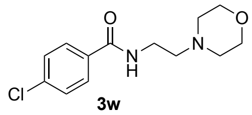

3w

^{13}C NMR ($CDCl_3$, 100 MHz)

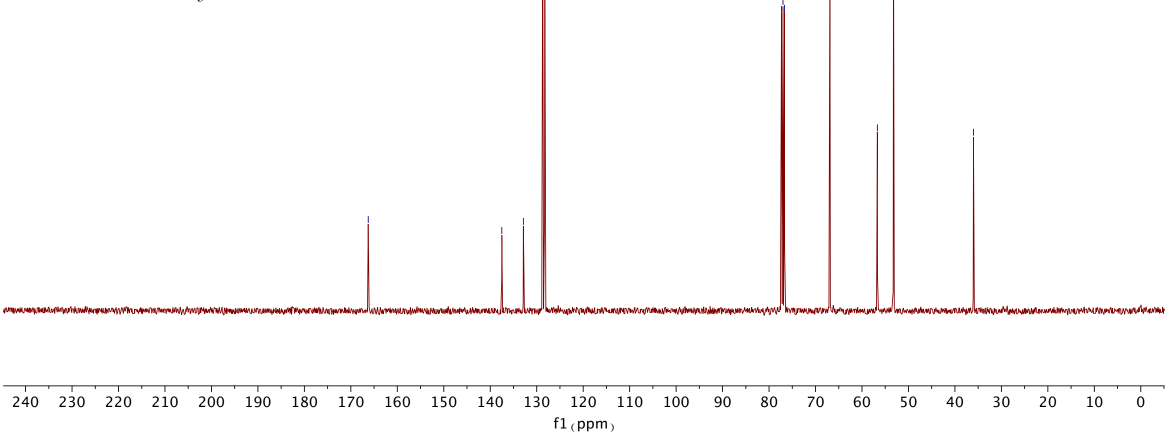

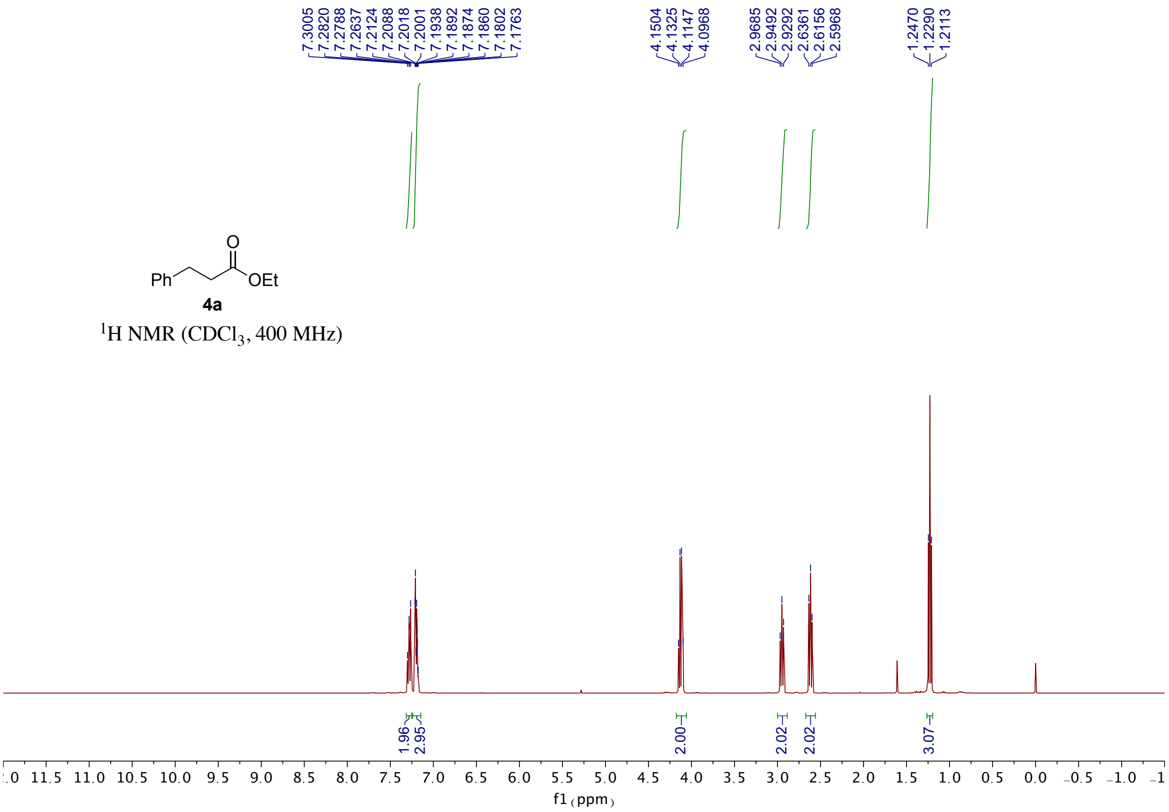
7.3005
7.2820
7.2788
7.2637
7.2124
7.2088
7.2018
7.2001
7.1938
7.1892
7.1874
7.1860
7.1802
7.1763
4.1504
4.1325
4.1147
4.0968
2.9685
2.9492
2.9292
2.6361
2.6156
2.5968
1.2470
1.2290
1.2113
Ph
O
OEt
4a
1H NMR (CDCl3, 400 MHz)
1.96
2.95
2.00
2.02
2.02
3.07
f1 (ppm)

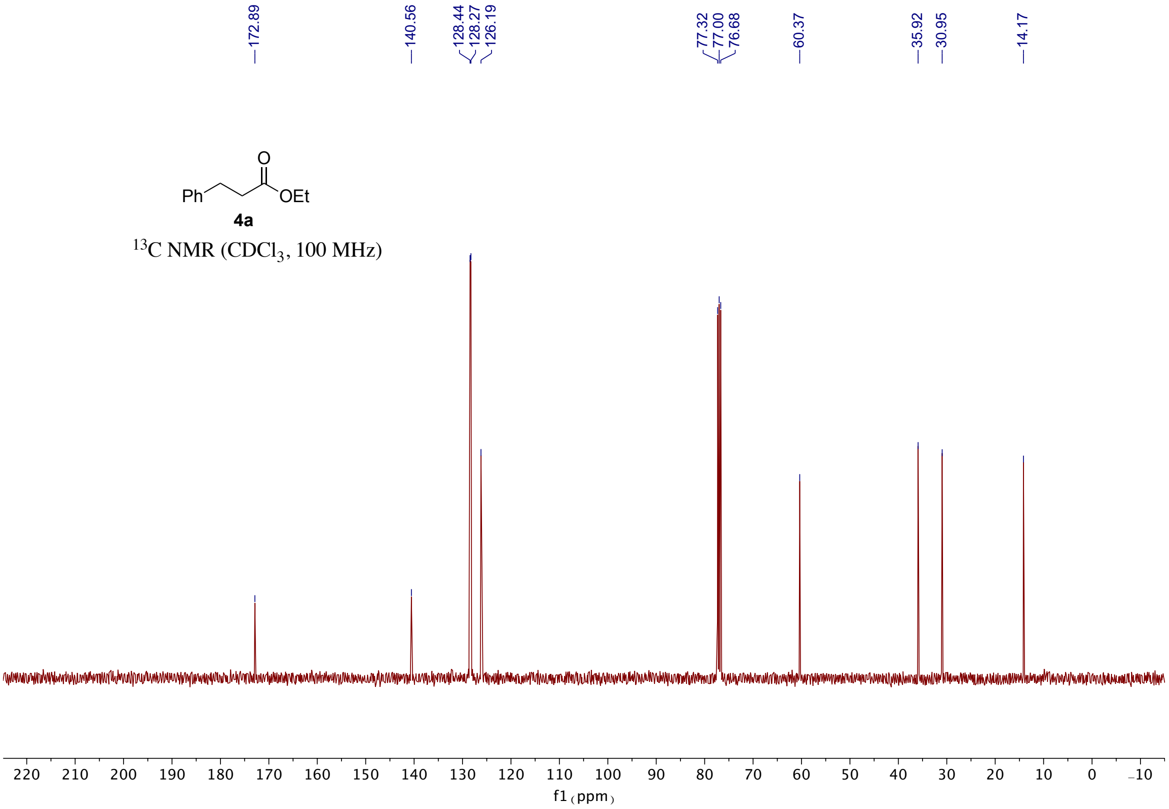
172.89
140.56
128.44
128.27
126.19
77.32
77.00
76.68
60.37
35.92
30.95
14.17
Ph
O
OEt
4a
13C NMR (CDCl3, 100 MHz)
f1 (ppm)

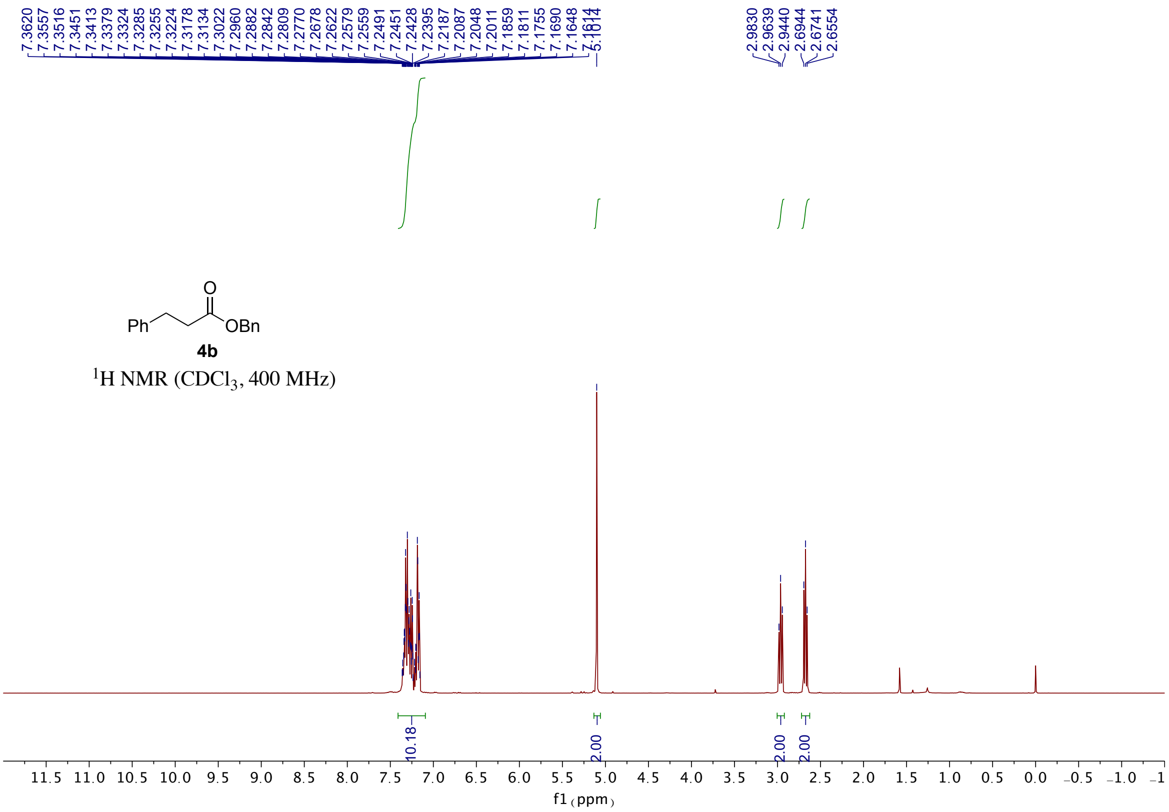
7.3620
7.3557
7.3516
7.3451
7.3413
7.3379
7.3324
7.3285
7.3255
7.3224
7.3178
7.3134
7.3022
7.2960
7.2882
7.2842
7.2809
7.2770
7.2678
7.2622
7.2579
7.2559
7.2491
7.2451
7.2428
7.2395
7.2187
7.2087
7.2048
7.2011
7.1859
7.1811
7.1755
7.1690
7.1648
7.1614
5.1014
2.9830
2.9639
2.9440
2.6944
2.6741
2.6554
O
Ph
OBn
4b
^{1}H NMR ($CDCl_3$, 400 MHz)
10.18
2.00
2.00
2.00
11.5 11.0 10.5 10.0 9.5 9.0 8.5 8.0 7.5 7.0 6.5 6.0 5.5 5.0 4.5 4.0 3.5 3.0 2.5 2.0 1.5 1.0 0.5 0.0 -0.5 -1.0 -1
f1 (ppm)

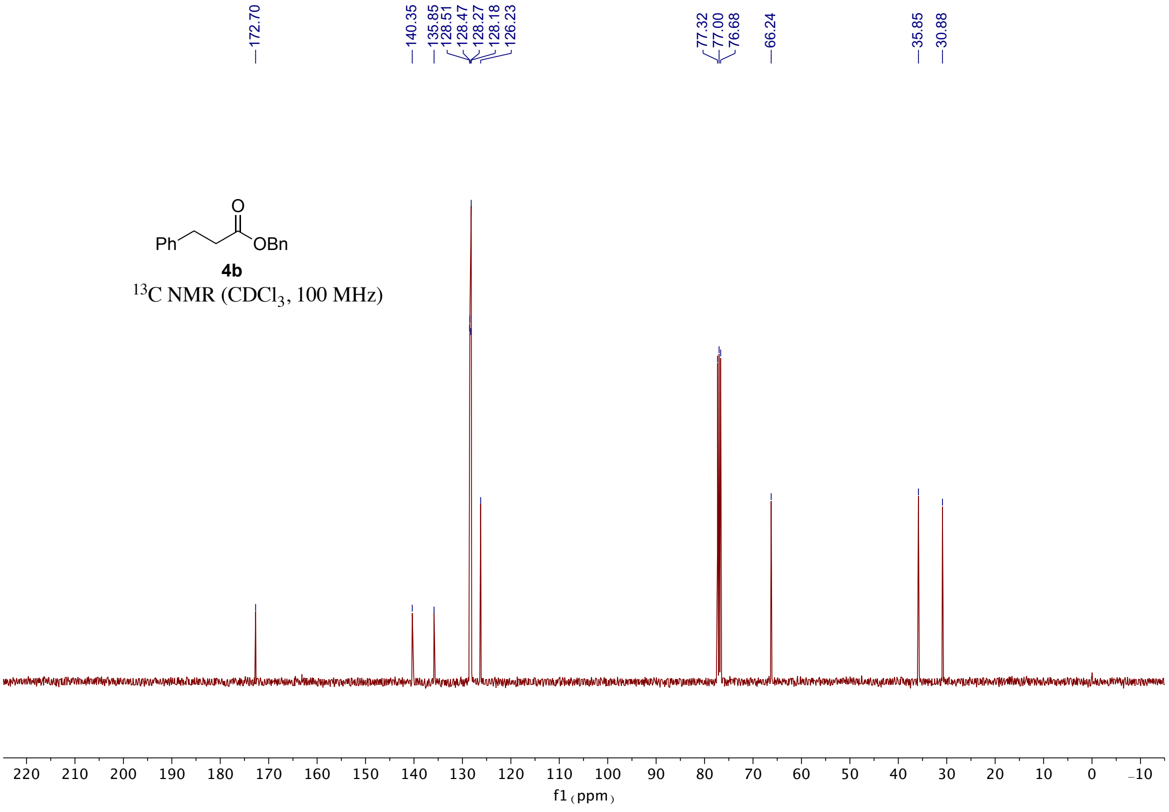
172.70
140.35
135.85
128.51
128.47
128.27
128.18
126.23
77.32
77.00
76.68
66.24
35.85
30.88
O
Ph
OBn
4b
^{13}C NMR ($CDCl_3$, 100 MHz)
220 210 200 190 180 170 160 150 140 130 120 110 100 90 80 70 60 50 40 30 20 10 0 -10
f1 (ppm)

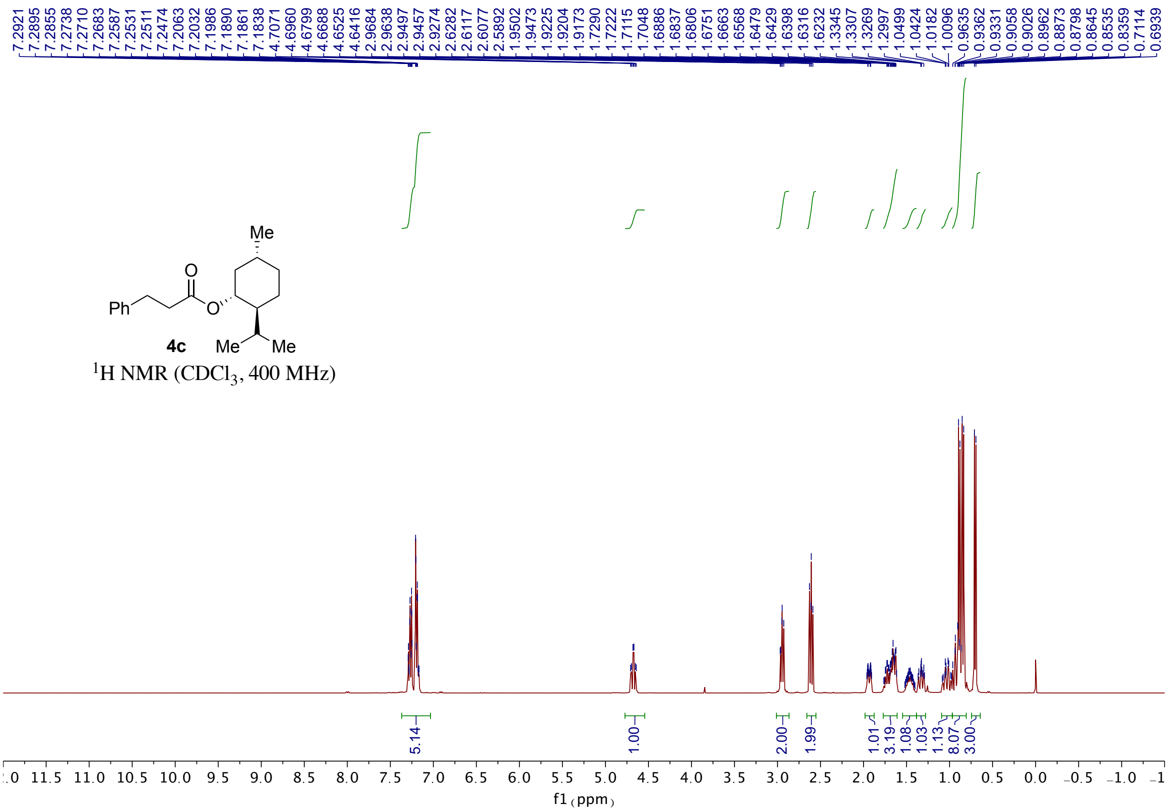
7.2921 7.2895 7.2855 7.2738 7.2710 7.2683 7.2587 7.2531 7.2511 7.2474 7.2063 7.2032 7.1986 7.1890 7.1861 7.1838 4.7071 4.6960 4.6799 4.6688 4.6525 4.6416 2.9684 2.9638 2.9497 2.9457 2.9274 2.6282 2.6117 2.6077 2.5892 1.9502 1.9473 1.9225 1.9204 1.9173 1.7290 1.7222 1.7115 1.7048 1.6886 1.6837 1.6806 1.6751 1.6663 1.6568 1.6479 1.6429 1.6398 1.6316 1.6232 1.3345 1.3307 1.3269 1.2997 1.0499 1.0424 1.0182 1.0096 0.9635 0.9362 0.9331 0.9058 0.9026 0.8962 0.8873 0.8798 0.8645 0.8535 0.8359 0.7114 0.6939
Me
O
Ph
O
4c
Me
Me
^{1}H NMR (CDCl$_3$, 400 MHz)
5.14
1.00
2.00
1.99
1.01
3.19
1.08
1.03
1.13
8.07
3.00
f1 (ppm)

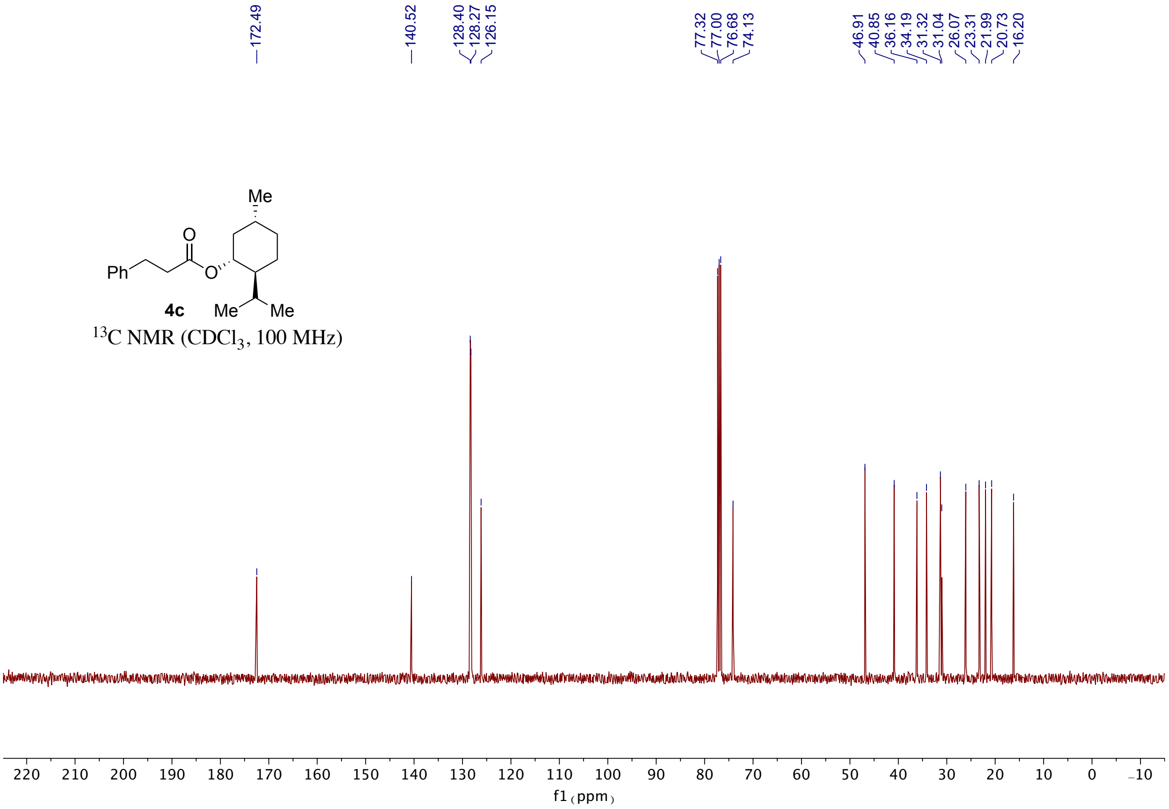
172.49
140.52
128.40 128.27 126.15
77.32 77.00 76.68 74.13
46.91 40.85 36.16 34.19 31.32 31.04 26.07 23.31 21.99 20.73 16.20
Me
O
Ph
O
4c
Me
Me
^{13}C NMR (CDCl$_3$, 100 MHz)
f1 (ppm)

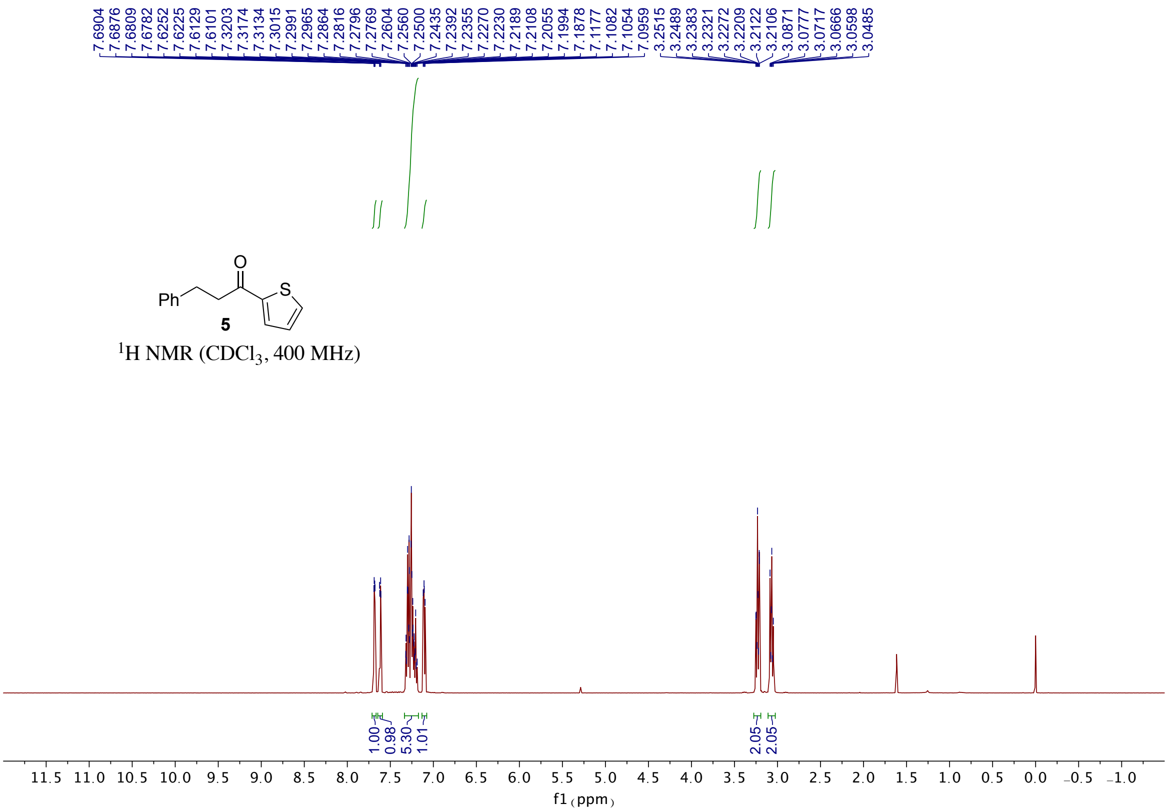

^{1}H NMR ($CDCl_3$, 400 MHz)

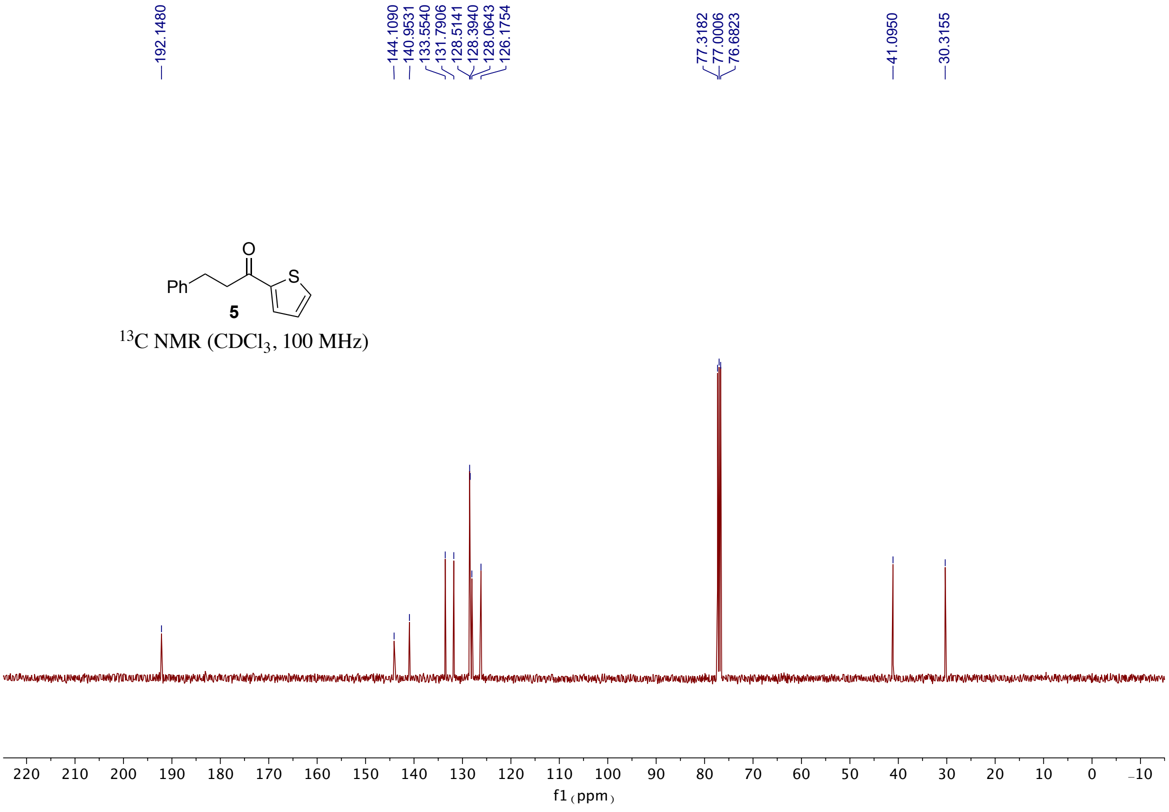

^{13}C NMR ($CDCl_3$, 100 MHz)

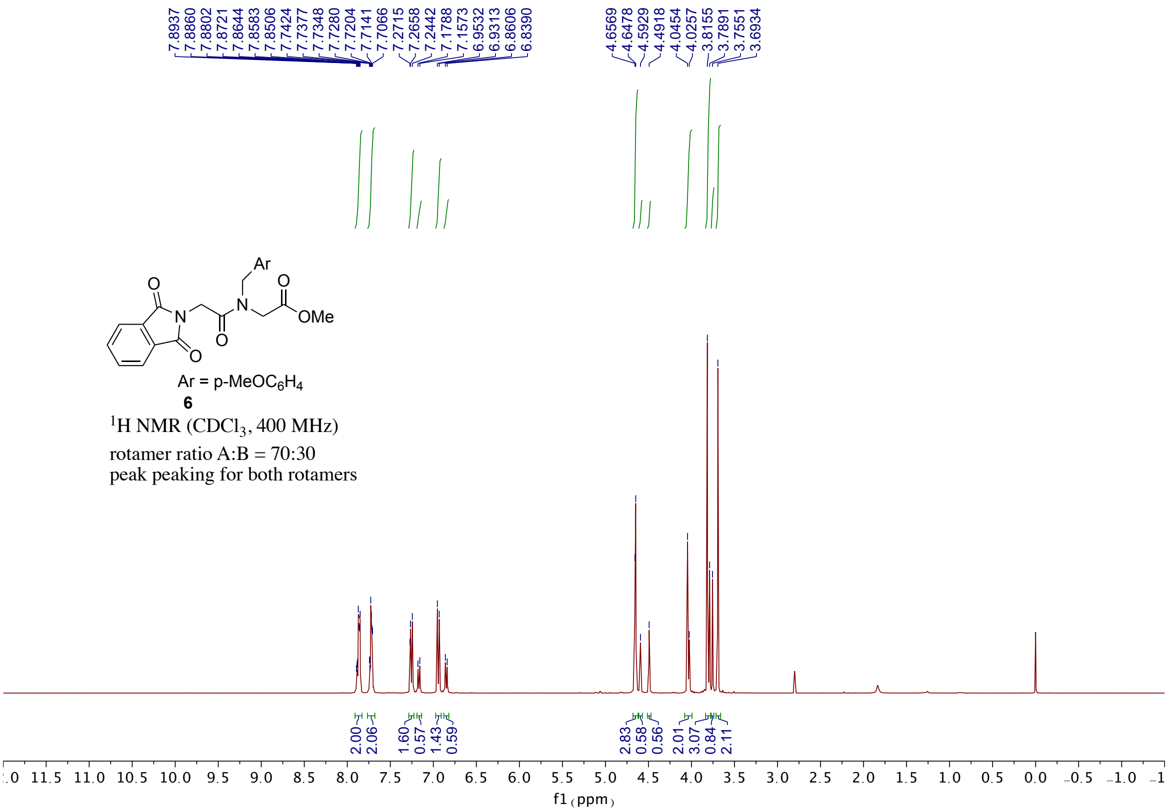
7.8937
7.8860
7.8802
7.8721
7.8644
7.8583
7.8506
7.7424
7.7377
7.7348
7.7280
7.7204
7.7141
7.7066
7.2715
7.2658
7.2442
7.1788
7.1573
6.9532
6.9313
6.8606
6.8390
4.6569
4.6478
4.5929
4.4918
4.0454
4.0257
3.8155
3.7891
3.7551
3.6934
Ar
O
N
N
O
OMe
O
O
Ar = p-MeOC6H4
6
1H NMR (CDCl3, 400 MHz)
rotamer ratio A:B = 70:30
peak peaking for both rotamers
2.00
2.06
1.60
0.57
1.43
0.59
2.83
0.58
0.56
2.01
3.07
0.84
2.11
f1 (ppm)

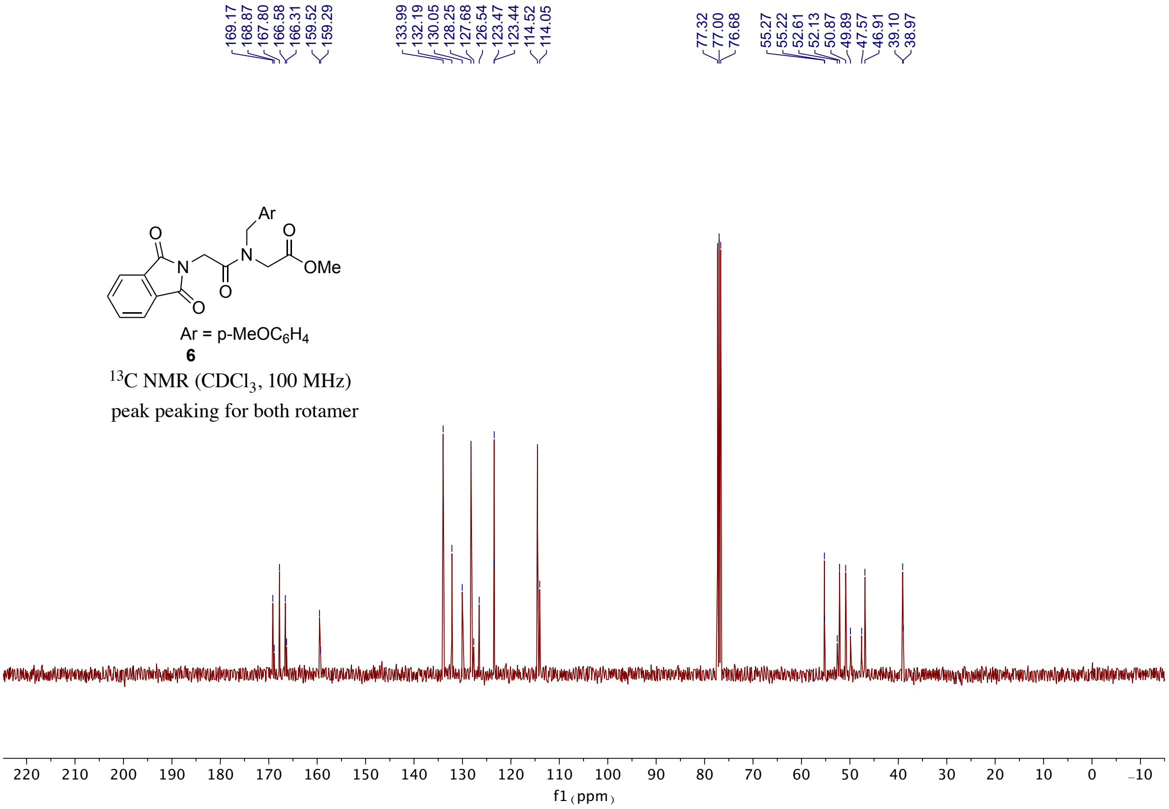
169.17
168.87
167.80
166.58
166.31
159.52
159.29
133.99
132.19
130.05
128.25
127.68
126.54
123.47
123.44
114.52
114.05
77.32
77.00
76.68
55.27
55.22
52.61
52.13
50.87
49.89
47.57
46.91
39.10
38.97
Ar
O
N
N
O
OMe
O
O
Ar = p-MeOC6H4
6
13C NMR (CDCl3, 100 MHz)
peak peaking for both rotamer
f1 (ppm)

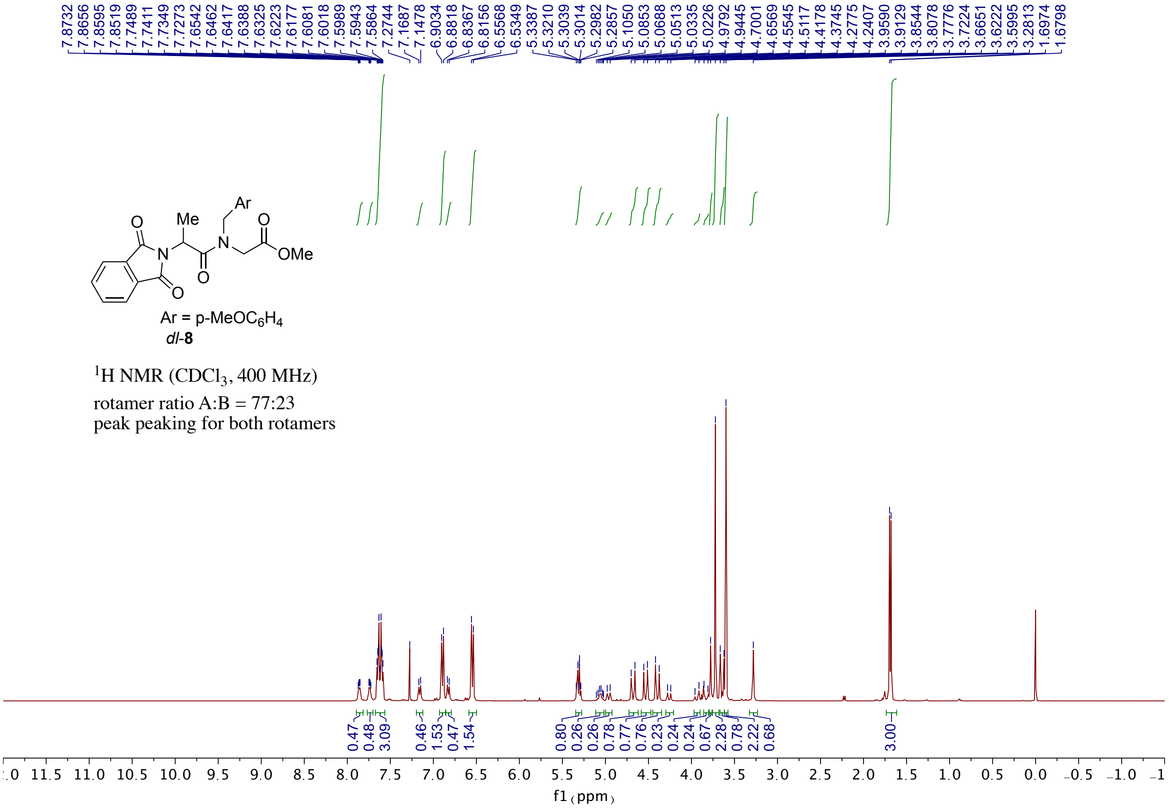

Ar = p-$MeOC_6H_4$
dl-**8**

^{1}H NMR ($CDCl_3$, 400 MHz)
rotamer ratio A:B = 77:23
peak peaking for both rotamers

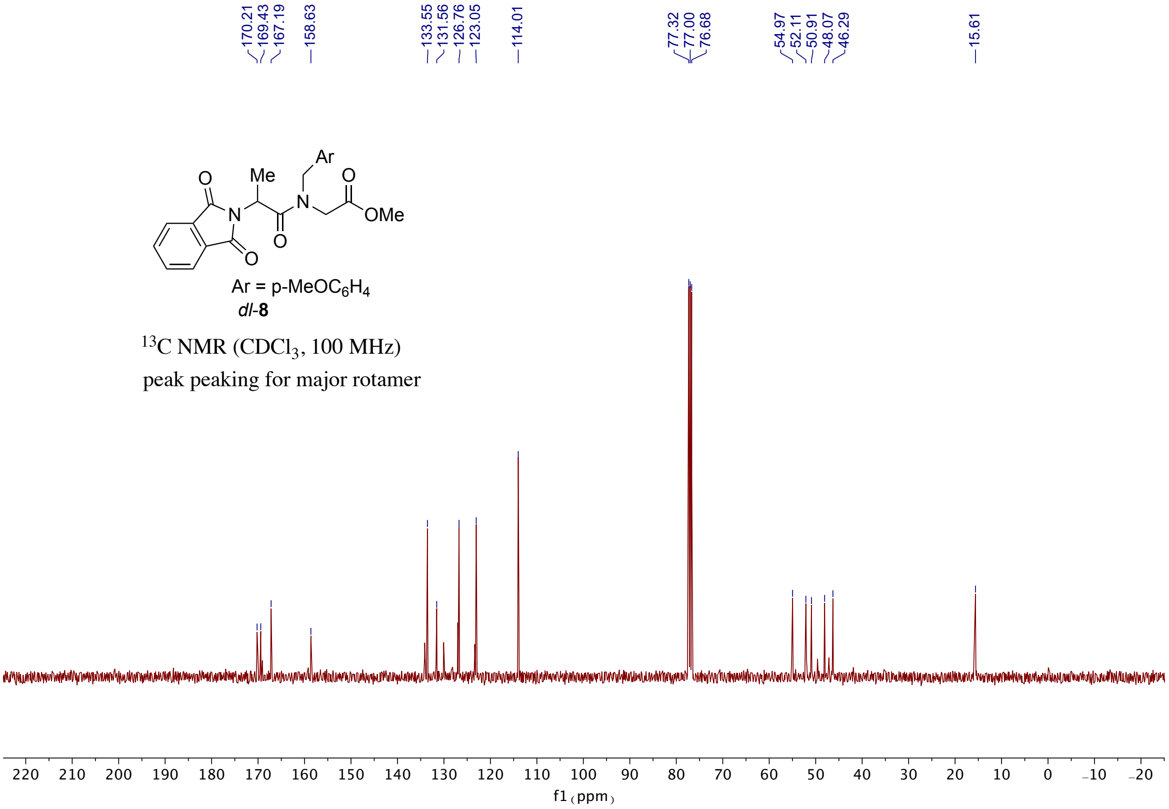

Ar = p-$MeOC_6H_4$
dl-**8**

^{13}C NMR ($CDCl_3$, 100 MHz)
peak peaking for major rotamer

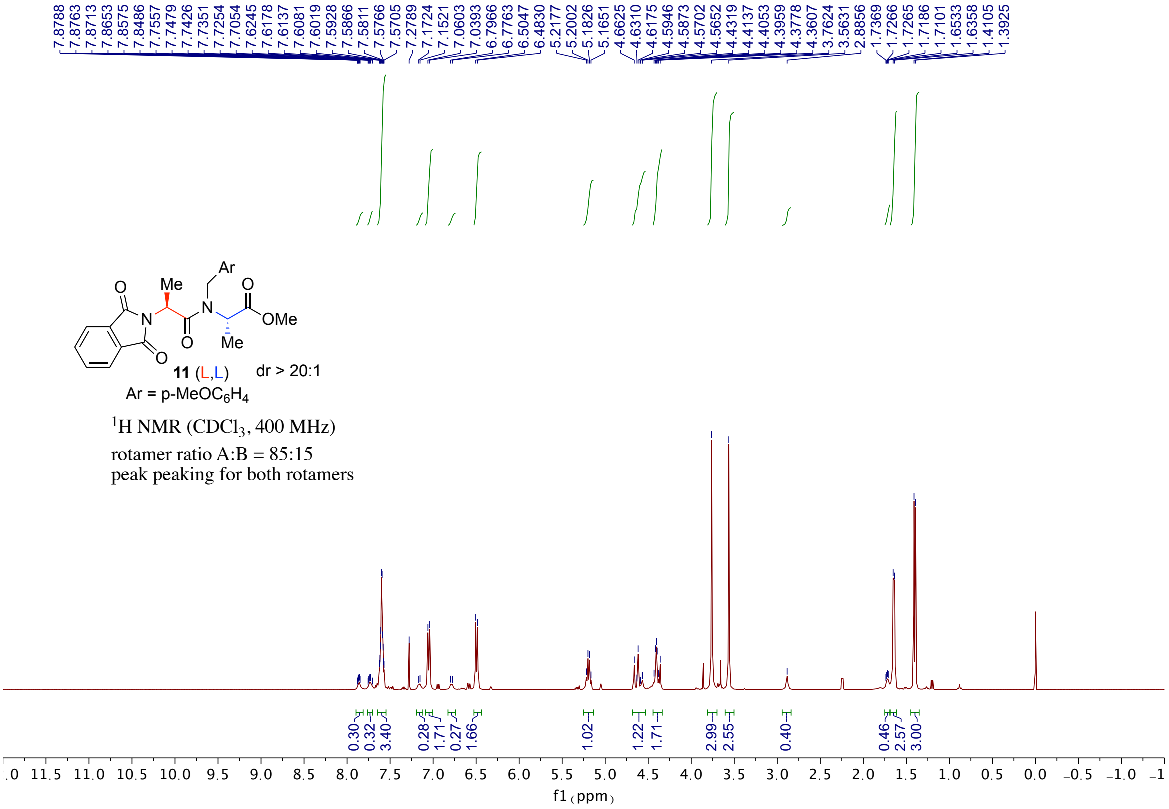
7.8788
7.8763
7.8713
7.8653
7.8575
7.8486
7.7557
7.7479
7.7426
7.7351
7.7254
7.7054
7.6245
7.6178
7.6137
7.6081
7.6019
7.5928
7.5866
7.5811
7.5766
7.5705
7.2789
7.1724
7.1521
7.0603
7.0393
6.7966
6.7763
6.5047
6.4830
5.2177
5.2002
5.1826
5.1651
4.6625
4.6310
4.6175
4.5946
4.5873
4.5702
4.5652
4.4319
4.4137
4.4053
4.3959
4.3778
4.3607
3.7624
3.5631
2.8856
1.7369
1.7266
1.7265
1.7186
1.7101
1.6533
1.6358
1.4105
1.3925
Ar
Me
O
N
O
N
O
OMe
Me
O
11 (L,L) dr > 20:1
Ar = p-MeOC6H4
1H NMR (CDCl3, 400 MHz)
rotamer ratio A:B = 85:15
peak peaking for both rotamers
0.30
0.32
3.40
0.28
1.71
0.27
1.66
1.02
1.22
1.71
2.99
2.55
0.40
0.46
2.57
3.00
f1 (ppm)

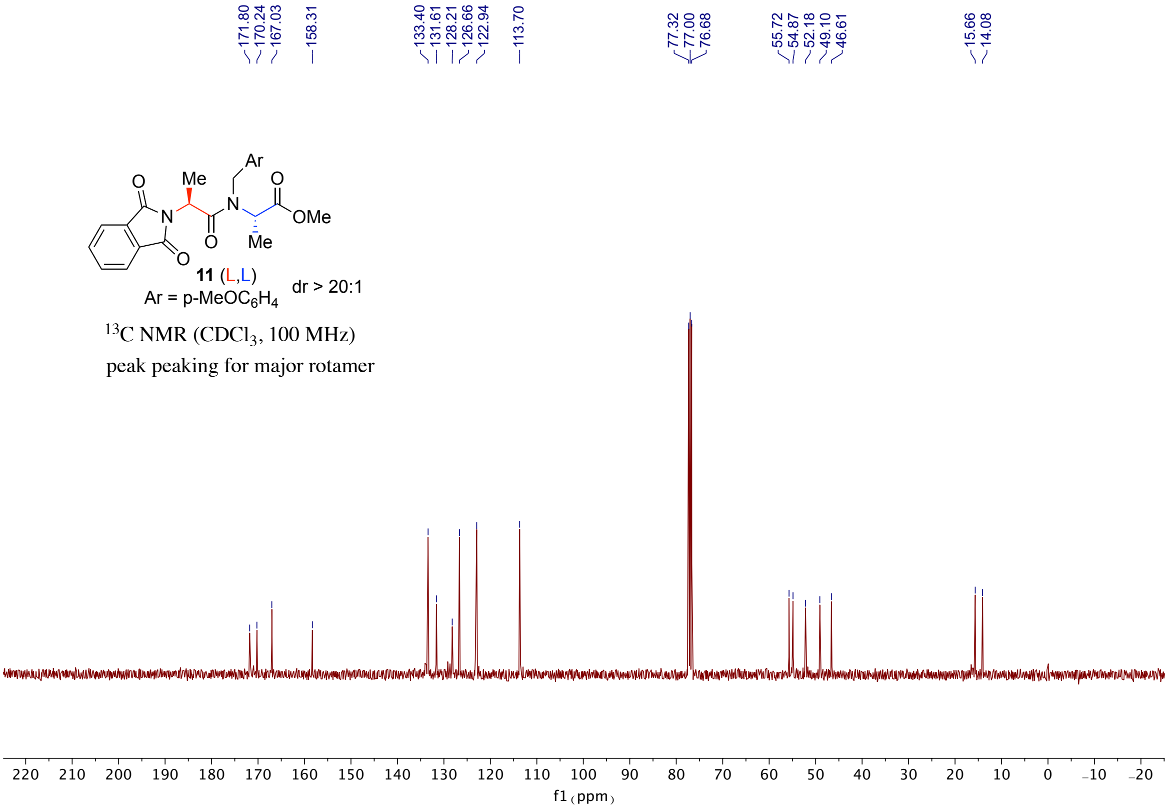
171.80
170.24
167.03
158.31
133.40
131.61
128.21
126.66
122.94
113.70
77.32
77.00
76.68
55.72
54.87
52.18
49.10
46.61
15.66
14.08
Ar
O
Me
O
N
N
OMe
O
Me
O
11 (L,L)
Ar = p-MeOC6H4
dr > 20:1
13C NMR (CDCl3, 100 MHz)
peak peaking for major rotamer
f1 (ppm)

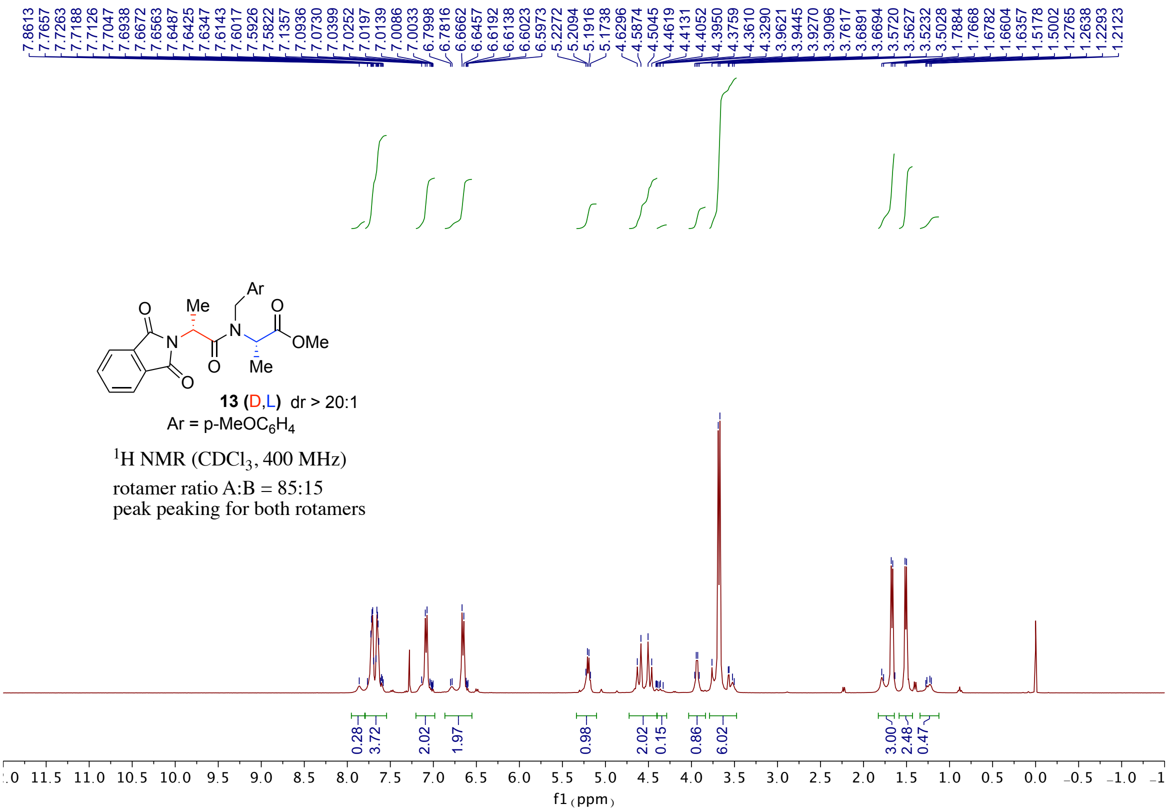
7.8613 7.7657 7.7263 7.7188 7.7126 7.7047 7.6938 7.6672 7.6563 7.6487 7.6425 7.6347 7.6143 7.6017 7.5926 7.5822 7.1357 7.0936 7.0730 7.0399 7.0252 7.0197 7.0139 7.0086 7.0033 6.7998 6.7816 6.6662 6.6457 6.6192 6.6138 6.6023 6.5973 5.2272 5.2094 5.1916 5.1738 4.6296 4.5874 4.5045 4.4619 4.4131 4.4052 4.3950 4.3759 4.3610 4.3290 3.9621 3.9445 3.9270 3.9096 3.7617 3.6891 3.6694 3.5720 3.5627 3.5232 3.5028 1.7884 1.7668 1.6782 1.6604 1.6357 1.5178 1.5002 1.2765 1.2638 1.2293 1.2123
Ar
Me
O
O
N
N
OMe
O
O
Me
13 (D,L) dr > 20:1
Ar = p-MeOC6H4
1H NMR (CDCl3, 400 MHz)
rotamer ratio A:B = 85:15
peak peaking for both rotamers
0.28 3.72 2.02 1.97 0.98 2.02 0.15 0.86 6.02 3.00 2.48 0.47
f1 (ppm)

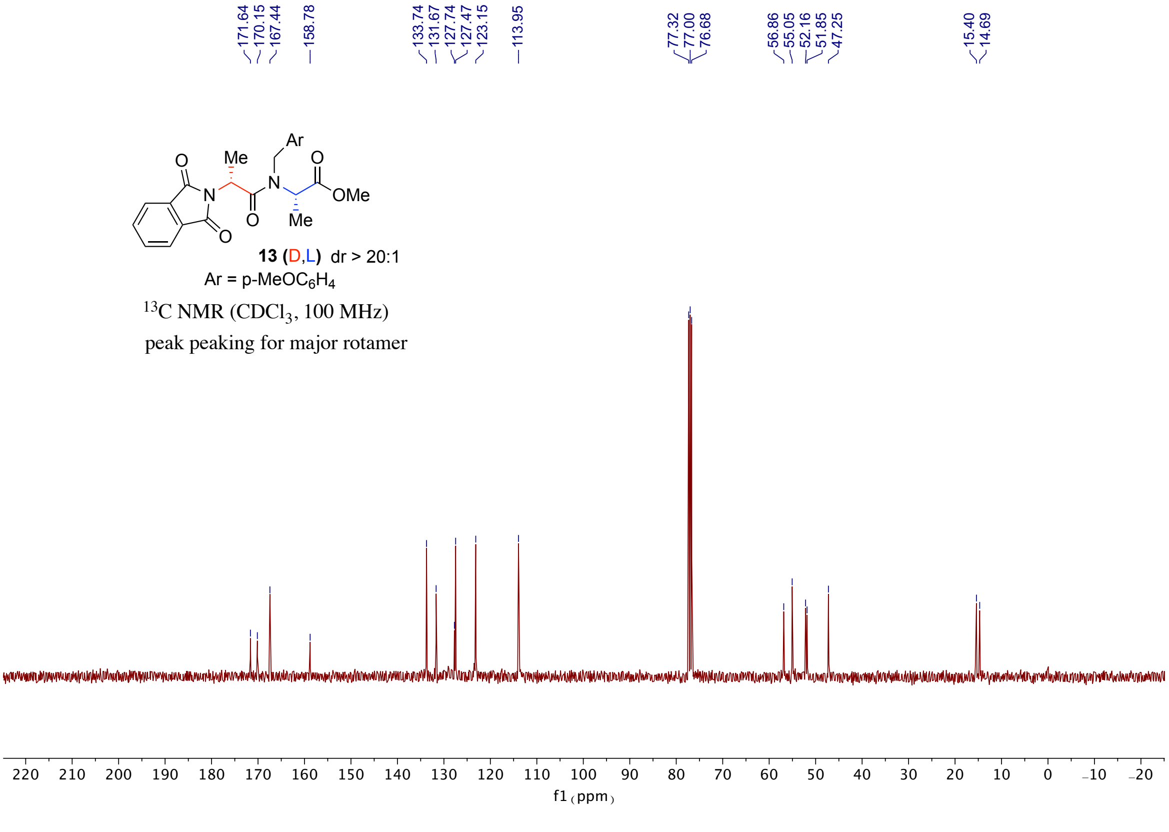
171.64 170.15 167.44 158.78 133.74 131.67 127.74 127.47 123.15 113.95 77.32 77.00 76.68 56.86 55.05 52.16 51.85 47.25 15.40 14.69
Ar
Me
O
O
N
N
OMe
O
O
Me
13 (D,L) dr > 20:1
Ar = p-MeOC6H4
13C NMR (CDCl3, 100 MHz)
peak peaking for major rotamer
f1 (ppm)

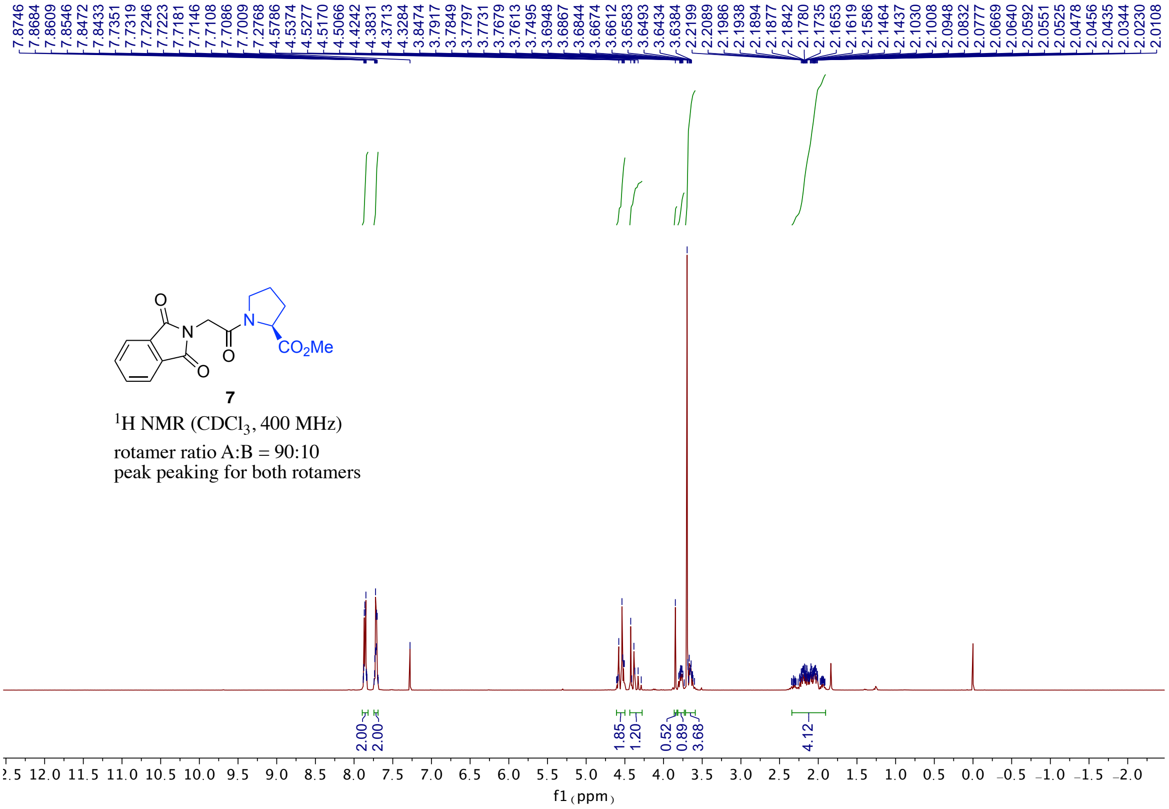
7
1H NMR (CDCl3, 400 MHz)
rotamer ratio A:B = 90:10
peak peaking for both rotamers
CO2Me
7.8746 7.8684 7.8609 7.8546 7.8472 7.8433 7.7351 7.7319 7.7246 7.7223 7.7181 7.7146 7.7108 7.7086 7.7009 7.2768 4.5786 4.5374 4.5277 4.5170 4.5066 4.4242 4.3831 4.3713 4.3284 3.8474 3.7917 3.7849 3.7797 3.7731 3.7679 3.7613 3.7495 3.6948 3.6867 3.6844 3.6674 3.6612 3.6583 3.6493 3.6434 3.6384 2.2199 2.2089 2.1986 2.1938 2.1894 2.1877 2.1842 2.1780 2.1735 2.1653 2.1619 2.1586 2.1464 2.1437 2.1030 2.1008 2.0948 2.0832 2.0777 2.0669 2.0640 2.0592 2.0551 2.0525 2.0478 2.0456 2.0435 2.0344 2.0230 2.0108
2.00 2.00 1.85 1.20 0.52 0.89 3.68 4.12
f1 (ppm)

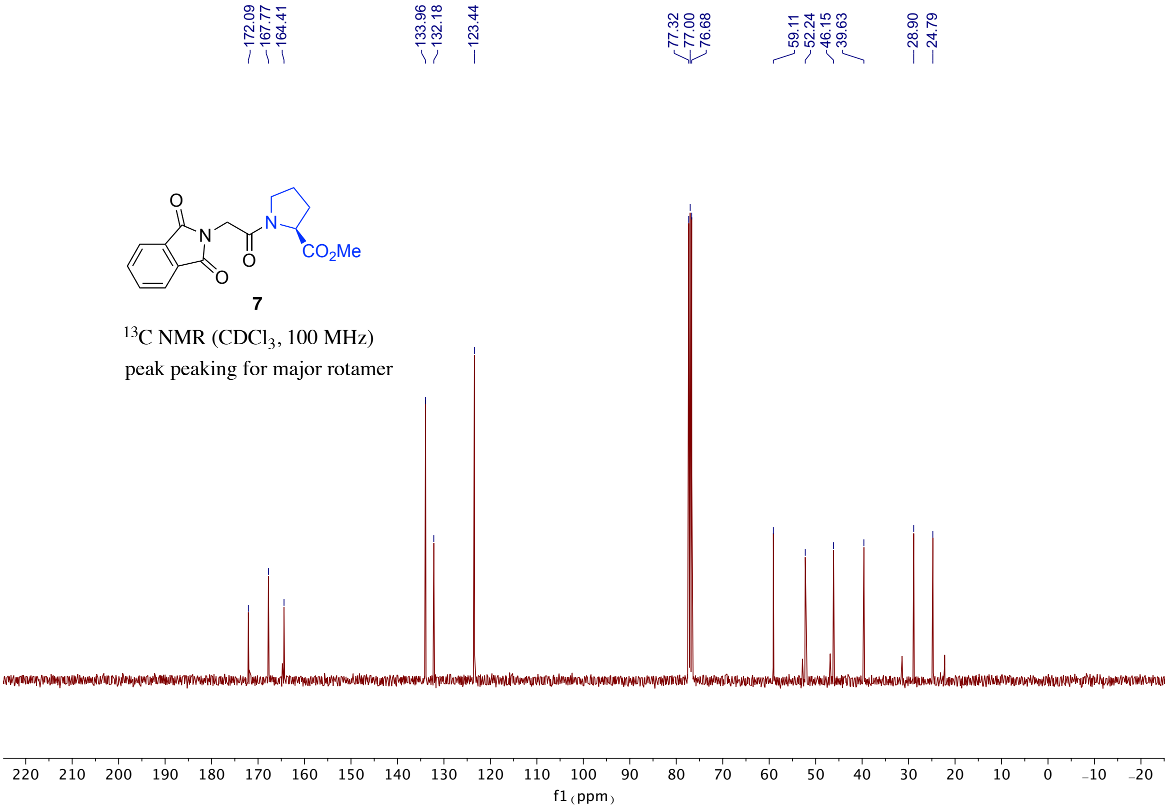
7
13C NMR (CDCl3, 100 MHz)
peak peaking for major rotamer
CO2Me
172.09 167.77 164.41 133.96 132.18 123.44 77.32 77.00 76.68 59.11 52.24 46.15 39.63 28.90 24.79
f1 (ppm)

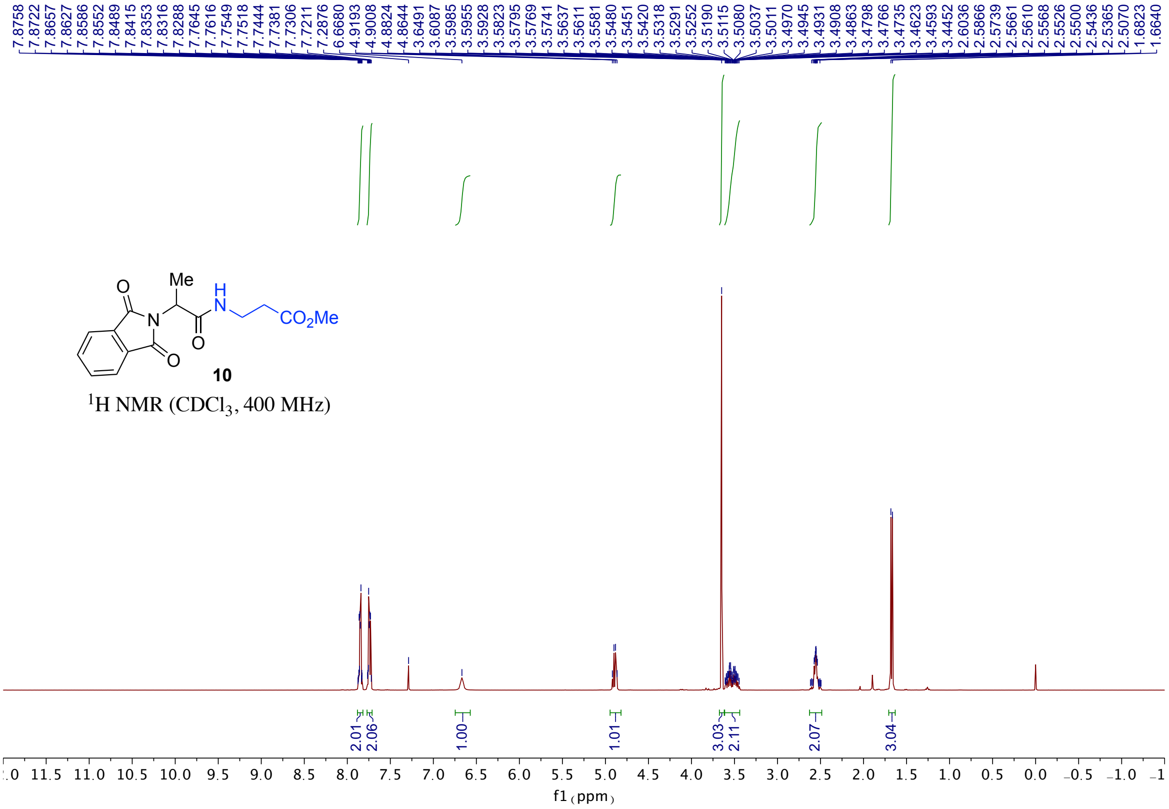
Me
H
N
CO2Me
10
1H NMR (CDCl3, 400 MHz)
f1 (ppm)

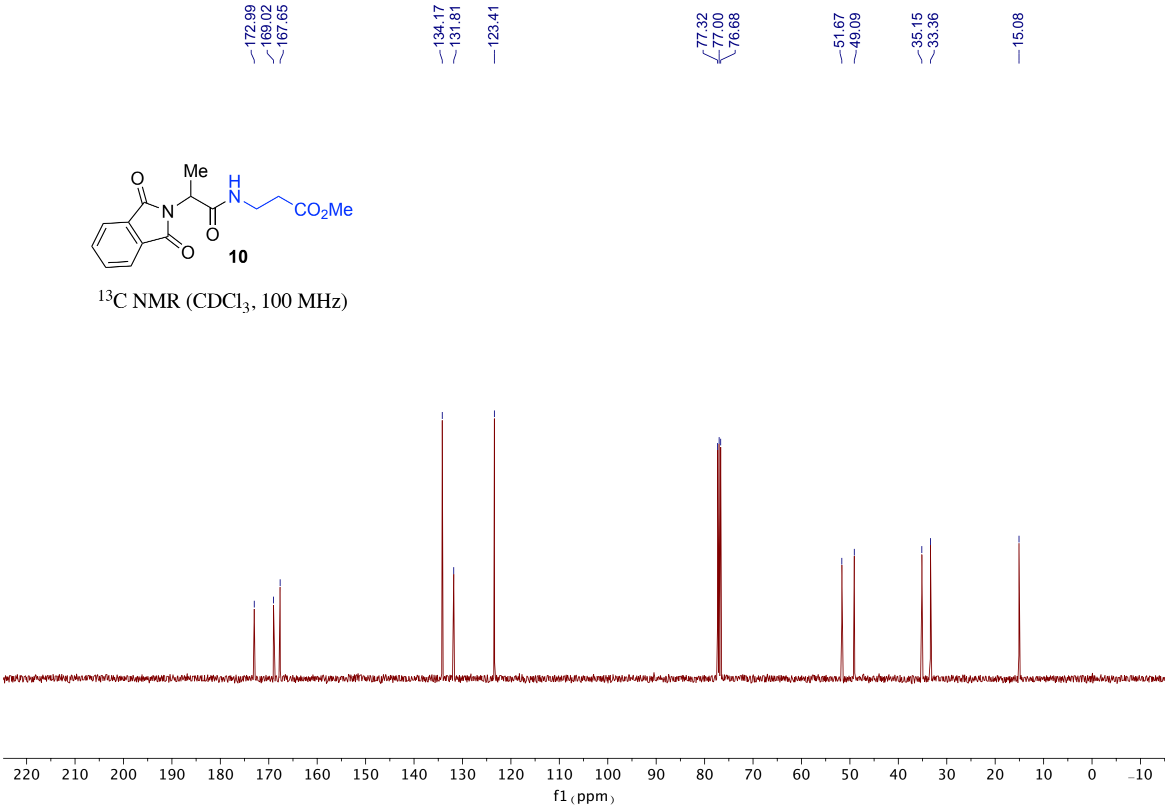
172.99
169.02
167.65
134.17
131.81
123.41
77.32
77.00
76.68
51.67
49.09
35.15
33.36
15.08
Me
H
N
CO2Me
10
13C NMR (CDCl3, 100 MHz)
f1 (ppm)

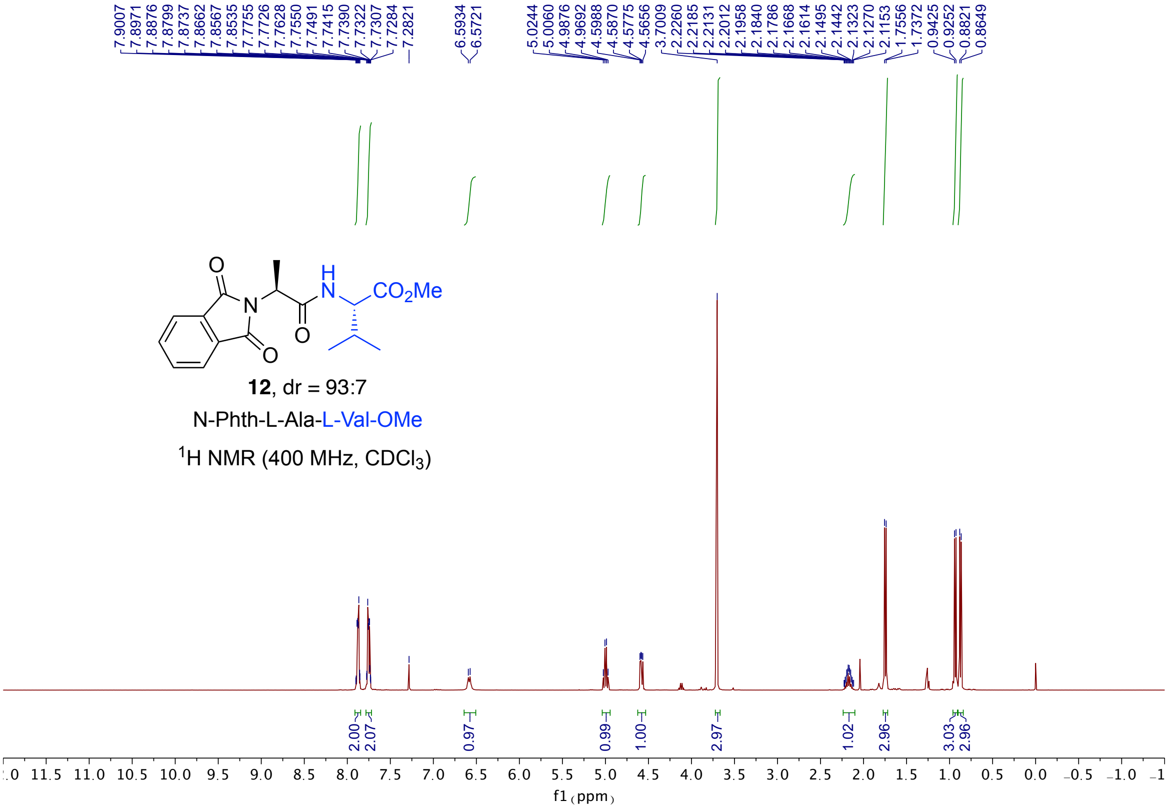

12, dr = 93:7

N-Phth-L-Ala-L-Val-OMe

^{1}H NMR (400 MHz, $CDCl_3$)

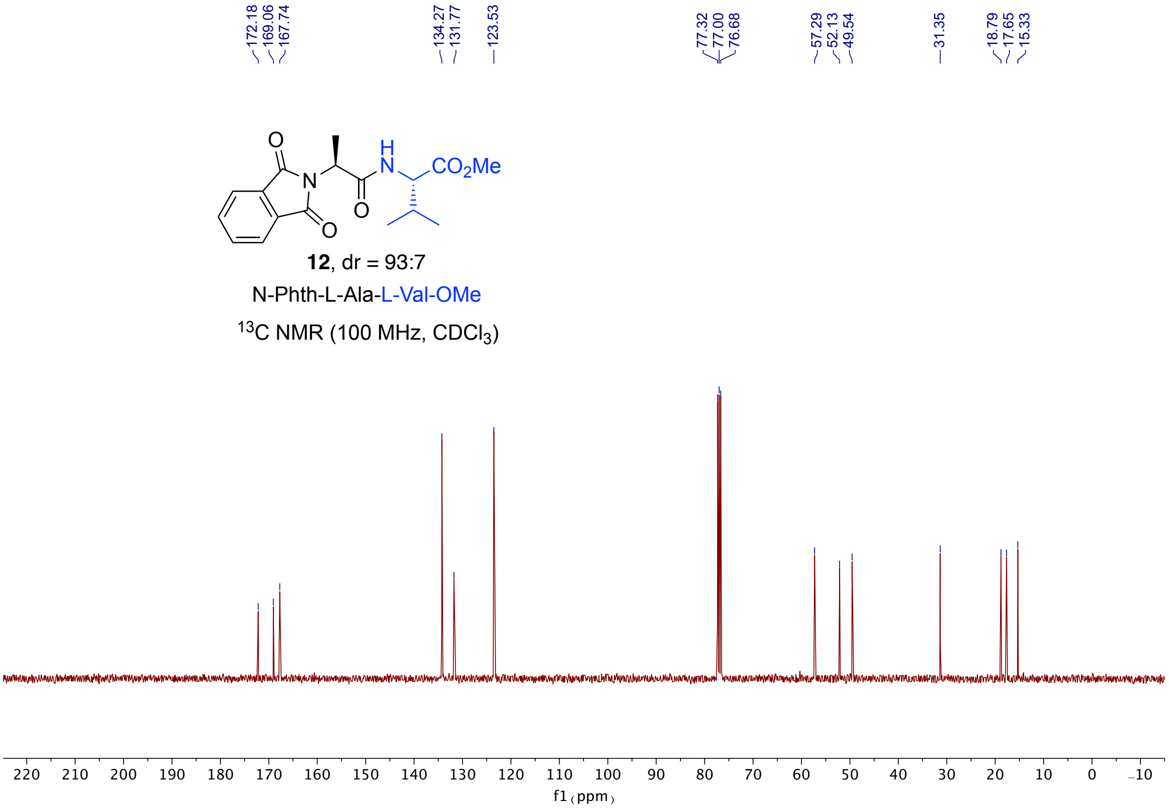

12, dr = 93:7

N-Phth-L-Ala-L-Val-OMe

^{13}C NMR (100 MHz, $CDCl_3$)

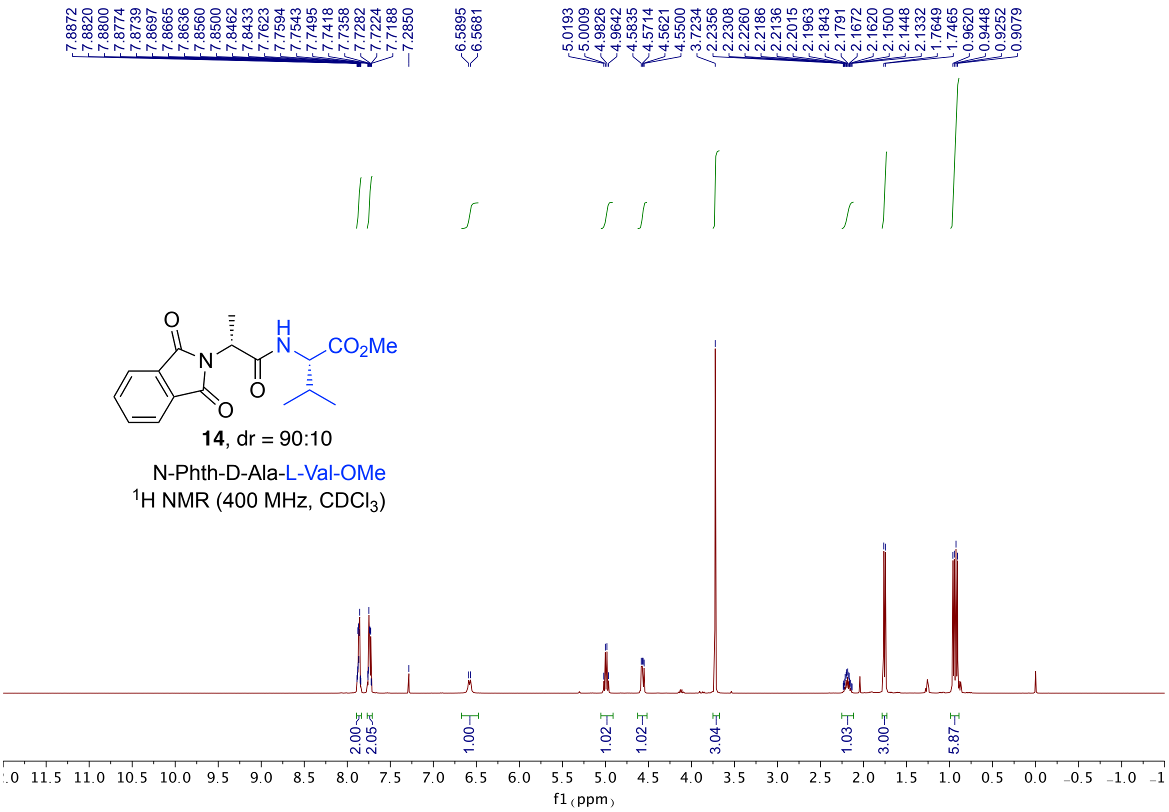

14, dr = 90:10
N-Phth-D-Ala-L-Val-OMe
^{1}H NMR (400 MHz, $CDCl_3$)
f1 (ppm)

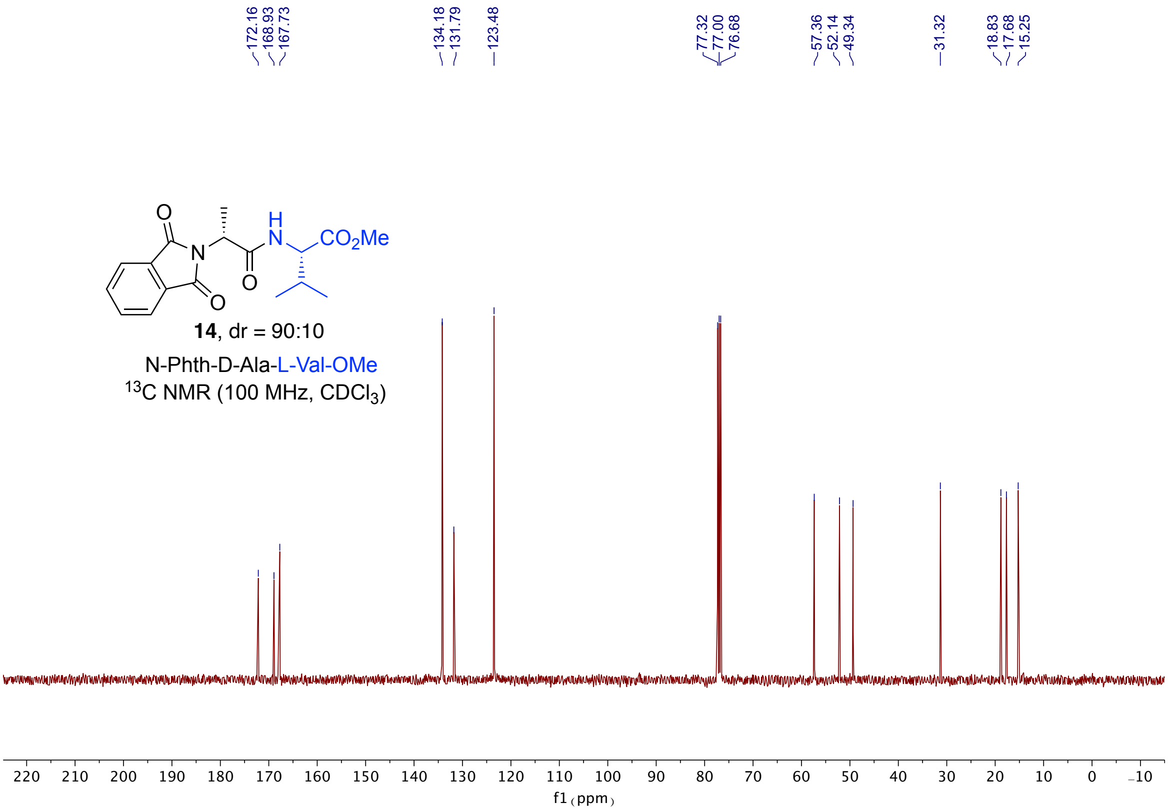

14, dr = 90:10
N-Phth-D-Ala-L-Val-OMe
^{13}C NMR (100 MHz, $CDCl_3$)
f1 (ppm)

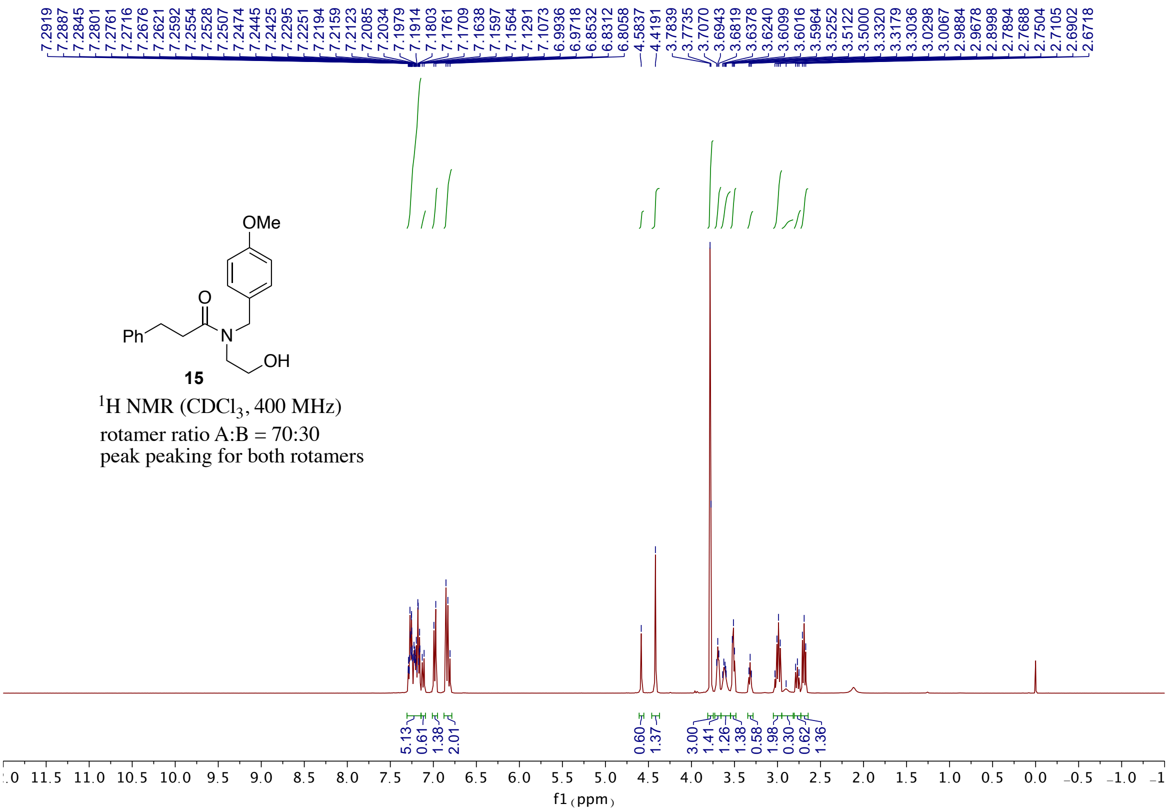
7.2919 7.2887 7.2845 7.2801 7.2761 7.2716 7.2676 7.2621 7.2592 7.2554 7.2528 7.2507 7.2474 7.2445 7.2425 7.2295 7.2251 7.2194 7.2159 7.2123 7.2085 7.2034 7.1979 7.1914 7.1803 7.1761 7.1709 7.1638 7.1597 7.1564 7.1291 7.1073 6.9936 6.9718 6.8532 6.8312 6.8058 4.5837 4.4191 3.7839 3.7735 3.7070 3.6943 3.6819 3.6378 3.6240 3.6099 3.6016 3.5964 3.5252 3.5122 3.5000 3.3320 3.3179 3.3036 3.0298 3.0067 2.9884 2.9678 2.8998 2.7894 2.7688 2.7504 2.7105 2.6902 2.6718
OMe
O
Ph
N
OH
15
1H NMR (CDCl3, 400 MHz)
rotamer ratio A:B = 70:30
peak peaking for both rotamers
5.13 0.61 1.38 2.01 0.60 1.37 3.00 1.41 1.26 1.38 0.58 1.98 0.30 0.62 1.36
f1 (ppm)

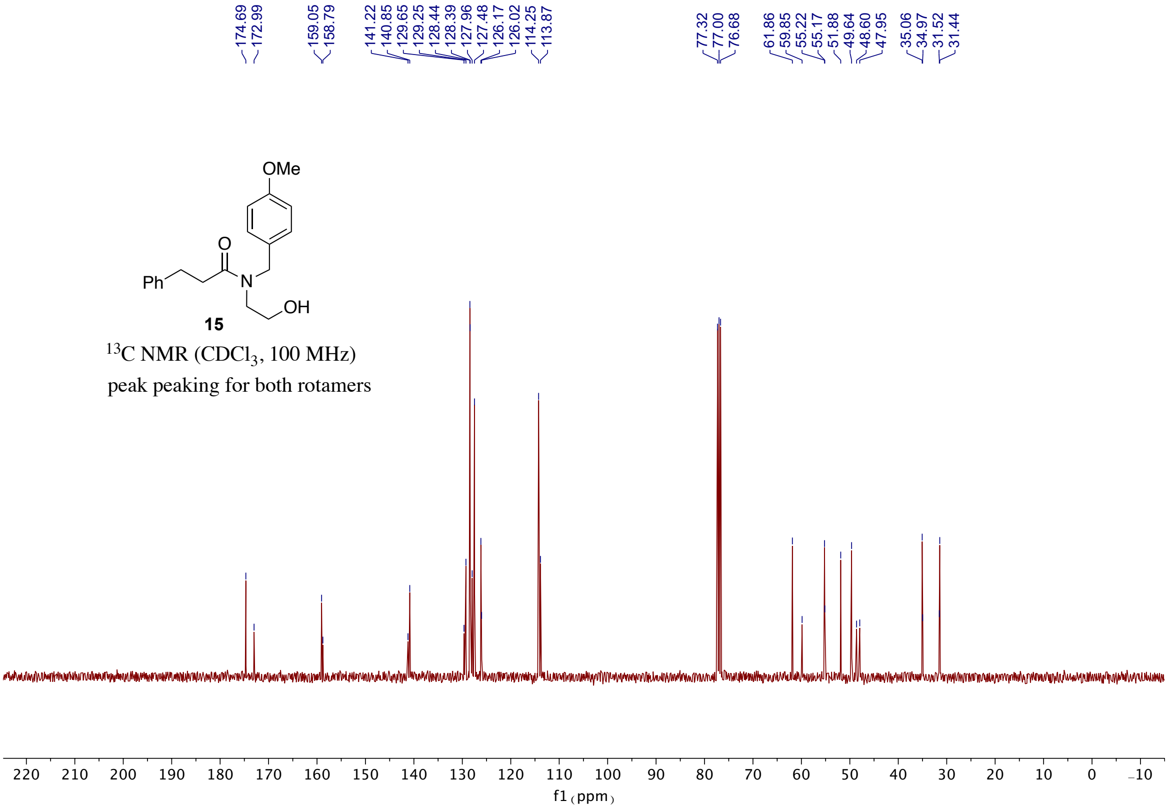
174.69 172.99 159.05 158.79 141.22 140.85 129.65 129.25 128.44 128.39 127.96 127.48 126.17 126.02 114.25 113.87 77.32 77.00 76.68 61.86 59.85 55.22 55.17 51.88 49.64 48.60 47.95 35.06 34.97 31.52 31.44
OMe
O
Ph
N
OH
15
13C NMR (CDCl3, 100 MHz)
peak peaking for both rotamers
f1 (ppm)

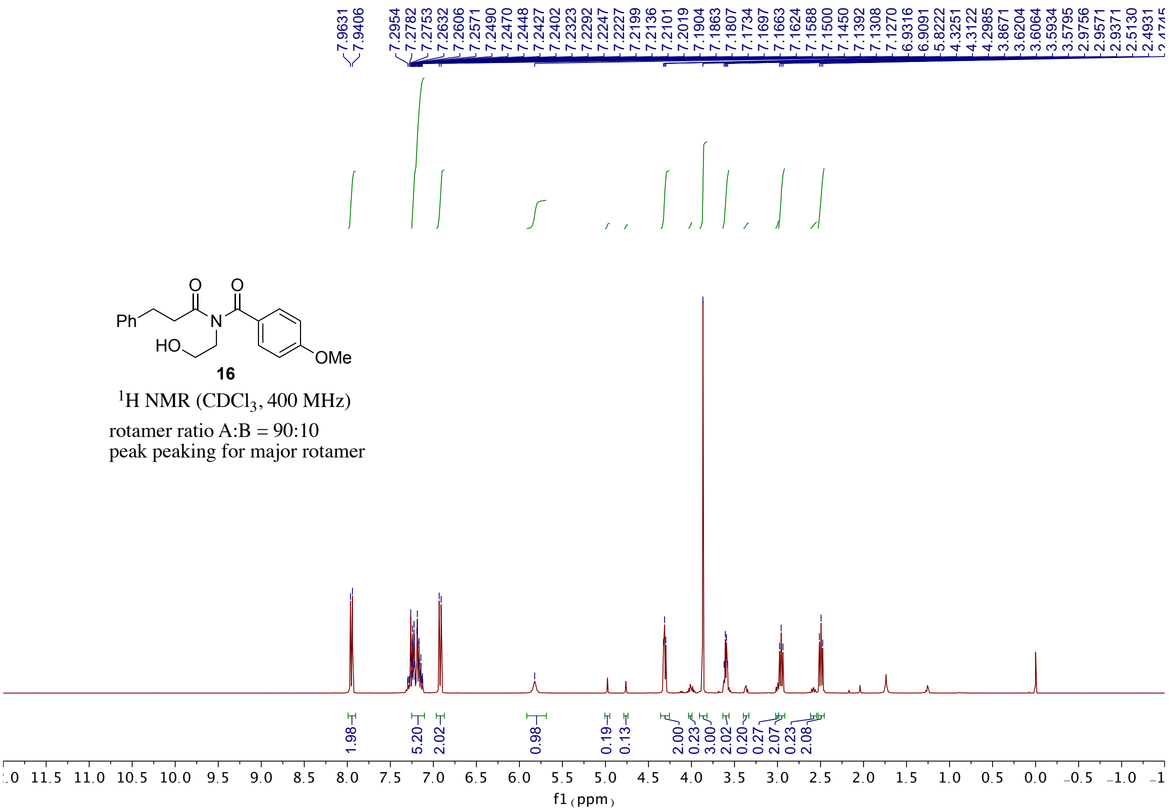

16
^{1}H NMR (CDCl$_3$, 400 MHz)
rotamer ratio A:B = 90:10
peak peaking for major rotamer

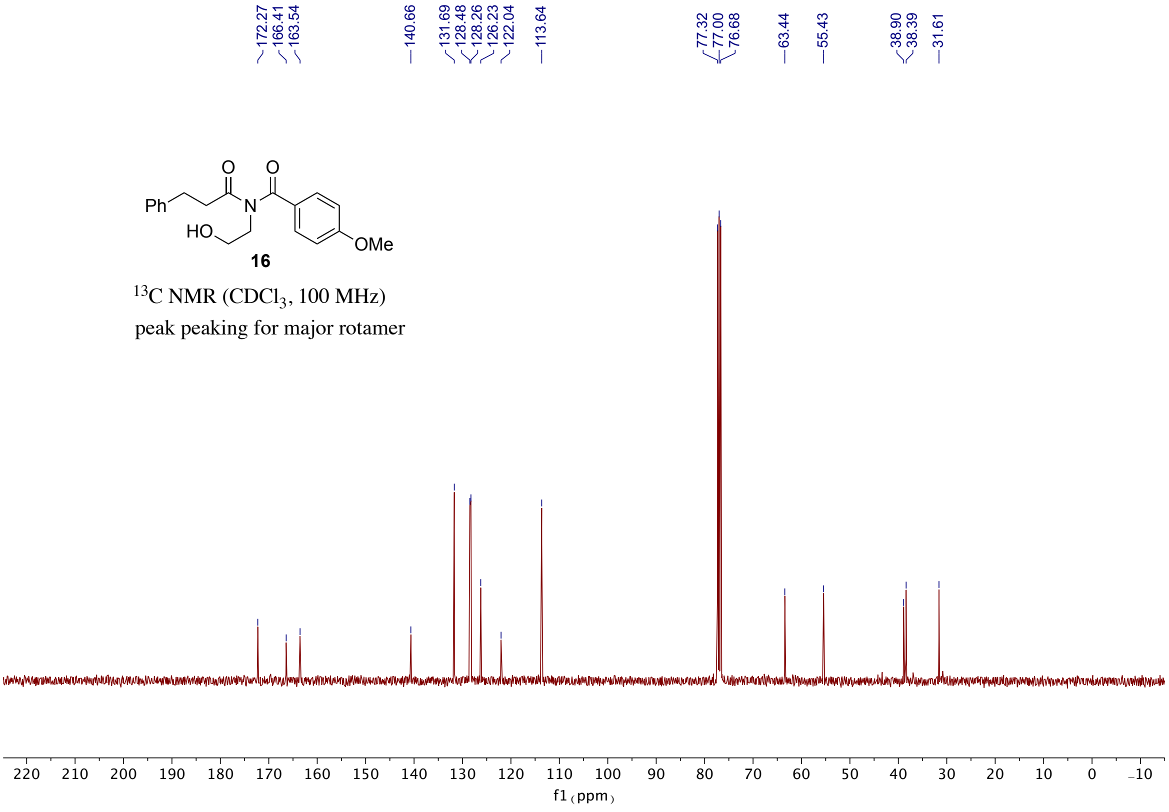

16
^{13}C NMR (CDCl$_3$, 100 MHz)
peak peaking for major rotamer

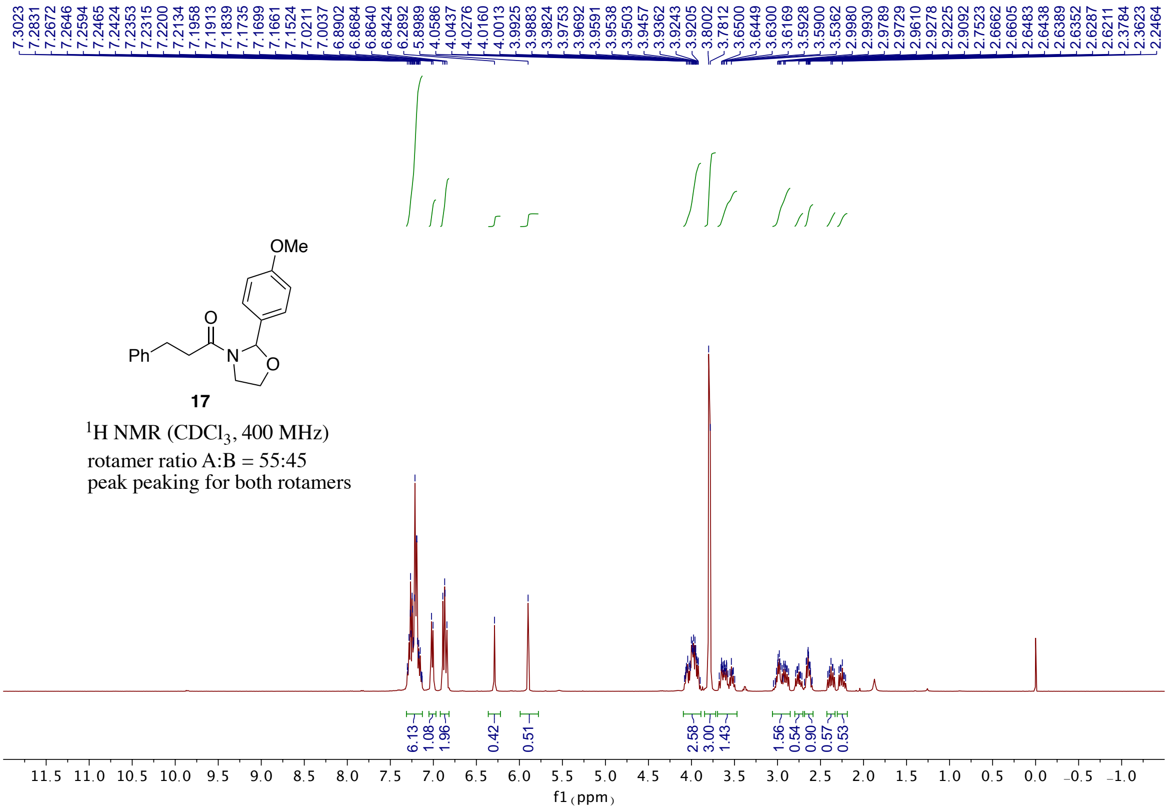
7.3023
7.2831
7.2672
7.2646
7.2594
7.2465
7.2424
7.2353
7.2315
7.2200
7.2134
7.1958
7.1913
7.1839
7.1735
7.1699
7.1661
7.1524
7.0211
7.0037
6.8902
6.8684
6.8640
6.8424
6.2892
5.8989
4.0586
4.0437
4.0276
4.0160
4.0013
3.9925
3.9883
3.9824
3.9753
3.9692
3.9591
3.9538
3.9503
3.9457
3.9362
3.9243
3.9205
3.8002
3.7812
3.6500
3.6449
3.6300
3.6169
3.5928
3.5900
3.5362
2.9980
2.9930
2.9789
2.9729
2.9610
2.9278
2.9225
2.9092
2.7523
2.6662
2.6605
2.6483
2.6438
2.6389
2.6352
2.6287
2.6211
2.3784
2.3623
2.2464
OMe
O
Ph
N
O
17
^{1}H NMR ($CDCl_3$, 400 MHz)
rotamer ratio A:B = 55:45
peak peaking for both rotamers
6.13
1.08
1.96
0.42
0.51
2.58
3.00
1.43
1.56
0.54
0.90
0.57
0.53
11.5 11.0 10.5 10.0 9.5 9.0 8.5 8.0 7.5 7.0 6.5 6.0 5.5 5.0 4.5 4.0 3.5 3.0 2.5 2.0 1.5 1.0 0.5 0.0 -0.5 -1.0
f1 (ppm)

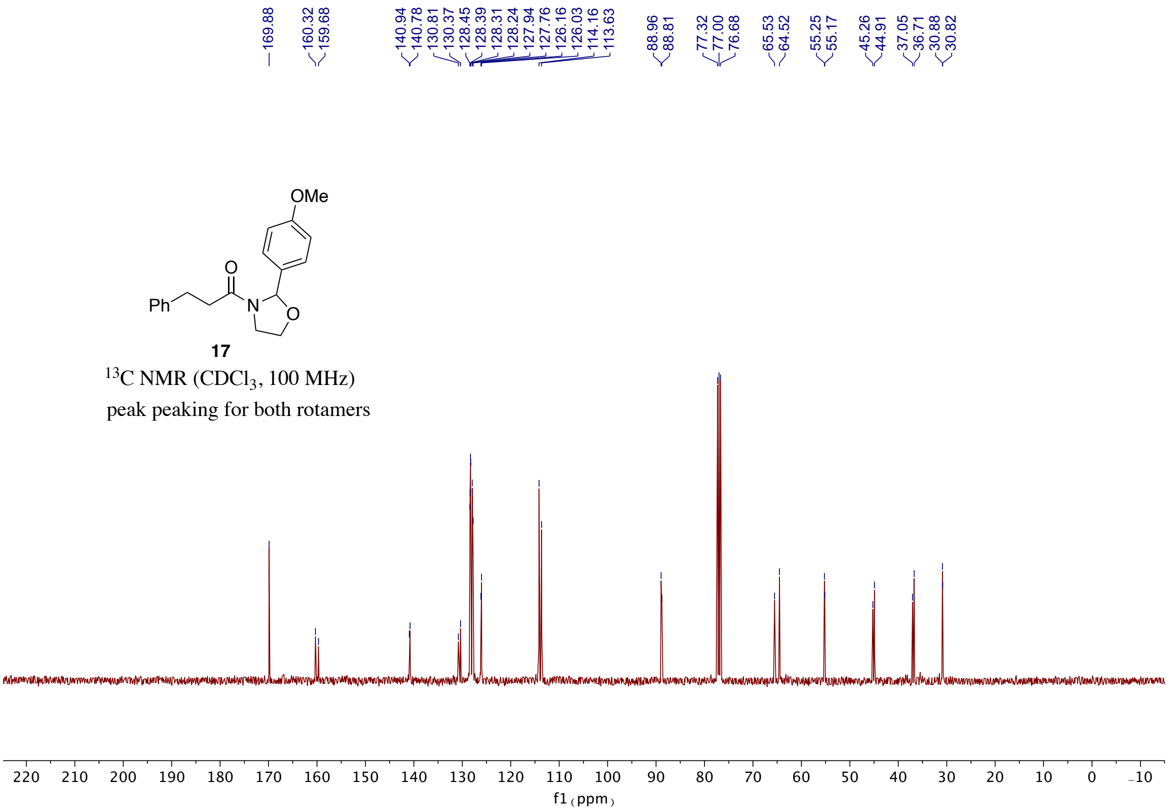
169.88
160.32
159.68
140.94
140.78
130.81
130.37
128.45
128.39
128.31
128.24
127.94
127.76
126.16
126.03
114.16
113.63
88.96
88.81
77.32
77.00
76.68
65.53
64.52
55.25
55.17
45.26
44.91
37.05
36.71
30.88
30.82
OMe
O
Ph
N
O
17
^{13}C NMR ($CDCl_3$, 100 MHz)
peak peaking for both rotamers
220 210 200 190 180 170 160 150 140 130 120 110 100 90 80 70 60 50 40 30 20 10 0 -10
f1 (ppm)

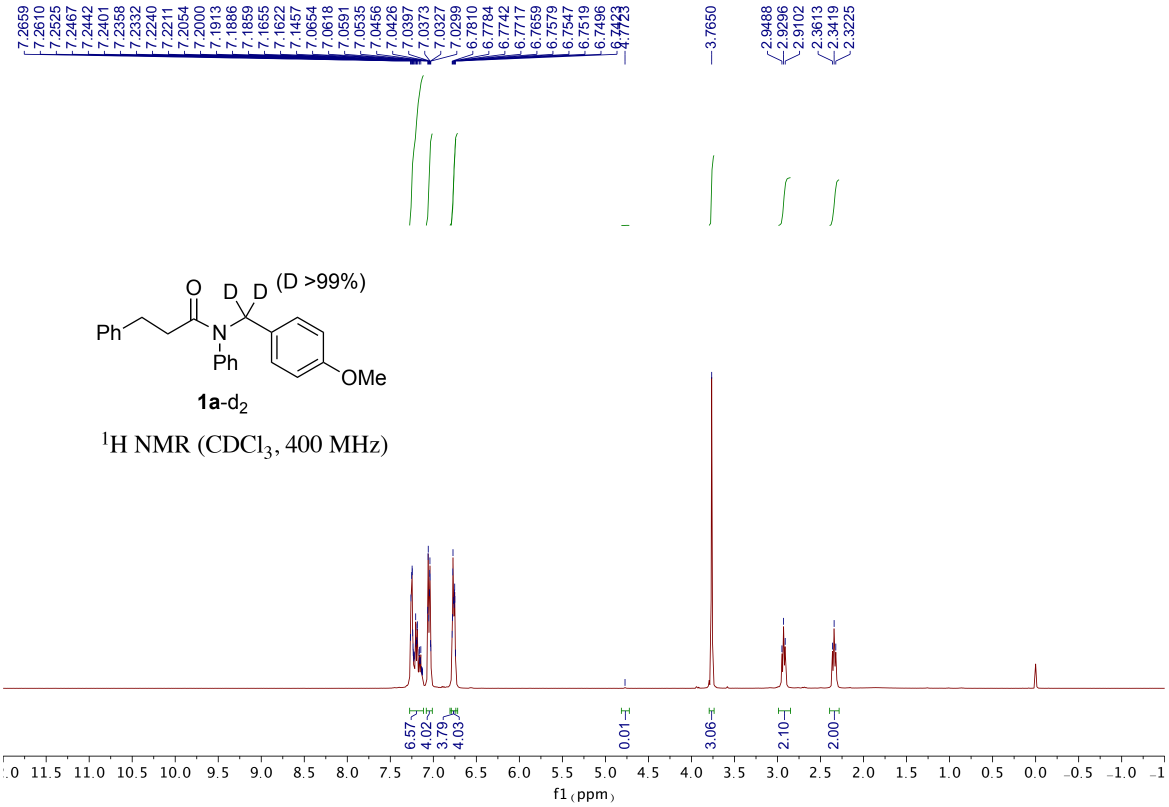
(D >99%)
1a-d2
1H NMR (CDCl3, 400 MHz)
f1 (ppm)

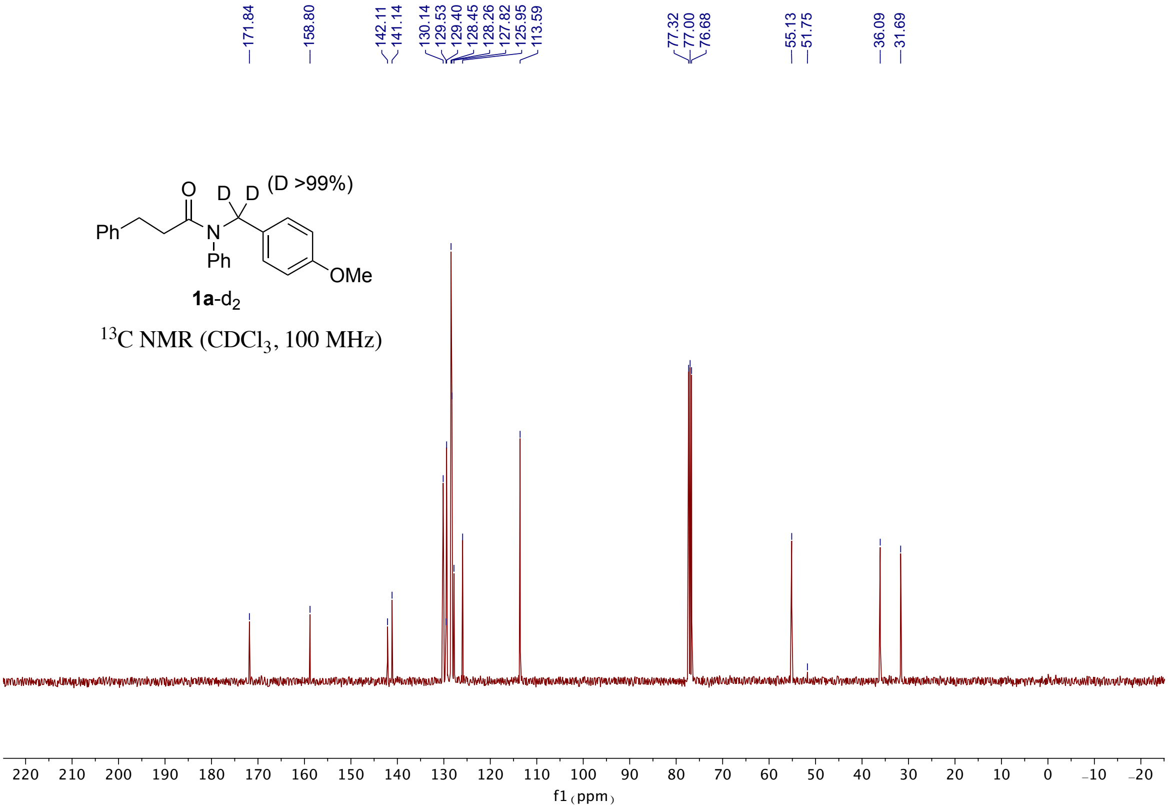
(D >99%)
1a-d2
13C NMR (CDCl3, 100 MHz)
f1 (ppm)

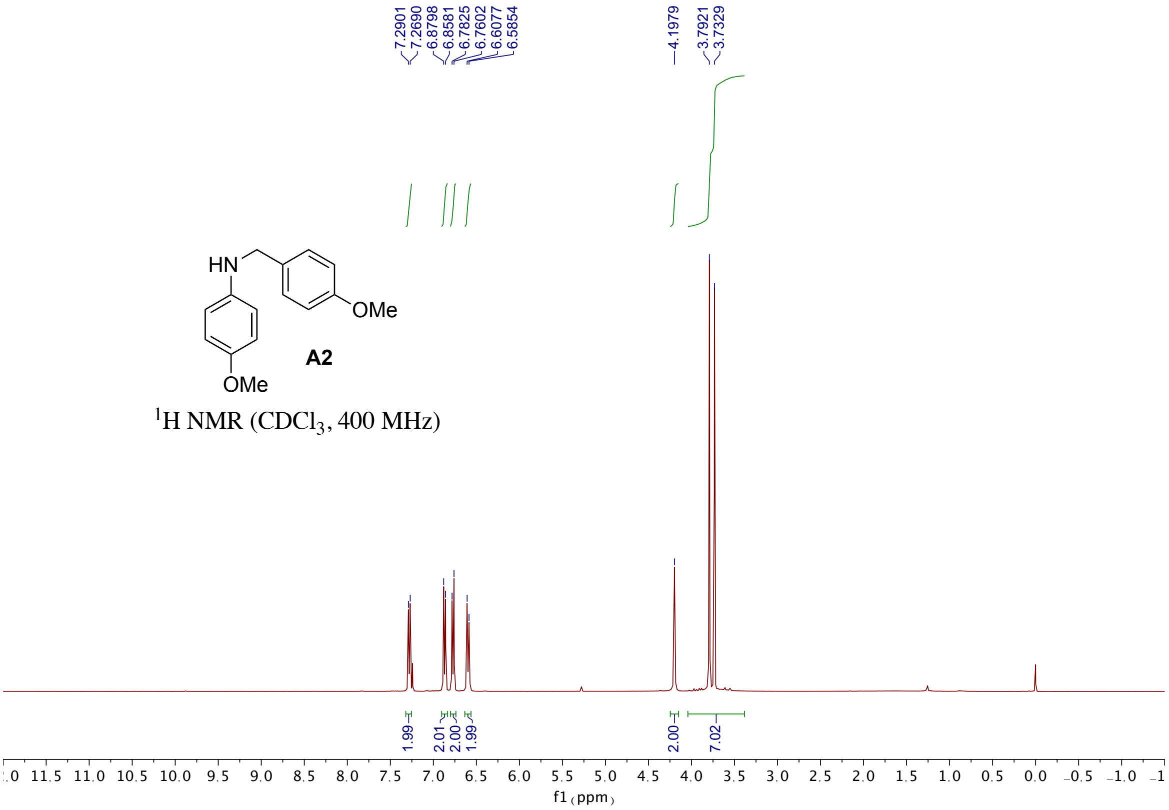

¹H NMR ($CDCl_3$, 400 MHz)

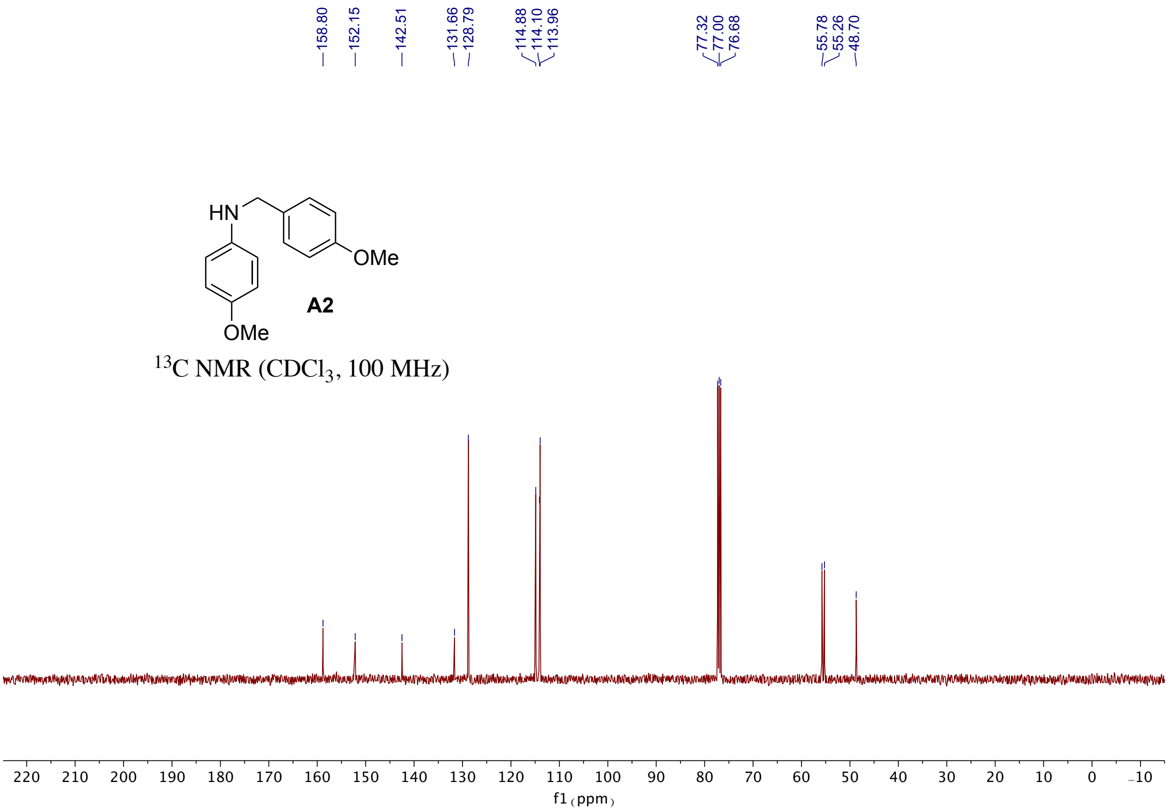

^{13}C NMR ($CDCl_3$, 100 MHz)

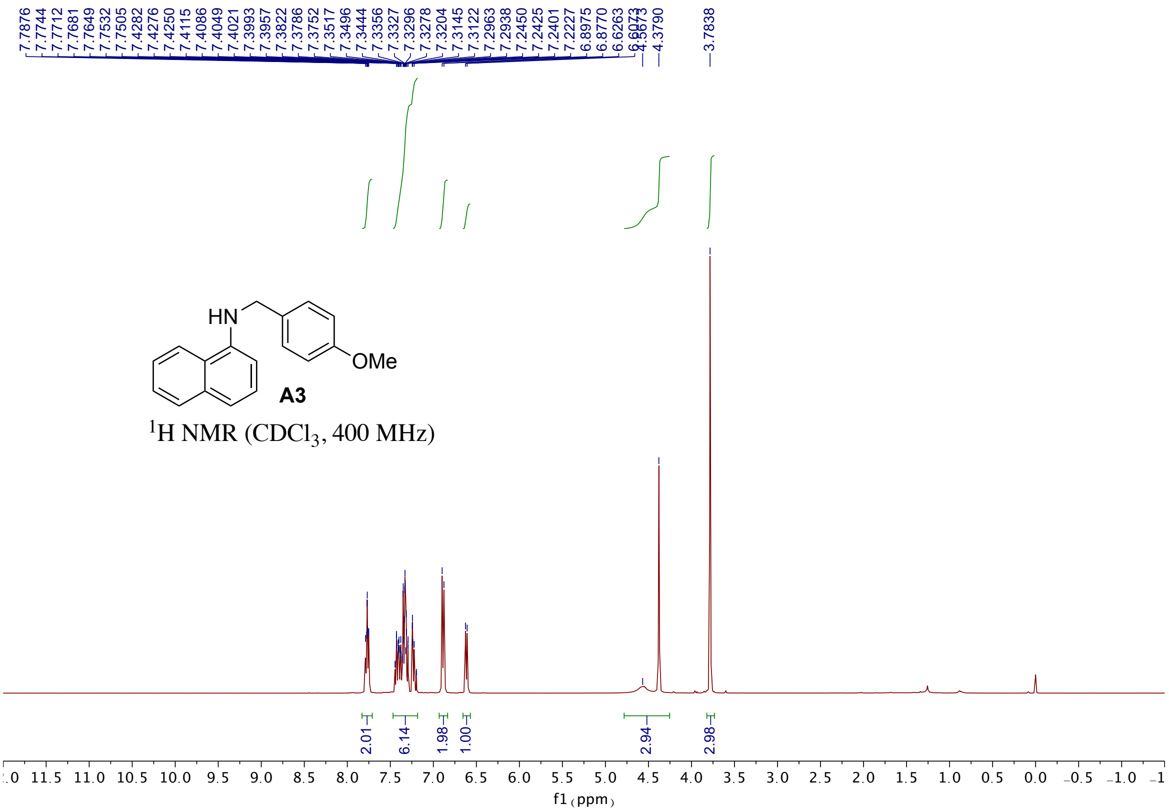
7.7876
7.7744
7.7712
7.7681
7.7649
7.7532
7.7505
7.4282
7.4276
7.4250
7.4115
7.4086
7.4049
7.4021
7.3993
7.3957
7.3822
7.3786
7.3752
7.3517
7.3496
7.3444
7.3356
7.3327
7.3296
7.3278
7.3204
7.3145
7.3122
7.2963
7.2938
7.2450
7.2425
7.2401
7.2227
6.8975
6.8770
6.6263
6.6073
4.5673
4.3790
3.7838
HN
OMe
A3
1H NMR (CDCl3, 400 MHz)
2.01
6.14
1.98
1.00
2.94
2.98
f1 (ppm)

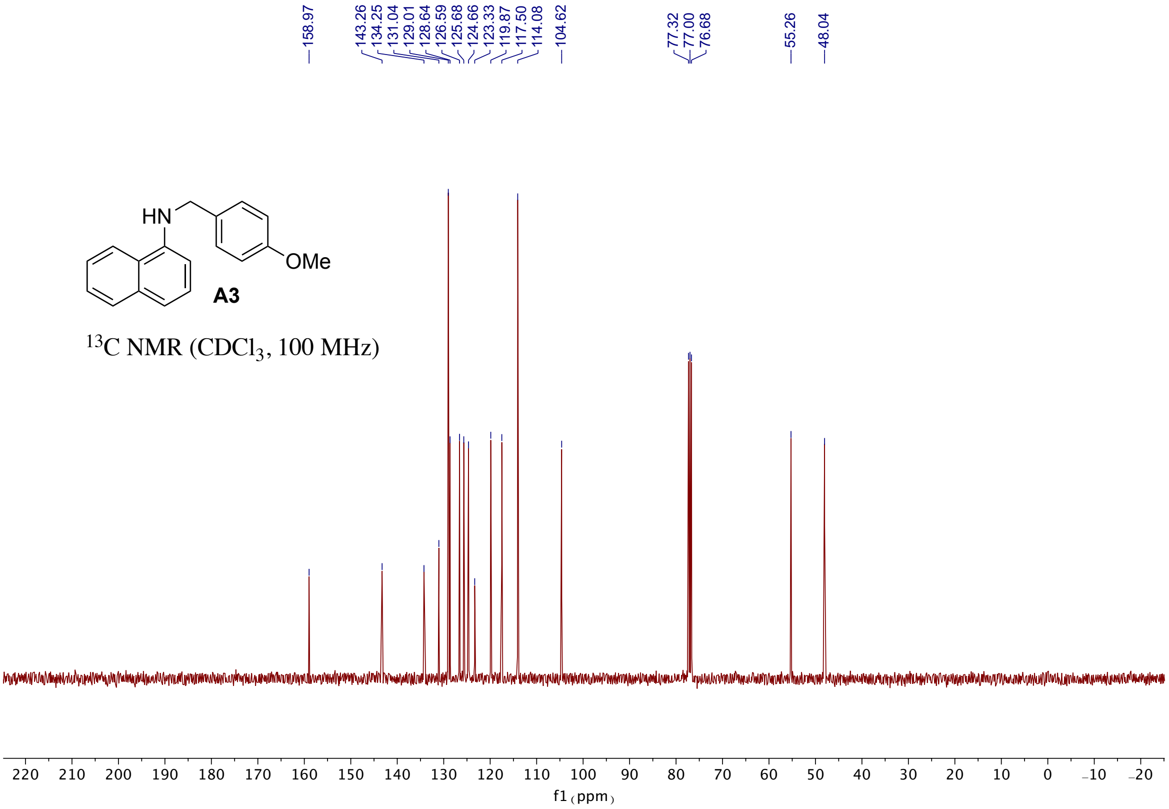
158.97
143.26
134.25
131.04
129.01
128.64
126.59
125.68
124.66
123.33
119.87
117.50
114.08
104.62
77.32
77.00
76.68
55.26
48.04
HN
OMe
A3
13C NMR (CDCl3, 100 MHz)
f1 (ppm)

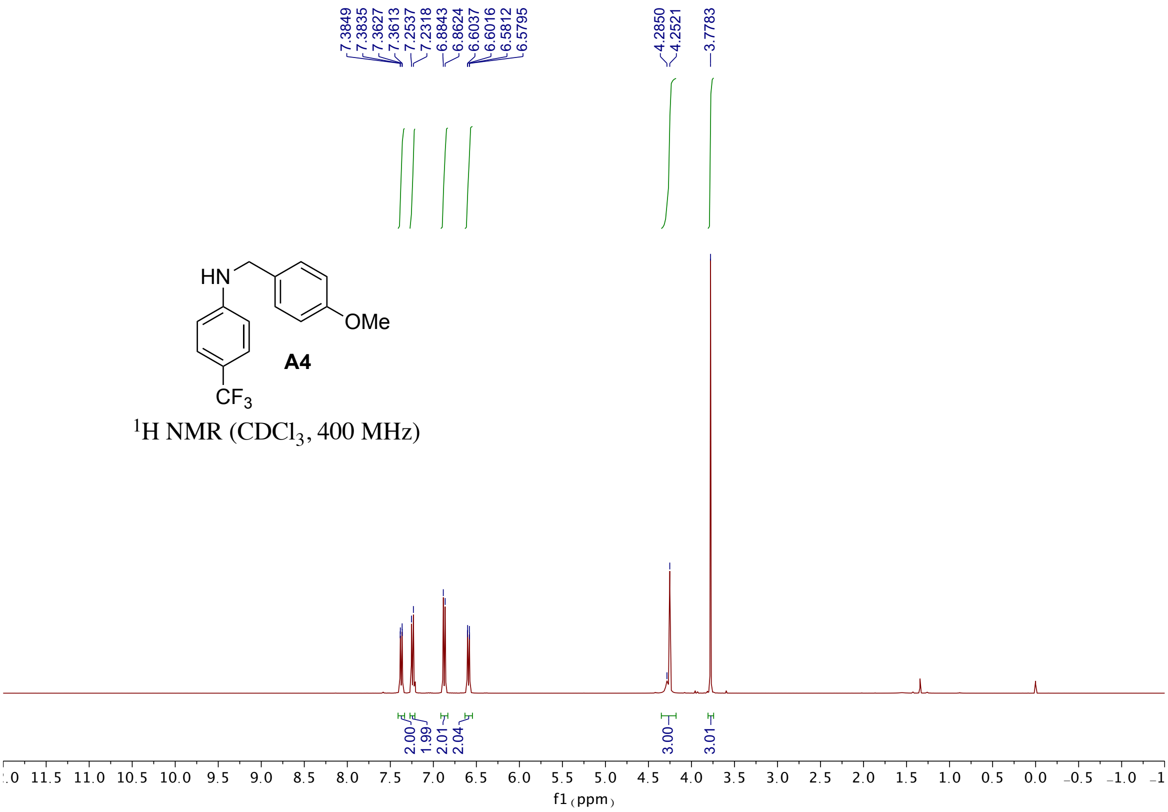

^{1}H NMR ($CDCl_3$, 400 MHz)

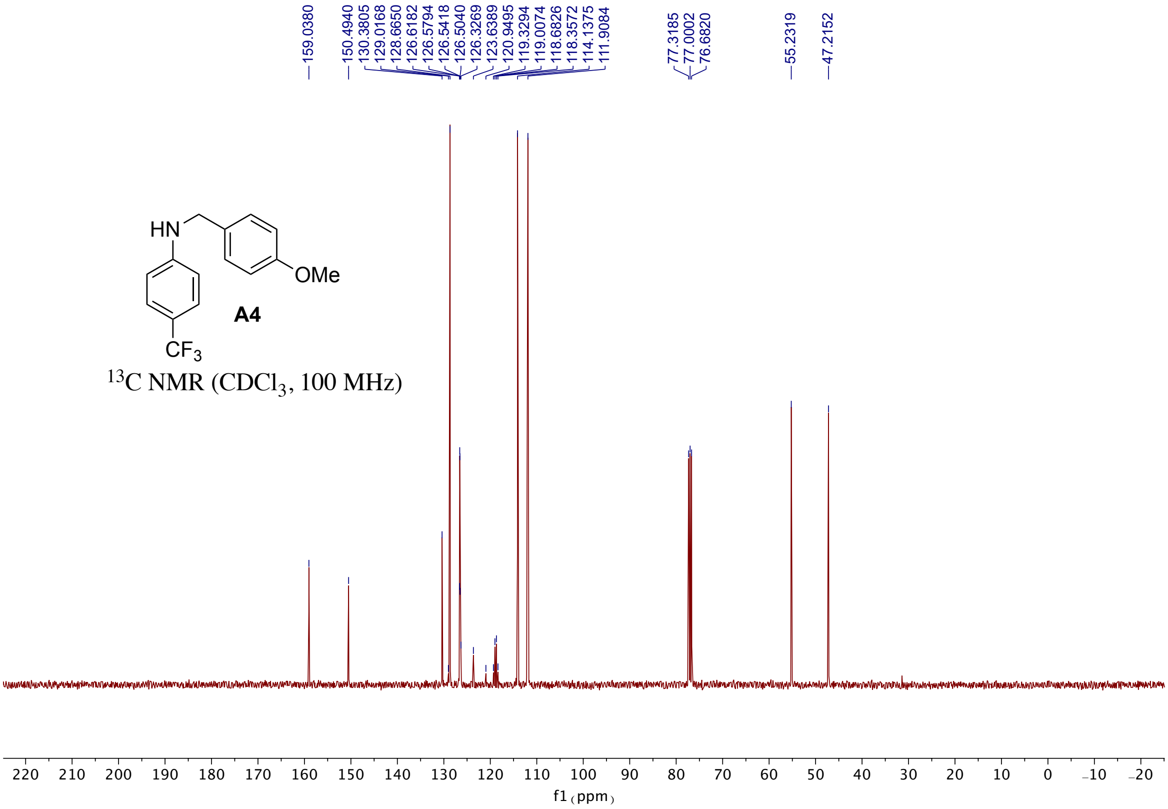

^{13}C NMR ($CDCl_3$, 100 MHz)

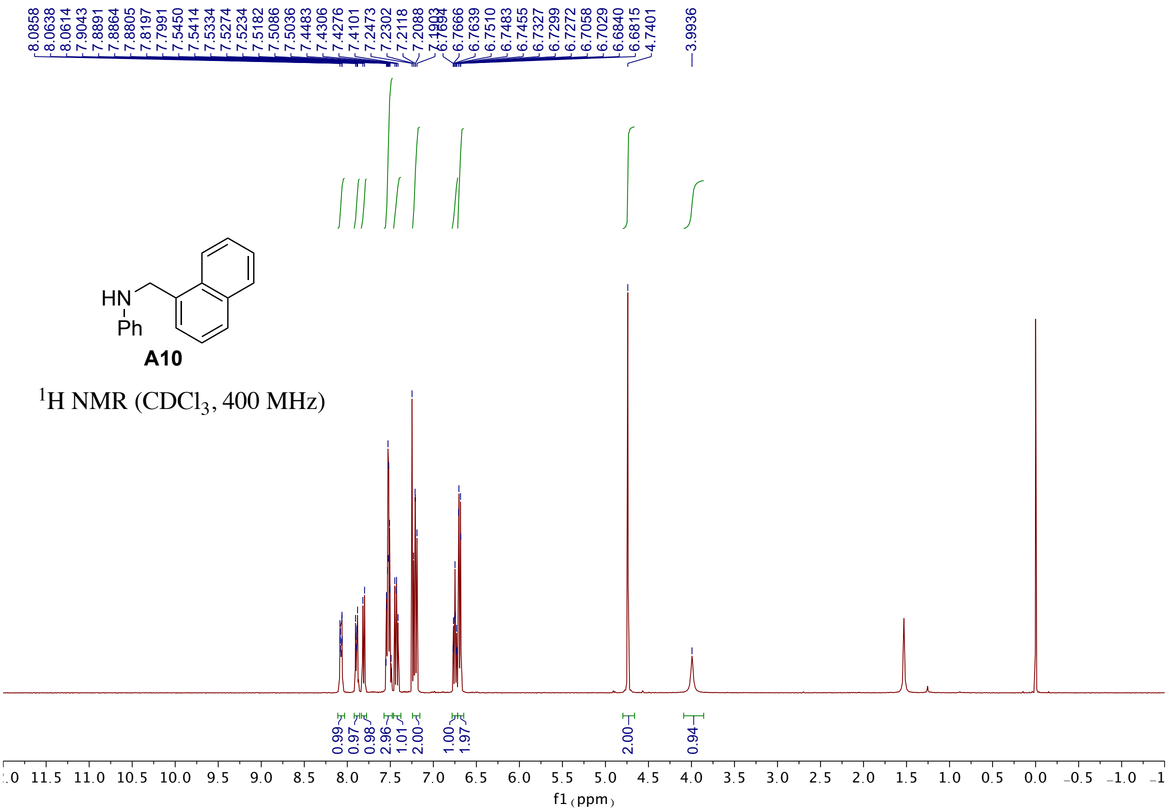
8.0858
8.0638
8.0614
7.9043
7.8891
7.8864
7.8805
7.8197
7.7991
7.5450
7.5414
7.5334
7.5274
7.5234
7.5182
7.5086
7.5036
7.4483
7.4306
7.4276
7.4101
7.2473
7.2302
7.2118
7.2088
6.7666
6.7639
6.7510
6.7483
6.7455
6.7327
6.7299
6.7272
6.7058
6.7029
6.6840
6.6815
4.7401
3.9936
HN
Ph
A10
1H NMR (CDCl3, 400 MHz)
0.99
0.97
0.98
2.96
1.01
2.00
1.00
1.97
2.00
0.94
f1 (ppm)

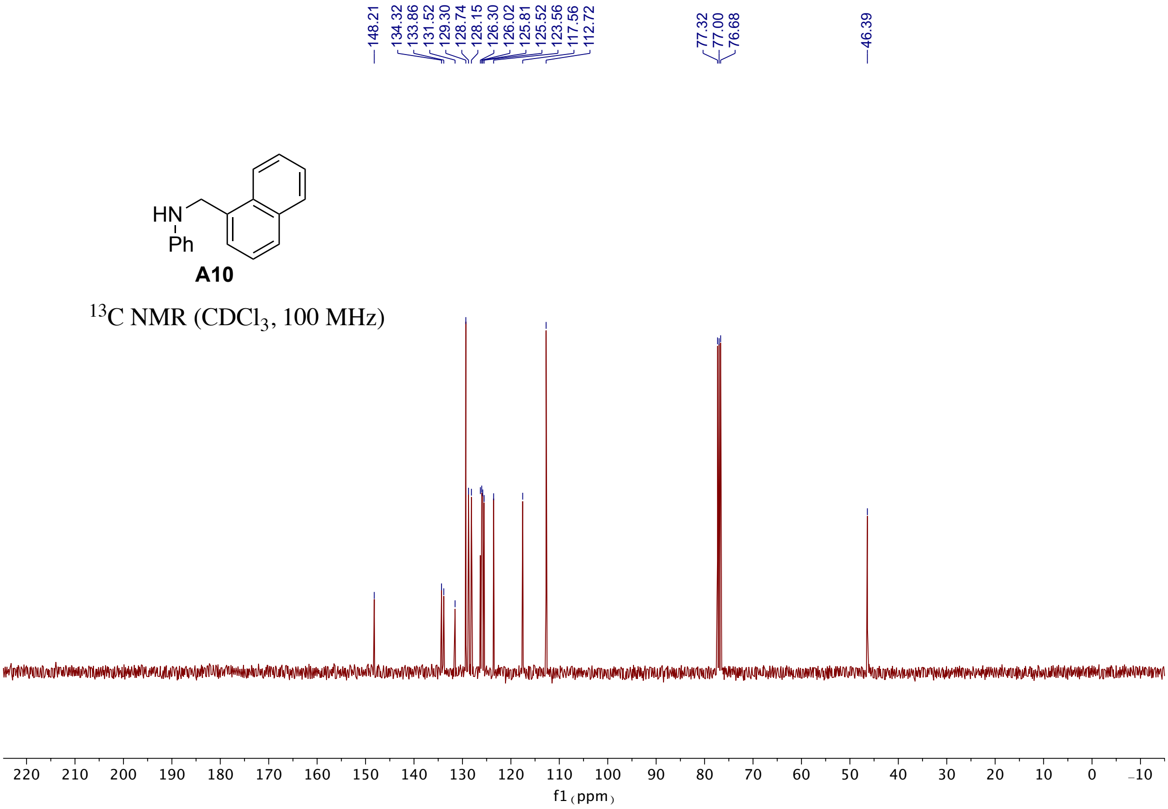
148.21
134.32
133.86
131.52
129.30
128.74
128.15
126.30
126.02
125.81
125.52
123.56
117.56
112.72
77.32
77.00
76.68
46.39
HN
Ph
A10
13C NMR (CDCl3, 100 MHz)
f1 (ppm)
